# Poster Session 2: Thursday, January 30, 2025 4:00 PM – 6:00 PM

**DOI:** 10.1002/pmf2.12026

**Published:** 2025-01-05

## 385 | “Keep Transfusing Until the Bleeding Stops”‐Massive Transfusion and Circulatory Overload in Obstetric Hemorrhage

Aaron W. Roberts^1^; Ghamar Bitar^1^; Ahmed Zaki Moustafa^2^; Carly Shahbazian^3^; Kate Drone^3^; Kendra Folh^3^; Sean C. Blackwell^1^

*^1^McGovern Medical School at UTHealth Houston, Houston, TX; ^2^Division of Maternal‐Fetal Medicine, Department of Obstetrics, Gynecology and Reproductive Sciences, McGovern Medical School at UTHealth Houston, University of Texas ‐ Houston, TX; ^3^Children's Memorial Hermann Hospital, Houston, TX*

4:00 PM ‐ 6:00 PM

**Objective**: Hemodynamic support and blood replacement during massive hemorrhage is critical to survival, but high‐volume massive transfusion (MTP) increases risk of transfusion associated circulatory overload (TACO). When faced with cardiac arrest from hemorrhagic shock restriction of fluid resuscitation may not be an option, and management of MTP goes beyond the moment bleeding is finally controlled. We aim to assess the risk and morbidity of TACO due obstetric hemorrhage managed with MTP.

**Study Design**: This retrospective cohort quality improvement study of parturients who suffered obstetric hemorrhage requiring large volume transfusion of blood products with more than four units of PRBC transfused as part of a MTP from June 2023 to July 2024 at our level IV center. Those cases with TACO were compared to those without.

**Results**: There were 5690 deliveries, 176 (3.1%) had PPH/MTP activation, of which 51 (28.9%) had > 4U MTP blood products transfused. The rate of TACO with MTP was 40% (N = 20/51). Placenta accreta spectrum alone was not a risk factor for TACO, neither was average estimated blood loss. Those with TACO had significantly more blood component volume/units transfused (Table 2). Patients with TACO had more diffuse intravascular coagulation (DIC) (75% vs 6%; p < 0.001), hemolysis (35% vs 3%; p = 0.002), ICU admission (75% vs 35%; p = 0.006), and acute kidney injury (50% vs 9%; p = 0.001). Post‐transfusion hemoglobin and nadir fibrinogen were similar between groups, despite more baseline anemia in the TACO group. The only maternal death in this study occurred in the TACO group.

**Conclusion**: High volume MTP in obstetric patients shows increased rates of TACO and other serious morbidity, mostly related to transfusion volume and development of DIC. There are few reliable predictors of morbidity. Those with TACO were not grossly more medically complicated at baseline, which underscores that TACO can happen to any parturient. Future investigation of techniques to limit hemorrhage, improve resuscitation quality, reduce volume administered, and mitigate sequalae of massive transfusion is warranted.

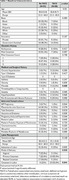

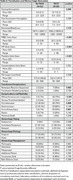

## 386 | Neighborhood Deprivation Impacted COVID‐19 Vaccination and Infection Rates Among Pregnant Patients

Abigail Bell^1^; Megan Foeller^1^; Candice Woolfolk^1^; Emily Dively, BSN^2^; Daniel Jackson^3^; Nandini Raghuraman^4^; Indira U. Mysorekar^5^; Jeannie C. Kelly^4^

*^1^Washington University School of Medicine in Saint Louis, St. Louis, MO; ^2^Washington University School of Medicine, St. Louis, MO; ^3^Mercy Hospital, St Louis, MO; ^4^Washington University School of Medicine in St. Louis, St. Louis, MO; ^5^Baylor College of Medicine, Houston, TX*

4:00 PM ‐ 6:00 PM

**Objective**: Area Deprivation Index (ADI) is strongly associated with obstetric outcomes but understudied during the COVID‐19 pandemic. We sought to evaluate the relationship between ADI and COVID‐19 outcomes among pregnant patients during the early pandemic.

**Study Design**: We performed a secondary analysis of a prospective longitudinal cohort study investigating the impact of COVID‐19 exposure in pregnancy on perinatal outcomes. Patients were recruited if they were pregnant between 12/23/20‐7/18/22 and serially evaluated for exposure to SARS‐CoV‐2 infection using serum antibody testing throughout pregnancy.  Address of residence at time of delivery was used to categorize patients into a low ADI group (≤ 50^th^ percentile, indicating low levels of deprivation) and a high ADI group (≥ 75^th^ percentile, indicating high levels of deprivation). Our primary outcome was COVID‐19 vaccination and infection during pregnancy.

**Results**: 306 patients were included. Those with higher ADI were younger (28 vs 32, p< 0.01), were less likely to be nulliparous (18% vs 38%, p = 0.02), had a higher average body mass index (35 vs 28, p = 0.01), and had higher rates of tobacco use (7% vs 2%, p = 0.03), asthma (17% vs 9%, p< 0.01), and depression (21% vs 17%, p = 0.01). There were notable differences in insurance type, race, and household income between groups (Table 1). Importantly, the high ADI group was less likely to receive COVID‐19 vaccination (24.2% vs 54%, p< 0.01), more likely to develop SARS‐CoV‐2 infection (59.3% vs 41.9%, p< 0.01), and more likely to be hospitalized due to COVID‐19 (3.9% vs 0%, p = 0.04, Table 2).

**Conclusion**: ADI differences were associated with vaccine hesitancy and COVID‐19 infection and severity. Overall, these findings highlight the complex interplay of factors impacting perinatal outcomes during the COVID‐19 pandemic, leading to two disparate perinatal experiences. Modifiable characteristics for both individuals and communities–such as tobacco use, obesity, and lack of vaccination–could serve as targets for interventions to improve perinatal outcomes.

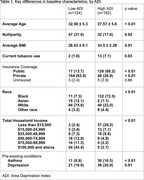

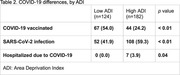

## 387 | Evaluating Indicators of Severe Maternal Morbidity based on Maternal OBCMI Score

Adina R. Kern‐Goldberger^1^; Megan R. Ansbro^2^; Antonio Bajan^2^; Elizabeth Raiff^3^; Justin R. Lappen^4^

*^1^Cleveland Clinic Lerner College of Medicine, Cleveland, OH; ^2^Cleveland Clinic Foundation, Cleveland, OH; ^3^Cleveland Clinic Ob/Gyn & Women's Health Institute, Cleveland, OH; ^4^Cleveland Clinic, Cleveland, OH*

4:00 PM ‐ 6:00 PM

**Objective**: Severe maternal morbidity (SMM) encompasses a diversity of adverse maternal medical outcomes that may be differentially triggered in patients with different risk profiles. This study evaluates incidence of component indicators of SMM by OBCMI level.

**Study Design**: This is a retrospective study of all deliveries > 20 weeks across a multi‐hospital academic health system from 1/1/2022‐6/30/2024. Patient clinical information was extracted from the electronic health record and OBCMI scores were constructed based on risk factors present at delivery and divided into brackets of low risk (0‐2), medium risk (3‐5), and high risk (>6) based on score distribution. Patient characteristics including OBCMI components and delivery admission SMM indicator variables were compared across brackets in univariable analysis using chi^2^.

**Results**: 28,812 deliveries were included: 67.4% with scores 0‐2, 21.0% with scores 3‐5, and 11.5% with scores > 6. There were significant differences in the incidence of each OBCMI component by score bracket (Table 1). Cardiomyopathy and pulmonary hypertension were the only comorbidities present exclusively in the highest risk bracket. Table 2 demonstrates the overall incidence of SMM by group: 0.5% in the lowest, 2.5% in the medium, and 8.1% in the highest risk bracket. Renal failure was the most common SMM indicator for all brackets, followed by disseminated intravascular coagulation in the 2 highest risk brackets and sepsis in the lowest risk bracket. Eclampsia co‐ranked as second most frequent SMM indicator in the highest risk bracket. Incidence of each SMM indicator across brackets was significantly different for all except ruptured aneurysm and vaso‐occlusive crisis.

**Conclusion**: SMM sub‐types occur at different relative frequencies across patient groups with different co‐morbidity‐based risk. Understanding these differences can shape patient counseling as well as target specific interventions to prevent sub‐types of SMM.

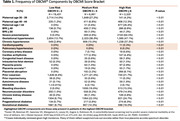

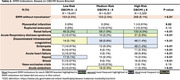

## 388 | Characterizing Sub‐Types of Severe Maternal Morbidity in Patients with Hypertensive Disorders of Pregnancy

Adina R. Kern‐Goldberger^1^; Megan R. Ansbro^2^; Antonio Bajan^2^; Elizabeth Raiff^3^; Justin R. Lappen^4^

*^1^Cleveland Clinic Lerner College of Medicine, Cleveland, OH; ^2^Cleveland Clinic Foundation, Cleveland, OH; ^3^Cleveland Clinic Ob/Gyn & Women's Health Institute, Cleveland, OH; ^4^Cleveland Clinic, Cleveland, OH*

4:00 PM ‐ 6:00 PM

**Objective**: Patients with preeclampsia are at increased risk for severe maternal morbidity (SMM). Elucidating risk for specific sub‐types of SMM can inform interventions to improve maternal outcomes. This study describes the incidence of SMM indicators in patients with hypertensive disorders of pregnancy (HDP). 

**Study Design**: This retrospective cohort study included all deliveries > 20 weeks across a multi‐hospital academic health system from 1/1/2022‐6/30/2024. Patient clinical information was extracted from the electronic health record and patients were classified as having gestational hypertension/mild preeclampsia (gHTN), severe preeclampsia (SPEC, inclusive of superimposed preeclampsia), or no HDP during the delivery admission. Patient characteristics classified according to OBCMI components and outcomes, including SMM sub‐types, were compared in univariable analyses using chi^2^ by hypertensive disorder of pregnancy status.

**Results**: 28,812 patients were included: 5,495 with gHTN (19.1%) and 2,432 with SPEC (8.4%). Patient co‐morbidities classified according to OB‐CMI components were different across groups for every condition except coronary artery disease, congenital heart disease, and autoimmune disease (Table 1). There were significant differences in incidence of preterm birth, cesarean delivery, ICU admission, extended postpartum length of stay (>5 days), and readmission, as well as non‐transfusion SMM, which ranged from 1.0% in patients without HDP to 1.8% in patients with gHTN and 9.1% in patients with SPEC (Table 2). Renal failure was the most frequent SMM indicator for all groups, occurring in 4.7% of patients with SPEC. Eclampsia was the second most common indicator in patients with SPEC, followed by disseminated intravascular coagulation (Table 2). 

**Conclusion**: SMM sub‐types have differential incidence based on HDP status. The high rate of renal failure in patients with SPEC could be an important target for care improvement or may reflect over‐coding of mild kidney injury, correction of which is an equally important quality target to accurately capture SMM.

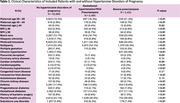

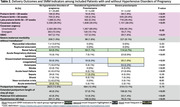

## 389 | Antenatal Risk Stratification Using the Obstetric Co‐Morbidity Index (OBCMI) during Prenatal Care

Adina R. Kern‐Goldberger^1^; Megan R. Ansbro^2^; Antonio Bajan^2^; Elizabeth Raiff^3^; Justin R. Lappen^4^

*^1^Cleveland Clinic Lerner College of Medicine, Cleveland, OH; ^2^Cleveland Clinic Foundation, Cleveland, OH; ^3^Cleveland Clinic Ob/Gyn & Women's Health Institute, Cleveland, OH; ^4^Cleveland Clinic, Cleveland, OH*

4:00 PM ‐ 6:00 PM

**Objective**: The obstetric comorbidity index (OBCMI) predicts risk for severe maternal morbidity (SMM) based on chronic and pregnancy‐specific conditions and was devised from comorbidities present at the time of delivery admission. This study assesses the ability of an OBCMI calculated during antenatal care to predict risk of SMM.

**Study Design**: This is an observational study of all deliveries > 20 weeks at 3 delivery hospitals within a large health system from 1/1/2022‐6/30/2024. Patient demographic and clinical information was extracted from the electronic health record (EHR) and OBCMI scores were calculated at 3 time points based on the clinical conditions identified from the EHR during that encounter: (1) initial prenatal visit, (2) 32‐week visit, and (3) delivery admission. Patient characteristics were evaluated in univariable analysis based on the presence of an OBCMI >3 at any of the 3 time points. Receiver‐operator characteristic (ROC) curves were constructed and compared for OBCMIs calculated at the initial and 32‐week visits versus the delivery admission OBCMI in terms of SMM prediction.

**Results**: 28,812 deliveries were included and 9,625 patients (33.4%) had an OBCMI > 3 at any point in pregnancy, with significant differences in all examined patient characteristics based on OBCMI score [Table]. The incidence of SMM was 1.8% (N = 525). The prediction of SMM by OBCMI scores calculated during antenatal care at both time points was significantly inferior, with area‐under‐the‐curve (AUC) of 0.59 (95% CI 0.57–0.61) for initial prenatal visit and 0.58 (95% CI 0.55–0.610) for the 32‐week visit, compared to AUC of 0.81 (95% CI 0.79–0.83) for delivery admission OBCMI (p < 0.01 for all comparisons) [Figure].

**Conclusion**: While the OBCMI reliably predicts SMM when calculated based on conditions documented at delivery, it performs poorly as a tool for antenatal risk stratification. This is likely multifactorial due to both EHR under‐coding at antenatal visits and the development of clinically significant risk factors late in pregnancy. Better tools for maternal risk prediction prior to delivery are essential.

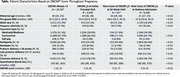

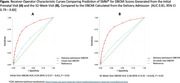

## 390 | Employment Experiences of High‐Risk Obstetric Patients: A Post‐Pregnant Worker's Fairness and PUMP Act Cohort Study

Adwoa A. Baffoe‐Bonnie^1^; Shakthi Unnithan^1^; Tracy Truong^1^; Thelma Fitzgerald^1^; Barvina Toledo^1^; Danielle Lanpher^1^; Kelley E. C. Massangele^2^; Maya Jackson^3^; Sarahn M. Wheeler^1^

*^1^Duke University School of Medicine, Durham, NC; ^2^National Diaper Bank Network, Diaper Bank of North Carolina, Hillsborough, NC; ^3^Mobilizing African American Mothers Through Empowerment⁠, Inc, Durham, NC*

4:00 PM ‐ 6:00 PM

**Objective**: The 2022 Providing Urgent Maternal Protections (PUMP) Act and the 2023 Pregnant Workers Fairness Act have recently changed employment laws in pregnancy. We evaluated employment benefits, knowledge of employment laws, and racial differences among hourly wage and salaried workers receiving high‐risk obstetric care.

**Study Design**: Self‐identified non‐Hispanic Black (NHB) and White (NHW) patients at a single high‐risk OB clinic within a Southeastern tertiary academic medical center were surveyed. We excluded major fetal anomalies, stillbirth or fetal demise. Participants completed surveys on work hours and duties, employment benefits at a single timepoint between 28 weeks’ gestation to 12 weeks postpartum.  Participants also reported if they received any information about employments laws or employment discrimination resources and the source of that information. Results were analyzed using descriptive statistics with SAS 9.4 (SAS Institute Inc., Cary, NC).

**Results**: 50/86 participated including 26 NHB and 24 NHW. Of these, 28 (56%) had hourly wage jobs, and 22 (44%) held salaried positions (Table 1). Thirty‐seven (74%) had health insurance, and 31 (62%) had paid maternity leave. NHB patients were more often hourly wage earners compared to NHW patients (19/26, 73% vs. 9/24, 37.5%). Although mandated by the PUMP Act, only 13 (26%) patients in the total cohort and 2 (7.1%) hourly workers reported having private lactation facilities in their workplaces. 33 (66%) received information about Family Medical Leave Act (FMLA), while fewer knew about the Pregnant Worker's Fairness Act (8, 16%) and the PUMP Act (8, 16%). Some participants who did not receive information expressed a desire for more information on these laws (Table 2).

**Conclusion**: While most participants had health insurance and paid maternity leave, a significant gap existed in workplace lactation support and knowledge of employment laws. These results highlight need for patient education about employment laws in pregnancy.

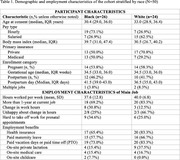

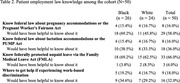

## 391 | Maternal and Neonatal Substance Screening Discordance After Implementation of an Obstetric Substance use Screening Protocol

Adwoa A. Baffoe‐Bonnie; Siera Lunn; Janea Cato; Lena Fried; Leyi Sun; Tracy Truong; Sarahn M. Wheeler; Jennifer J. M. Cate

*Duke University School of Medicine, Durham, NC*

4:00 PM ‐ 6:00 PM

**Objective**: To evaluate cases of discordance between maternal and neonatal drug screening before and after implementing an obstetric substance use screening protocol.

**Study Design**: This was a retrospective cohort study performed at a single tertiary care center using electronic health record data with pre‐ (July 1, 2020‐June 9, 2021) and post‐intervention (June 10, 2021‐May 31, 2022) design after implementation of an obstetric substance use screening protocol incorporating pre‐specified indications for maternal biologic testing and universal maternal consent. Concordances and discordances in neonatal and maternal drug screening were reported as frequency (percentage) in each time period. and compared by time period using test of two proportions. In the cohort of neonates with drug test and no maternal drug test, race, ethnicity, payor status, admission to the NICU, readmission, and neonate UDS results were compared by time period using Fisher's exact tests.

**Results**: 6552 neonates were born in the study period with 3163 (48.3%) pre and 3389 (51.7%) post implementation. 349/6552 (5.3%) neonates were drug screened (195/3163 [6.2%] pre vs. 154/3389 [4.5%] post, p = 0.004). 172 were ordered in the absence of a maternal test (63/195 [32.3%] pre, 109/154 [70.8%] post; p< 0.001) (Figure). Among 172 drug screens ordered in the absence of maternal tests, 80 were positive (24/63 [38.1%] pre, 56/109 [51.4%] post, p = 0.093). The number of neonates testing positive for specific substances did not significantly differ between the pre‐ and post‐periods (Table).

**Conclusion**: After implementing the obstetric substance use screening protocol, neonatal substance use screening in the absence of maternal screening occurred more frequently post‐intervention than pre‐intervention despite similar rates of positive tests and substances for which neonates tested positive. These findings suggest a need for further investigation into the factors driving this increase in discordant testing and the potential implications for both neonatal and maternal care and support strategies in the future.

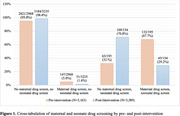

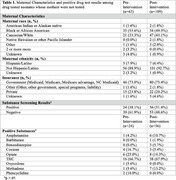

## 392 | Prior Delivery History and Risk of Placenta Accreta Spectrum

Alesha M. White^1^; Ariana Weiss^1^; Jessica E. Pruszynski^2^; Christina L. Herrera^1^

*^1^University of Texas Southwestern Medical Center, Dallas, TX; ^2^University of Texas Southwestern, Dallas, TX*

4:00 PM ‐ 6:00 PM

**Objective**: Previous cesarean delivery, prior uterine procedures, in vitro fertilization and placenta previa are known risk factors for placenta accreta spectrum (PAS). Further studies investigating prior delivery characteristics and risk of PAS are limited. The objective of this study was to describe additional risk factors in prior deliveries associated with the development of PAS.

**Study Design**: This was a case‐control study at a single institution from June 2009 through June 2022 that included patients with prenatal suspicion of PAS based on imaging and confirmed at the time of delivery. Cases were matched to controls based on age, race, number of cesarean deliveries and prior vaginal birth. Prior delivery characteristics included preterm prelabor rupture of membranes, preterm birth, chorioamnionitis, delivery stage, uterine incision, bladder flap closure, peritoneal closure and manual placental extraction. Location of placenta in relation to the internal os on second trimester sonogram in the prior pregnancy was also analyzed. A conditional logistic regression model was used, that accounted for 3:1 matching, to compare cases to controls. A p‐value less than 0.05 was significant.

**Results**: 55 cases were matched to 165 controls. There were no differences in maternal characteristics or comorbidities. Cases were more likely to have experienced preterm birth (OR 3.00 95% CI 1.37‐6.57) and have had a manual placenta extraction (OR 5.25 95% CI 1.54‐17.93) in a prior delivery. Placental location in a prior pregnancy was not significantly different between cases and controls (Table).

**Conclusion**: Preterm birth and manual extraction of the placenta in a prior pregnancy appear to be additional risk factors for development of PAS in future pregnancies. Further multicentered studies with a larger population are needed to further understand other risk factors for PAS.

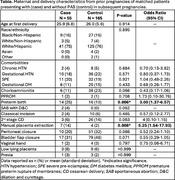

## 393 | Postpartum EPDS Scores in Prenatally Suspected Placenta Accreta Spectrum

Alesha M. White^1^; Ellery R. Cohn^1^; Mishel Malik^1^; Jessica E. Pruszynski^2^; Catherine Y. Spong^1^; Christina L. Herrera^1^

*^1^University of Texas Southwestern Medical Center, Dallas, TX; ^2^University of Texas Southwestern, Dallas, TX*

4:00 PM ‐ 6:00 PM

**Objective**: Placenta accreta spectrum (PAS) is a life‐threatening condition and may impact mental health. Peripartum hysterectomy is also independently associated. This study's objective was to assess the association of prenatally suspected PAS and Edinburgh Postnatal Depression Scale (EPDS) scores.

**Study Design**: This was a case‐control study of singleton pregnancies from 2010 to 2024. Cases with prenatal suspicion of PAS based on imaging were matched to controls by age, parity, cesarean delivery and whether the cesarean was scheduled. Those with fetal anomalies or stillbirth after 24 weeks gestation were excluded. Postpartum EPDS scores were compared with patients with prenatal suspicion of PAS by hysterectomy status using the Kruskal‐Wallis test and to matched controls using the Wilcoxon signed rank test.

**Results**: Of 184 cases matched 101 had available EPDS scores. This was significantly different from matched controls where 150 scores were available (55% vs 82% %, p< 0.001). Sixty‐two (61.4%) of cases with EPDS scores underwent hysterectomy. Median EPDS scores were not statistically higher (3 (1‐7) vs 3 (1‐4), p = 0.114) comparing cases to matched controls. Among cases, patients that underwent hysterectomy had significantly higher median EPDS scores (4 (2‐8) vs 2 (0‐5), p = 0.049). The percentage of EPDS scores that would require intervention and referral (score of ≥13) were not significantly different between cases and controls (p = 0.206), however all five cases with EPDS score ≥13 had a hysterectomy compared to none in the controls (p = 0.048).

**Conclusion**: Patients with prenatal suspicion of PAS were less likely to attend their routine postpartum visit. Compared to matched controls, these patients did not have higher postpartum EPDS scores following delivery but performance of hysterectomy increased EPDS score significantly. Providing counseling services in pregnancy and enhanced postpartum interventions may be of benefit.

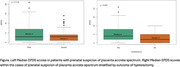

## 394 | Impact of Antenatally Suspected Uterine Window on Maternal and Neonatal Outcomes

Alexa L. Cohen^1^; Carly Pickett^2^; Taylor Douglas^2^; Grace M. Lee^2^; Chloe Porigow^2^; Pe'er Dar^2^; Georgios Doulaveris^2^

*^1^University of Miami Health System, Miami, FL; ^2^Montefiore Medical Center, Albert Einstein College of Medicine, New York, NY*

4:00 PM ‐ 6:00 PM

**Objective**: Prenatal sonographic suspicion for uterine window at a prior cesarean scar niche may impact mode and timing of delivery. We aimed to investigate maternal and neonatal outcomes in patients with prenatal suspicion of uterine window.

**Study Design**: Retrospective cohort study of all patients with a sonographic diagnosis of a uterine window at a tertiary referral academic institution from 2014‐2024. Uterine window was defined as lower uterine myometrial segment >10mm in length and thickness < 2mm on 2nd trimester transvaginal ultrasound. Patients were compared to controls with a history of cesarean delivery (CD) without a uterine window, electing a repeat CD. All study patients were planned for repeat CD at early term (37 weeks). Primary outcome was a composite adverse maternal outcome (uterine rupture, blood loss >1.5L, transfusion, intensive care unit (ICU) admission, need for vasopressors and hysterectomy). Secondary outcomes included APGAR < 7, NICU admission and a composite neonatal morbidity outcome (sepsis, respiratory distress, and IVH or NEC). Multivariable logistic regression analysis was performed.

**Results**: Study included 103 patients with mid‐gestation suspicion of uterine window and 103 matched controls. There was no significant difference in age, race/ethnicity, BMI, or parity between groups. Uterine rupture occurred in two (1.94%) study patients and one (0.9%) control. Delivery on the planned date was similar between groups (67.0% vs 69.9%, p = 0.6). Actual delivery occurred earlier for study patients (36.3 ±0.2 weeks vs 38.2 ±0.2 weeks, p< 0.01). Maternal composite adverse outcome was higher in study patients (22.3% vs 11.7%, p = 0.04). Study neonates had similar APGAR scores, but were more likely to be admitted to NICU (20.4% vs 9.7%, p = 0.03) and had higher morbidity (16.5% vs 6.8%, p = 0.02), compared with controls. 

**Conclusion**: Patients with 2^nd^ trimester sonographic suspicion for uterine window are at increased risk for iatrogenic earlier delivery, and higher maternal and neonatal morbidity. Possible benefits of earlier scheduled repeat CD should be weighed against the increased neonatal risks.

## 395 | Isolated Large Fetal Abdominal Circumference is Associated with Adverse Perinatal Outcomes in Non‐Diabetic Pregnancies

Alexa L. Cohen^1^; Eliane Shinder^2^; Grace M. Lee^2^; Chloe Porigow^2^; Pe'er Dar^2^; Georgios Doulaveris^2^

*^1^University of Miami Health System, Miami, FL; ^2^Montefiore Medical Center, Albert Einstein College of Medicine, New York, NY*

4:00 PM ‐ 6:00 PM

**Objective**: The Society of Maternal Fetal Medicine currently recognizes an association between large for gestational age (LGA), defined as estimated fetal weight (EFW) >90%, and adverse perinatal outcomes. Our study aimed to assess the impact of isolated large fetal abdominal circumference (AC) on maternal and neonatal outcomes in non‐diabetic pregnancies.

**Study Design**: Retrospective cohort analysis of patients at an urban academic institution who had ultrasonography between 35‐36 weeks to estimate fetal weight. Those with multiple gestation, pregestational or gestational diabetes, prior cesarean delivery (CD) and contraindication to vaginal delivery were excluded. Outcomes were compared between three groups: a) appropriate for gestational age (AGA): AC and EFW between 10‐90%, b) Isolated large AC: AC >90% and c) LGA: EFW >90%. Groups were matched by parity and gestational age by ultrasound. Primary outcome was primary CD. Secondary outcomes included shoulder dystocia, hemorrhage >1.5L, composite adverse maternal outcome (transfusion, infection, ICU admission, intubation, and mortality) and composite adverse neonatal outcome (APGAR < 7 at 5 min, NICU admission, neonatal morbidity and mortality). Analysis was performed using multivariate logistic regression.

**Results**: 765 patients were included: 255 in each group. There was no difference in age, parity, body mass index and hypertension among groups. Macrosomia (>4000 grams) at birth was confirmed in 2.4% of AGA, 12.5% of large AC and 38.0% of LGA group (p< 0.01). Patients with isolated large AC were more likely to have primary CD (aOR 2.41, 95%CI 1.56‐3.71, p< 0.01), hemorrhage (aOR 2.76, 95%CI 1.19‐6.45, p = 0.01) and composite adverse neonatal outcome (aOR 2.98, 95%CI 1.71‐5.21, p< 0.01), when compared to AGA group. Outcomes were similar between large AC and LGA groups.

**Conclusion**: Isolated large AC with normal EFW is associated with an increased risk of primary CD and adverse perinatal outcomes in non‐diabetic pregnancies. Including isolated large AC in the definition of LGA may improve counseling, delivery planning, and perinatal management.

## 396 | Accuracy of Point of Care Urine Drug Screen Testing in Pregnancies Complicated by Opioid Use

Alexander M. Harrison, Sr.^1^; Cynthia Cockerham^2^; Nancy Hendrix^3^; Asmita Shrestha^2^; Wendy Whitley^2^; Barbara V. Parilla^2^

*^1^University Of Kentucky, Lexington, KY; ^2^University of Kentucky, Lexington, KY; ^3^University of Kentucky, University of Kentucky, KY*

4:00 PM ‐ 6:00 PM

**Objective**: To evaluate the accuracy of point of care (POC) versus the gold standard quantitative urine drug screen in a comprehensive perinatal substance treatment program. 

**Study Design**: This study was from a longitudinal cohort of women receiving prenatal care through a comprehensive perinatal substance use treatment program between 2014 and 2024. We assessed participants who had both a POC and a quantitative urine drug screen (UDS) result during the same prenatal visit. Postpartum visits were excluded.

**Results**: Data was available for 2079 samples collected for a POC analysis and a quantitative drug screen, and the results were compared. We looked specifically at the accuracy of identifying opioids as most of our patients are on medication for opioid use disorder (MOUD). A true positive was defined as the POC matching the gold standard urine quantitative result. 78.2% (76.3, 79.9%) of the time the POC test yielded results matching the quantitative assessment. 21.8% of the time the POC was positive for an opiate when the quantitative was negative either for an opiate or for that specific opiate. The test yielded a positive predictive value of 79.5% (78.7, 80.2%) and a negative predictive value of 64.9% (58.2, 71.0%). 

**Conclusion**: POC is not an accurate tool for use in pregnancy when evaluating compliance with MOUD. False positive and false negative results can adversely affect decisions that impact the patient and fetus. This can result in loss of trust in the medical system, decreased likelihood to engage in care, and poorer outcomes. More accurate quantitative tests should be utilized for evaluating compliance with treatment.

## 397 | Increased Buprenorphine Dosing Frequency Was Not Associated With An Increase In Neonatal Opioid Withdrawal Syndrome

Alexander M. Harrison, Sr.^1^; Cynthia Cockerham^2^; Katherine Vignes^3^; Asmita Shrestha^2^; Ronald Clarkson^2^; Gregory Hawk^2^; John O'Brien^2^; Barbara V. Parilla^2^

*^1^University Of Kentucky, Lexington, KY; ^2^University of Kentucky, Lexington, KY; ^3^Oschner, New Orleans, LA*

4:00 PM ‐ 6:00 PM

**Objective**: Pregnancy alters the pharmacokinetics of buprenorphine with the need for increased dosing frequency to stay in the therapeutic range. We sought to evaluate whether increased dosing frequency is associated with an increased risk of Neonatal Opioid Withdrawal Syndrome (NOWS).

**Study Design**: This study was from a longitudinal cohort of pregnant persons receiving prenatal care through a multidisciplinary program for substance use disorder (SUD) between 2015 and 2024. We compared outcomes between patients prescribed once‐a‐day (QD) dosing verses twice a day (BID) or greater dosing of buprenorphine. Women not taking buprenorphine at delivery were excluded as well as those with less than 6 visits. Fisher's exact and two‐sample t‐tests were used for our statistical analysis.

**Results**: 367 patients were included, with 266 (72.5%) receiving QD dosing versus 101 (27.5%) dosed BID or more.  Demographics were similar between groups including tobacco use. There was no significant difference in gestational age at delivery (38.4 +/‐ 2.2, 38.1 +/‐ 1.9;, p = 0.25), rate of preterm birth (9.4%, 12.9%, p = 0.34), birthweight percentile (37.6 +/‐ 25.5, 32.5 +/‐ 22.7, p = 0.08), rate of NOWS (48.9%, 45.5%, p = 0.68), length of hospital days (11 [6 to 20.2], 13 [6 to 20.0], p = 0.37) or NOWS treatment days (15 [10 to 20], 14 [11 to 24], p = 0.28).

**Conclusion**: An increase in dosing frequency was not associated with an increased risk of NOWS. Our data suggests that providers may be able to dose buprenorphine based on patient's needs (cravings and withdrawal symptoms) without concern of increasing the risk of NOWS.

## 398 | Unplanned Cesarean Delivery is Associated with Higher Risk of Postpartum Depression and Engagement in Care

Alexandra N. Mills^1^; Nicola F. Tavella^2^; Rachel Meislin^2^; Chelsea A. DeBolt^2^

*^1^Division of Maternal‐Fetal Medicine, Icahn School of Medicine at Mount Sinai, New York, NY; ^2^Icahn School of Medicine at Mount Sinai, New York, NY*

4:00 PM ‐ 6:00 PM

**Objective**: Postpartum depression (PPD) is a serious pregnancy complication, affecting 10‐15% of postpartum people. Studies suggest an association between method of delivery (MOD) and PPD. Intrapartum experiences may contribute to PPD development and affect patient engagement in healthcare after birth through attendance at the six week postpartum visit (PPV). This study aims to interrogate the associations between MOD and PPD as well as PPV attendance in a large national pregnancy dataset.

**Study Design**: This was a retrospective study using the Pregnancy Assistance Monitoring Systems (PRAMS) national dataset by the United States Centers for Disease Control and Prevention (CDC). The cohort includes patients for whom MOD, demographic, and outcome data was available. The outcome of interest was PPD (via survey‐administered PHQ‐2 and patient‐reported via survey), and attendance at the 6‐week PPV (patient‐reported via survey). The predictor variable was MOD: vaginal delivery (VD), planned cesarean delivery (CD), or unplanned CD. Chi‐squared tests examined differences between groups and logistic regression models adjusting for maternal factors estimated adjusted odds ratios (aOR) of outcomes given MOD.

**Results**: This study included 6078 pregnancies: 3481 VDs (57.3%), and 2597 CDs (42.7%). Of the CDs, 1066 were planned CDs while 1531 were unplanned CDs. These groups differed significantly by maternal age, years of education, and household income. While those who delivered by planned CD did not have increased odds of PPD compared to VD, those who delivered by unplanned CD had increased odds of  PPD as compared to VD (aOR 1.19 (1.04, 1.44)), and were significantly more likely to attend a postpartum visit (aOR 1.22 (1.05, 1.56)).

**Conclusion**: This study found that participants who delivered by unplanned CD but not planned CD are at increased odds of developing PPD and attending PPV compared to VD. Future work should focus on the physical and psychosocial stressors of an unplanned CD that may contribute to PPD development and postpartum care engagement.

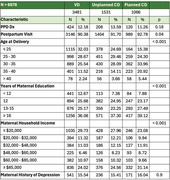

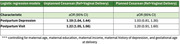

## 399 | Risks Factors of Perinatal Morbidity After Instrumental Delivery Among Nulliparous Women

Alexiane LOUIS^1^; Paul BERVEILLER^2^; Bertrand GACHON^3^; PATRICK ROZENBERG^4^; ANNE ROUSSEAU^5^

*^1^CH Poissy Saint Germain en laye, Poissy, Poissy, Ile‐de‐France; ^2^CH Poissy Saint Germain en Laye, Poissy, Ile‐de‐France; ^3^CH Poitiers, Poitiers, Poitou‐Charentes; ^4^UVSQ, Neuilly sur seine, Ile‐de‐France; ^5^UVSQ, Poissy, Ile‐de‐France*

4:00 PM ‐ 6:00 PM

**Objective**: Instrumental vaginal delivery is a common practice, especially among nulliparous women, and may increase the risk of neonatal morbidity. However, there is a paucity of recent data regarding the risk factors for neonatal morbidity in this context. The goal is to identify risk factors of neonatal morbidity during instrumental vaginal deliveries among nulliparous women.

**Study Design**: This is an ancillary study of the prospective cohort study INSTRUMODA, the aim of which was to evaluate the protective effect of episiotomy on anal sphincter injury among nulliparous women during instrumental vaginal delivery. This study was conducted in 111 maternity units in France between April 2021 and March 2022. Instrumental deliveries were included from 34 weeks of gestation. Our primary outcome was a composite criterion of neonatal morbidity, including: Apgar score < 7 at 5 minutes, pH < 7.10, neonatal intensive care unit admission, hypothermia therapy, peripartum death, and cephalic or upper‐limb trauma. The association between potential risk factors and perinatal morbidity was assessed using logistic regression, accounting for a center effect. Imputation was performed for missing data.

**Results**: A total of 11,938 instrumental deliveries were included. The primary outcome was observed in 16.9% of deliveries (table 1). Increased perinatal morbidity was associated with sequential instrument use (aOR = 1.50, 95% CI = 1.26–1.76), occiput posterior position (a OR = 1.30, 95% CI = 1.07–1.58), and delivery between 8 PM and 8 AM (aOR = 1.16, 95% CI = 1.05–1.29) (table 2). There was no significant difference in neonatal morbidity related to the type of instrument used (vacuum or forceps) or associated obstetric pathology (macrosomia, preeclampsia, IUGR).

**Conclusion**: Sequential use of instruments, occiput posterior fetal position, and deliveries occurring during night shifts are risk factors of neonatal morbidity in case of instrumental vaginal births among nulliparous women. However, neither the type of instrument nor associated obstetric pathology influence neonatal morbidity.

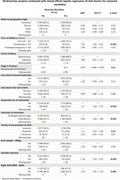

## 400 | Maternal and Neonatal Outcomes Following Maternal Cardiac Arrest

Allison Kurzeja^1^; Lindsay Yeh^1^; Anna Buford^2^; Yevgenia Y. Fomina^2^; Jessica E. Pruszynski^2^; C. Edward Wells^1^

*^1^University of Texas Southwestern Medical Center, Dallas, TX; ^2^University of Texas Southwestern, Dallas, TX*

4:00 PM ‐ 6:00 PM

**Objective**: Maternal cardiac arrest is an uncommon but catastrophic event. We sought to define the maternal and neonatal outcomes associated with maternal code at our public tertiary care center.

**Study Design**: This is a single‐institution retrospective cohort study of cases of maternal cardiac arrest from 2010 through 2024 at a large county hospital. Demographics and data regarding maternal code events, delivery, and neonatal outcomes were obtained from the medical record. All cases of maternal codes in the setting of pregnancy or within six months postpartum were included. A descriptive statistical analysis was completed.

**Results**: Thirty‐eight maternal codes were identified during the study timeframe, with 169,848 deliveries, this is consistent with 2 per 10,000 births. Of the 38 cases, 6 occurred at a gestational age of less than 20 weeks. Thirty‐five codes occurred in the hospital with 25 (71%) resulting in return of spontaneous circulation (ROSC). Of the 3 cases of cardiac arrest outside of the hospital, only one patient survived. Overall, 23 individuals (61%) survived until discharge with an average length of stay of 18.2 days. For survivors, ROSC was achieved within 5 minutes in 74%. Eleven of the 38 individuals had a live fetus at 24 weeks or greater at the time of code. A resuscitative hysterotomy occurred in 9 out of 11 (82%) with a median time from code to delivery of 4 minutes. All of the neonates who underwent resuscitative hysterotomy survived until discharge. NICU admission occurred in the majority (78%), with 44% requiring mechanical ventilation and 33% complicated by hypoxic‐ischemic encephalopathy. An additional 13 patients experienced a code in the immediate peripartum period with a median time from delivery to maternal code of 22 minutes.

**Conclusion**: Despite the infrequent and high acuity nature of maternal codes, achievement of ROSC and survival remains high, especially in the event of in‐hospital cardiac arrest. Rapid delivery of the neonate, within four minutes of maternal code, was associated with a high likelihood of neonatal survival in the setting of viability.

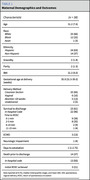

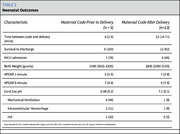

## 401 | Increasing Antimicrobial Resistance Among Post‐Cesarean Surgical Site Infections at a Large Public Hospital from 2009‐2019

Allison Kurzeja^1^; Isabelle Mulder^1^; Amanda C. Zofkie^1^; Dena Taherzadeh^2^; Darlene Madriz^2^; April R. Gorman^1^; Scott W. Roberts^1^; Emily H. Adhikari^1^

*^1^University of Texas Southwestern Medical Center, Dallas, TX; ^2^Parkland Health, Dallas, TX*

4:00 PM ‐ 6:00 PM

**Objective**: To evaluate trends of bacterial composition and antimicrobial resistance among post cesarean surgical site infections (SSIs) in a cohort exposed to contemporary antibiotic prophylaxis.

**Study Design**: This is a single‐institution retrospective cohort study of post‐cesarean SSIs for whom culture data was available between 2009‐2019 at a large county hospital. Medical records were reviewed to examine microbes isolated, and antimicrobial susceptibility data over time and by wound class. Wounds were clinically classified as superficial, deep, or organ space SSIs according to National Healthcare Safety Network guidelines. Logistic regression was completed to look at trends over time for antimicrobial resistance and bacterial composition.

**Results**: Of 41,338 cesarean sections, 484 cultured wounds were identified. 104 individuals (21.5%) had chorioamnionitis preceding delivery. SSI incidence remained below 2% consistently; however, positive cultures increased during the study (p = 0.005). For all organisms isolated, resistance to any antibiotic increased at a rate of 0.65 per year (p = 0.13 for trend). Escherichia coli (E.coli) was the most prevalent microbe identified in 67(13.8%), including 55(11.4%) superficial and 12(2.5%) deep or organ space infections. Significant increases in both the frequency of E. coli isolates among superficial SSIs (0.75 per year, p = 0.005) and antibiotic resistance of E.coli (0.49 per year, p = 0.014) were seen. Of E. coli isolates with susceptibility testing, 9/23 (37.5%) were resistant to ampicillin, 25/58 (49.1%) to ampicillin‐sulbactam, 13/63 (23%) to gentamicin, and 23/66 (34.8%) to Bactrim.

**Conclusion**: While post‐operative cesarean SSI rates have remained relatively constant at a single center over the past decade, antibiotic resistance is increasing, particularly among E. coli isolates. The impact of increasing antimicrobial resistance on maternal and neonatal morbidity and infection‐associated healthcare costs warrants further study, and safety data for newer antimicrobial agents in pregnancy and lactation are urgently needed.

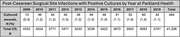

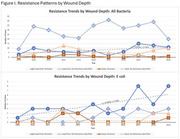

## 402 | Polyhydramnios is Associated with Worse Perinatal Outcomes Among Non‐Anomalous Births Complicated by Diabetes

Alyssa R. Hersh; Bharti Garg; Wendy Tian; Kristin C. Prewitt; Amy M. Valent; Aaron B. Caughey

*Oregon Health & Science University, Portland, OR*

4:00 PM ‐ 6:00 PM

**Objective**: Pre‐existing diabetes mellitus (DM) is associated with adverse perinatal outcomes. Polyhydramnios is more common among pregnant persons with DM, particularly those with suboptimal glycemic control. Our study examined perinatal outcomes in patients with DM in pregnancy with co‐occurring polyhydramnios.

**Study Design**: This was a retrospective cohort study of births in California complicated by pre‐existing DM between 2008‐2020. We included singleton, non‐anomalous births between 32 and 42 weeks of gestation. We assessed numerous maternal and neonatal outcomes among births complicated by polyhydramnios compared to those without polyhydramnios. We performed bivariate and multivariable logistic regression analyses adjusting for significant confounders with a p‐value of 0.05.

**Results**: There were 56,106 people with DM included in this study, of which 1,767 (3.1%) had polyhydramnios. Polyhydramnios was associated with worse outcomes for both pregnant persons and their neonates. There were strong associations observed between polyhydramnios and cesarean delivery despite stratification by history of cesarean delivery, and there were also strong associations with concurrent macrosomia (46.3% vs 19.4%, p< 0.001; aOR 3.41, 95% CI 3.07‐3.78) and infant death (0.7% vs 0.2%, p< 0.001; aOR 2.48, 95% CI 1.28‐4.81). Additionally, hypertensive disorders, severe maternal morbidity, lower APGAR at 5 minutes, neonatal ICU admission, and respiratory distress syndrome were more common among people with DM and polyhydramnios.

**Conclusion**: Polyhydramnios was associated with significantly worse outcomes among pregnant people with pre‐pregnancy DM. These results highlight the importance of strategies to mitigate the risk of development of polyhydramnios and optimize glycemic control in patients with DM to improve perinatal outcomes.

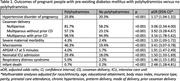

## 403 | Perinatal Outcomes Pre‐ and Post‐Arrive Trial Publication Among Low‐Risk Term Nulliparous Births in California

Alyssa R. Hersh; Bharti Garg; Megha Arora; Aaron B. Caughey

*Oregon Health & Science University, Portland, OR*

4:00 PM ‐ 6:00 PM

**Objective**: Following publication of the ARRIVE trial in 2018, there were notable changes to pregnancy management of low‐risk nulliparous patients at term, particularly in rates of induction of labor. Therefore, we sought to assess the differences in management and outcomes pre‐ and post‐ARRIVE trial publication.

**Study Design**: This was a retrospective cohort study of singleton, non‐anomalous, nulliparas between 39‐42 weeks of gestation in California. To assess only low‐risk pregnant persons, we excluded births complicated by chronic hypertension, diabetes, breech presentation, advanced maternal age, oligohydramnios, polyhydramnios, and placental conditions requiring cesarean delivery. We assessed births in the two years before and after publication of the ARRIVE trial, 2016‐2017 and 2019‐2020, respectively. We assessed overall outcomes among the entire cohort, then stratified by week of gestation. Chi squared and multivariable logistic regression were used for statistical analyses.

**Results**: There were 151,779 births in 2016‐2017 and 126,829 births in 2019‐2020 that met our inclusion criteria. Compared with pre‐ARRIVE trial publication, induction of labor increased (31.8% vs. 39.1%, p< 0.001) and the rate of cesarean delivery decreased (22.7% vs. 21.3%, p< 0.001). There was a lower rate of adverse neonatal outcomes post‐ARRIVE trial publication, including NICU admission (7.4% vs. 6.4%, p< 0.001), respiratory distress syndrome (0.49% vs. 0.44%, p = 0.041), and stillbirth (0.09% vs. 0.06%, p = 0.002). Outcomes remained similar upon stratification by week of gestation.

**Conclusion**: In this study, we found that maternal and neonatal outcomes were improved on a state level after publication of the ARRIVE trial in 2019‐2020 when compared to 2016‐2017. As management of low‐risk nulliparas at term continues to evolve, it is critical to continue to assess outcomes on a population level.

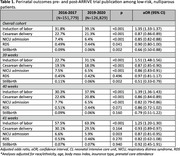

## 404 | Circadian Variations of Glucose Challenge Test Results In Pregnancy: Examining Non‐Linear Trends

Amanda Makol^1^; Luke Keating^2^; R. Jonathan Robitsek^1^; Tristen Tang^1^; Bailey Lien^1^; Lena Woo^1^; Ivan Ngai^1^

*^1^Flushing Hospital Medical Center, Queens, NY; ^2^Flushing Hsopital Medical Center, Queens, NY*

4:00 PM ‐ 6:00 PM

**Objective**: Research has documented circadian variations in glucose challenge test (GCT) results by comparing morning to afternoon collection. Variations by collection time have implications for the performance of potentially unnecessary glucose tolerance tests (GTTs). However, more detailed examination of the complex non‐linear trends in GCT results across collection times has yet to be conducted.  Therefore, we aim to examine non‐linear relations between collection time and GCT values using polynomial regression.

**Study Design**: This is a retrospective cohort study of all pregnancies 2017‐2023 for which GCT was collected after 24 weeks in a community hospital in New York. Patients with pregestational diabetes mellitus (DM), or early diagnosis of DM in the 1st or 2nd trimester were excluded. To examine non‐linear trends in GCT values, we used polynomial regression with a stepwise approach to adding polynomial terms to the initial linear model, assessing model fit with each inclusion. 

**Results**: Our sample included 5341 pregnancies for which GCT was collected. The sample was highly racially/ethnically diverse (Asian: 23.62%, Hispanic/Latino: 66.39%, Other: 10.99%). In the final model, we found significant linear (B = .47, *p* = .034), quadratic (B = ‐.18, *p*< .001), and cubic terms (B = ‐.02, *p*< .001), indicating a non‐linear relationship of GCT values with collection time. Evaluation of inflection points showed that GCT values reached a local maximum at approximately 1:30pm. 

**Conclusion**: GCT values vary with diurnal rhythms, such that observed values appear to increase throughout the AM, peak in the early afternoon, and then trend downwards. These findings have clinical implications, as GCTs collected in the afternoon may lead to more false positives and potentially unnecessary further testing by GTT.  Overall, these data suggest that GDM screening protocols that include the GCT should be refined to account for the effect of time of day on test performance.

## 405 | Adherence to Antiseizure Medications in Pregnancy

Amelie Pham^1^; Andrew D. Wiese^1^; Andrew J. Spieker^1^; Chad You^2^; Ashley A. Leech^1^; Margaret A. Adgent^1^; Carlos G. Grijalva^1^; Sarah S. Osmundson^1^

*^1^Vanderbilt University Medical Center, Nashville, TN; ^2^Vanderbilt University Medical Center, Nasvhille, TN*

4:00 PM ‐ 6:00 PM

**Objective**: To evaluate antiseizure medication (ASM) adherence in pregnancy.

**Study Design**: We constructed a retrospective cohort of births (2007‐2019) among patients ≥ 20 weeks’ gestation with continuous Tennessee Medicaid enrollment 90 days before conception through delivery, using linked healthcare claims, prescription fills, hospital discharge, and birth certificate data. We included births to patients aged 15‐44 with evidence of ASM use before and during pregnancy, defined by ≥ 2 prescription fills in the 90 days before conception and ≥ 1 prescription fill between conception and delivery, for a single ASM (monotherapy for newer (levetiracetam, lamotrigine, oxcarbazepine, zonisamide) or older (carbamazepine, phenytoin, and valproate) ASM). We excluded births where patients switched to an alternative ASM or used > 1 ASM (polytherapy). We categorized timing of ASM discontinuation and calculated adherence as the percentage of days covered by filled ASM prescriptions from conception through delivery, accounting for hospitalizations. We evaluated ASM use and adherence by medication, class, and associated epilepsy diagnosis.

**Results**: Among 2,135 eligible births with ASM monotherapy, 30% had an epilepsy diagnosis and 41% had no diagnosis traditionally associated with ASMs (Table 1). Overall median adherence was low (23%, IQR 12‐67%) with 58% discontinuing ASMs before the third trimester (Table 2). Adherence did not significantly differ by ASM class, but was higher among levetiracetam and phenytoin users and significantly higher among births with an epilepsy diagnosis compared to those without (Table 2). Most common ASMs used, by class, were lamotrigine and carbamazepine in all users compared to levetiracetam and phenytoin in those with epilepsy.

**Conclusion**: The American Academy of Neurology recommends continued use of most ASMs in pregnancy when clinically indicated. In our cohort of births with regular ASM use preceding pregnancy, ASM adherence in pregnancy was poor. These findings emphasize the need for improved patient counseling on ASM use in pregnancy.  

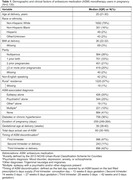

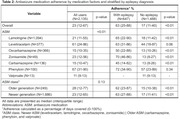

## 407 | is Timing of Onset of Early Fetal Growth Restriction Associated with Worse Outcomes?

Anam Farooqui^1^; Emily K. Guernsey^2^; Vanya Manthena^2^; Kelly Nelson Kelly^2^; Andrew Rausch^2^; Ryan E. Longman^2^; Ashish Premkumar^2^

*^1^University of Chicago, Chicago, IL; ^2^Pritzker School of Medicine, University of Chicago, Chicago, IL*

4:00 PM ‐ 6:00 PM

**Objective**: Fetal growth restriction (FGR) is associated with significant neonatal morbidity and mortality, which can especially be impacted by timing of onset. Limited data are available to assess whether timing of diagnosis among the high‐risk cohort of early‐onset FGR (i.e., diagnosis at < 32 weeks) is associated with adverse outcomes.

**Study Design**: We conducted a single‐site retrospective study of singleton gestations with early‐onset FGR, diagnosed between 2020‐2023. The exposure was gestational age (GA) at time of diagnosis, dichotomized into 20‐27 weeks (group 1) and 28‐31 weeks (group 2). The primary outcome was a composite of adverse neonatal outcomes (i.e., neonatal intensive care unit admission, respiratory distress syndrome, need for intubation, sepsis, necrotizing enterocolitis, intraventricular hemorrhage, periventricular leukomalacia, retinopathy of prematurity, death). Secondary outcomes were GA at delivery, route of delivery, diagnosis of small‐for‐gestational age, neonatal length of stay, and all components of the primary outcome. Bi‐ and multivariate analyses were performed.

**Results**: 94 participants were eligible; 52% (n = 49) were in group 1 and 48% (n = 45) were in group 2. Individuals with earlier onset of FGR were more likely to have public payor insurance, be nulliparous and have hypertensive disorders (Table 1). The frequency of the composite outcome was not significantly different between the two groups (45 v. 29%, RR 1.55, 95% CI 0.89‐2.70), even after controlling for receipt of antenatal steroids (aRR 1.29, 95% CI 0.82‐2.04). Furthermore, there were no differences in any of the secondary outcomes (Table 2).

**Conclusion**: GA at diagnosis of early‐onset FGR is not associated with worse maternal or perinatal outcomes, though the directionality of the findings warrants further investigation with a larger sample size.

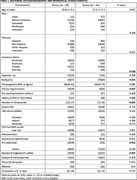

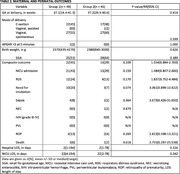

## 408 | Impact of Health‐Related Social Needs on Postpartum Anxiety in an Underserved Urban Community

Andrea Rizkallah^1^; Ashlyn K. Lafferty^2^; Robert B. Martin^2^; Elaine L. Duryea^2^; Kristie R. Wilburn‐Wren^2^; Donald D. McIntire^2^; Catherine Y. Spong^2^; David B. Nelson^2^

*^1^UT Southwestern, Dallas, TX; ^2^University of Texas Southwestern Medical Center, Dallas, TX*

4:00 PM ‐ 6:00 PM

**Objective**: The aim of this study was to determine the association of health‐related social needs (HRSNs) with postpartum anxiety for individuals living in underserved communities. We hypothesized that individuals with greater social needs would have increased rates of anxiety after birth.

**Study Design**: This was a prospective observational study of patients delivered from 1 Oct 2020 through 31 Dec 2022 with subsequent 1‐year follow‐up postpartum. Participants living in underserved areas identified through a community‐health needs assessment were enrolled in a dedicated, multidisciplinary postpartum care program. HRSNs were assessed upon enrollment using a standardized assessment modeled from the HealthyPeople2030 framework, and medical services were provided for 12 months. Patients were screened for anxiety using Generalized Anxiety Disorder‐7 (GAD‐7), at 1, 3, 6, and 9 months with positive scores ≥ 10 prompting mental health referral. Cochran‐Armitage trend test was used to examine the association between number of HRSN and GAD‐7 and the number of visits completed in relation to the type of HRSN.

**Results**: Of 1647 enrolled, 64.5% (N = 1063) reported at least one HRSN with 28% (N = 468), 16% (N = 257), and 21% (N = 338) of individuals reporting one, two, or more than two HRSN, respectively. The most prevalent HRSNs were poor WiFi (23.6%), access to childcare (21%), and missed electric or gas bill (15.3%). There was a significant association between increasing number of reported HRSN and screening positive for anxiety using GAD‐7 instrument (P < 0.001, Figure). Individually, a reported need for transportation to medical appointments (N = 158) and access to childcare (N = 364) were significantly associated with fewer number of follow‐up visits (both P < 0.01).

**Conclusion**: Among patients living in an underserved urban community, increasing number of HRSN was significantly associated with higher GAD scores, and specific HRSNs were associated with lower rates of postpartum follow‐up. Addressing HRSN provides the opportunity to impact maternal wellbeing and postpartum follow‐up.

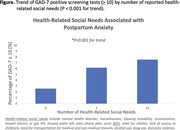

## 409 | Development of Common Data Elements for Maternal Health Research: Results from a Delphi Consensus Process

Elizabeth Stierman^1^; Carrie Wolfson^2^; Sarah Clifford^1^; Meighan Mary^1^; Amanda Burgess^1^; Khyzer Aziz^3^; Sean O'Reilly^1^; Jessica L. Gleason^4^; Diane Gumina^5^; Paul Nagy^3^; Andreea A. Creanga^1^

*^1^Johns Hopkins University, Baltimore, MD; ^2^Johns Hopkins Bloomberg School of Public Health, Baltimore, MD; ^3^JHU, Baltimore, MD; ^4^Epidemiology Branch, NICHD‐NIH, Bethesda, MD; ^5^NIH, Bethesda, MD*

4:00 PM ‐ 6:00 PM

**Objective**: Common data elements (CDEs, i.e., standardized questions paired with allowable responses) are critical for providing consistency in data collection and promoting research collaborations. This study aims to determine a *minimum reporting set of CDEs* by NICHD grantees conducting maternal health (MH) research.

**Study Design**: NICHD convened a 64‐expert panel including representatives from large research networks, ACOG, SMFM, CDC, HRSA, and NIH. Experts formed 2 working groups supporting development of biomedical and psychosocial CDEs. Two modified Delphi processes, each with 2 voting rounds, were undertaken. In Delphi 1 (03/24‐05/24; 84% response rate), experts voted on MH constructs for standardized data collection categorized as: Tier 1 (all MH research), Tier 2 (thematic MH research areas), or neither. Constructs for which >80% of experts affirmed the importance of standardization were retained for categorization to Tier 1 or 2 based on majority vote. The Tier 1 list was restricted to constructs with >75% of experts identifying the construct as Tier 1, forming the *minimum set of constructs* for all MH research. For measuring these constructs, in Delphi 2 (07/24‐08/24; 70% response rate), experts used 5‐point Likert scales to rate the feasibility and validity of specific CDEs identified via landscape analysis and literature searches.

**Results**: Experts voted on 267 biomedical and 194 psychosocial constructs; 202 biomedical (146 Tier 1; 56 Tier 2) and 144 psychosocial (90 Tier 1; 54 Tier 2) constructs were retained. Restriction to constructs with >75% Tier 1 votes yielded 35 biomedical and 22 psychosocial constructs in the *minimum set of constructs* for all MH researchers (Table). A total of 89 biomedical and 115 psychosocial CDEs were rated by experts during Delphi 2. Of these, depending on available data source (EHR, survey, other), the *minimum reporting set* includes 50‐60 CDEs/CDE bundles.

**Conclusion**: Reporting of CDEs across MH research is expected to improve data quality, study consistency, data interoperability, and our understanding of how to improve MH.

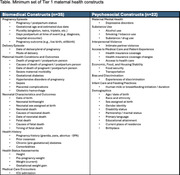

## 410 | Does Fetal Growth Restriction Actually Resolve? Maternal and Neonatal Risks with Resolved Fetal Growth Restriction

Angela Nakahara^1^; Olivia Feltner^1^; William Fesmire^2^; Victoria Schlesinger^1^; Grace Anne Holladay^1^; Anna Belk^3^; Patricia Goedecke^1^; Jim Wan^2^; Kerri Brackney^1^

*^1^University of Tennessee Health Science Center, Memphis, TN; ^2^University of Tennessee–Department of Preventative Medicine, Memphis, TN; ^3^Regional One Health, Memphis, TN*

4:00 PM ‐ 6:00 PM

**Objective**: Little guidance exists on the management of FGR that resolves, and potential neonatal outcomes including birth weight and NICU admission are unknown. We compared the likelihood of identifying small for gestational age (SGA) infants in fetuses with resolved FGR (based on estimated fetal weight (EFW) and abdominal circumference (AC) >10^th^ percentile) compared to those without history of FGR.

**Study Design**: A retrospective case control study of fetal growth ultrasounds (US) performed at our institution from 01/2019‐04/2024 was conducted. US evaluating biometric parameters for singleton pregnancies originally meeting criteria for FGR based on EFW or AC < 10^th^ percentile, with resolution of both EFW and AC parameters, were included. Controls were matched based on year of birth, gestational age (GA) and maternal age at delivery. Primary outcome was prevalence of SGA neonate at birth; secondary outcomes were need for NICU admission, GA at birth and mode of delivery. We applied a mixed effects logistic regression model comparing cases and controls.

**Results**: A total of 140 infants were evaluated. Cases with resolved FGR had a 2.26 RR (95% CI 1.44‐3.24) for SGA. When controlling for hypertensive disorders of pregnancy, the RR was 2.31 (95% CI 1.46‐3.32). The mean GA of delivery was 37 weeks for both groups (p >0.9) and NICU admission rates were similar (24% vs 24%, p >0.9). Cesarean section (CS) was more likely in pregnancies with resolved FGR (37% vs 26%, p = 0.039) and more likely to be performed for non reassuring fetal heart tones (NRFHT) than those without history of FGR (10% vs 2.9%, p = 0.025).

**Conclusion**: Neonates with history of FGR are more likely to be SGA compared to those with normal growth profile in antenatal period and are more likely to need CS for NRFHT.

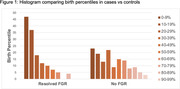

## 411 | Time is Money: Comparing Costs in 12 Vs 24 Hours of Postpartum Magnesium

Angela Nakahara^1^; Bailey Huggins^1^; Claire Sorenson^1^; Elise Sickle^1^; Victoria Schlesinger^1^; Zach Berry^1^; Patricia Goedecke^1^; Jim Wan^2^; Paul J. Wendel^1^; Kerri Brackney^1^

*^1^University of Tennessee Health Science Center, Memphis, TN; ^2^University of Tennessee–Department of Preventative Medicine, Memphis, TN*

4:00 PM ‐ 6:00 PM

**Objective**: This study evaluates the implementation of a protocol for shortened magnesium sulfate (MAG) duration in postpartum seizure prophylaxis for preeclampsia (preE). We compared MAG administration of 12 vs 24 hours, assessing cost‐effectiveness, patient safety, and postpartum length of stay (LOS), utilizing clinical parameters to identify suitable candidates.

**Study Design**: This interrupted time series and retrospective cohort study evaluated all patients with preE who received MAG and delivered at a tertiary academic center from 04/2022‐04/2024. Patients meeting clinical criteria were considered for shortened 12 (G1) vs traditional 24 hour (G2) MAG administration. Primary outcome was difference in mean total hospital cost (MTHC) after delivery pre vs post protocol; secondary outcomes were number of patients with G1 course, rates of postpartum preE readmissions (PPRA), severe maternal morbidity (SMM) and LOS between groups. Wilcoxon rank sum test was applied to compare variables between G1 vs G2, with alpha = 0.05 as statistically significant.

**Results**: A total of 656 patients were evaluated; 53% (n = 349) were post protocol, of which 23% (n = 80) met criteria for G1. There were no statistically significant differences in gestational age at delivery, delivery for preE, or mode of delivery between pre vs post protocol groups. The MTHC was not significantly different after protocol initiation ($9158 vs $9514, p = 0.4) however LOS was shorter post protocol (2.72 vs 3.02 days, p = 0.004). Post protocol, G1 had less MTHC ($6840 vs $9850, p = 0.001) and shorter LOS (2.23 vs 2.87 days, p < 0.001). PPRA rates were not significantly different pre protocol (10 vs 6.9%) with RR 0.7 (95% CI 0.4‐1.1). Overall SMM was decreased post protocol (7.2% vs 14%, p = 0.004) with G1 having lower SMM vs G2 (0% vs 9.3%, p = 0.005).

**Conclusion**: Shortened MAG course is a cost effective consideration with shortened LOS and decreased PPRA, but likely requires a larger study group to detect overall differences in MTHC. SMM may be decreased with shortened MAG however, direct causation cannot be established.

## 412 | Previable Premature Rupture of the Membranes and Neonatal Mortality/Morbidity in a National Retrospective Cohort

Anja Gojic^1^; Yanchen Wang^1^; Eleanor Pullenayegum^2^; Amit Mukerji^1^; Swati Agrawal^1^; Eugene Ng^3^; Miroslav Stavel^4^; Yun‐Yi Cui^1^; Sarah D. McDonald^1^

*^1^McMaster University, Hamilton, ON; ^2^Hospital for Sick Children and University of Toronto, Toronto, ON; ^3^University of Toronto, Toronto, ON; ^4^Royal Columbian Hospital, New Westminster, BC*

4:00 PM ‐ 6:00 PM

**Objective**: Counselling after previable preterm premature rupture of the membranes (PPROM) is challenging due to older previable PPROM cutoffs and small studies. Our objective was to assess neonatal mortality/morbidity after previable PPROM < 22 weeks versus no PPROM to assist in counselling at viability and decision making.

**Study Design**: We conducted a retrospective cohort study of liveborn singletons born < 32 wk’ gestation from 2018‐2022, who received active resuscitation (including delivery room deaths), or did not need it, and were admitted to a neonatal intensive care unit within the Canadian Neonatal/Preterm Birth Network database. Among previable PPROM infants, gestational age at birth and latency period were determined. Our primary outcome was a composite of neonatal mortality or severe morbidity consisting of any one or more of severe respiratory distress syndrome, severe intraventricular hemorrhage, severe necrotizing enterocolitis, severe retinopathy of prematurity, moderate/severe bronchopulmonary dysplasia, cystic periventricular leukomalacia or sepsis. Outcomes were compared between infants with and without previable PPROM < 22 wk, using generalized estimating equations to account for hospital effect and adjusting for confounders and covariates.

**Results**: As onset of previable PPROM (n = 225) increased from ≤16 to 21 wk’ gestation, median gestational age at birth decreased from 27.5 wk (IQR 24.5, 29.0) to 25.0 (IQR 23.0, 28.0) and latency period decreased from 86.5 days (IQR 63.5, 98.0) to 30.0 (IQR 15.0, 49.0, Table 1). Infants with previable PPROM had increased odds of neonatal mortality/severe morbidity (90%), compared to those without (66%, n = 7846, adj OR 1.71, 95% CI 1.14 to 2.56, Table 2). There was a trend toward more sepsis with previable PPROM at 21 wk than earlier.

**Conclusion**: In a large, contemporary cohort of previable PPROM (< 22 wk) reaching viability, an inverse relationship was seen between timing of rupture and gestational age at birth, decreasing from a median of 27.5 to 25 wk. Almost all previable PPROM infants experienced mortality/morbidity, almost double the odds without PPROM.

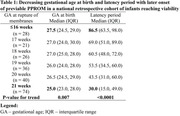

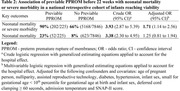

## 413 | Induction Compared with Expectant Management at Term for Individuals with a Prior Cesarean

Ann M. Bruno; On behalf of the Eunice Kennedy Shriver National Institute of Child Health and Human Development Maternal‐Fetal Medicine Units Network

*University of Utah Health, Salt Lake City, UT*

4:00 PM ‐ 6:00 PM

**Objective**: It is common to await spontaneous labor in individuals undertaking a trial of labor after cesarean (TOLAC) over concern of uterine rupture and its associated harms. We evaluated whether this concern is warranted.

**Study Design**: Secondary analysis of a multicenter cohort of patients delivering on randomly selected days at 17 U.S. hospitals (2019‐20). Data were manually abstracted from the medical record by trained staff. Term individuals with a prior cesarean delivery (CD) were included while those with a contraindication to TOLAC, planned repeat CD, multifetal gestation, stillbirth, or fetal anomaly were excluded. The primary outcome was vaginal birth after cesarean (VBAC). Secondary outcomes included uterine rupture, maternal morbidity, neonatal morbidity, and NICU admission. Multivariable modeling estimated the association between induction compared with expectant management and outcomes by gestational week. Adjusted Desirability Of Outcome Ranking (DOOR) analyses calculated the probability that induction leads to more desirable dyadic (paired maternal‐neonatal) outcomes. The ordinal DOOR outcome ranged from VBAC without uterine rupture or morbidity (most desirable) to CD with uterine rupture and maternal and neonatal morbidity (least desirable).

**Results**: Of the 935 individuals included, mean maternal age was 31 years (± 5), median body mass index 31.8 kg/m^2^ (interquartile range 27.7‐36.7), and 89% had one prior CD. In adjusted multivariable models, there was no association between induction and odds of VBAC in week 37, 38 or 40; in week 39, induction was associated with increased odds of VBAC (Table). Induction and expectant management had similar odds of maternal morbidity at all weeks, while induction was associated with neonatal morbidity and NICU admission only at week 37. In DOOR analyses, induction in week 39 resulted in a higher probability of a desirable outcome for the dyad (adjusted DOOR probability 55%, 95% CI 51‐59%; Figure).

**Conclusion**: For those undertaking TOLAC, induction compared with expectant management in gestational week 39 optimized outcomes for the maternal‐neonatal dyad.

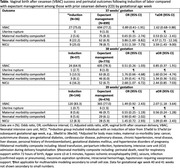

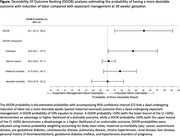

## 414 | Maternal and Obstetric Factors Associated with Successful Cerclage Treatment: A Prediction Model

Annabelle L. van Gils^1^; Jasper T. Lamens^1^; Flip W. W. van der made^2^; Sanne J. Gordijn^3^; Brenda J. Hermsen^4^; Brenda M. M. Kazemier^1^; Martijn A. Oudijk^5^; Eva Pajkrt^1^

*^1^Amsterdam UMC, Amsterdam, Noord‐Holland; ^2^St. Franciscus Hospital, Rotterdam, Zuid‐Holland; ^3^University Medical Center Groningen, Groningen, Groningen; ^4^Onze Lieve Vrouwe Gasthuis, Amsterdam, Noord‐Holland; ^5^Amsterdam UMC, location University of Amsterdam, Amsterdam, Noord‐Holland*

4:00 PM ‐ 6:00 PM

**Objective**: Cervical cerclage treatment effectively reduces the risk of recurrent preterm birth (PTB) in specific subgroups. This study aimed to identify maternal and obstetric factors influencing success of cerclage treatment (i.e. no PTB < 32 weeks) to develop a prediction model.

**Study Design**: This multicenter prospective cohort study analyzed data of the PC‐study (NTR4415) and associated observational cohort. We included patients with prior sPTB < 34 weeks and a singleton pregnancy who received a history‐based (HB) or an ultrasound‐indicated (UI) cerclage. Primary outcome was PTB < 32 weeks. Maternal and obstetric factors were included in multivariate logistic regression (MLR) and a risk model was composed for the HB‐ and UI group. Subsequently, outcomes were stratified by risk scores (high, medium and low).

**Results**: In total, 222 patients received cerclage treatment. PTB < 32 weeks occurred in 43/222 cases (19.4%), of which 13/85 in the HB group (15.3%) and 30/137 (21.9%) in the UI group. In the total group, MLR showed that higher maternal age (p = 0.04), non‐Caucasian ethnicity (p = 0.02) and smoking (p = 0.045) were associated with decreased cerclage success (i.e. higher PTB < 32 weeks). In the HB group, event rates were considered too low for proper MLR. In the UI group, higher maternal age (p = 0.008), smoking (p = 0.005), lower gestational age at placement (p< 0.001) and lower CL (p = 0.004) were associated with decreased cerclage success. Individual risk scores in this group were calculated using the composed risk model. Scores < ‐1.7 were considered low‐risk, ‐1.7 to 0 medium‐risk and > 0 high risk. For high‐risk patients, the UI‐model had a positive predictive value (PV) of 81.3% for occurrence of PTB < 32 weeks. Low‐risk patients had a PV of 91.2% for no PTB < 32 weeks. The AUC of the ROC‐curve was 0.81 (95% CI 0.72–0.90).

**Conclusion**: Several maternal and obstetric risk factors significantly influence the success rate of cerclage treatment. The developed risk model effectively stratifies patients with an UI cerclage into high, medium, and low risk categories and can be a valuable tool in counseling patients.

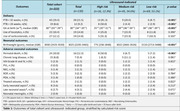

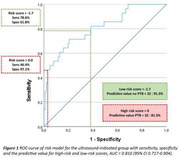

## 415 | Defining Specific Bile Acid Profiles in Intrahepatic Cholestasis of Pregnancy

Anne M. Ambia^1^; Jeffrey McDonald^2^; Yevgenia Y. Fomina^1^; Andrea Rizkallah^2^; C. Edward Wells^3^; Catherine Y. Spong^3^; Donald D. McIntire^3^; David B. Nelson^3^

*^1^University of Texas Southwestern, Dallas, TX; ^2^UT Southwestern, Dallas, TX; ^3^University of Texas Southwestern Medical Center, Dallas, TX*

4:00 PM ‐ 6:00 PM

**Objective**: Although intrahepatic cholestasis of pregnancy (ICP) is often diagnosed using total serum bile acid (BA) measurements, there is a paucity of information on the individual bile acid profiles in this enigmatic condition.  Thus, our aim was to define the specific BA profiles among a cohort of pregnant individuals with ICP.

**Study Design**: This was a prospective, observational cohort study of pregnant individuals evaluated for ICP from February 2022 to July 2023 a single academic center. Patients were characterized into pruritis of pregnancy (total BA < 10 mmol/L, referent), ICP (BA 10‐39 mmol/L), and severe ICP (BA >40 mmol/L) using a quantitative enzymatic assay.  At the time of initial evaluation for ICP, an additional blood sample was collected to evaluate for a selection of primary (cholic, chenodeoxycholic), secondary (deoxycholic), glycol‐conjugated (glycocholic, glycodeoxycholic, glycolithocholic), and tauro‐conjugated (taurocholic, taurochenodeoxycholic, taurodeoxycholic, taurolithocholic) forms of BA. These measurements were conducted using liquid chromatography and mass spectrometry methods.

**Results**: There were 39 patients evaluated: 5 with pruritis, 24 with ICP, and 10 with severe ICP.  Levels of glycocholic, taurocholic, and taurochenodeoxycholic acids were significantly increased in those with ICP. When comparing patients with ICP to severe ICP, there was an increase in both glycol and tauro‐conjugated BA (Figure).

**Conclusion**: Beyond total BA, patients with ICP have significantly elevated levels of specific BA profiles that offer an important opportunity to better understand the pathophysiology and treatment targets for this enigmatic condition.

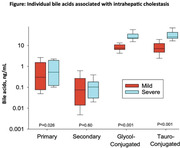

## 416 | Intrapartum Glycemic Control with IV Insulin Infusion in Pregestational and Gestational Diabetes

Annkay Alexander^1^; Yuanfan Ye^2^; Victoria C. Jauk^1^; Frances Burgan^1^; Carolyn Webster^1^; Ashley N. Battarbee^2^

*^1^University of Alabama at Birmingham, Birmingham, AL; ^2^Center for Women's Reproductive Health, University of Alabama at Birmingham, Birmingham, AL*

4:00 PM ‐ 6:00 PM

**Objective**: ACOG recommends maintaining maternal glucose < 110mg/dL in labor, often managed with IV insulin infusion. Our objective was to describe the degree of intrapartum glycemic control achieved with IV insulin infusion and identify factors associated with intrapartum glycemic control.

**Study Design**: A retrospective cohort study of pregnant persons with pregestational or gestational diabetes who received IV insulin infusion during labor at a tertiary center was conducted (2015‐2024). Antepartum fetal demise and deliveries < 22 weeks were excluded. At our center, IV insulin titration is individualized by clinicians based on hourly glucose values and response to prior adjustments. The primary outcome was percentage of intrapartum glucose values within target range of 65‐110mg/dL (TIR_Labor_). We compared TIR_L_ and other glycemic measures by diabetes type. Multivariable linear regression with backward stepwise selection was used to identify sociodemographic and clinical factors associated with TIR_L._

**Results**: Of 896 included persons, 49 (5%) had A1GDM, 305 (34%) had A2GDM, 365 (41%) had T2DM, and 177 (20%) had T1DM. Overall, 52% were non‐Hispanic Black, 32% non‐Hispanic White, 14% Hispanic; 65% had government insurance; mean BMI was 40±9 kg/m2; mean delivery GA was 35.8±3.2 weeks; and 72% had labor induction. The mean TIRL was 51±33% with differences in glycemic control by diabetes type (Table 1). A2GDM (vs T1D), labor induction, use of SMBG (vs CGM) for monitoring, greater number of intrapartum glucose measurements and later delivery year were associated with higher TIR_L_, whereas higher HbA1c and corticosteroids < 7 days before delivery were associated with lower TIR_L_ (Table 2).

**Conclusion**: Strict intrapartum glycemic control (65‐110 mg/dL) is difficult to achieve with clinician‐managed IV insulin infusion. Future interventions to improve intrapartum control including alternatives to clinician‐managed IV insulin should address potential barriers such as diabetes type, suboptimal glycemic control before admission, recent antenatal corticosteroids, and short labor duration with fewer intrapartum glucose measurements.

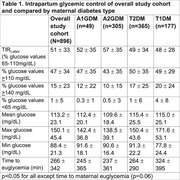

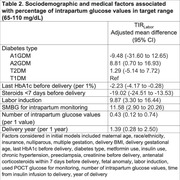

## 417 | Time in Range and Barriers to Achieving Continuous Glucose Monitoring Targets in Type 2 Diabetes

Annkay Alexander^1^; Amber Sanders^2^; Claire A. McIlwraith^1^; Brian E. Brocato^1^; Ashley N. Battarbee^3^

*^1^University of Alabama at Birmingham, Birmingham, AL; ^2^Lincoln Memorial University DeBusk College of Osteopathic Medicine, Harrogate, TN; ^3^Center for Women's Reproductive Health, University of Alabama at Birmingham, Birmingham, AL*

4:00 PM ‐ 6:00 PM

**Objective**: For pregnant persons with type 1 diabetes (T1D) using CGM, the recommended time in target range (TIR, 63‐140 mg/dL) is > 70%. However, TIR recommendations for pregnant persons with type 2 diabetes (T2D) are uncertain. Our objectives were to evaluate TIR achieved before delivery among pregnant persons with T2D and identify barriers to achieving TIR > 70%.

**Study Design**: Retrospective cohort study of gravidae with T2D who used CGM and received care at a single, tertiary center (2019‐2024). Timed CGM data was downloaded, and TIR in 30 days pre‐delivery was calculated. Factors were compared between persons with TIR > 70% vs £ 70%. Multivariable logistic regression with backward selection identified the factors independently associated with TIR > 70%. Sensitivity analyses evaluated factors associated with TIR > 70% in 2 weeks and %TIR 30 days pre‐delivery.

**Results**: Of 134 pregnant persons included, the average TIR during 30 days pre‐delivery was 59.5±19.2% with 43 (32%) achieving TIR > 70% pre‐delivery. Of 22 sociodemographic and clinical factors, only pre‐pregnancy HbA1c and baseline insulin dose differed between TIR > 70% vs £ 70% (Table 1) and were independently associated with TIR > 70% in regression models (Table 2). For every 1% increase in pre‐pregnancy HbA1c, there was 26% lower odds of TIR > 70% (aOR 0.74, 95% CI 0.60‐0.94). For every 10 unit increase in baseline insulin dose, there was 16% lower odds of TIR > 70% (aOR 0.84, 95% CI 0.74‐0.94). These findings were unchanged in sensitivity analyses evaluating TIR > 70% in 2 weeks and %TIR in 30 days pre‐delivery.

**Conclusion**: In this cohort of gravidae with T2D, the average TIR in 30 days pre‐delivery was 60% with only 1/3 achieving the T1D‐recommendation of TIR > 70%. Barriers to achieving TIR > 70% that should be addressed include improving glycemic control before pregnancy and identifying effective interventions for gravidae with high insulin requirements. The lack of association with other factors suggests that patients may be able to achieve TIR > 70% pre‐delivery despite unfavorable social determinants or later access to prenatal care and CGM.

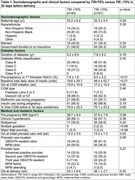

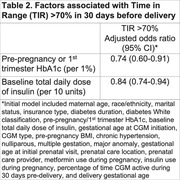

## 418 | Postpartum Gastroenterology Referrals and Follow Up in Pregnant Patients with Viral Hepatitis

Anthony Chartier^1^; Kaitlyn Stark^2^; Han‐Yang M. Chen^3^; Irene A. Stafford^3^

*^1^Department of Obstetrics, Gynecology and Reproductive Sciences, McGovern Medical School at UTHealth, Houston, TX; ^2^University of Texas Health Science Center at Houston ‐ McGovern Medical School, Houston, TX; ^3^McGovern Medical School at UTHealth Houston, Houston, TX*

4:00 PM ‐ 6:00 PM

**Objective**: The objective of this study was to determine rates of appropriate postpartum gastroenterology (GI) referral and follow up in pregnant patients with hepatitis B and C.

**Study Design**: After institutional review board approval, we performed a retrospective cohort study among 19,974 pregnant patients who delivered at a safety net hospital system between 1/2019 and 12/2022. The primary outcomes were referrals to GI and postpartum follow‐up with GI. All baseline demographics and obstetrical variables were collected, including viral hepatitis status. Differences in the baseline characteristics and outcomes were examined using chi‐square or Fisher's exact test for categorical variables. A P value of < 0.05 was considered significant. Multivariable Poisson regression models with robust error variance were used to examine the association, and an adjusted relative risk (aRR) with 95% CIs was calculated.

**Results**: Out of the 19,974 pregnant patients in this cohort, 82 (0.4%) had hepatitis B and 49 (0.2%) had hepatitis C. Hepatitis B was more prevalent in Black women, limited prenatal care, and lack of substance abuse, p = < 0.001, for each. (Table 1). Patients with hepatitis B were significantly more likely to be referred to GI than hepatitis C patients, with 62 (75.6%) of hepatitis B patients referred and 22 (44.9%) of hepatitis C patients referred (p < 0.001). Furthermore, patients with hepatitis B were significantly more likely to attend a follow‐up appointment with GI during the postpartum period. 23 (28.1%) of hepatitis B patients attended a GI appointment postpartum, compared to 5 (10.2%) of hepatitis C patients (p = 0.016). After adjustment, hepatitis B patients were 2.60 times more likely to be referred or seen by GI (CI: 1.54‐4.38), compared to hepatitis C patients.

**Conclusion**: This study demonstrates low rates of indicated GI referrals for pregnant women with viral hepatitis, despite screening recommendations to test all pregnant women to prevent mother‐to‐child transmission and to link them to care services for treatment. Further studies are needed to evaluate the reasons for these gaps.

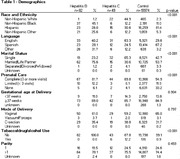

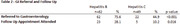

## 419 | Sociodemographic Differences in Perinatal Outcomes Following Publication of the Chap Trial

Anthony E. Melendez Torres^1^; Sarah K. Dotters‐Katz^1^; Sarahn M. Wheeler^1^; Rachel L. Wood^2^

*^1^Duke University School of Medicine, Durham, NC; ^2^Duke University, Durham, NC*

4:00 PM ‐ 6:00 PM

**Objective**: The April 2022 CHAP trial showed that tight chronic hypertension (cHTN) control decreased preterm birth (PTB) and low birthweight (BW) < 2500g. We conducted a difference in differences (DID) analysis to assess population‐level sociodemographic disparities in these outcomes before and after publication and dissemination of the CHAP trial.

**Study Design**: Population level DID analysis from February 2021 to August 2023 using US Natality data including patients with a singleton, non‐anomalous gestation, cHTN, and who initiated prenatal care at < 6 months gestation. Pre‐ and post‐CHAP periods were defined *a priori* as February 2021‐January 2022 and September 2022‐August 2023 respectively with a dissemination period from February‐August 2022. Primary outcomes were PTB< 35 weeks, PTB< 37 weeks, and BW< 2500g. Differences in outcomes were compared between Black and White race, Hispanic and non‐Hispanic ethnicity, and privately and publicly insured patients during pre‐ and post‐CHAP trial periods. The difference in differences was then calculated. Average treatment effect differences were obtained using linear regression with Stata18 SE.

**Results**: Black, Hispanic, and publicly insured patients had higher rates of PTB and low BW during the study period compared to their White, non‐Hispanic, and privately insured counterparts (Table 1). Rate differences between subgroup categories were significant during the pre‐ and post‐CHAP periods for PTB< 35 weeks, PTB< 37 weeks, and BW< 2500g, except for BW< 2500g during the pre‐CHAP period in the ethnicity subgroup. Treatment effect differences in differences were not significant for any primary outcome across subgroups (Table 1).

**Conclusion**: Sociodemographic disparities in PTB and low BW in patients with cHTN were identified across racial, ethnic, and payor status subgroups both prior to and after the CHAP trial. However, existing disparities were unchanged by dissemination of CHAP trial. Future research should assess in more granular detail if application of CHAP trial interventions can improve these disparities.

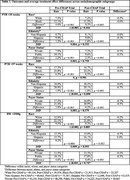

## 420 | Maternal Placental Vascular Pathology Association with Aspirin Response Among High‐Risk Singleton Pregnancies

Ariana Spiegel^1^; Joanna Chan^1^; Walter Kraft^1^; Steven McKenzie^1^; Rupsa C. Boelig^2^

*^1^Thomas Jefferson University Hospital, Philadelphia, PA; ^2^Sidney Kimmel Medical College, Thomas Jefferson University, Philadelphia, PA*

4:00 PM ‐ 6:00 PM

**Objective**: Placental vascular pathologies are noted in pregnancies complicated by preeclampsia and preterm birth.  Aspirin initiation in high‐risk pregnancies reduces the risk of these outcomes. However, it is unclear how aspirin response affects placental histopathology. Our objective is to evaluate the relationship between aspirin response and placental vascular pathology in high‐risk pregnancies taking 81mg aspirin daily.

**Study Design**: This is a secondary analysis of a prospective cohort of high‐risk singletons taking aspirin daily. Placental maternal vascular pathology was categorized according to Amsterdam classification system. The primary outcome was maternal vascular malperfusion (MVM). Aspirin response was assessed by Platelet Function Assay‐100 epinephrine closure time (PFA‐100) at baseline (before aspirin, < 16 weeks), 2‐4 weeks after aspirin initiation (follow up‐1), and at 28‐32 weeks gestation (follow up‐2). The exposure was PFA‐100 assessed as a continuous and categorical variable (<150 seconds, inadequate response) in those with and without placental MVM. The Mann‐Whitney‐U test and Chi‐squared test were used.

**Results**: Of the original cohort (n = 130), 91 participants took 81mg aspirin daily, completed requisite follow‐up, and had placental histopathology available. 38 (42%) exhibited placental MVM; baseline characteristics were similar in those with and without MVM (Table 1). There was no significant difference in aspirin response in those with and without placental MVM (Table 2), although there was a higher rate of inadequate aspirin response at 28‐32 weeks in those who developed MVM. However, this was not statistically significant (p = 0.14).

**Conclusion**: We found a high rate of placental MVM in high‐risk singletons taking aspirin daily, and a strong association between placental MVM and preterm birth. We did not find a significant association between aspirin response and placental MVM, although a larger study should be done to evaluate the relationship between aspirin response and placental vascular pathology especially when assessed in the 3rd trimester.

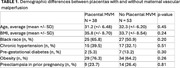

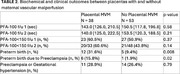

## 421 | Reducing Bias in AI Models for Fetal Heart Ultrasound: Assessing Annotation Quality

Aris T. Papageorghiou^1^; J Alison Noble^1^; Olga Patey^1^; Mostafa Sarker^1^; Netzahualcoyotl Hernandez‐Cruz^1^; Divyanshu Mishra^1^; Beverly Tsai‐Goodman^2^

*^1^University of Oxford, Oxford, England; ^2^Royal Brompton Hospital, London, England*

4:00 PM ‐ 6:00 PM

**Objective**: When building machine learning algorithms in a collaborative manner, annotating ultrasound videos of fetal heart “sweeps” is important prerequisite. These annotations are undertaken by experts following standard guidelines and criteria. To ensure these data for training machine learning algorithms are reliable, we aimed to create a system to quantify inter and intra‐annotation variation. The ultimate aim is to create quality assurance systems of video annotations to reduce annotator bias.

**Study Design**: Two experienced cardiologists (A1 and A2) each annotated 4,539 individual ultrasound frames from ten fetal heart ultrasound videos (transverse sweep). Individual frames received one of five fetal cardiac labels through manual annotation. Pairwise comparisons were made between annotations conducted two weeks apart by A1 (intra‐annotator agreement) and between cardiologists A1 and A2 (inter‐annotator agreement). Inter‐ and intra‐annotator agreements were quantified on a frame‐by‐frame basis using Intra‐Class Correlation Coefficient [ICC] and Kappa score values.

**Results**: Successful labelling of all frames were carried out. Intra‐annotator reproducibility showed correlation strong agreement (ICC = 74.9 %, Kappa score of 81%). Inter‐annotator agreement between A1 and A2 was moderate (ICC = 66.6, Kappa score of 60%).

**Conclusion**: The results show high reproducibility of annotations of frames of the fetal heart from ultrasound sweeps for a single annotator. However, when comparing annotations between different annotators, the results are less consistent, indicating only moderate agreement. These variations have implications for quality assurance and what constitutes “ground truth” when using fetal heart annotations in ultrasound videos for training machine learning algorithms.

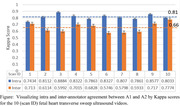

## 422 | Impact of Fetal Sex on Adverse Pregnancy and Neonatal Outcomes in Individuals with Pre‐Existing Diabetes

Arlin Delgado; Kaitlyn E. James; Andrea G. Edlow; Camille E. Powe; Lydia L. Shook

*Massachusetts General Hospital, Boston, MA*

4:00 PM ‐ 6:00 PM

**Objective**: Pre‐existing diabetes mellitus (DM)–diabetes identified prior to pregnancy ‐ is increasing in prevalence among pregnant individuals and is associated with adverse maternal and neonatal outcomes. Prior work suggests that fetal sex impacts underlying placental dysfunction leading to differential pregnancy risks.  The objective of this study is to assess the impact of fetal sex on placentally‐mediated adverse pregnancy outcomes in individuals with DM.

**Study Design**: This was a retrospective cohort study of 503 pregnant individuals with pre‐existing DM and known fetal sex receiving care at a major urban academic center between 1998‐2016. The exposure of interest was fetal/infant sex. The primary outcome was a composite of preterm birth, hypertensive disorder of pregnancy (HDP), small for gestational age (SGA), and stillbirth, and the secondary outcome was NICU admission.  A composite of large for gestational age (LGA), hypoglycemia, hyperbilirubinemia, shoulder dystocia, and respiratory distress syndrome (RDS) was assessed.  Mixed effects logistic regression models were built to assess the relationship between fetal sex and the pregnancy/neonatal outcomes, accounting for multiple pregnancies and adjusting for 1^st^ Trimester BMI, parity, and race/ethnicity.   

**Results**: Of the 503 pregnant individuals included, 258 pregnancies had a female fetus (51%) and 245 pregnancies had a male fetus (49%)–Table 1; no significant differences in sociodemographic or clinical characteristics were observed between groups. No significant differences in either composite outcome was identified, however there was a significantly higher likelihood of NICU admissions for males (adjusted odds ratio 1.83 [1.14, 2.94], p = 0.012, Table 2).

**Conclusion**: Although we did not identify differences in composite adverse pregnancy or neonatal outcomes in individuals with DM by offspring sex, the significantly higher rate of NICU admission suggests male neonatal vulnerability to the impact of maternal DM and warrants further investigation.

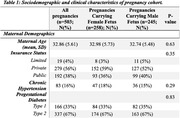

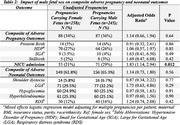

## 423 | Self‐Perceptions and Decision‐Making Factors regarding CBD Use Among Pregnant People

Mia Hodges^1^; Sarah Bader^2^; Emily Hardisty^3^; Neeta L. Vora^3^; Asha N. Talati^3^

*^1^University of North Carolina, Chapel Hill, NC; ^2^University of North Carolina at Greensboro, Greensboro, NC; ^3^University of North Carolina at Chapel Hill, Chapel Hill, NC*

4:00 PM ‐ 6:00 PM

**Objective**: To understand perceptions of risks and benefits, and factors influencing CBD use among pregnant people given limited data regarding CBD use during pregnancy.

**Study Design**: Participants were drawn from a parent study at UNC‐CH on CBD use and reproductive genetics. Eligible participants were pregnant within the last 3 years, had a history of CBD use, or were willing to discuss hypothetical scenarios of CBD use during pregnancy. One on one semi structured interviews were conducted by a single interviewer, recorded, coded, and transcribed. Domains of the interview guide included experience with CBD or other substances, decision making influences, and information seeking behaviors. Interviews were coded using grounded theory methods by three coders (MH, SHB, AT) using Dedoose software. Sampling for interviews was completed once thematic saturation was achieved.

**Results**: Ten participants were interviewed (Table 1), including one who used CBD during pregnancy and five who used CBD when not pregnant and chose not to continue during pregnancy.  Five major themes emerged: (1) Risk and benefit perceptions, (2) identifying trustworthy resources, (3) disclosure, (4) suggestions for practice, and (5) differences between pregnancies (Table 2). Importantly, participants reported frequently receiving discordant opinions when seeking information from various sources, identifying a gap in available data given the prevalence of CBD use. Additionally, participants revealed how relationship with an obstetric provider and perceptions of how a provider will receive information about CBD use, determined revealing use.

**Conclusion**: This study highlights the myriad of factors influencing pregnant patients' decisions on CBD use. These findings underscore the need for more research on CBD safety during pregnancy given the prevalence of use around gestation and first trimester exposure and can inform the development of educational tools for obstetric providers to better discuss CBD use with reproductive‐aged patients.

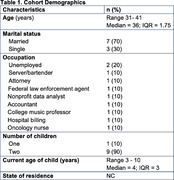

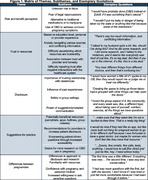

## 424 | Implementation Challenges for Prenatal Genetic Screening (PGS) at Federally Qualified Health Centers

Asha N. Talati^1^; Maura Jones Pullins^2^; Mia Hodges^2^; Emily Hardisty^1^; Madeline Dyke^1^; Neeta L. Vora^1^

*^1^University of North Carolina at Chapel Hill, Chapel Hill, NC; ^2^University of North Carolina, Chapel Hill, NC*

4:00 PM ‐ 6:00 PM

**Objective**: To identify implementation challenges for prenatal genetic services (PGS) (aneuploidy and carrier) at federally qualified health centers (FQHCs).

**Study Design**: Qualitative interview study of maternity care providers (MCPs) at FQHCs in NC. Eligible participants were those that see pregnant patients, order and provide counseling for prenatal genetic screening, and follow up results. One‐on‐one semi‐structured interviews were conducted by a single interviewer using an audio‐video platform, were recorded, and transcribed. Interviews focused on the following domains based on the Consolidated Framework for Implementation Research (CFIR) constructs: (1) perceived value of genetic screening; (2) perceptions of advancing prenatal genetic technologies; (3) individuals and structural barriers to PGS in public health settings; and (4) recommendations for improved implementation. Interviews were completed after categorical saturation was reached. Interviews were immediately analyzed after interview using a rapid qualitative analytic process.

**Results**: Twelve MCPs were interviewed across FQHCs in 12 counties in NC. 9 providers identified as maternal health program coordinators or managers (RN), and 3 providers identified as advanced practice providers (NP). Mean number of years in practice among the cohort was 6.6 (SD 2.4). In describing their patient populations,  all providers reported seeing patients who have Medicaid, presumptive Medicaid, or uninsured. Patient languages in all clinical settings were described as English and Spanish, with one clinic reporting a large Haitian Creole population. Several key themes were identified: (1) MCPs value PGS and describe inequities in access, informed consent, and  types of testing between public and private healthcare settings; (2) health literacy and cost are the most cited barriers to PGS implementation; (3) resources for education and expert consultation are needed in rural areas.

**Conclusion**: Resources to address health literacy, cost, and access to genetics expertise are necessary to improve implementation of PGS at FQHCs.

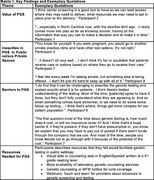

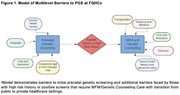

## 425 | Duration of High‐Dose Postpartum Oxytocin Infusion and Postpartum Bleeding Outcomes in Individuals Undergoing Vaginal Delivery

Ashley E. Shea^1^; Christina T. Blanchard^1^; Alan T. Tita^2^; Ashley N. Battarbee^1^; Akila Subramaniam^1^

*^1^Center for Women's Reproductive Health, University of Alabama at Birmingham, Birmingham, AL; ^2^University of Alabama at Birmingham, Birmingham, AL*

4:00 PM ‐ 6:00 PM

**Objective**: High‐dose prophylactic postpartum (PP) oxytocin (80 IU) has been suggested to reduce postpartum hemorrhage (PPH); however the optimal infusion duration is unknown.  We sought to compare rates of PPH and other bleeding outcomes between high‐dose (80 IU) oxytocin infused over 1 versus 4 hours.

**Study Design**: Secondary analysis of a database of all vaginal deliveries >22 weeks at a large academic referral center (1/1/2013‐12/31/2018). Our institution's protocol for PP oxytocin infusion was altered on 10/15/2015 for vaginal deliveries only from 80 IU/500 mL over 1 hour to 80 IU/500 mL over 4 hours with initial 100 mL bolus prior to 4 hour infusion. All vaginal deliveries with available outcomes were included. Patients were categorized as receiving oxytocin over 1 versus 4 hours, individually confirmed in the medication administration record. Primary outcome was a composite of estimated blood loss (EBL) >1000 mL, 2nd line uterotonic use, and any blood transfusion (secondary outcomes in Figure). Generalized estimating equation models were used to estimate adjusted odds ratios (aORs) and 95% confidence intervals (CI) using the 4 hour group as referent.

**Results**: Of 16,793 deliveries, 12,092 met inclusion criteria: 5,675 received 80U oxytocin for 1‐hour (47%), 6,417 (53%) for 4‐hours. There were multiple differences in baseline characteristics between groups (Table). Compared to the 4‐hour group, the 1‐hour group had increased odds of the primary composite (aOR 1.21, 95% CI 1.03‐1.42), driven by an increase in 2nd line uterotonic use (aOR 1.39,1.16‐1.67) (Figure). While there was no difference in EBL >1000 mL or blood transfusion, the 1‐hour group had increased odds of HCT decline >6% (aOR 1.83, 1.33‐2.54).

**Conclusion**: Infusion of PP high‐dose oxytocin over 1 hour compared to 4 hours is associated with increased odds of postpartum bleeding–mainly 2nd‐line uterotonic use and HCT decline > 6%. Given that 2nd line uterotonic use is clinician dependent, further investigation, utilizing less subjective outcomes such as transfusion, is needed to further define optimal high‐dose oxytocin duration.

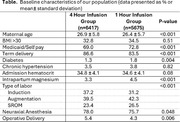

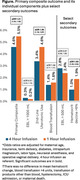

## 426 | Impact of Nicu Length of Stay on Outcomes of Birthing People with Opioid use Disorder

Ashley Boerrigter; Cynthia Cockerham; Julie Bowers‐Pryor; Ronald Clarkson; Gregory Hawk; Barbara V. Parilla

*University of Kentucky, Lexington, KY*

4:00 PM ‐ 6:00 PM

**Objective**: To determine the impact of NICU length of stay on health outcomes of birthing people with opioid use disorder (OUD). Primary outcome was EDPS and GAD‐7 scores.

**Study Design**: Longitudinal prospective cohort of pregnant persons receiving care through a comprehensive perinatal substance use treatment program between 2014 and June 2024. Characteristics of every patient who participated in the program were analyzed regardless of prenatal program participation or complete delivery outcome information; a minimum of postpartum program participation and neonatal outcomes were required for inclusion in the study. Participants were divided into groups based on whether their infant stayed more or fewer than 15 days in the NICU. Fisher's exact and two‐sample t‐tests were utilized for statistical analysis.

**Results**: There was no significant difference in postpartum EDPS and GAD‐7 scores when groups were analyzed by infant's duration of stay in the NICU (<15 days, n = 183 or 15+ days, n = 106). However, EPDS score >13 at weeks 11–15 postpartum was observed in 33% of patients with infant NICU stays >15 days as compared to 0% with shorter stays, though this was not statistically significant (p = 0.4667). Birthing parents of infants who stayed < 15 days in the NICU were more likely to be exclusively breastfeeding at time of discharge (38.1% vs 32.6%, p = 0.0331) and room in with their infant (70.6% vs 47.1%, p = 0.001). There was no significant difference in attendance at postpartum visits (74% vs 72%, p = 0.6106).

**Conclusion**: When stratified at the 15‐day mark, duration of infant's NICU stay did not appear to be associated with any significant difference in birthing parents’ EDPS or GAD‐7 scores. However, 33% of parents experienced an EPDS score suggestive of depression as compared to 0% in the shorter stay group. Further studies are needed to evaluate the mental health of parents when their neonate has an extended hospital stay for neonatal opioid withdrawal syndrome (NOWS). Breastfeeding and rooming‐in are strategies associated with shorter stays for NOWS, which our study also found.

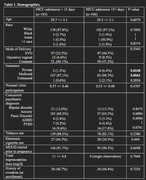

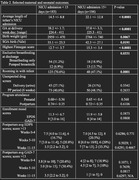

## 427 | Personalized Infant Birth Weight Percentile Charts for Accurate Assessment of Neonatal Growth using Machine Learning

Natalie Suder^1^; Ashley Zimmermann^2^; Rachel Kim^3^; Karen Huang^3^; Maria Teresa Benedetto^1^; Mio Sawai^1^; Teresa Cheon^1^; Yuzuru Anzai^1^

*^1^Lenox Hill Hospital, NY; ^2^Northwell Lenox Hill Hospital, New York, NY; ^3^University of Chicago, Chicago, IL*

4:00 PM ‐ 6:00 PM

**Objective**: Identifying small for gestational age (SGA) and large for gestational age (LGA) neonates is imperative to manage potential neonatal complications. The Hadlock growth curve, the most widely used standardized growth curve in the United States, overlooks maternal factors such as race, height, BMI, and pregnancy weight gain, which are known to impact fetal weight. This study aims to create a personalized birth weight percentile chart to improve accuracy of diagnosing SGA and LGA neonates.

**Study Design**: Using the 2022 CDC natality dataset, a least squares quantile regression model was developed using Python to predict the 10th and 90th percentile birth weights based on maternal factors. To improve model accuracy, gestational diabetes, gestational hypertension, plurality, tobacco use, infant sex, and gestational age (GA) were included. Polynomial regressions determined the best‐fit degrees. The model focused on pregnancies from 27 to 42 weeks GA, excluding extreme birth weights (< 200 grams or > 5000 grams) and unknown parameters (n = 3,428,570). Validity of the model was confirmed using an 80:20 split for testing and training, respectively.

**Results**: Among the factors in the model, plurality had the strongest effect on determining the fetal weight, followed by GA, maternal race, cigarette smoking, fetal gender, and maternal height, even though GA should not alter the percentile. Pre‐pregnancy maternal BMI and pregnancy weight gain showed much smaller effects in this model. The quantile loss at the 10th percentile was 66 grams and 90th percentile was 72 grams.

**Conclusion**: The model was trained using a large, national dataset and shows high accuracy with low quantile losses; therefore, this model can supplement physicians’ diagnoses of SGA and LGA and aid in appropriate delivery preparations. However, the model results should be interpreted with caution because medical complications and tobacco smoking status used for the model are known factors that can affect fetal growth. In the next model, we aim to determine the SGA threshold that is associated with adverse fetal outcomes.

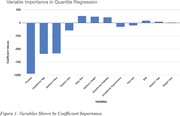

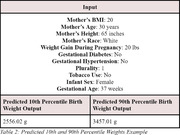

## 428 | Interactive Lecture Improves Resident Knowledge and Comfort with Periviable Delivery Counseling

Ashli Gibb; Carolyn Chatterton; Fatima Ali; Kara Patek

*Detroit Medical Center/Wayne State University, Detroit, MI*

4:00 PM ‐ 6:00 PM

**Objective**: Counseling patients at risk for periviable delivery (PVD) is a challenging part of obstetrics. Available resources for PVD counseling (PVDC) include previously published best practices and the National Institute for Child Health and Human Development (NICHD) Calculator for Extremely Preterm Birth Outcomes. Given the necessary nuanced provider knowledge, ethical complexity, and emotional environment of a PVD, we assessed ObGyn resident PVD knowledge and comfort with counseling before and after an educational intervention.

**Study Design**: ObGyn residents at a large urban center were given a pre‐survey to assess experience, knowledge, and comfort with PVDC. An interactive lecture was given, with case review and group discussion. Those unable to attend lecture were given a reading for comparison. A post‐survey was distributed. Paired t‐test was used for statistical analysis.

**Results**: 39 residents participated, and 32 (82.1%) completed the pre‐survey. Collectively, residents reported counseling 100 patients a year on PVD, and receiving 1.6 hours of PVD education a year. A majority (87.5%) felt that patients poorly understood after counseling. 21 residents completed the post‐survey: 18/26 post‐lecture and 3/13 post‐reading. Post‐lecture, comfort with PVDC significantly improved for all residents (mean 1.9 vs 3.6 on a 5‐point Likert Scale; p< 0.01) and PGY1s (mean 1.5 vs 3.2; p = 0.01). Knowledge also improved as 50% of residents named all 5 NICHD calculator factors post‐lecture, versus 12.5% pre‐survey. 100% of residents felt the lecture was very or extremely valuable, and 94.4% plan to implement changes in their practice. Residents post‐reading had minimal improvement in PVDC comfort, felt it was slightly or moderately valuable, and none would implement changes.

**Conclusion**: At centers with a high incidence of PVD, resident education is crucial to counseling and patient care. Interactive lectures with an opportunity to practice counseling significantly improved comfort and knowledge. These findings highlight a need for high‐quality resident education and resources to improve care for patients at risk for PVD.

## 429 | Cost‐Effectiveness of Antepartum Versus Postpartum Treatment of Latent Tuberculosis Infection

Ava D. Mandelbaum; Megha Arora; Sarah K. Dzubay; Aaron B. Caughey

*Oregon Health & Science University, Portland, OR*

4:00 PM ‐ 6:00 PM

**Objective**: Treatment of latent tuberculosis infection (LTBI) during pregnancy is typically reserved for high‐risk cases due to concerns about potential side effects. However, pregnancy provides a unique interaction with the healthcare system, offering an opportunity to treat LTBI and prevent future progression to active tuberculosis (TB). This study aimed to evaluate the health outcomes and cost‐effectiveness of antepartum versus postpartum treatment among pregnant individuals with LTBI.

**Study Design**: A decision‐analytic model using TreeAge software was constructed to compare the outcomes and cost‐effectiveness of antepartum versus postpartum treatment of LTBI. The theoretical cohort included 20,000 individuals with LTBI based on the number of annual pregnancies and prevalence of LTBI in the United States. Outcomes included the number of complete treatments, drug‐induced hepatotoxicity, progression to active TB, fatality from active TB, and cases of untreated latent TB based on data demonstrating lower adherence and follow‐up in the postpartum period. Model inputs were derived from the literature. The threshold for cost‐effectiveness was $100,000/ quality‐adjusted life year (QALY). Sensitivity analyses were performed to assess the robustness of the results.

**Results**: Compared to postpartum treatment, the antepartum treatment strategy resulted in 4,217 additional treatment completions, 239 fewer cases of progression to active TB, and 17 fewer fatalities from active TB (Table 1). Additionally, the postpartum group had 4,520 cases of untreated LTBI related to the rate of lower follow‐up postpartum. Antepartum treatment was the cost‐effective and dominant strategy, resulting in cost savings of $8,555,587 and an increase in 4,266 QALYs relative to postpartum treatment.

**Conclusion**: The antepartum treatment strategy for LTBI was cost‐effective and resulted in better health outcomes compared to postpartum treatment. These findings may inform future policies that integrate LTBI treatment into prenatal care to potentially improve outcomes and enhance resource allocation.

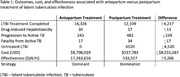

## 430 | Management of Placenta Accreta Spectrum with Cesarean Hysterectomy Versus Conservative Management: a Cost‐Effectiveness Analysis

Ava D. Mandelbaum^1^; Binh Luu^2^; Andrew H. Chon^1^; Tania F. Esakoff^3^; Deirdre J. Lyell^4^; Aaron B. Caughey^1^

*^1^Oregon Health & Science University, Portland, OR; ^2^Oregon Health and Science University, Vancouver, WA; ^3^Cedars Sinai Medical Center, Los Angeles, CA; ^4^Stanford University, Palo Alto, CA*

4:00 PM ‐ 6:00 PM

**Objective**: Cesarean hysterectomy is the standard treatment for placenta accreta spectrum (PAS), but alternative conservative management strategies (e.g., leaving the placenta in situ), have been proposed to preserve fertility. This study aimed to evaluate the cost‐effectiveness of conservative management for PAS.

**Study Design**: A decision‐analytic model was built using TreeAge software to compare the outcomes and cost‐effectiveness of cesarean hysterectomy versus conservative management. The theoretical cohort included 5,000 individuals in the U.S. with PAS. Maternal outcomes included severe postpartum hemorrhage (>3000 mL estimated blood loss), adjacent organ injury, severe maternal morbidity (including intensive care unit admission, sepsis, and disseminated intravascular coagulation), endometritis, maternal death, and readmission. The number of hysterectomies performed within and after 24 hours of delivery (primary and delayed, respectively) was calculated for the conservative management group. Model inputs were derived from the literature. The threshold for cost‐effectiveness was set at $100,000 per quality‐adjusted life year (QALY). Sensitivity analyses were performed to assess the robustness of the results.

**Results**: Cesarean hysterectomy resulted in 2,137 more severe postpartum hemorrhages, 413 more adjacent organ injuries, and 516 more cases of non‐hemorrhage SMM, and 8 more maternal deaths compared to conservative management. Conversely, conservative management led to 542 more cases of endometritis and 1,268 more readmissions. Additionally, 347 primary and 202 delayed hysterectomies were performed in the conservative management group. Conservative management resulted in a $64 million cost reduction and an increase of 7,609 QALYs relative to cesarean hysterectomy.

**Conclusion**: Compared to cesarean hysterectomy, conservative management was cost‐effective, with distinct trade‐offs in maternal outcomes, such as reduced severe hemorrhage and increased endometritis and readmission. Additional studies are needed to refine the selection of patients undergoing conservative management for fertility preservation.

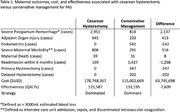

## 431 | Patient‐Similarity Tool Enhancing Decision‐Making on Whether to Place Cervical Cerclage

Avihu Krieger^1^; Rakefet Yoeli‐Ullman^2^; Abraham Tsur^3^

*^1^Sheba Medical Center, Tel Aviv, Tel Aviv; ^2^Sheba Medical Center, Ramat Gan, HaMerkaz; ^3^Sheba Medical Center, Ramat Gan, Tel Hashomer, Israel, Ramat Gan, HaMerkaz*

4:00 PM ‐ 6:00 PM

**Objective**: Decision‐making for cervical cerclage placement is complex and requires consideration of specific patient characteristics. We sought to develop a *patient similarity tool* to augment clinical decision‐making and enhance shared decision‐making based on the clinical course of similar patients. Patient similarity identifies patients with similar clinical characteristics to predict outcomes and personalize treatment plans.

**Study Design**: We used a retrospective cohort of all pregnancies at a tertiary center from 2010‐24, including all individuals with discussions considering cervical cerclage. The list of patients and their clinical variables served as the analytic DATABASE for the patient similarity tool. We defined 16 variables for similarity assessment that were available in the electronic health record. An additional seven variables will require manual collection: cervical dilation, membranes bulging, cervical length, amniotic fluid sludge, uterine activity, WBC, and CRP. Based on the index patient's similarity parameters, the tool detects similar patients and describes their maternal and neonatal outcomes with and without cerclage.

**Results**: Among 138,439 deliveries during the study period, 684 clinical scenarios considering cervical cerclage were captured. Cerclage was performed in 343 (50.1%) of these cases. Table 1 compares individuals with and without cerclage based on the variables selected for similarity assessment. Significant differences were observed in maternal age, gravidity, previous painless dilation, previous cerclage, last pregnancy gestational age (GA) at delivery, spontaneous conception, multifetal pregnancy, and GA at decision time. Figure 1 illustrates an index patient and presents similar patients and their outcomes with and without cerclage.

**Conclusion**: Developing a patient similarity tool to augment decision‐making and decision sharing regarding cervical cerclage placement is feasible. We speculate that our proposed tool may improve data‐driven decision‐making and empower patient participation in shared decision‐making.

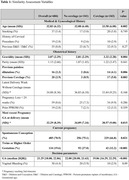

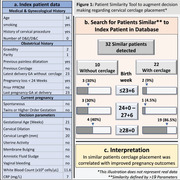

## 432 | Early Pregnancy Levels of Cholesterol in Maternal Serum and Pregnancy Outcomes

Ayal Haimov^1^; Eyal Sheiner^2^; Tamar Wainstock^2^

*^1^Ben Gurion University of the Negev, Ramat Gan, HaMerkaz; ^2^Department of Obstetrics and Gynecology, Soroka University Medical Center, Ben‐Gurion University of the Negev, Beer‐Sheva, Israel., Beer Sheva, HaDarom*

4:00 PM ‐ 6:00 PM

**Objective**: Elevated maternal serum lipid concentrations during late pregnancy are associated with pregnancy complications. The goal of this study was to investigate the association between early pregnancy maternal serum levels of cholesterol and selected pregnancy outcomes.  

**Study Design**: A prospective cohort study was conducted, including pregnant women with a singleton pregnancy, recruited between 11‐13 gestational weeks. The independent variable was maternal serum level of cholesterol (high cholesterol was defined as >200 mg/dL). The incidence of the following pregnancy outcomes was compared between women with high and low cholesterol: abortion, gestational hypertension, gestational diabetes mellitus, gestational length, preterm birth, birth weight, low birthweight, macrosomia, cesarean delivery and low 5 minutes Apgar score.

**Results**: The study included 201 women. The incidence of high cholesterol was 35% (n = 70). Mean birthweight was higher among women with high cholesterol (Table 2, 3392.71 ± 426 versus 3220.78 ± 448 grams, p‐value = 0.01). Similarly, women with high cholesterol were more likely to deliver a macrosomic newborn (10.1% versus 3.2%, p‐value = 0.057). Multivariable linear model, adjusted for gender and maternal diseases, found that an increase in one unit of cholesterol was significantly related with an increase of 2.85 grams in birthweight (95% CI = 0.98 ‐ 4.73, p‐value = 0.003). Multivariable logistic model which adjusted for gender and maternal BMI, found that every unit increase of cholesterol was significantly related with an increased risk for macrosomia (OR = 1.025, 95% CI = 1.003–1.047, p‐value = 0.025). No association was found between cholesterol and others pregnancy outcomes.

**Conclusion**: Higher birthweight and a risk for macrosomia were found among women with high early pregnancy cholesterol levels, even after controlling for maternal BMI. Lowering maternal cholesterol levels may aid in prevention of pregnancy complications associated with high birthweight. 

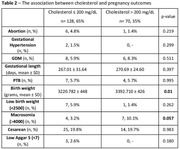

## 433 | Early Pregnancy Levels of Acute Phase Reactants in Maternal Serum and Pregnancy Outcomes

Ayal Haimov^1^; Eyal Sheiner^2^; Tamar Wainstock^2^

*^1^Ben Gurion University of the Negev, Ramat Gan, HaMerkaz; ^2^Department of Obstetrics and Gynecology, Soroka University Medical Center, Ben‐Gurion University of the Negev, Beer‐Sheva, Israel., Beer Sheva, HaDarom*

4:00 PM ‐ 6:00 PM

**Objective**: Inflammation and infection within the fetoplacental unit have been suggested as a mechanism involved in pregnancy complications. The goal of this study was to investigate the association between early pregnancy maternal serum levels of the following acute phase reactants (ARPs): CRP (C‐ reactive protein), albumin, triglyceride, HDL and selected pregnancy outcomes.  

**Study Design**: A prospective cohort study was conducted, including pregnant women with a singleton pregnancy, recruited between 11‐13 gestational weeks. The independents variables were serum levels of the following ARPs: CRP, Albumin, Triglyceride, and HDL. The incidence of the following pregnancy outcomes was compared between women with high and low levels of the measures APRs: abortion, hypertensive disorders, gestational diabetes mellitus, gestational length, preterm birth (PTB), birth weight, low birthweight, macrosomia, cesarean delivery and low 5 minutes Apgar score.

**Results**: The study included 201 women. No association was found between the ARPs and pregnancy outcomes (Table 2), for instance, the incidence of PTB were 5.8% (n = 8) versus 5.5% (n = 3), among women with high and low HDL, respectively (p = 0.917), and 5.3% (n = 6) versus 6.6% (n = 5) among women with high and low Albumin, respectively (p = 0.704). Birth weight was 3220.75 ± 403 versus 3317.51 ± 467 grams among women with low and high CRP, respectively (p = 0.160), and 3296.39 ± 433.26 versus 2676.05 ± 464.76 grams among women with high and low Albumin, respectively (p = 0.758). These findings were supported by multivariable logistic and linear models, which adjusted for maternal BMI and maternal age.

**Conclusion**: Based on the current study, early pregnancy levels of ARPs are not associated with an increased risk for adverse pregnancy outcomes. 

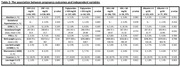

## 434 | Amyloid‐β (Aβ) Proteoforms Impair Trophoblast Survival and Function ‐ Relevance to Defective Placentation in Preeclampsia

Bani Medegan Fagla^1^; Cielo Dela Rosa^1^; Ana C. Valencia‐Olvera^2^; Guomao Zhao^1^; Almudena Veiga‐Lopez^2^; Juan M. Weng^2^; Irina A. Buhimschi^1^; Elvis Ticiani^2^

*^1^University of Illinois at Chicago, College of Medicine, Chicago, IL; ^2^University of Illinois at Chicago College of Medicine, Chicago, IL*

4:00 PM ‐ 6:00 PM

**Objective**: Misfolding of proteins and their subsequent assembly into supramolecular aggregates are key mechanisms involved in neurodegenerative diseases such as Alzheimer's disease (AD). These aggregates range from small prefibrillar cytotoxic oligomers to large β‐sheet rich fibrils that accumulate in tissues and disrupt normal function. In AD, the accumulation of amyloid‐β (Aβ) oligomers (O_Aβ_) and fibrils (F_Aβ_) in the brain is a disease hallmark. Excess Aβ aggregates were recently detected in the serum, urine, and placenta of patients with preeclampsia, which is now considered a protein misfolding disease of pregnancy. Whether Aβ aggregates directly impact placental function thereby contributing to pathogenesis of preeclampsia has yet to be elucidated. We aimed to characterize the effect of different Aβ proteoforms on trophoblast cell survival and function.

**Study Design**: We exposed an immortalized human extravillous trophoblast (EVT) cell line (HTR8/SVNeo) to exogenous unaggregated (U_Aβ_), O_Aβ_, or F_Aβ_ preparations (0.001‐10µM) and assessed their effect on cell survival by measuring cytotoxicity (MTT assay), migration (scratch assay) and invasion (Transwell assay ± epidermal growth factor [EGF], an essential regulator of placental development). Data from n = 3‐5 independent experiments was analyzed. A dual apoptosis/necrosis assay was used to determine the mechanism of cell death.

**Results**: **1)** Both O_Aβ_ and F_Aβ_ decreased EVT survival with O_Aβ_ having a more profound effect (p< 0.001); at higher concentrations, U_Aβ_ also decreased cell viability likely due to spontaneous oligomerization (Fig. **A**). **2)** EVT death occurred primarily via apoptosis (not shown). **3)** While O_Aβ_ decreased EVT migration (increased gap size), F_Aβ_ increased cell migration compared to vehicle control (Fig. **B**). **4)** Both O_Aβ_ and F_Aβ_ inhibited EGF‐mediated invasion (p = 0.017) without affecting baseline invasion (Fig. **C**).

**Conclusion**: Presence of excess Aβ aggregates during placental development has the potential to contribute to preeclampsia pathogenesis by disrupting key cellular processes such as EVT survival, migration and invasion.

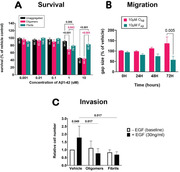

## 435 | APOE4 Genotype Increases Susceptibility to Fetal Growth Restriction in the Context of Preeclampsia

Bani Medegan Fagla^1^; Cielo Dela Rosa^1^; Savita Sundar^1^; Erykah Walton^1^; Jason York^1^; Guomao Zhao^1^; Aswathi Jayaram^1^; Leon M. Tai^1^; Mert Ozan Bahtiyar^2^; Catalin S. Buhimschi^1^; Irina A. Buhimschi^1^

*^1^University of Illinois at Chicago, College of Medicine, Chicago, IL; ^2^Yale University, New Haven, CT*

4:00 PM ‐ 6:00 PM

**Objective**: *APOE4, *one of three alleles of the lipid regulating gene *APOE*, is the greatest genetic risk factor for sporadic Alzheimer's disease (AD) particularly in females. Preeclampsia (PE), a pregnancy‐specific disorder, is characterized by abnormal deposition of misfolded proteins in the placenta, analogous to the hallmark protein aggregates in AD brain. We sought to investigate in a human cohort the association between *APOE* genotype and risk of PE with or without fetal growth restriction (FGR; EFW < 10%). Additionally, the effect of *APOE4* was investigated mechanistically in a transgenic mouse model where the mouse *ApoE* gene was substituted by either human *APOE4* or *APOE3* (the common allele).

**Study Design**: We genotyped 181 consecutively enrolled maternal‐fetal dyads divided by pregnancy phenotype in three clinical groups: **1)** PE without FGR (n = 56), **2)** PE with FGR (n = 21), and **3)** non‐PE pregnancies (n = 104). DNA was extracted from maternal or cord blood and subjected to PCR. Risk association was determined by logistic regression. For mechanistic studies, we induced a PE‐like syndrome in pregnant APOE4^+/+^ and APOE3^+/+^ mice using the reduced uteroplacental perfusion pressure (RUPP) procedure. Control mice underwent sham surgeries (n = 6‐7/group/genotype). Endpoints consisted of kidney morphology (glomerular size), fetal weights, placental efficiency, and placental ApoE mRNA expression by RT‐qPCR.

**Results**: **1)** In the human cohort, fetal carriage of at least one *APOE4* allele was associated with increased odds of PE with FGR (p = 0.043, Fig. 1); **2)** An interaction of *APOE4* with female fetal sex was further identified (p = 0.024); **3)** In mice, placental efficiency was reduced post‐RUPP in both genotypes (Fig. 2A) but only APOE4^+/+^ had decreased fetal weights (Fig. 2B) and kidney injury (p = 0.014, Fig. 2C); **4)** RT‐qPCR of mouse placenta indicated significantly upregulated ApoE mRNA in response to RUPP in both genotypes.

**Conclusion**: ApoE participates to placental homeostasis during pregnancy. *APOE4* may have a reduced capacity to maintain optimal fetal growth in the context of PE‐associated placental dysfunction compared to *APOE3*.

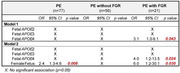

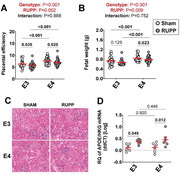

## 436 | Changes in Uterine, Placental Metrics, and Vascularization in Placenta Left In Situ for PAS Management

Barouyr Ajemian^1^; Donatella Gerulewicz Vannini^1^; Eleazar E. Soto^2^; Farah H. Amro^2^; Baha M. Sibai^2^; Edgar A. Hernandez‐Andrade^2^

*^1^University of Texas Health Science Center in Houston, McGovern Medical School, Houston, TX; ^2^McGovern Medical School at UTHealth Houston, Houston, TX*

4:00 PM ‐ 6:00 PM

**Objective**: In our center, one of our placenta accreta spectrum (PAS) management approaches is leaving the placenta left in situ (LIS) for either delayed hysterectomy (6‐7 weeks later), or uterine preservation. We aim to describe changes in uterine and placental metrics and vascularization in individuals with placenta LIS after delivery. We compared those with planned delayed hysterectomy or uterine preservation, to those with unscheduled hysterectomy (UH).

**Study Design**: In this retrospective cohort, 32 individuals with placenta LIS were evaluated. Our management approach includes postpartum ultrasound biweekly until hysterectomy or passage of placental tissue. The individuals were divided into two groups, group I: successful planned procedure (scheduled hysterectomy or uterine preservation), group II: UH (defined as that occurring prior the scheduled date mainly due to vaginal bleeding). Measurements included: uterus length and height, and placental thickness. Directional color Doppler ultrasound was applied to evaluate the lower uterine segment using the following settings PRF = 22‐25 cm/sec, medium filter, and 1.4 dB gain. Images were analyzed using Image J software. Quantitative vascularization (QV) was defined as the percentage of pixels containing color information over the total number of pixels in the studied area. Average change per week of the mentioned parameters were calculated. Differences in metrics and QV between UH and successful conservative management were calculated.

**Results**: Of 32 patients, 17 (53%) had a successful planned procedure (10 scheduled hysterectomy and 7 retained their uterus) and 15 (47%) had an UH. Differences between the two groups are presented in table 1. The reduction in uterine metrics and QV in those with successful conservative management was more pronounced, but not statistically significant.

**Conclusion**: This is the first report showing longitudinal change in uterine, placental metrics and QV in those with the placenta LIS. More studies are needed to identify clinical variables which may predict successful conservative management of PAS.

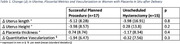

## 437 | The Impact of Weight Gain on Pregnancy Outcomes in Patients with Low BMI

Taha Elfarra^1^; Leah M. Hefelfinger^2^; Jessica Lin^3^; Braxton Forde^2^

*^1^University of Cincinnati, Cincinnati, OH; ^2^University of Cincinnati College of Medicine, Cincinnati, OH; ^3^Cincinnati Children's Hospital Medical Center, Cincinnati, OH*

4:00 PM ‐ 6:00 PM

**Objective**: The association between adverse pregnancy outcomes and obesity is established, however the impact of low maternal weight upon outcomes is much less studied. We sought to evaluate the odds of adverse pregnancy outcomes in the setting of low pre‐pregnancy BMI as well as if these risks are mitigated with adequate weight gain in pregnancy. 

**Study Design**: Retrospective cohort study of all United States births in 2021 using National Vital Statistics US birth certificate data. Pregnancy demographics, as well as maternal and neonatal outcomes were compared between a referent group of normal pre‐pregnancy BMI with adequate pregnancy weight gain and 3 investigational cohorts: low pre‐pregnancy BMI with either adequate, inadequate, or greater than recommended pregnancy weight gain. Multivariate logistical regression models were used to calculate the odds of adverse maternal and neonatal outcomes. 

**Results**: During study period, 520,552 patients had normal pre‐pregnancy BMI and adequate weight gain, compared to 40,144 patients 36,650 patients, and 26,155 patients with low pre‐pregnancy BMI and adequate, inadequate, or greater than recommended weight gain, respectively. Demographical data was significantly different amongst groups (Table 1). Interestingly, after adjustment for differences in demographics, low pre‐pregnancy BMI was associated with adverse neonatal outcomes only when weight gain was inadequate (aOR of composite adverse neonatal outcomes 1.6, 95% CI 1.6‐1.7, Figure 1), whereas low pre‐pregnancy BMI with either adequate or greater than recommended weight gain has similar neonatal outcomes (aOR 1.0, 1.0‐1.0, aOR 1.0, 0.9‐1.0, respectively). Of equal interest, low pre‐pregnancy BMI was associated with adverse maternal outcomes regardless of weight gain in the pregnancy (Figure 1).

**Conclusion**: Low pre‐pregnancy BMI is associated with adverse neonatal outcomes only in the setting of inadequate pregnancy weight gain, whereas low pre‐pregnancy BMI is associated with adverse maternal outcomes regardless of weight gain in the pregnancy.

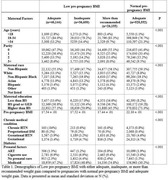

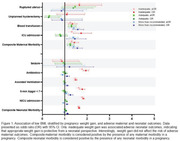

## 438 | The Importance of the Number of Prenatal Visits on Pregnancy Outcomes

Anna Gehret^1^; Braxton Forde^2^

*^1^Ohio University College of Medicine, Athens, OH; ^2^University of Cincinnati College of Medicine, Cincinnati, OH*

4:00 PM ‐ 6:00 PM

**Objective**: Most guidelines recommend roughly 11‐15 prenatal visits in each pregnancy, with possibly more for high‐risk pregnancies. While no prenatal care is associated with adverse outcomes, the true risk of limited prenatal care is unknown. Thus, we sought to evaluate the association of the number of prenatal visits with pregnancy outcomes.

**Study Design**: Retrospective cohort of all United States births in 2022 using National Vital Statistics US birth certificate data. Pregnancy demographics, as well as maternal and neonatal outcomes were compared between a referent group of patients that attended 11‐15 prenatal visits with 3 investigational cohorts: patients with < 5 prenatal visits, 5‐10 prenatal visits, and > 15 prenatal visits. Linear regression models were used to calculate the attributable effect of prenatal visit number upon delivery gestational age, and multivariate logistic regression was used to calculate the odds of adverse maternal and neonatal outcomes.

**Results**: During study period, 232,616 patients had < 5 prenatal visits, while 1,341,990 had 5‐10, 1,702,481 had 11‐15, and 320,008 had > 15 visits. Demographics were significantly different amongst groups (Table 1).  While all study groups had increased rates of adverse outcomes, the findings were strongest with < 5 visits, which was attributable to a 1.5 week (95% CI 1.44‐1.54) decrease in gestational age at delivery, even after adjustment for known risk factors of preterm birth. Conversely, attending 5‐10 visits attributed to only 0.66 week (95% CI 0.64‐0.68) decrease, and attending > 15 visits attributed to a clinically irrelevant 0.19 week decrease in delivery gestational age. Similar trends were identified when evaluating maternal and neonatal outcomes, in which < 5 prenatal visits significantly increased the odds of adverse maternal and neonatal outcomes (Figure 1).

**Conclusion**: Attending < 5 prenatal visits is strongly associated with adverse pregnancy outcomes. As patients attend more visits, the odds of adverse outcomes decrease. This highlights the extremely important role of routine prenatal care upon maternal and child health. 

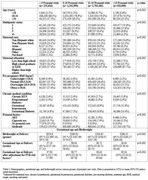

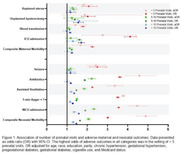

## 439 | Birth Experiences of Discrimination on Labor and Delivery

Bridget C. Huysman^1^; Paige Wilder^1^; Maya Bussey^2^; Lori Atwood^1^; Mia Malcolm^2^; Monique Gill^2^; Rachel Witt^3^; Ebony B. Carter^4^; Eve Colson^2^; Candice Woolfolk^5^

*^1^Washington University in St. Louis, St. Louis, MO; ^2^Washington University in St. Louis/Barnes‐Jewish Hospital, St. Louis, MO; ^3^University of Minnesota, Minneapolis, MN; ^4^University of North Carolina, Chapel Hill, NC; ^5^Washington University School of Medicine in Saint Louis, St. Louis, MO*

4:00 PM ‐ 6:00 PM

**Objective**: Discrimination and racism impact obstetric care and approximately 1 in 5 birthing people will report mistreatment during maternity care. We sought to assess perceived discrimination experienced by birthing persons at the time of their delivery admission.

**Study Design**: This is a prospective survey of 129 birth people presenting for delivery from 03/2024 to 05/2024 using the Discrimination in Medical Settings Scale (DMSS) administered prior to discharge. Birthing people admitted for an indication not related to delivery, who delivered off L&D or precipitously, or unable to speak English were excluded. DMSS scores were compared between people of color (BIPOC) and White and assessed by reports of any discrimination, and DMSS scores >75^th^ percentile (score 8) and  ≥90^th^ percentile (score 11). Survey responses were compared between groups using the Mann Whitney U test and multivariable logistic regression was used to estimate adjusted odds ratios(aOR) and 95% confidence intervals (CI) after adjusting for relevant confounders. 

**Results**: Prior to discharge, 176 birthing people were approached to complete the survey and 47 declined to participate. Over 60% of the cohort identified as a person of color. Comparing by race, BIPOC and White birthing people differed by highest educational level achieved and insurance and employment status. In bivariate analysis, there was a statistically significant difference in reported DMSS scores ≥90^th^ percentile between BIPOC and White birthing people with 5 times higher likelihood of reporting discrimination (14 (17%) vs. 2 (4%), p = 0.03, OR 5.42 [95% CI 1.18, 25.00])(Table 1). There was a statistically significant difference in security calls while in medical care between Black and White birthing people, respectively (13 (20%) vs. 5 (7%), p = 0.04)(Table 2).

**Conclusion**: Discrimination is experienced more by people of color on labor and delivery. Calls made to security calls by staff were more commonly experienced by Black birthing people. Future work is needed to explore how experiences of discrimination contribute to obstetric outcomes and care seeking.  

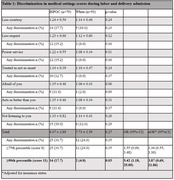

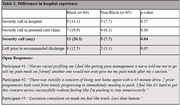

## 440 | Association of Transversus Abdominus Plane Block and Postoperative Opioid Consumption Following Planned Cesarean Hysterectomy

Brittany M. Hood; Jessica C. Ehrig; Michael Hofkamp

*Baylor Scott and White Health, Temple, TX*

4:00 PM ‐ 6:00 PM

**Objective**: The rate of placenta accreta spectrum is 1 in 272. To combat the opioid epidemic, alternative pain management strategies including multimodal analgesia have been investigated. We hypothesized that patients who underwent planned cesarean hysterectomy (C‐HYST) who received a transversus abdominus plane (TAP) block would have lower 24‐hour postoperative opioid consumption compared to patients who did not.

**Study Design**: Our IRB approved the study and waived informed consent. Patients with C‐HYST who did not require postoperative mechanical ventilation at our hospital from July 1, 2014 to December 31, 2022 were eligible for inclusion. Our primary outcome was 24‐hour postoperative opioid consumption. We converted opioid use to morphine milligram equivalents (MME). For patients with patient‐controlled analgesia (PCA), the closest documented entry to 24 hours was used to calculate opioid consumption. Demographic, physical, and clinical data were recorded.

**Results**: Demographic and physical characteristics of patients with TAP block following C‐HYST with (n = 23) and without TAP block (n = 26) were no different. Patients with TAP block following C‐HYST had a 24‐hour opioid consumption of 30.8 + 29.8 mg compared to 80.4 + 59.4 mg for patients without a TAP block (p< 0.001). Patients with PCA (n = 12) had an average deviation in documentation from the 24‐hour period of 41 minutes. For patients with TAP block, the average time from wound closure to TAP block was 69 minutes. 

**Conclusion**: Patients with TAP block following C‐HYST had statistically significant reductions in 24‐hour opioid consumption compared to patients without TAP block. A limitation of this study was that patients without TAP block had statistically significant lower acetaminophen consumption compared to patients with TAP block. Increased acetaminophen administration may explain a modest decrease in opioid consumption but is unlikely to fully explain the magnitude of reduction observed in our study. Another limitation was imprecise data for patients with intravenous PCA. TAP blocks should be considered for patients who undergo C‐HYST procedures.

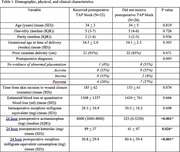

## 441 | Pregnancy Under Fire: Disparities in Firearm‐Related Injuries During Pregnancy in the United States, 2017‐2021

Brittany Arditi^1^; Rachel Herz‐Roiphe^2^; Alexander M. Friedman^1^; Adina R. Kern‐Goldberger^3^; Cynthia Gyamfi‐Bannerman^4^; Kartik K. Venkatesh^5^; Timothy Wen^6^

*^1^Columbia University Irving Medical Center, New York, NY; ^2^Columbia University Medical Center, New York, NY; ^3^Cleveland Clinic Lerner College of Medicine, Cleveland, OH; ^4^University of California, San Diego, San Diego, CA; ^5^The Ohio State University, Columbus, OH; ^6^University of California, San Diego, Irvine, CA*

4:00 PM ‐ 6:00 PM

**Objective**: Firearm violence is a preventable public health crisis in the US, and pregnant patients are not immune from this epidemic. Studies have shown that pregnant individuals are more likely to die due to homicide than the three leading obstetric causes of maternal mortality. Our objective was to evaluate disparities in firearm‐related injuries (FRI) during pregnancy.

**Study Design**: A cross‐sectional analysis of delivery hospitalizations with FRI was conducted using the Nationwide Inpatient Sample from 2017‐2021. We analyzed trends of FRI stratified by self‐reported race, payor, hospital region, and location/teaching status. Trend significance was determined using the Cochran‐Armitage test. Multivariate logistic regression analyses, adjusting for patient and hospital factors, were performed to evaluate the effects of payor, self‐reported maternal race, hospital region, and hospital teaching status on the incidence of FRI.

**Results**: Of 17.5 million deliveries, 6,365 were complicated by FRI (3.6 per 10,000 delivery hospitalizations). In unadjusted analysis, Black patients (OR 1.35, 95% CI 1.15‐1.58) and patients delivering at urban teaching hospitals (OR 1.44, 95% CI 1.1‐1.88) experienced higher rates of FRI; this finding did not hold after adjustment for patient and hospital factors. In both univariable and adjusted analyses, patients with public insurance were at increased risk of FRI (Medicare aOR: 3.10, 95% CI 2.07‐4.66; Medicaid aOR 1.33, 95% CI 1.12‐1.59 vs. private insurance). Rates of FRI were lower in the Midwest (aOR 0.53, 95% CI 0.43‐0.65) and South (aOR 0.68, 95% CI 0.58‐0.81) compared to the Northeast [Table 1]. Rates of FRI increased over time for non‐Hispanic Black, non‐Hispanic White, and Hispanic patients, as well as for those with public insurance. Rates of FRI also increased over time at urban teaching hospitals, and in all regions except the Midwest (p< 0.01) [Figure 1].

**Conclusion**: There are disparities in firearm related injuries by geographic location and payor status in the United States. Knowledge of such disparities can help to guide public policy aimed at preventing gun violence.

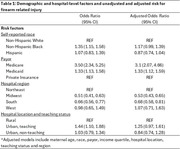

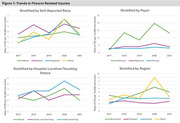

## 442 | Pregnancy Outcomes in Gestational Diabetes Based on Method of Diagnosis: GCT ≥ 200 Versus OGTT

Candace Levian^1^; Mariam Naqvi^2^; Alexandra Navarrete^1^; Mona Garcia^1^; Tania F. Esakoff^2^

*^1^Cedars‐Sinai Medical Center, Los Angeles, CA; ^2^Cedars Sinai Medical Center, Los Angeles, CA*

4:00 PM ‐ 6:00 PM

**Objective**: Gestational diabetes mellitus (GDM) can be diagnosed based on 1‐hour glucose challenge test (GCT) ≥ 200 mg/dL or ≥ 2 abnormal values on 3‐hour oral glucose tolerance testing (OGTT). We sought to investigate medication needs and pregnancy outcomes among patients with GDM by method of diagnosis.

**Study Design**: This is a retrospective cohort study of patients with GDM who delivered at a single institution from 2020‐2023. Comparisons were made between two groups based on abnormal diagnostic test: 1‐hour GCT result ≥ 200 mg/dL versus 1‐hour GCT < 200 mg/dL and abnormal OGTT. Data were obtained by chart abstraction. The primary outcome was need for insulin initiation. Secondary adverse pregnancy outcomes were also described. Comparisons were performed using χ^2^ and t‐test, with multivariable logistic regression to adjust for confounders.

**Results**: We identified 587 patients with GDM during the study period, of whom 111 (19%) were diagnosed by 1‐hour GCT ≥ 200 mg/dL and 476 (81%) had a glucose of 140‐199 mg/dL and subsequently met Carpenter‐Coustan criteria on 3‐hour OGTT. Background characteristics were similar between cohorts, though the ≥ 200 mg/dL group was more likely to be Black and was diagnosed at an earlier gestational age (Table 1). Patients with a 1‐hour GCT ≥ 200 mg/dL were more likely to require insulin during their pregnancy (53% vs 30%, *P* < 0.01) and to develop poor glycemic control (28% vs 15%, *P* < 0.01), defined as failure to meet recommended glycemic targets > 50% of the time despite medical therapy. A 1‐hour GCT ≥ 200 mg/dL was associated with higher likelihood of a large for gestational age (LGA) neonate (17% vs 8%, *P* < 0.01) and neonatal intensive care unit (NICU) admission (28% vs 18%, *P* = 0.02). After adjustment for potential confounders, these associations remained significant (Table 2).

**Conclusion**: Patients diagnosed with GDM based on 1‐hour GCT ≥ 200 mg/dL were more likely to require insulin and have poor glycemic control. LGA and NICU admission were more frequent in this population compared to those diagnosed with abnormal 3‐hour OGTT.

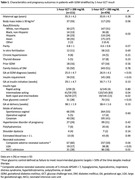

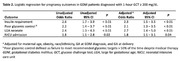

## 443 | Pregnancy Outcomes in Gestational Diabetes Mellitus: Ethnic‐Specific Differences Among a Diverse Asian‐American Population

Candace Levian^1^; Mariam Naqvi^2^; Alexandra Navarrete^1^; Mona Garcia^1^; Tania F. Esakoff^2^

*^1^Cedars‐Sinai Medical Center, Los Angeles, CA; ^2^Cedars Sinai Medical Center, Los Angeles, CA*

4:00 PM ‐ 6:00 PM

**Objective**: The prevalence of gestational diabetes mellitus (GDM) is higher in Asian populations. Comparative data on pregnancy outcomes among specific ethnic groups within Asian cohorts is lacking. Our objective was to study Asian patients with GDM and describe perinatal outcomes by country of origin.

**Study Design**: Retrospective cohort study of Asian patients with GDM stratified by region of origin (East: China, Korea, Japan, Taiwan; Southeast: Philippines, Thailand, Vietnam; South: India) who delivered at a single institution from 2020‐2023. Classification of race/ethnicity was self‐reported. Data were obtained through chart abstraction. Comparisons were performed using χ^2^ and t‐test. Multivariable logistic regression was then performed to adjust for potential confounders.

**Results**: We identified 667 patients with GDM during the study period, of whom 274 (41%) identified as Asian (36% East, 32% Southeast, 32% South). Specific ethnic groups within each region are shown in the Figure. Southeast and South Asian patients were more likely to be obese compared to those from Eastern regions (27% and 26% vs 11%, *P* = 0.01) (Table). South Asian patients were more likely to require insulin for blood glucose management and to develop poor glycemic control, defined as failure to meet recommended glycemic targets > 50% of the time despite medical therapy. Southeast Asian patients were more likely to require rapid‐acting insulin and to develop a hypertensive disorder of pregnancy. Other maternal and neonatal outcomes were similar between groups. After adjustment for obesity, Southeast and South Asian patients remained more likely to require insulin compared to East Asian patients (aOR 3.0, CI 1.5‐5.8 and aOR 2.0, CI 1.1‐4). Poor glycemic control remained associated with South Asian descent compared to East Asian after adjustment for obesity (aOR 2.5, CI 1.1‐5.9).

**Conclusion**: There are differences in glycemic management between Asian ethnic groups. Though Asian race is a known risk factor for GDM, patients who identify as Asian represent a heterogeneous group with diverse backgrounds and different clinical outcomes.

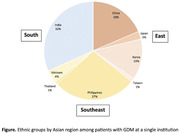

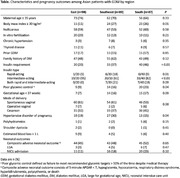

## 444 | The Impact of Senate Bill 8 on LARC and Permanent Contraception Trends in Texas

CeCe Cheng^1^; Maria I. Smereka^2^; Belem Suarez^3^; Paula A. Jaimes^3^; Carolina Vivas‐Valencia^3^; Alexander Testa^4^; John J. Byrne^5^

*^1^University of Washington, Seattle, WA; ^2^University of Texas Health Science Center at San Antonio, San Antonio, TX; ^3^The University of Texas at San Antonio, San Antonio, TX; ^4^School of Public Health, University of Texas Health Science Center at Houston, Houston, TX; ^5^University of Texas Health San Antonio, San Antonio, TX*

4:00 PM ‐ 6:00 PM

**Objective**: The goal of this study was to determine the impact of Senate Bill 8 (SB8) on trends in long‐acting reversible contraception (LARC) and permanent sterilization (PS) procedures in the state of Texas.

**Study Design**: We conducted a retrospective cohort study utilizing the Texas Outpatient Public Use Data to characterize the relationship between the Texas “Heartbeat Act” (SB8) and trends in LARC/PS procedures before (1/2021‐6/2021) and after (10/2021‐3/2022) its passage in 9/2021. We excluded data from 7/2021‐9/2021 which included the passage of SB8 and treated it as a washout period. Inclusion criteria were patients aged 15 to 54 who underwent LARC placement or female PS for contraceptive purposes. We excluded patients who underwent procedures for presumably non‐contraceptive purposes (ie. ectopic pregnancy requiring salpingectomy). ICD‐10 diagnostic and procedure codes were used. We used patients’ home zip codes and the Rural and Urban commuting area codes to categorize geographic area.

**Results**: Of the 26,576 patients who met inclusion criteria during the study period, 14,006 (52.7%) and 12,570 (47.3%) received LARC/PS in the 6 months prior to and after passage of SB8, respectively. A decrease was observed in LARC use after SB8 (12.9 v 11.8%, p = 0.0048), while there was a significant increase in PS procedures (87.1 v 88.2%, p = 0.0027). A significant increase was noted in LARC/PS after SB8 in Hispanic patients compared to other racial/ethnic groups (31.9 v 33.2%, p< 0.0001). There were increases in patients ages < 20 (3.2 v 3.7%, p = 0.021), 30‐39 (33.9 v 36.7%, p< 0.0001), and a decrease in those ≥50 (14.4 v 10.0%, p< 0.0001) post‐SB8. More patients with Medicaid (20.7 v 21.8%, p = 0.03) and commercial insurance (40.6 v 42.4%, p = 0.003) received LARC/PS after SB8. The rate of rural LARC/PS users increased after SB8 (15.2 v 17.9%, p≤ 0.0001) compared to urban dwellers (84.6 v 82.1%, p≤ 0.0001).

**Conclusion**: The significant increase in PS use compared to LARC after SB8 may indicate the desire for more reliable forms of contraception in areas where access to reproductive healthcare is at risk.

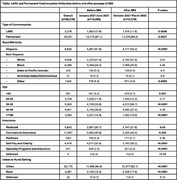

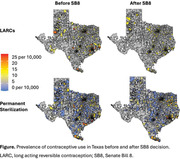

## 445 | Regional Differences in LARC and Permanent Contraception procedures in Texas after the Dobbs Decision

CeCe Cheng^1^; Maria I. Smereka^2^; Belem Suarez^3^; Paula A. Jaimes^3^; Carolina Vivas‐Valencia^3^; Alexander Testa^4^; John J. Byrne^5^

*^1^University of Washington, Seattle, WA; ^2^University of Texas Health Science Center at San Antonio, San Antonio, TX; ^3^The University of Texas at San Antonio, San Antonio, TX; ^4^School of Public Health, University of Texas Health Science Center at Houston, Houston, TX; ^5^University of Texas Health San Antonio, San Antonio, TX*

4:00 PM ‐ 6:00 PM

**Objective**: The aim of this study is to determine if there are differences in long‐acting reversible contraception (LARC) and permanent contraception (PS) procedures in Texas before and after the Dobbs v Jackson Women's Health Organization (Dobbs) decision.

**Study Design**: We conducted a retrospective cohort study utilizing the Texas Outpatient Public Use Data to determine rates of LARC/PS procedure before (1/2022 to 6/2022) and after (7/2022 to 12/2022) the Dobbs decision in June of 2021. Inclusion criteria were patients aged 15 to 54 who underwent LARC placement or female PS for contraceptive purposes. We excluded patients who underwent procedures for presumably non‐contraceptive purposes(ie. ectopic pregnancy requiring salpingectomy). ICD‐10 diagnostic and procedure codes were used. We used patients’ home zip codes and the Rural and Urban commuting area codes to categorize geographic setting.

**Results**: Of the 13,544 patients who met inclusion criteria during the study period, 6675 (49.3%) and 6869 (50.7%) received LARC/PS in the 6 months prior to and after passage of the Dobbs decision, respectively. Although there were no differences in overall rates of LARC/PS placement after passage of Dobbs, we noted a significant increase in LARC/PS procedures in patients from rural areas (17.9 v 20%, p = 0.0017) and a significant decrease in patients from urban areas (82 v 79.9%, p = 0.0013). Apart from a decrease in LARC/PS utilization after passage of Dobbs in the Hispanic population (32.7 v 31%, p = 0.03), there were no significant racial/ethnic differences in LARC/PS utilization after the passage of Dobbs. Similarly, there were no changes in LARC/PS rates across different age groups except for a decrease in the ≥50 age group (14.3 v 11.8%, p< 0.0001). There were no differences in LARC/PS rates based on insurance type.  

**Conclusion**: The slight decline in LARC/PS procedures for patients living in urban areas and of Hispanic ethnicity in Texas following the Dobbs decision may indicate emerging healthcare disparities related to changes in reproductive health policy.

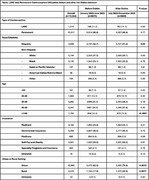

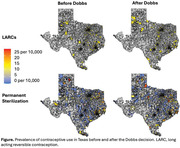

## 446 | Strict Blood Pressure Control and Risk of Postpartum Readmission

Celeste A. Green; Brooklynn Earls; Haleh Sangi‐Haghpeykar; Dominique Williams; April D. Adams

*Baylor College of Medicine, Houston, TX*

4:00 PM ‐ 6:00 PM

**Objective**: Hypertensive disorders of pregnancy (HDP) comprise approximately 10% of postpartum readmissions. Strict discharge criteria for the delivery encounter are a suggested practice for reduction in HDP‐related readmission. We evaluated whether implementation of discharge criteria requiring BP < 150/100 for at least 24 hours reduced the likelihood of postpartum readmission for HDP.

**Study Design**: Analysis included live‐ and stillbirths >/ = 20 weeks’ at a single site from 2019‐2022. Readmissions included inpatient stays with any hypertension‐related primary diagnosis code 1‐42 days after discharge. Patients discharged pre‐implementation of strict BP discharge criteria, (1/1/19‐1/31/22) were compared to those discharged post‐implementation (2/1/22‐12/31/22).  Race and ethnicity were excluded from regression models to avoid conflating the social construct of race with biological contributors to a clinical outcome.

**Results**: We reviewed 23,734 deliveries and 358 readmissions. HDP‐related readmission rates were 1.4% (N = 259) pre‐implementation of strict BP discharge criteria, and 2.1% (N = 99) post‐implementation, p< .0003. Our delivery cohort was racially and ethnically more diverse than the United States’ population, though EMR‐reported Black patients were readmitted disproportionately more than others (Tables). The most common readmission diagnosis was severe preeclampsia. Post‐implementation of strict BP discharge criteria, a higher proportion of patients (N = 307, 6.4%) also presented to triage for HDP issues than pre‐implementation (N = 1068, 5.7%), p = .0003. The odds ratio (OR) for HDP readmission post‐2/1/22 was 1.53 (CI 1.2‐1.9, p = .0004). After adjusting for confounders, the OR was 1.48 (CI 1.13‐1.92, p = .004).

**Conclusion**: HDP readmission risk was higher after implementing strict BP discharge criteria. This may represent more severe disease exacerbations, and strict BP discharge criteria may mitigate risk of unnecessary readmissions. Close outpatient follow‐up, remote BP monitoring, and patient education on HDP exacerbation may encourage appropriate presentations to care and prevent out‐of‐hospital mortality.

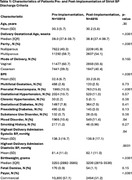

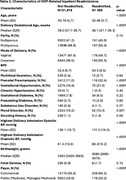

## 447 | Timing of Delivery of Preterm Membrane Rupture at 34 Weeks and Beyond: a Cost‐Effectiveness Analysis

Celine Huynh^1^; Sarah K. Dzubay^2^; Megha Arora^2^; Jacqueline M. Powell^3^; Aaron B. Caughey^2^

*^1^Oregon Health and Science University, Portland, OR; ^2^Oregon Health & Science University, Portland, OR; ^3^University of Wisconsin School of Medicine and Public Health, Madison, WI*

4:00 PM ‐ 6:00 PM

**Objective**: To examine outcomes and the cost‐effectiveness of immediate delivery at 34 weeks’ gestation versus expectant management to 35, 36, and 37 weeks in the setting of preterm premature rupture of membranes (PPROM).

**Study Design**: A decision‐analytic model was built using TreeAge software to compare outcomes of immediate delivery versus expectant management to 35, 36, and 37 weeks in a theoretical cohort of 35,913 pregnant people with PPROM at 34 weeks. Clinical outcomes included stillbirth, neonatal death, neurodevelopmental disorder, maternal mortality, healthy neonate, maternal sepsis leading to ICU admission, neonatal sepsis, NICU admission, costs and quality adjusted life years (QALYs). Probabilities, costs, and utilities were derived from literature.

**Results**: In our cohort, expectant management resulted in fewer neonatal deaths, neurodevelopmental disorders, and NICU admissions, in addition to increasing the number of healthy neonates delivered across all expectant management weeks (Table 1). Conversely, expectant management was found to increase the number of cases of stillbirth, maternal mortality, maternal sepsis, and neonatal sepsis. Each increasing week of expectant management yielded additional QALYs but was associated with increased costs. In this comparison, the optimal outcome and most cost‐effective was expectant management to 37 weeks. One‐way sensitivity analysis for the cost of antepartum care revealed that when the cost of antepartum admission exceeded $54,267 then management up to 36 weeks became cost‐effective as compared to waiting until 37 weeks. One‐way sensitivity analysis for the cost of NICU stay demonstrated that at 16 times the NICU cost, expectant management to 36 weeks becomes dominated by 37 weeks.

**Conclusion**: Management of PPROM at 34‐36 weeks’ gestation remains controversial, with guidelines incorporating both immediate delivery and expectant management. The current model suggests that ongoing expectant management to 37 weeks may improve outcomes and be a cost‐effective strategy.

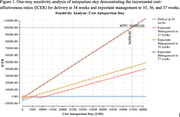

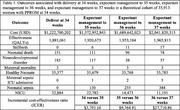

## 448 | Safe Reduction of NTSV Cesarean Delivery Rates using Multidisciplinary Quality Improvement Efforts

Chandler M. McGee^1^; Celeste A. Green^1^; Lauren Shubert^2^; Adewunmi Babalola^2^; Audra Timmins^2^; Stephanie Gonzales‐Hughes^2^; Sharon Burks^2^; Khanh Nguyen^2^; Erin Gonzales^2^; Sheena Glover^2^; Tara Barrick^2^; Courtney Thompson^2^; Grace Achim^2^; Matt Carroll^2^; Christina Davidson^2^

*^1^Baylor College of Medicine, Houston, TX; ^2^Texas Children's Pavilion for Women, Houston, TX*

4:00 PM ‐ 6:00 PM

**Objective**: The rate of cesarean deliveries (CD) in nulliparous, term, singleton, vertex (NTSV) patients at our hospital has been ∼30% since we began tracking this measure in 2016. Our aim was to evaluate the impact of quality improvement (QI) initiatives on this metric.

**Study Design**: Our level IV urban academic center formed a multidisciplinary workgroup in 4/2023. We reviewed CD rates stratified by indication, physician practice, race and ethnicity, which were regularly presented to the department and practice leaders. Nurses audited oxytocin management for adherence to hospital protocol and introduced non‐pharmacologic options to help patients cope with labor pain. Retrospective chart review of all NTSV CD from 5‐10/2023 determined if the indication for CD met ACOG criteria for failed induction of labor (FIOL), active phase arrest (APA), second stage arrest (SSA), and prelabor CD. Results were presented at departmental meetings and each physician was sent a scorecard with their total NTSV CD and adherence to ACOG criteria.

**Results**: The NTSV CD rate decreased in concordance with hospital QI efforts, to a nadir of 27.2% in the 2024 calendar year to date (Fig 1). Severe newborn complications (SNC) rate was similar before and after QI efforts (0.44% vs 0.61%, p = 0.13). Among all 298 NTSV CD, 13.8% did not meet ACOG criteria. FIOL was the most common indication (23%) that did not meet criteria across all racial and ethnic groups. Prelabor CD not meeting ACOG criteria were highest for White and Hispanic patients, driven by CD for estimated fetal weight lower than ACOG thresholds.

**Conclusion**: Our hospital NTSV CD rate decreased without an increase in SNC rates. Our chart audit allowed us to identify an opportunity to target FIOL and prelabor CD as future interventions. Sharing disaggregated data coupled with detailed chart review to identify hospital‐specific drivers of non‐medically indicated CD may be strategies to safely reduce NTSV CD rate and drive quality improvement initiatives.

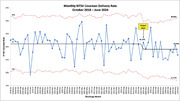

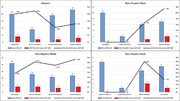

## 449 | is Severity of Congenital Cardiac Disease Associated with Increased Perinatal Mood Symptoms

Christina Paidas Teefey^1^; Juliana S. Gebb^2^; Alexandria Budney^3^; Nahla Khalek^1^; Shelly Soni^1^; Desiree Fiorentino^1^; Joanna C.M. Cole^3^; Julie S. Moldenhauer^1^; Jack Rychik^3^

*^1^Richard D. Wood Jr. Center for Fetal Diagnosis and Treatment at CHOP, Philadelphia, PA; ^2^Richard D. Wood, Jr Center for Fetal Diagnosis and Treatment, Children's Hospital of Philadelphia, Philadelphia, PA; ^3^Children's Hospital of Philadelphia, Philadelphia, PA*

4:00 PM ‐ 6:00 PM

**Objective**: To assess whether fetal cardiovascular disease severity is associated with perinatal mood symptoms in mothers with prenatal diagnosis of congenital heart disease (CHD). 

**Study Design**: Expectant mothers completed validated screens for depressive, anxiety and traumatic symptoms following diagnosis of CHD (Time 1) and immediately postpartum (Time 2). Postpartum Depression Screening Scale (PDSS) was used to identify symptoms of depressive risk (≥ 60 and ≥ 21) and Impact of Events Scale (IES‐R) was administered to determine traumatic risk (≥ 33) in a cohort of patients at a single fetal therapy center from December 1, 2012 through July 1, 2017. A fetal cardiologist reviewed echocardiographic imaging blinded to clinical course and assigned a Fetal Cardiovascular Disease Severity Score (FCDSS score severity range:1, lowest thru 7, highest) based on previously validated scale (Davey et al., 2014). Demographics were collected including mental health history confirmed by a licensed perinatal psychologist. Statistical analysis was performed using Fisher's exact test and chi squared test for categorical variables and Mann‐Whitney U test for continuous variables.

**Results**: Depressive risk was 47% (n = 177/380) during pregnancy and 34% (n = 129/380) postpartum. Anxiety and traumatic stress risk was 14% (n = 52/380) during pregnancy and 4.7% (n = 18/380) postpartum. Increasing FCDSS did not corelate with clinical depressive, anxiety or traumatic stress risk during pregnancy or postpartum (Table 1). While FCDSS was not associated with increased risk for mood symptoms, patients with a FCDSS ≥ 5 (n = 197), received more psychosocial support during pregnancy (Table 2). History of maternal mental health diagnosis strongly correlated with increased depressive risk in pregnancy (67% vs 43%, p = 0.001) and postpartum (50% vs 31%, p = 0.009).

**Conclusion**: Severity of fetal cardiovascular disease is not associated with additional depressive, anxiety and traumatic risk. Pre‐existing maternal mental health diagnosis is strongly associated with perinatal mood symptoms emphasizing the importance of perinatal psychosocial support.

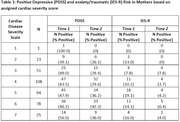

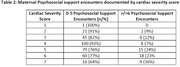

## 450 | Refractory Hypotension with Combined Spinal Epidural and Prophylactic Phenylephrine Infusion During Scheduled Cesarean Delivery

Kristina Fin^1^; Christine E. Henricks^1^; Elise A. Rosenthal^2^; Jessica E. Pruszynski^3^; Jacqueline Galvan^1^; David B. Nelson^1^; Elaine L. Duryea^1^

*^1^University of Texas Southwestern Medical Center, Dallas, TX; ^2^NYU Langone Health, Brooklyn, NY; ^3^University of Texas Southwestern, Dallas, TX*

4:00 PM ‐ 6:00 PM

**Objective**: Prophylactic phenylephrine infusion is commonly used to manage regional anesthetic‐induced hypotension during cesarean deliveries, aiming to stabilize maternal blood pressure and maintain uteroplacental perfusion to prevent fetal and maternal effects. This study evaluates the incidence of refractory hypotension with combined spinal epidural (CSE) despite the use of prophylactic phenylephrine infusion. 

**Study Design**: This retrospective cohort study included all scheduled cesarean deliveries (CD) at term from August 2023 to September 2023, excluding patients with acute indications for CD. All patients received CSE for neuraxial anesthesia. Hypotension was defined as a systolic BP < 90 mmHg or a diastolic BP < 60 mmHg with a 20% decrease from baseline BP. Patients with hypotension post‐CSE were compared to those without hypotension. Data on additional vasopressor requirements and neonatal outcomes were collected. Statistical analyses performed included chi‐squared, Fisher's exact, and Kruskal‐Wallis tests.  

**Results**: Among 170 patients undergoing scheduled cesarean delivery with CSE, 60% experienced hypotension despite prophylactic phenylephrine. Fetal heart rate deceleration occurred in 13.7% of hypotensive patients versus 5.9% of non‐hypotensive patients (p = 0.09). Median umbilical cord pH was 7.29 [7.25, 7.31] in hypotensive patients and 7.3 [7.27, 7.33] in non‐hypotensive patients (p = 0.03). Neonatal ICU admission rates were similar between groups. Rescue vasopressors were used in 75.5% of hypotensive cases and 32% of non‐hypotensive cases (p< 0.01). 

**Conclusion**: The study highlights that while prophylactic phenylephrine infusion is used to prevent hypotension during cesarean deliveries, 60% of patients still experience significant hypotension, requiring additional interventions. Further research is necessary to understand risk factors and interventions for refractory hypotension during combined spinal epidural anesthesia in scheduled cesarean deliveries to optimize maternal and neonatal outcomes. 

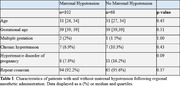

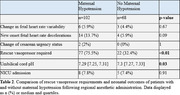

## 451 | Effectiveness of Intravenous Ferric Carboxymaltose for Severe Anemia in the Late Third Trimester

Christine E. Henricks; Rachel C. Schell; Catherine Y. Spong; Elaine L. Duryea

*University of Texas Southwestern Medical Center, Dallas, TX*

4:00 PM ‐ 6:00 PM

**Objective**: Iron deficiency anemia is associated with adverse pregnancy outcomes. Prior studies have shown reduced morbidity with intravenous (IV) iron therapy, but the optimal timing of administration is unknown. The objective of this study was to evaluate whether administration timing of IV ferric carboxymaltose in the late third trimester impacts maternal hematologic indices and outcomes. 

**Study Design**: A retrospective cohort study was conducted from October 2020 to May 2024 of IV ferric carboxymaltose for the treatment of severe anemia in the third trimester, defined by a hemoglobin < 8 g/dL or hematocrit < 25%, plus iron < 40 mcg/dL or ferritin < 15 ng/mL. Eligible patients received two infusions one week apart, regardless of gestational age at diagnosis. Patients beginning treatment early, defined as < 36 weeks gestation (WGA), were compared to those with late treatment starting ≥ 36 WGA. Mann‐Whitney U and Chi‐squared tests were used for statistical analysis. 

**Results**: Of 129 patients, 101 received IV iron < 36 weeks (early) and 28 ≥ 36 weeks (late). Hemoglobin at diagnosis of severe anemia was not different, median 7.3 [7.0,7.7] g/dL in early cohort and 7.6 [7,7.8] g/dL in the late cohort (p = 0.121). The median rise in hemoglobin between diagnosis and delivery was 3.8 [2.7, 5.2] g/dL for the entire population. There was no difference in gestational age at delivery between the cohorts. Importantly, hemoglobin levels at delivery were not different between the early (11.2 [10.5,12.0] g/dL) and late (10.6 [9.6,11.2] g/dL) cohorts (p = 0.251). The incidence of peripartum transfusion was not different, 5.0% in the early cohort and 3.6% in the late cohort (p = 0.759). 

**Conclusion**: Patients receiving IV ferric carboxymaltose for severe anemia ≥36 WGA demonstrated similar improvements in hemoglobin levels compared to those receiving treatment earlier in gestation and were no more likely to require transfusion at delivery. IV ferric carboxymaltose should not be withheld in the late third trimester based on perceived futility with advancing gestational age.  

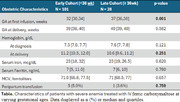

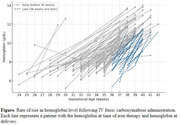

## 452 | Accuracy of Ultrasound‐Derived Estimated Fetal Weight in Peri‐Viable Births

Colleen P. Judge‐Golden^1^; Sally Kuehn^2^; Sarah Ellestad^1^; Sarah K. Dotters‐Katz^2^; Amanda M. Craig^1^

*^1^Duke University Hospital, Durham, NC; ^2^Duke University School of Medicine, Durham, NC*

4:00 PM ‐ 6:00 PM

**Objective**: As neonatal resuscitation is offered at increasing extremes of prematurity, ultrasound‐derived estimated fetal weight (EFW) is a crucial data point in patient counseling. We aimed to evaluate accuracy of EFW compared to neonatal birthweight (BW) in peri‐viable births and identify factors associated with accuracy.

**Study Design**: Medical records were abstracted for all liveborn, non‐anomalous neonates born at 22w0d‐24w6d at a single tertiary institution between 1/2013‐5/2024, with ultrasound‐derived EFW within 7 days of delivery. Primary outcome was absolute percent difference (accuracy) between EFW (Hadlock) and BW. Linear regression and Kruskall‐Wallis tests were used to assess associations of accuracy with imaging variables, maternal and pregnancy characteristics. Adjusted linear regression was performed including maternal BMI at EFW (an *a priori *plausible association) and variables associated with accuracy in univariate analyses at p< 0.1.

**Results**: 141 neonates from 129 pregnancies were included. Median gestational age at delivery was 23w4d, with median BW 560g. Most deliveries (83%) were sequelae of cervical insufficiency, preterm labor, or preterm prelabor rupture of membranes; 17% were iatrogenic for maternal or fetal indications. Median time from EFW to delivery was 2 days. A certified sonographer performed 85% of ultrasounds. EFW was highly correlated with BW (p< 0.001, R^2^ = 0.72); 54% of EFWs underestimated BW. Median absolute percent difference was 7.1% [IQR 2.9%,12.2%]. In unadjusted analyses, increasing BW (p = 0.04) and multiple gestations (p = 0.002) were associated with improved accuracy; oligohydramnios was associated with decreased accuracy (p = 0.04). Maternal BMI, sonographer level, fetal presentation and EFW timing were not associated with accuracy. Adjusting for BMI, oligohydramios and multiples, BW remained inversely associated with EFW accuracy (coefficient ‐0.016, p = 0.013).

**Conclusion**: Ultrasound‐derived EFW was an excellent predictor of BW among peri‐viable deliveries at our institution. Accuracy declined with lower BW, which may be considered in antenatal counseling.

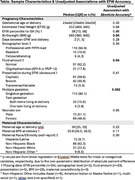

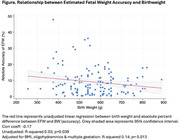

## 453 | Association Between Instrumental Vaginal Delivery and the Risk of Postpartum Depression

CYRIELLE PRAT BALAGNA^1^; Marianne Jacques^2^; Marie Viaud^3^; Sarah Tebeka^4^; Aurélien Seco^5^; Nathalie Lelong^3^; Camille Le Ray^3^

*^1^INSERM UMR 1153, Obstetrical, Perinatal and Pediatric Epidemiology Research Team (Epopé), Center for Epidemiology and Statistics, FHU PREMA, Université de Paris, PARIS, Ile‐de‐France; ^2^Obstetrical, Perinatal and Pediatric Epidemiology Research Team (EPOPé) ‐ Centre for Research in Epidemiology and Statistics (CRESS), Université Paris Cité, Inserm, Paris, Ile‐de‐France; ^3^INSERM UMR 1153, Obstetrical, Perinatal and Pediatric Epidemiology Research Team (Epopé), Center for Epidemiology and Statistics, FHU PREMA, Université de Paris, Paris, Ile‐de‐France; ^4^Department of Psychiatry, AP‐HP, Louis Mourier Hospital, Colombes, Ile‐de‐France; ^5^Université Paris Cité, Inserm, Centre for Research in Epidemiology and StatisticS (CRESS), Obstetrical Perinatal and Pediatric Epidemiology Research Team (EPOPé),, Paris, Ile‐de‐France*

4:00 PM ‐ 6:00 PM

**Objective**: Postpartum depression(PPD) affects 10‐20% of women and previous studies have identified instrumental vaginal birth(VB) as a risk factor. Our study aimed to test this association, in full‐term pregnancies with a trial of vaginal delivery, and to explore mediating factors.

**Study Design**: We used data from the observational nationwide population‐based study ENP 2021, including women who completed the Edinburgh Postnatal Depression Scale(EPDS) at 2‐month. Multiple pregnancies, non‐cephalic presentations, preterm births and planned cesareans were excluded. An EPDS score ≥ 13 was used as a validated proxy for PPD. Mode of delivery (spontaneous VB/instrumental VB/cesarean), type and indication of the instrument were collected. The association between instrumental VB and PPD was studied using univariate and multivariate analyses and weighted to account for 2‐month attrition. Mediation analyses were conducted after identification of intermediate factors: maternal composite criterion (postpartum hemorrhage+/‐obstetric anal sphincter injury), episiotomy, neonatal composite criterion (Apgar score ≤ 7+/‐neonatal transfer), pain and disrespectful care during delivery. Two sensitivity analyses were performed among women at low psychiatric risk and using an EPDS score ≥ 11.

**Results**: The study population included 6084 women: 4473(73.5%) had spontaneous VB, 915(15.0%) instrumental VB and 696 (11.5%) cesareans. PPD prevalence was 16.3%[15.3‐17.3] reaching 19.3% for instrumental VB (aOR 1.35[1.10‐1.66]) compared with 15.6% for spontaneous VB (reference) and 16.7% for cesareans (aOR 1.05[0.83‐1.33]). Fetal heart rate abnormalities indication was associated with higher PPD prevalence. The indirect effect mediated by disrespectful care during delivery explained 18% of the association between instrumental VB and PPD (aOR of indirect effect: 1.04[1.01‐1.07]). We found no significant mediation effect for other factors. Sensitivity analyses found similar results.

**Conclusion**: The prevalence of PPD was significantly higher with instrumental VB, an association partly mediated by disrespectful care during delivery.

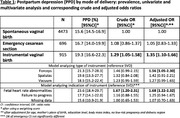

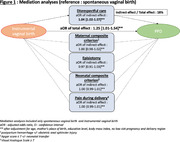

## 454 | The Association between Post‐Traumatic Stress and Perineal Pain in Patients with Birth Injury

Dana R. Canfield^1^; Emmanuel Elijah^2^; Omar Mesina^3^; Rachel L. Wiley^2^; Aava Saffarnia^2^; Alexandra Nutaitis^2^; Lindsey A. Burnett^4^

*^1^UC San Diego Health, San Diego, CA; ^2^University of California, San Diego, San Diego, CA; ^3^University of California San Diego Health, San Diego, CA; ^4^UC San Diego Medical Center, La Jolla, CA*

4:00 PM ‐ 6:00 PM

**Objective**: While the impact of emotional distress on perceived pain has been shown in several studies, few have focused on patients with birth injury. Our goal was to determine whether post‐traumatic stress is associated with a higher incidence of reported pain in birth‐injured patients.

**Study Design**: This was a retrospective cohort study of patients seen in a urogynecology clinic specializing in the management of birth injury. Patients completed surveys addressing physical and emotional wellbeing which included a Peritraumatic Stress Inventory (PDI) to assess for childbirth‐related trauma symptoms. Demographic and delivery variables were compared between patients who had a PDI score of >14, a cut‐off associated with a greater risk of developing post‐traumatic stress disorder. Pain was assessed as a binary variable based on response to the question “are you having perineal/vaginal pain?”. Demographic and delivery factors associated with high PDI were added to a multivariable logistic regression model of pain (Table 1). Edinburgh Depression Scale (EPDS) score was excluded given concern for effect mediation.

**Results**: 48 patients completed the PDI and were included in our analysis. Patients with low PDI were assessed at an average of 11.34 (SD 4.53) weeks postpartum and patients with high PDI at 10.33 weeks (SD 6.24). Incidence of stool and gas incontinence was significantly greater in the high PDI group. While antepartum EPDS scores did not significantly differ, postpartum scores were greater in high PDI group. After adjusting for stool and gas incontinence, patients were more likely to report pain (OR 1.22; 95% CI ‐0.26, 0.47) but this difference was not statistically significant. However, elevated PDI was associated with leakage of stool in our adjusted model (OR 1.75; 95% CI 1.14, 2.17).

**Conclusion**: We found that psychologically traumatic birth was positively but insignificantly related to reported pain and significantly associated with increased stool incontinence. While further studies are needed, our results point towards a relationship between an emotionally traumatic birth and physical recovery.

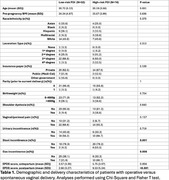

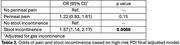

## 455 | Adverse Childhood Experiences and Perceived Control Over Birth

Dana R. Canfield^1^; Cynthia Gyamfi‐Bannerman^2^; Emmanuel Elijah^2^; Karoline Gutierrez^3^; Minhazur R. Sarker^2^; Rachel L. Wiley^2^; Elizabeth Nicole Teal^2^; Marni B. Jacobs^4^

*^1^UC San Diego Health, San Diego, CA; ^2^University of California, San Diego, San Diego, CA; ^3^University of Miami, Miami, FL; ^4^University of California, San Diego Health, San Diego, CA*

4:00 PM ‐ 6:00 PM

**Objective**: Adverse childhood experiences (ACEs) have been shown to impact adult health but have not been studied in connection with perceived control over labor, termed labor agentry. We aimed to study the impact of ACEs on labor agentry as recorded on the Labor Agentry Scale (LAS), a 29‐item Likert‐scale response survey in which higher score corresponds with greater agentry.

**Study Design**: In this prospective cohort study, patients were enrolled from high‐risk obstetric clinics at a single institution from 27‐34 weeks gestation. Participants were asked to complete mental health surveys on enrollment including ACE‐Q, a 10‐item measure to assess adverse experiences prior to age 18. At the time of birth participants completed the LAS. Patients were divided into three conventional ACE categories: low‐risk (ACE = 0), moderate‐risk (ACE = 1‐3), and high‐risk (ACE = 4+). LAS was analyzed as a continuous variable. Demographic factors independently associated with ACE scores were included in a multivariable linear regression model of labor agentry score, except history of a mental health condition given concern for effect mediation (Tables 1 and 2).

**Results**: Of 59 patients that completed the ACE‐Q and LAS, 17 (28.8%) were low risk, 29 (49.2%) moderate risk, and 13 (22.0%) high risk. Participants in the high‐risk ACE category tended to be younger (Table 1, p = 0.005). Mean labor agentry was 159.9, 166.6, and 162.7 for low, moderate, and high‐risk ACE, respectively, and did not differ significantly by ACE group (p = 0.74).  Compared to the low‐risk ACE group, moderate and high‐risk ACE scores were associated with the higher LAS in our adjusted model, but this was not statistically significant.

**Conclusion**: We did not find significant differences in agentry by ACE score. While additional studies are needed to confirm these findings, is plausible that patients with trauma history are more accustomed to a lack of agentry and this alters their expectations and perceptions during childbirth, attenuating differences that would otherwise be found between groups.

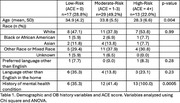

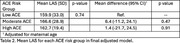

## 456 | Comparison of Prophylactic Antibiotics Regimens in Pregnancies Complicated by Cervical Insufficiency Requiring Cervical Cerclage

Daniel J. Martingano^1^; Marwah Al‐Dulaimi^2^; Sandra Kumwong^3^; Andrea Ouyang^4^; Lauren Cue^5^; Ashley Nguyen^2^; Francis X. Martingano^6^; Shailini Singh^7^; Alexander Ulfers^8^; Mark Rebolos^2^; Kristin Cohen^9^; Donald Morrish^2^; Iffath A. Hoskins^10^

*^1^St. John's Episcopal Hospital‐South Shore and William Carey University College of Osteopathic Medicine, Far Rockaway, NY; ^2^St. John's Episcopal Hospital‐South Shore, Far Rockaway, NY; ^3^Touro College of Osteopathic Medicine‐Harlem Campus, New York, NY; ^4^William Carey University, Hattiesburg, MS; ^5^Rutgers University and the Jersey City Medical Center, Jersey City, NJ; ^6^NYU Grossman School of Medicine ‐ NYU Brooklyn, New York, NY; ^7^AtlantiCare Regional Medical Center, Pamona, NJ; ^8^Walter Reed National Military Medical Center, Bethesda, MD; ^9^RWJBarnabas Health ‐ Trinitas Regional Medical Center, Elizabeth, NJ; ^10^Albert Einstein College of Medicine ‐ Montefiore Medical Center, New York, NY*

4:00 PM ‐ 6:00 PM

**Objective**: The effect of  prophylactic antibiotics with or without tocolytic medications on gestational latency remains uncertain. This study sought to evaluate the effectiveness of adjunctive antibiotic regimens on increasing gestational latency for pregnancies complicated by cervical insufficiency requiring cervical cerclage.

**Study Design**: We conducted a multi‐center, prospective observational study from 7/2022 to 7/2024 comparing all pregnancies complicated by cervical insufficiency requiring cervical cerclage at a gestational age range of 16 0/7 to 23 6/7 weeks. History‐indicated (HC) and physical‐exam indicated (PC) cerclages were included. Antibiotic regimens used as covariates included cefazolin and/or azithromycin, and were determined based on physician preference. Each regimen received indomethacin and utilized a vaginal surgical approach. Each regimen was compared to the total patients receiving a different regimen. Patients with multiple gestations, additional tocolytic medication use, or prior vaginal progesterone use, were excluded. The primary outcomes included completed weeks gestation at time of delivery (CW), preterm and term deliveries, as respective, discrete events.

**Results**: The study included 209 patients with 87 requiring a PC and 112 requiring HC. Demographic factors were not significantly different. In the PC group, 43 (49.4%) received cefazolin, 16 (18.4%) received azithromycin, 28 (32.2%) received both. In the HC group, 78 (70.0%) received cefazolin, 6 (5.4%) received azithromycin, and 28 (25%) received both. Kaplan‐Meier survival analysis (Figure 1) noted significantly greater pregnancy latency when comparing cefazolin to azithromycin for PC (p = 0.049). No significant differences in gestational latency or rates of preterm or term delivery were noted in the HC group for any regimen or when comparing other PC regimens.

**Conclusion**: It is reasonable to administer cefazolin when performing a physical‐exam indicated cervical cerclage with a vaginal approach and may offer greater pregnancy latency, with likely no additional benefit when using azithromycin alone or in combination with cefazolin.

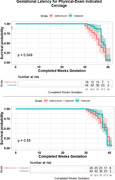

## 457 | Comparison of Antiemetic Regimens for 1st Trimester Pregnancies Complicated by Cannabinoid Hyperemesis Syndrome

Daniel J. Martingano^1^; Amanda F. Francis Oladipo^2^; Marwah Al‐Dulaimi^3^; Sandra Kumwong^4^; Andrea Ouyang^5^; Lauren Cue^6^; Ashley Nguyen^3^; Francis X. Martingano^7^; Shailini Singh^8^; Mark Rebolos^3^; Alexander Ulfers^9^; Kristin Cohen^10^; Donald Morrish^3^; Iffath A. Hoskins^11^

*^1^St. John's Episcopal Hospital‐South Shore and William Carey University College of Osteopathic Medicine, Far Rockaway, NY; ^2^Hackensack University Medical Center, Hackensack, NJ; ^3^St. John's Episcopal Hospital‐South Shore, Far Rockaway, NY; ^4^Touro College of Osteopathic Medicine‐Harlem Campus, New York, NY; ^5^William Carey University, Hattiesburg, MS; ^6^Rutgers University and the Jersey City Medical Center, Jersey City, NJ; ^7^NYU Grossman School of Medicine ‐ NYU Brooklyn, New York, NY; ^8^AtlantiCare Regional Medical Center, Pamona, NJ; ^9^Walter Reed National Military Medical Center, Bethesda, MD; ^10^RWJBarnabas Health ‐ Trinitas Regional Medical Center, Elizabeth, NJ; ^11^Albert Einstein College of Medicine ‐ Montefiore Medical Center, New York, NY*

4:00 PM ‐ 6:00 PM

**Objective**: Cannabinoid hyperemesis syndrome (CHS) is characterized by cyclic vomiting in chronic cannabis users. CHS can both exacerbate and mimic hyperemesis gravidarum (HG) in 1^st^ trimester pregnancies and is often refractory to antiemetic medications use to treat HG alone. This study sought to compare antiemetic regimens in cases of concurrent CHS in first trimester pregnancies.

**Study Design**: We conducted a multi‐center, prospective observational study from 7/2022 to 7/2024 comparing all pregnant patients with concurrent CHS at gestational age range of 8 0/7 to 13 6/7 weeks. CHS was diagnosed by Rome IV criteria for cyclical vomiting syndrome and cannabis use more than 4 times per week on average. The primary outcomes included need to switch medication and patient‐reported resolution confirmed by provider assessment, as discrete events. Patients taking overlapping medications or with allergies to any included medications were excluded. Monotherapy oral medications including metoclopramide, odansetron, cyproheptadine, diphenhydramine, and topical capsaicin were included as covariates. All patients endorsed routine hot‐water bathing to aid in resolution of symptoms. Medication choice was determined by physician preference.

**Results**: The study included 537 patients diagnosed with CHS. Baseline demographic factors were not significantly different. Patients who received metoclopramide were less likely to need to switch to an alternative medication (56.2% v. 28.3%, *p*  = 0.002) with a 29% decreased risk when adjusted for confounders (RR 0.71, 95% CI 0.43‐0.89, p = 0.002). Patients who received topical capsaicin were more likely to report improvement in appetite (51.9% v. 20.2%, p = 0.032), with a 31% increased likelihood when adjusted for confounders (RR 1.31, 95% CI 1.44‐1.93, p = 0.001). This same group, however, demonstrated the lowest compliance rate (11.9% v. 45.9%, p = 0.005).

**Conclusion**: Metoclopramide and topical capsaicin in conjunction with routine hot‐bathing are reasonable and efficacious antiemetic regimens for 1^st^ trimester pregnancies complicated by CHS.

## 458 | Predicting the need for Blood Transfusion Based on CBC at admission in Normal Vaginal Delivery

Daniel Gabbai^1^; Itamar Gilboa^1^; Anat Lavie^2^; Yariv Yogev^3^; Emmanuel Attali^1^

*^1^Lis Hospital for Women's Health, Tel Aviv Sourasky Medical Center, Tel‐Aviv, Tel Aviv; ^2^Tel Aviv Sourasky Medical Center, Tel Aviv Sourasky Medical Center, Tel Aviv; ^3^Lis Maternity Hospital, Sourasky Medical Center, Tel Aviv University, Tel Aviv Sourasky Medical Center, Tel Aviv*

4:00 PM ‐ 6:00 PM

**Objective**: The relationship between admission complete blood count (CBC) parameters, specifically the Neutrophil‐to‐Lymphocyte Ratio (NLR), and the requirement for packed red blood cell (pRBC) transfusion following vaginal delivery remains underexplored. This study aims to investigate the association between various CBC parameters, including NLR, and the need for pRBC transfusion, and to evaluate their predictive value for this outcome.

**Study Design**: A retrospective cohort study was conducted at a tertiary, university‐affiliated medical center with about 12,500 annual deliveries (2012‐2023). Women were categorized based on the need for blood transfusion, determined by severe ongoing haemorrhage, symptomatic anemia with Hb levels of 7–8 g/dL, or postpartum Hb levels < 7 g/dL. Maternal demographics, CBC characteristics at admission, and delivery outcomes were analyzed. Multivariate logistic regression identified predictors for pRBC transfusion, and a risk prediction score was developed and evaluated using the ROC curve

**Results**:  During the study period, 145,833 women delivered in our center. The CBC parameters of 37,631 women who delivered vaginally were available. Of them, 957 (2.5%) required pRBC transfusion.

Advanced maternal age, nulliparity, and conception by IVF were associated with increased pRBC transfusion. In contrast, spontaneous onset of delivery (compared to induction) was associated with a lower likelihood of transfusion.

Regarding admission CBC parameters, Hemoglobin < 10.5 g/dL (OR: 6.8, 95% CI: 5.8‐8.02, p< 0.001) and Neutrophil to Lymphocyte Ratio (NLR) > 5 (OR: 1.55, 95% CI: 1.3‐1.87, p = 0.001) were associated with an increased rate of pRBC transfusion.

A scoring model, using a cutoff of 6 [Table 1], successfully predicted blood cell transfusion with an area under the curve of 0.78 (95% CI: 0.75‐0.79, p‐value < 0.001), achieving 85% sensitivity and 77% specificity. (Figure 1]

**Conclusion**: CBC parameters, especially hemoglobin and NLR, along with maternal factors, are valuable predictors for the need of pRBC transfusion in vaginal deliveries.

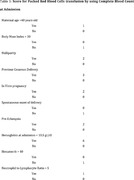

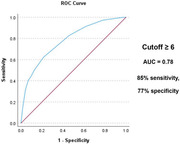

## 459 | Risk Factors for Prolonged Hospitalization following Vacuum‐Assisted Delivery

Daniel Gabbai^1^; Lee Reicher^2^; Anat Lavie^3^; Yariv Yogev^4^; Emmanuel Attali^1^

*^1^Lis Hospital for Women's Health, Tel Aviv Sourasky Medical Center, Tel‐Aviv, Tel Aviv; ^2^Weizmann Instutute Of Science, Weizmann Institute of Science, HaMerkaz; ^3^Tel Aviv Sourasky Medical Center, Tel Aviv Sourasky Medical Center, Tel Aviv; ^4^Lis Maternity Hospital, Sourasky Medical Center, Tel Aviv University, Tel Aviv Sourasky Medical Center, Tel Aviv*

4:00 PM ‐ 6:00 PM

**Objective**: We aimed to determine risk factors for prolonged hospitalization following vacuum‐assisted delivery (VAD).

**Study Design**: A retrospective cohort study, in a single, university‐affiliated tertiary medical center with approximately 12,500 deliveries annually (2012‐2022) was conducted. Standard practice in our department entails a post‐vaginal delivery hospital stay of 48 to 72 hours. Length of stay following a VAD was analyzed and parturients who underwent VAD were categorized into two groups by length of hospitalization (more and less than 7 days). Maternal and neonatal characteristics were compared. Risk factors for prolonged hospitalization were examined using univariate and multivariate logistic regression. 

**Results**: (1) Overall, 133,035 deliveries occurred during the study period. Of them, 9,440 (7.1%) were by VAD.

(2) The post‐partum hospitalization period was distributed as follows: 9134 (96.76%) women were hospitalized for up to 7 days, and 306 (3.34%) were hospitalized for more than 7 days.

(3) Using multivariate analysis: preterm delivery (delivery prior to 37 weeks) (RR 5.4 95%CI [1.6‐17], p = 0.005); preeclampsia (RR 6.0 95%CI [1.9‐19], p = 0.002), induction of labor (RR 3.4 95%CI [1.8;6.3], p< 0.001), obstetric anal sphincter injury (RR 3.2 95%CI [1.2‐8], p = 0.013), postpartum hemorrhage which required blood transfusion (RR 3.3 95%CI [1.3‐8.4], p = 0.012), and Apgar Score under 7 at minute 5 (RR 3.4 95%CI [1.4‐39], p = 0.015), were identified as independent risk factors for prolonged hospitalization [Table]

**Conclusion**: Risk factors associated with prolonged hospitalization following a VAD can be identified, with preterm delivery and Apgar score < 7 at minute 5 being the most prominent.

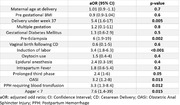

## 460 | Diagnostic Value of CFDNA Fetal Fraction in Patients with Prenatally Suspected Placenta Accreta Spectrum Disorder

Danielle L. Chirumbole^1^; Christian M. Parobek^1^; Alex Tai^1^; Haleh Sangi‐Haghpeykar^1^; Yamely H. Mendez^1^; Christina C. Reed^2^; Arthur Ladron De Guevara^1^; Keneshia Lane^1^; Claire Hoppenot^1^; Amir A. Shamshirsaz^1^; Michael A. Belfort^3^; Jessian L. Munoz^1^; Hendrik A. Lombaard^4^

*^1^Baylor College of Medicine, Houston, TX; ^2^Baylor College of Medicine at Texas Children's Pavilion for Women, Houston, TX; ^3^Texas Children's Hospital and Baylor College of Medicine, Houston, TX; ^4^Texas Children's Hospital, Houston, TX*

4:00 PM ‐ 6:00 PM

**Objective**: The pathophysiology of placenta accreta spectrum (PAS) is unknown but thought to be related to myometrial disruption at the uterine‐placental interface. Noninvasive prenatal testing (NIPT) analyzes circulating placental DNA, and it has been postulated that PAS may affect the fetal fraction (FF) of cell‐free DNA (cfDNA). The purpose of this study is to investigate the relationship between FF and PAS pathology in patients with prenatally suspected PAS.

**Study Design**: This was a case‐control study utilizing a large database of pregnancies with suspected or proven PAS delivered between 6/2012 and 7/2024 at a single academic institution. Pregnancies were excluded if FF was not reported. Other exclusion criteria were lack of suspicion for PAS on prenatal imaging, twins, fetal demise, abnormal NIPT, fetal structural anomalies, or postnatal diagnosis of a genetic/metabolic disorder. The primary outcome was mean FF in pregnancies with a final clinical diagnosis of no PAS or FIGO1‐2 (less complex) versus FIGO3A‐C (more complex). A generalized linear mixed model was used and FF was log‐transformed to achieve normality. Results were reported as mean FF ± standard error of the mean. Adjusted means were also reported after assessing for confounders.  

**Results**: Of the 468 pregnancies assessed, 165 had NIPT results reporting FF. Of these, 128 met full inclusion criteria. The mean FF in the less complex group did not differ from the more complex group (9.6% ± 0.49, n = 81 vs 10.7% ± 0.64, n = 47; p = 0.22). However, after adjusting for number of prior cesarean deliveries and maternal BMI, the mean FF in the less complex group was significantly lower than the more complex group (9.3% ± 0.48 vs 11.1% ± 0 .64, p = 0.03). Other factors including maternal age, maternal medical comorbidities, gestational age at time of NIPT, and placenta previa were not found to correlate with FF or FIGO grade.

**Conclusion**: Higher FIGO grade PAS may be associated with a higher FF in pregnancies with prenatal suspicion for PAS. Thus, NIPT may have the potential to assist with delivery preparation and prognostication in this high‐risk patient population.

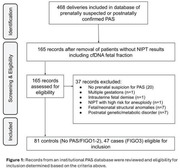

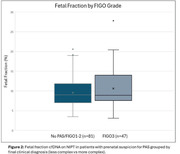

## 461 | Gestational Diabetic Placental T‐Cells Cause Hypertension and Impaired Glucose Utilization in Pregnant GDM Rat Model

Danielle Frieson; Jackson Strong; Baoying Zheng; Jean Vel; Nathan Campbell; Babbette LaMarca; Evangeline Deer

*University of Mississippi Medical Center, Jackson, MS*

4:00 PM ‐ 6:00 PM

**Objective**: Gestational diabetes mellitus (GDM) refers to glucose intolerance, insulin sensitivity and beta islet cell dysfunction during pregnancy. GDM pathogenesis is associated with hypertension, impaired placental and renal function, oxidative stress and increased circulating CD4+ T cells. Importantly, there are limited animal models available to study GDM or alternative treatment options. This study sought to determine if a possible mechanism for GDM is placental CD4+ T cell programming in the patient and to test alternative treatment options for GDM.

**Study Design**: Upon delivery, blood and placental GDM (GDM‐CD4+ T cells) and normotensive (NP+NP) placental CD4+ T cells were collected. Purified CD4+T cells were injected into pregnant nude athymic rats on gestational day (GD) 12. On GD 13, a separate cohort rats were treated with Metformin (GDM+Met; 300 mg/kg/day) or MitoTempo (GDM+Mito;1 mg/kg/d). Mean arterial pressure (MAP), circulating glucose, and glucose tolerance tests (GTT) were performed on GD 19. Mitosox Red was used to measure mtROS production in HUVECs incubated with sera from GDM, GDM women treated with Metformin (GDM+Met) or Normotensive women. A one‐way ANOVA was used for statistical analysis.

**Results**: MAP was higher in GDM‐CD4+ T cell rats (121±2, n = 8, p< 0.05) compared to GDM+MET (95±2, n = 6), GDM+Mito (95±4, n = 7), NP+NP CD4+ T cells (96±4, n = 7), NP+NP MET (99±4, n = 8), and NP nude controls (105±3 mmHg, n = 8) rats. Blood glucose was elevated in GDM‐CD4+ T cell rats (227±39 mg/dl, n = 8, p< 0.05) compared to GDM+MET (111±11 mg/dl, n = 7), GDM+Mito (97±2 mg/dl, n = 7), NP+NP CD4+ T cells (89±3 mg/dl, n = 7), NP+NP MET (96±2 mg/dl, n = 8), and NP nude controls (82±9 mg/dl, n = 8) rats. GTT was impaired in GDM CD4+ T rats, but was improved with Met and Mito treatment. Mitochondrial ROS was increased in HUVECS treated with GDM patient sera (p< 0.05) compared to GDM+Met or Normotensive patient sera.

**Conclusion**: Our findings indicate that GDM CD4+ T cells cause a diabetic phenotype suggesting a new model to investigate mechanisms and therapies.

## 462 | Outcomes in Individuals with Expectant Management of Previable PROM in Texas following SB8

Danna Ghafir^1^; Emily Fahl^1^; Nancy Ukoh^1^; Han‐Yang M. Chen^1^; Sean C. Blackwell^1^; Julie Gutierrez^2^; Irene A. Stafford^1^

*^1^McGovern Medical School at UTHealth Houston, Houston, TX; ^2^McGovern Medical School at The University of Texas Health Science Center at Houston (UTHealth), Houston, TX*

4:00 PM ‐ 6:00 PM

**Objective**: We sought to compare outcomes of pregnant individuals diagnosed with previable PROM and expectantly managed at our center before and after implementation of Texas Senate Bill 8 (SB8).

**Study Design**: This is a retrospective cohort study across three tertiary care hospitals from January 1, 2018‐March 31, 2023. ICD‐10 codes were used to identify all hospital deliveries of individuals with previable PROM. Trained research staff completed chart review. Patients with singleton or twin gestions and clinical diagnosis of PROM < 22 wks 0 days were studied, excluding those with IUFD and/or active PTL. Expectant management was defined as continuation of pregnancy (no immediate delivery) after admission and delivery for spontaneous PTL, IUFD, or development of a medical indication (infection, bleeding, or other indications). We compared clinical characteristics and outcomes of individuals with expectant management before and after the implementation of SB8 on September 1, 2021. Our primary outcome was composite adverse outcome, which included ICU admission, transfusion, and/or sepsis. Adjusted relative risk (aRR) with 95% CIs was calculated. Multivariable Poisson regression was used to adjust for confounding factors for the primary outcome.

**Results**: Over the 5‐year study period, 118 individuals met inclusion criteria (49 pre‐SB8 vs 69 post‐SB8). There were no differences in clinical characteristics pre‐ vs post‐SB8 (Table 1). There was no difference in the rate of the composite adverse outcome (26.5% vs 37.7%, aRR = 1.50, 95% CI = 0.85‐2.62), after adjusting for age, race and ethnicity, and gestational age at delivery. Time from rupture to delivery was significantly longer in the post‐SB8 cohort (median [IQR]: 3 [1‐7] days vs 6 [2‐12] day, p = 0.048). The post‐SB8 cohort demonstrated a higher rate of sepsis (12.2% vs 31.9%, aRR = 2.69, 95% CI = 1.20‐6.03).

**Conclusion**: After SB8, individuals with PROM < 22 wks had longer latency period and higher rates of sepsis compared to those before SB8. We speculate this may be due to delayed intervention by providers to wait for medical indications that qualify as “medical emergency.”

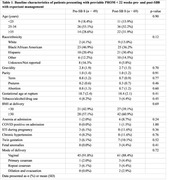

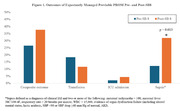

## 463 | Characteristics of Gestational Surrogate Pregnancy in a Large Integrated Healthcare Setting

Darios Getahun^1^; Fagen Xie^1^; Theresa M. Im^1^; Daniella Park^1^; Vicki Y. Chiu^1^; Yinka Oyelese^2^; Morgan R. Peltier^3^; Michael J. Fassett^4^

*^1^Kaiser Permanente Southern California, Department of Research & Evaluation, Kaiser Permanente Southern California/Pasadena, CA; ^2^Beth Israel Deaconess Medical Center/Harvard Medical School, Boston, MA; ^3^Jersey Shore University Medical Center, Department of Psychiatry and Behavioral Health, Jersey Shore University Medical Center/Neptune City, NJ; ^4^Kaiser Permanente West Los Angeles Medical Center, Department of Obstetrics & Gynecology, Kaiser Permanente West Los Angeles Medical Center/Los Angeles, CA*

4:00 PM ‐ 6:00 PM

**Objective**: To describe maternal and infant characteristics of gestational surrogate (GS) pregnancies in an integrated healthcare system with a large, diverse pregnant population.

**Study Design**: An electronic health records (EHR)‐based retrospective cohort study of pregnant members delivered at 20‐44 weeks’ gestation at Kaiser Permanente Southern California from 01/01/2008–12/31/2023 (N = 636,300) was performed. Unstructured data abstracted from EHRs were used to determine GS status via natural language processing (NLP), validated by manual chart review (positive predictive value = 96%). Hospital in‐ and outpatient physician encounters, and laboratory records were used to ascertain medical and obstetrical histories. Adjusted risk ratios (aRR) and 95% confidence intervals (CI) derived from robust Poisson regression were used to describe the magnitude of associations.

**Results**: Among pregnant members who delivered during the study period, 1,109 (1.7/1000) were GS pregnancies. Maternal age ≥ 25 years, non‐Hispanic white, multiple gestation (aRR: 10.12, 95% CI: 8.6‐11.92), and privately funded KP membership (aRR: 2.48, 95% CI: 2.12‐2.89) were more likely to be associated with gestational surrogacy. GS patients were more likely to be overweight (aRR: 1.93, 95% CI: 1.56‐2.14) and obese (aRR: 2.15, 95% CI: 1.80‐2.56). Patients with a GS pregnancy were less likely to smoke (aRR: 0.34, 95% CI: 0.17‐0.69) or drink alcohol (aRR: 0.54, 95% CI: 0.47‐0.63) during pregnancy. Children of GS were more likely to be born preterm (20‐33 weeks [aRR: 2.32, 95% CI: 1.73‐3.10], 34‐36 [aRR: 2.50, 95% CI: 2.08‐3.01] weeks of gestation), and at a birthweight < 2500g (aRR: 1.57, 95% CI: 1.26‐1.95), whereas no difference in child's sex was observed.

**Conclusion**: The findings suggest that gestational surrogates tended to be older, non‐Hispanic white, overweight or obese, and seldom smoked or consumed alcohol during pregnancy. They tended to have multiple gestation, preterm and low birthweight children, and privately funded KP membership.

## 464 | Evaluation of Maternal and Neonatal Outcomes Based on Indication for Cerclage

David M. Esterquest^1^; Rachel K. Harrison^2^; Calla Holmgren^2^

*^1^Advocate Lutheran General Hospital, Advocate Lutheran General Hospital, IL; ^2^Advocate Health Care, Advocate Health Care, IL*

4:00 PM ‐ 6:00 PM

**Objective**: Comparison of pregnancy outcomes in those undergoing ultrasound, history, and exam‐indicated cerclage placement in a large US hospital system.

**Study Design**: Retrospective cohort study of singleton pregnancies requiring cerclage at multiple hospitals in a large hospital system between 2020‐2023. Subjects were included if they had a cerclage placed during pregnancy and available maternal and neonatal outcomes. They were excluded if maternal and neonatal outcomes were not available. Patients were then separated into three groups based on cerclage indication: history (HI), ultrasound (US) and exam indicated (EI). Our primary outcome was delivery < 37 weeks. Maternal and neonatal outcomes were then compared between groups using Chi‐squared analysis, ANOVA, as well as logistic and linear regression, with a p‐value < 0.05 being considered statistically significant.

**Results**: 405 singleton pregnancies were analyzed (201 history‐indicated, 132 ultrasound‐indicated, 72 exam‐indicated). Groups were similar in terms of BMI, race, ethnicity, marital status and insurance type, but exhibited a statistical difference in maternal age and nulliparity. Patients in the EI cohort delivered earlier than HI and US groups at an average gestational age of 33.7 w (p = 0.004) . Delivery at < 32 w was most common in EI and US cohorts compared to HI (30.6% vs 18.9% and 12.9%, p = 0.004). Delivery < 32w and < 28w were associated with EI and US groups compared to HI after controlling for confounders . Incidence of neonatal Grade III or IV IVH, sepsis and NICU admission were higher in the EI and US groups compared to HI group. Duration of NICU stay (in days) and incidence of fetal or neonatal demise were greater in the EI cohort compared to the HI group, and these findings persisted in the adjusted analysis (aLRC 37.63, 95%CI 2.82‐72.43 and aOR 9.01, 95% CI 1.25‐64.87, respectively).

**Conclusion**: Exam‐indicated cerclage is associated with delivery at earlier gestation, greater risk of delivery at < 32 w and < 28 w, longer NICU admission, and increased risk of fetal or neonatal demise compared with history‐indicated cerclage.

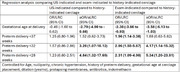

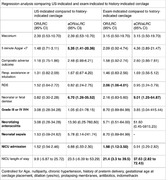

## 465 | Evaluation of Prophylactic Antibiotic use in Ultrasound‐Indicated Cerclage Placement in a Large Us Hospital System

David M. Esterquest^1^; Rachel K. Harrison^2^; Calla Holmgren^2^

*^1^Advocate Lutheran General Hospital, Advocate Lutheran General Hospital, IL; ^2^Advocate Health Care, Advocate Health Care, IL*

4:00 PM ‐ 6:00 PM

**Objective**: Compare pregnancy outcomes in patients that did and did not receive antibiotic prophylaxis for ultrasound‐indicated cerclage placement

**Study Design**: Retrospective cohort study of singleton pregnancies undergoing ultrasound‐indicated (US) cerclage at multiple hospitals within a large US hospital system between 2020‐2023. Subjects were included if they met criteria for US‐indicated cerclage. They were excluded if they did not have detailed operative notes and pre‐ and post‐op documentation, or if the maternal and neonatal outcomes were not available. Patients were separated into two groups: those who received antibiotics at the time of cerclage placement and those that did not. Patient demographics, socio‐economic factors, and medical co‐morbidities were compared as well as operative findings and use of indomethacin.  The primary outcome was gestational age at delivery. Secondary outcomes included rates of cesarean section and preterm delivery at < 37 weeks (w), < 32 w, and < 28 w. Student's t‐test, chi‐squared, and logistic and linear regression were used for statistical analysis, and p‐value < 0.05 was considered statistically significant.  

**Results**: 127 singleton pregnancies were analyzed, with 97 receiving antibiotics. Patients across both cohorts were similar in terms of age, parity, BMI, race, ethnicity, marital status, and type of insurance. Additionally, patients did not differ significantly regarding their medical co‐morbidities. Those who received antibiotics were more likely to have a dilated cervix at the time of cerclage placement (53.6% vs 26.7%, p = 0.010) as well as prolapsed membranes (17.5% vs 0%, p = 0.014). Indomethacin use did not vary significantly between groups. Gestational age at delivery as well as incidence of delivery at < 37 w, < 32 w, and < 28 w did not vary significantly between the two groups, even when adjusting for confounding factors (table). There was no difference in rate of cesarean between the groups. 

**Conclusion**: Antibiotic use at the time of ultrasound‐indicated cerclage is not associated with a decreased risk for preterm delivery.

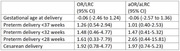

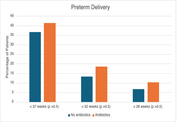

## 467 | Maternal Morbidity Disparities Amongst Native Hawaiian and Pacific Islander Populations Following Implementation of Safety Bundles

Deepraj K. Pawar^1^; Mary Tschann^2^; Julie Kathman^3^; Rebecca Delafield^4^; Pai‐Jong Stacy Tsai^2^

*^1^University of Hawaii, Honolulu, HI; ^2^University of Hawaii, John Burns School of Medicine, Honolulu, HI; ^3^Health Association of Hawaii, Honolulu, HI; ^4^University of Hawaii, Manoa, Honolulu, HI*

4:00 PM ‐ 6:00 PM

**Objective**: The Alliance for Innovation on Maternal Health (AIM) has developed evidence‐based patient safety bundles (PSB) to decrease severe maternal morbidity (SMM) for several obstetric outcomes including  hemorrhage, severe hypertension, and sepsis. Ten of 12 hospitals with birthing facilities in Hawaii adopted AIM PSB in 2021. With evidence of racial disparity among SMM, there is limited data on Native Hawaiian and Pacific Islander people. Our goal was to examine racial and ethnic differences in SMM among OB hemorrhage cases since the implementation of AIM PSB in Hawaii.

**Study Design**: We abstracted AIM SBP data from the AIM Data Center, and stratified SMM among OB hemorrhage  (excluding ectopic pregnancy and spontaneous abortion) by patient race and year.  The groups reported were those with 5 or more incidence of hemorrhage in any given year: Japanese, Filipino, Native Hawaiian, Micronesian, Other Polynesian, Non‐Hispanic White, and Hispanic.

**Results**: From 2016 to 2023, 9545 deliveries were included. Micronesian patients had the highest average SMM rates among OB hemorrhage (27.1%, Table 1), then Other Polynesian (22.7%)  and Hispanic (22.1 %) patients. Japanese patients had the lowest rate (18.7%). By year, Micronesian patients had a 30.4% and 32.3% SMM rate in 2020 and 2021, respectively, to 20.2% by 2023 (Graph 1) . Similarly, patients identifying as Other Polynesian had a 38.6%, 22.8%, and 25% SMM rate from 2019‐2021, to 14.9% and 18.9% in 2022 and 2023. Native Hawaiian patients had a SMM rate of 20.4% in 2021, 18.7% in 2022, and 22.1% in 2023.

**Conclusion**: Micronesian and Polynesian patients experienced clinically significant reduction in SMM among OB hemorrhage after the implementation of AIM PSB in 2021. Native Hawaiian patients did not experience a similar decrease. Native Hawaiian and Pacific Islanders are overrepresented in Hawaiʻi's birthing population compared to the general population, but experience worse outcomes relative to other racial groups.  Further studies are warranted to investigate why select populations may benefit from these interventions and how we might provide more equitable care.

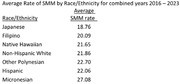

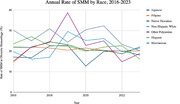

## 468 | Timing of Delivery in Pregnancies Complicated by Small for Gestational Age with Concurrent Chronic Hypertension

Deirdre Buckley^1^; Kavisha Khanuja^1^; Moti Gulersen^2^; Huda B. Al‐Kouatly^3^; Rodney A. McLaren, Jr., Jr.^3^

*^1^Sidney Kimmel Medical College at Thomas Jefferson University, Philadelphia, PA; ^2^Sidney Kimmel Medical College of Thomas Jefferson University, Philadelphia, PA; ^3^Thomas Jefferson University Hospital, Philadelphia, PA*

4:00 PM ‐ 6:00 PM

**Objective**: The American College of Obstetricians and Gynecologists recommends delivery at 34 0/7 ‐ 37 6/7 weeks for pregnancies complicated with fetal growth restriction and concurrent maternal conditions, such as chronic hypertension (CHTN), based on limited evidence. Our objective was to assess neonatal morbidity at birth for each week of gestation in births complicated with small for gestational age (SGA) and CHTN to assess optimal timing of delivery between 34 0/7 ‐ 37 6/7 weeks.

**Study Design**: This was a population‐based cohort study of non‐anomalous, singleton, live births complicated by CHTN and SGA between 34 and 37 completed weeks using data from the U.S. Natality Vital Statistics Database from 2015 to 2022. Planned deliveries were births at each completed week from 34 to 37 weeks that had a labor induction or cesarean delivery without labor, excluding spontaneous labor. Expectant management group included all births that delivered on or after the following week (i.e., planned birth at 34 0/7‐34 6/7 compared to all births ≥ 35 0/7 weeks). The primary outcome was a neonatal adverse composite, which was compared between both groups at each week using multivariable analyses. 

**Results**: There were 157,985 births that met inclusion criteria. After adjusting for the differences between both groups, planned delivery prior to 36 weeks was associated with greater odds of neonatal morbidity than those expectantly managed (planned 34 weeks birth: 20.3% vs expectant births > 35 weeks: 7.1%, aOR 3.83, 95% CI 3.12‐4.70; planned 35 weeks birth: 13.8% vs expectant births > 36 weeks: 5.8%, aOR 2.18, 95% CI 1.74‐2.75). Planned delivery ≥ 36 0/7 had similar odds of composite neonatal morbidity than those expectantly managed (Figure 1).

**Conclusion**: In births complicated by SGA and concurrent CHTN, planned delivery prior to 36 weeks was associated with greater odds of neonatal morbidity compared to expectant management. This data suggests that optimal delivery timing is ≥ 36 weeks. A randomized trial is needed to confirm these findings.

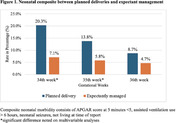

## 469 | Change in Late Preterm PROM Guidelines Result in Increased Expectant Management

Derek Lee^1^; Helena Randle^2^; Tara A. Lynch^1^

*^1^Albany Medical Center, Albany, NY; ^2^University of Maryland Medical Center, Baltimore, MD*

4:00 PM ‐ 6:00 PM

**Objective**: To investigate the rates of expectant management of preterm prelabor rupture of membranes (PPROM) past 34w0d before and after practice guideline changes in March 2020 at a tertiary level medical center and the corresponding maternal and neonatal outcomes.

**Study Design**: This is a retrospective cohort study of 299 gravid, singleton patients who underwent PPROM prior to 36w6d and delivered at 34w0d to 36w6d between June 2015 and June 2022. The primary outcome is NICU admission rate, and secondary outcomes include PPROM latency, corticosteroid administration, composite neonatal morbidity (5‐minute Apgar < 7, umbilical artery pH < 7.10, assisted ventilation, respiratory distress syndrome, hypoxic ischemic encephalopathy, antibiotic administration >72 hours, intraventricular hemorrhage, seizures, death) and composite maternal morbidity (chorioamnionitis, placental abruption, maternal sepsis, maternal transfusion).

**Results**: 176 patients (58.9%) before and 123 patients (41.1%) after March 2020 were included. No patients before 2020 and 6 patients (4.8%) after 2020 experienced PPROM before 34w0d and delivered in the late preterm period. There was no difference in PPROM and delivery gestational ages (Table 1). PPROM latency was longer after 2020 (1.01±0.32 vs. 0.52±0.11 days, p = 0.001). More patients underwent expectant management (26.8% vs. 11.9%, p< 0.001) and received corticosteroids (78.0% vs. 60.2%, p = 0.001) after 2020. There was no difference in NICU admission rates and composite neonatal and maternal morbidities (Table 1). In the patients who underwent expectant management, PPROM occurred at a higher gestational age prior to March 2020 (Table 2, p = 0.04). There was no difference in delivery gestational age, latency, NICU admission rates, and composite neonatal and maternal morbidities in patients who underwent expectant management (Table 2).

**Conclusion**: Significantly more patients underwent expectant management of PPROM past 34w0d after March 2020, but there was no difference in NICU admission rates and composite maternal and neonatal morbidities, even in those who underwent expectant management.

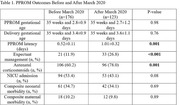

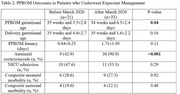

## 470 | Comparison of Weight Gain versus BMI During Pregnancy on Perinatal Outcomes in Singleton Term Pregnancies

Devangi Dave Mahtani; Eleanor R. Germano; Irene A. Stafford; Han‐Yang M. Chen

*McGovern Medical School at UTHealth Houston, Houston, TX*

4:00 PM ‐ 6:00 PM

**Objective**: Excessive gestational weight gain (GWG) and obesity have significant adverse perinatal risks. Our study compares the impact of excessive GWG on maternal and neonatal outcomes in pregnancies with normal pre‐pregnancy BMI (18.5–24.9) to those who had pre‐pregnancy BMI ≥ 25 with recommended weight gain.

**Study Design**: We performed a retrospective cohort study using the National Vital Statistics Database and included singleton, non‐anomalous live births who delivered at term from 2016‐2022. We excluded patients with multifetal gestations, chronic hypertension and pre‐gestational diabetes. We compared three groups: Group 1 (normal pre‐pregnancy BMI with excessive GWG), Group 2 (overweight pre‐pregnancy BMI (25.0–29.9) with recommended GWG) and Group 3 (obese pre‐pregnancy BMI (≥ 30) with recommended GWG). Recommended GWG was defined according to AIUM standards across BMI strata. Multivariate Poisson regression models with robust error variance were used to examine the association. Adjusted relative risk (aRR) with 95% confidence intervals were calculated.

**Results**: Our study included 6,352,304 patients. Compared to Group 1, overweight and obese patients had a higher risk of composite adverse maternal outcome, mostly due to increased risk of gestational diabetes and cesarean birth. Those with recommended GWG despite being overweight or obese were less likely to have hypertensive disorders (Group 2 aRR 0.92, 95% CI 0.91‐0.92) and operative vaginal birth (Group 2 aRR 0.93, 95% CI 0.92‐0.94; Group 3 aRR 0.73, 95% CI 0.72‐0.74). Compared to Group 1, Group 2 had a reduced risk of the composite neonatal adverse outcome (aRR 0.74, 95% CI 0.74‐0.74), mostly due to a reduced risk of large for gestational age fetuses (aRR 0.64, 95% CI 0.64‐0.64) and NICU admission (aRR 0.98, 95% CI 0.98‐0.99).

**Conclusion**: While pre‐pregnancy BMI is a crucial predictor of these outcomes, recommended weight gain during pregnancy mitigates these risks by improving maternal and neonatal outcomes. Additional studies are needed to quantify the risk of excessive GWG in those with other high‐risk conditions.

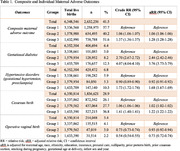

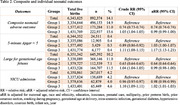

## 471 | The Impact of Maternal Comorbidities on Neonatal Outcomes in Growth Restricted Fetuses

Dhara Kadakia^1^; Olivia Grubman^2^; Hajer Naveed^3^; Mia A. Heiligenstein^4^; Thomas Owens^1^; Guillaume Stoffels^3^; Nathan S. Fox^5^; Zainab Al‐Ibraheemi^1^; Lois Brustman^1^

*^1^Icahn School of Medicine at Mount Sinai West, New York, NY; ^2^Westchester Medical Center, Westchester, NY; ^3^Icahn School of Medicine at Mount Sinai, New York, NY; ^4^Mount Sinai West, Astoria, NY; ^5^Icahn School of Medicine at Mount Sinai Hospital, New York, NY*

4:00 PM ‐ 6:00 PM

**Objective**: To determine the impact on neonatal outcomes in fetal growth restricted fetuses (FGR) in patients with and without medical comorbidities.

**Study Design**: A retrospective cohort study performed on singleton gestations from 2018‐2022.Patients included had FGR at the last scan prior to delivery. A composite of maternal comorbidities included: chronic hypertension(cHTN), pregestational diabetes(DM), connective tissue disease(CT), IVF, IBD, thrombophilia and maternal age >40. A sub‐group of comorbidities with a vascular component (DM, cHTN, CT) was reviewed. FGR was divided into 3 groups: Mild(mFGR): EFW >10% with AC< 10%, Moderate (MFGR):EFW 3rd‐10%, Severe (sFGR):EFW< 3rd%.The primary outcome was a neonatal composite: NICU Admission, APGAR < 5 at 5 minutes, and hypoglycemia. Logistic regressions were used to examine the relationship between maternal comorbidities, timing of FGR diagnosis, and neonatal outcomes.

**Results**: Total patients were 534: 297 mFGR, 184 MFGR, and 53 with sFGR. Of 534 patients, 105(19.7%) had comorbidities (of these 48 had vascular comorbidities); 429 had no comorbidities. 127(23.8%) of 534 had an adverse neonatal outcome. Adverse outcomes increased significantly with severity of FGR:15.8% in mFGR; 26.6% in MFGR;58.5% in sFGR. In a univariable analysis, cHTN, AMA, early FGR diagnosis, and FGR severity were significantly associated with an increased risk of adverse outcomes (Table 1).The odds of an adverse outcome were 2.17 times more in those with any comorbidity compared to those without comorbidities(OR 2.17, 95% CI 1.37‐3.4).When a vascular comorbidity was present, an adverse outcome was 4.50 times more likely compared to those with no comorbidities(OR 4.5, 95% CI 2.45‐8.28).The presence of a vascular comorbidity < 32wks had a significant association with an adverse neonatal outcome in all severities of FGR (Table 2).

**Conclusion**: In the setting of FGR, the presence of maternal medical comorbidities, particularly vascular, had significantly increased risk of adverse neonatal outcomes.These findings could help us better counsel patients in regards to antepartum testing and patients' expectations.

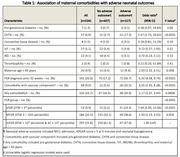

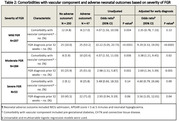

## 472 | Determining the Role of Pregnancy‐Induced Alterations to B Cells in Decreasing Risk for Breast Cancer

Dhivyaa Anandan^1^; Marygrace Trousdell^2^; Amritha Varshini Hanasoge Somasundara^2^; Camila dos Santos^2^

*^1^Stony Brook Medicine, Stony Brook, NY; ^2^Cold Spring Harbor Laboratory, Cold Spring Harbor, NY*

4:00 PM ‐ 6:00 PM

**Objective**: An early age of first pregnancy (< 25 years) decreases the lifelong risk of developing breast cancer (BC). This study characterizes how B cells that infiltrate the gland during pregnancy and lactation may play a role in preventing BC.

 **Study Design**: Wildtype female mice and a mouse model of Brca1 loss (Krt5^CRE‐ERT2^Brca1^fl/fl^p53^+/‐^) were used in this study. Single‐cell RNA‐seq (scRNA‐seq) datasets were generated from mammary tissue of nulliparous and parous mice with Brca1 loss without tumors. B cell receptor (BCR)‐seq datasets were generated from nulliparous and parous wildtype mice. Differential gene expression and clustering analyses were performed using the R pipeline Seurat. Clonotype analysis and extraction of BCR sequences were performed using Loupe V(D)J Browser by 10X Genomics. Recombinant post‐pregnancy mammary IgG antibodies were synthesized by the CSHL Antibody & Phage Display Core Facility and tested by immunofluorescent (IF) staining.

 **Results**: scRNA‐seq analysis showed that parous mice with Brca1 loss have an expansion of antibody‐secreting B cells in their mammary glands (MGs), suggesting that B cells that infiltrate the MG during pregnancy remain there and may secrete antibodies that inhibit mammary tumor formation. To analyze these B cells on a clonal level, we analyzed BCR‐seq data to identify the most abundant IgG clones unique to the parous MG. We extracted the sequences of these clones and used them to synthesize recombinant IgG antibodies. Using IF staining of mammary tissue from healthy mice (nulliparous, pregnant, and parous) and mice with mammary pre‐neoplasia, we found that post‐pregnancy IgG antibodies recognize antigens induced by pregnancy and lactation that are also present in mammary pre‐neoplastic tissue. We are currently testing these antibodies in our mouse model of Brca1 loss to evaluate their ability to block mammary tumor development.

**Conclusion**: In addition to their role in secreting antibodies into breastmilk that confer immunity to the nursing infant, our results suggest a role for pregnancy‐induced antibody‐secreting B cells in long‐term breast oncoprotection.

## 473 | Change in BMI Category: Impact on Maternal and Neonatal Outcomes

Eleanor R. Germano; Devangi Dave Mahtani; Irene A. Stafford

*McGovern Medical School at UTHealth Houston, Houston, TX*

4:00 PM ‐ 6:00 PM

**Objective**: Pre‐pregnancy BMI is a risk factor for the development of hypertensive disorders of pregnancy (PIH). However, the association between weight gain during pregnancy and the development of hypertensive disorders is understudied. The objective was to determine if change in BMI category change (eg overweight to class I obesity) was associated with development of PIH. Secondary outcomes were maternal composite outcome and fetal composite outcome.

**Study Design**: This was a retrospective cohort study performed using data from the National Vital Statistics Database of individuals with singleton, non‐anomalous neonates from 2016‐2020. Pregnancies with multiple gestations, anomalous neonates, pre‐gestational diabetes, or chronic hypertension were excluded. Demographic and obstetrical variables were recorded. Groups were determined as no change in BMI (Group 1) or change in BMI (Group 2) from pre‐pregnancy to delivery. Differences between groups were examined using chi‐square or Fisher's exact test. Multivariate regression models were used to calculate adjusted relative risk (aRR) with 95% confidence intervals (CI).

**Results**: A total of 19,718,555 pregnancies met inclusion criteria and 15,426,805 (78%) pregnancies were noted to have a change in BMI category. The overall rate of PIH was 6.6%. After multivariable adjustment, there was a significant difference in the rate of PIH. Compared to individuals in Group 1, individuals with a Group 2 were more likely to develop PIH (aRR of 1.39 (95% CI [1.38‐1.39], p< 0.001). In the secondary outcome analysis, Group 2 compared to Group 1 had higher rates of maternal composite outcome (aRR 1.11, CI [1.11‐1.11, p< 0.001]) and fetal composite outcome (aRR 1.37, CI [1.37‐1.38], p< 0.001).

**Conclusion**: There was a significant association between change to a higher BMI category and development of PIH along with an increase in composite adverse maternal and fetal outcomes. Future studies are required to further delineate risks for patients whose delivery BMI changes to class III obesity as well as stratified risks for women with pre‐pregnancy class IV obesity and above.

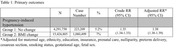

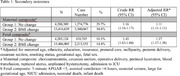

## 474 | The Impact of Watching an Informative‐Video on Possible Obstetric Emergencies Before Labor‐A Randomized Controlled Trial

Eliel Kedar Sade^1^; Ilia Kleiner^2^; Shir Lev^2^; Shiran Rona^2^; Daniel Tairy^3^; Hadas Miremberg^2^; Jacob Bar^2^; Eran Weiner^1^; Noa Gonen^4^

*^1^Wolfson Medical Center, Wolfson Medical Center, Tel Aviv; ^2^Wolfson Medical Center, Wolfson Medical Center, HaMerkaz; ^3^Edith Wolfson Medical Center, Holon, HaMerkaz; ^4^Sheba Medical Center, Ramat gan, HaMerkaz*

4:00 PM ‐ 6:00 PM

**Objective**: In most obstetric departments women (especially nulliparous) are rarely counseled in detail regarding possible obstetric emergencies that might occur during labor.  This randomized controlled trial aimed to evaluate the effect of an informative video regarding possible obstetric emergencies during labor on maternal anxiety, childbirth experience, and peripartum satisfaction among nulliparous women anticipating vaginal delivery.

**Study Design**: Participants (≥37 weeks gestation) were randomly assigned to an intervention group or a control group. The intervention group watched a 5‐minute video planned and produced by our department on the management of delivery and “what to expect” during common obstetric emergencies, including fetal bradycardia, assisted delivery, emergency cesarean delivery, and postpartum hemorrhage. The control group received standard obstetric care. Anxiety levels were assessed at three points using the State‐Trait Anxiety Inventory (STAI): at recruitment prior to video exposure (STAI 1), upon admission to the delivery room (STAI 2), and postpartum (STAI 3). Both groups also completed an 11‐item Childbirth Experience Questionnaire (CEQ). The study was powered to detect a 5 points difference in the primary outcome (STAI 2).

**Results**: A total of 161 participants were approached, and 127 were included in the final analysis after completing all study questionnaires. Of these, 63 were in the exposure group, and 64 in the control group (figure). There were no significant demographic differences between the groups, and baseline anxiety levels were comparable (STAI 1). Upon admission to the delivery room, anxiety levels remained similar between the control and exposure groups (STAI 2: 41 [33‐48] vs. 42 [34‐48], respectively; p = 0.84). Notably, immediate postpartum anxiety levels were significantly lower in the exposure group (STAI 3: 30 [24‐38] vs. 28 [21‐33]; p = 0.03). There were no differences in the CEQ between the groups (table).

**Conclusion**: Watching the pre‐delivery informative video about possible obstetric complications was associated with lower anxiety levels immediately postpartum.

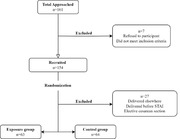

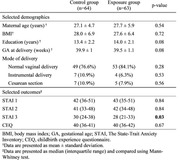

## 475 | Differences in Intimate Partner Violence Screening During Pregnancy for Us‐Born Versus Immigrant Birthing People

Elizabeth L. Mirsky^1^; Ashley Boerrigter^1^; Gregory Hawk^1^; Emily A. DeFranco^2^

*^1^University of Kentucky, Lexington, KY; ^2^Department of Obstetrics and Gynecology, University of Kentucky, Lexington, KY*

4:00 PM ‐ 6:00 PM

**Objective**: Investigate whether socioeconomic and language barriers to healthcare unique to immigrant birthing people in the US are reflected in differences in frequency of screening for intimate partner violence (IPV) in the preconception, pregnancy, and postpartum periods.

**Study Design**: Cross‐sectional study using Pregnancy Risk Assessment Monitoring System (PRAMS) data from 2020 to 2021. PRAMS collected data on screening for physical and emotional IPV before, during and after pregnancy, as well as experience of IPV before and during pregnancy. The prevalence of screening was compared between immigrant versus US‐born status, and relative risks calculated. Weighted analyses of PRAMS data were employed to be reflective of representative state populations using predefined methods.

**Results**: There were 63,761 respondents in the study period, representative of an estimated population of 3,039,459 from participating US states. Sociodemographic characteristics differed between US‐born and immigrant status (Table 1). Approximately 3 out of 4 respondents reported being screened for IPV during pregnancy, with screening rates lower for IPV before and after pregnancy. Immigrant respondents were overall less likely to be screened for IPV before pregnancy than US‐born, but more likely to be screened during and after pregnancy (Table 2). US‐born respondents were more likely to experience IPV before and during pregnancy than immigrant respondents; however, there was no significant association between being screened for IPV and foreign‐born status among those reporting having experienced IPV.

**Conclusion**: Approximately 1 in 4 patients reported not been screened for IPV. Among those who reported experiencing IPV, over one‐third were not screened. While multiple barriers to IPV screening exist, immigrant‐born status was not associated with lower rates of IPV screening during or after pregnancy. This highlights the importance of IPV screening among all pregnancies, regardless of perceived risk status.

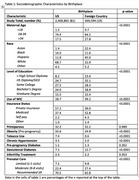

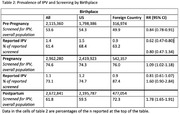

## 476 | Prenatal Fentanyl Exposure and Replication of a Novel Neonatal Syndrome Study at a Single Institution

Elizabeth L. Mirsky; Cynthia Cockerham; Wendy Whitley; Gregory Hawk; Asmita Shrestha; John O'Brien; Barbara V. Parilla

*University of Kentucky, Lexington, KY*

4:00 PM ‐ 6:00 PM

**Objective**: To determine if findings from a previous study on a novel neonatal syndrome associated with prenatal fentanyl exposure (Wadman et al., 2023) can be identified at our institution.

**Study Design**: Potential cases of this novel syndrome were identified from a prospectively collected database of obstetric patients with substance use disorder (SUD) enrolled in a multispecialty treatment program from 2014 to 2024. The database was screened for neonatal findings consistent with this novel syndrome in cases with prenatal fentanyl exposure resembling Smith‐Lemli‐Opitz (SLO) syndrome including microcephaly, cleft palate, clubfoot, rocker bottom feet, toe syndactyly, single palmar crease, hypoplastic corpus callosum, and hypospadias.

**Results**: 639 patients were enrolled in a perinatal substance use treatment program and study.  Of the 103 patients found to have neonates with a small head size (head circumference < 10%), 34 individuals self‐reported fentanyl use in the 30 days prior to enrollment in the program, with an additional 17 reporting use within the last year. Six of these individuals had confirmatory drug screening for fentanyl. Of the 6 cases with confirmed fentanyl use on drug screening, all patients screened positive in the first trimester. Four neonates displayed characteristic anomalies consistent with a SLO‐like syndrome (Table 1). All cases shared lagging head growth, while additional anomalies identified included cleft palate, short nasal tip, thin upper lip, micrognathia, and hypospadias. Of the cases with anomalies, all individuals had polysubstance use. Genetic screening/diagnostic testing varied but an assessment of cholesterol metabolism was not performed. 

**Conclusion**: This study suggests the potential association of prenatal fentanyl exposure with a novel syndrome. As the opiate crisis continues, fueled by rising fentanyl use, more data is needed to confirm and delineate this association and determine the possible long‐term developmental effects of such a syndrome.

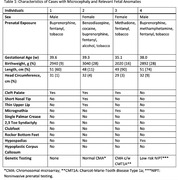

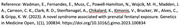

## 477 | Asymmetrical vs. Symmetrical Large for Gestational Age Infants in Pregestational Diabetics: Differences in Neonatal Morbidity

Ellen M. Murrin^1^; Barak Rosenn^2^; Shelley Ehrlich^3^; Jane Khoury^3^; Francis Mimouni^4^; Menachem Miodovnik^5^

*^1^Inova Fairfax Medical Campus, Falls Church, VA; ^2^Jersey City Medical Center, Jersey City, NJ; ^3^Cincinnati Children's Hospital Medical Center, Cincinnati, OH; ^4^Sackler School of Medicine Tel Aviv University, Tel Aviv, Tel Aviv; ^5^Inova Fairfax Medical Campus, Fairfax, VA*

4:00 PM ‐ 6:00 PM

**Objective**: Research has highlighted the importance of distinguishing between asymmetrical large for gestational age (aLGA) and symmetrical large for gestational age (sLGA) infants, with the former being associated with higher risk of neonatal complications. We hypothesized that the prevalence and complications of aLGA is higher in infants born to mothers with type 1 diabetes (T1D) compared to type 2 diabetes (T2D).

**Study Design**: Women with insulin‐treated pregestational diabetes (PGDM) were recruited and managed prospectively. Singleton pregnancies recruited before 14 weeks were included in this analysis. Patients were seen every 1‐2 weeks and glycemic control was strictly managed using standardized protocols. Infants were weighed and measured immediately after birth and monitored for hyperbilirubinemia, hypoglycemia, and acidosis. LGA (birthweight > 90^th^%ile) was categorized as aLGA if ponderal index (PI = weight/length^3^) was > 90^th^%ile for gestational age, and sLGA if the PI was ≤ 90^th^%ile. Outcomes were compared between infants with sLGA and aLGA and between infants born to mothers with T1D and T2D. Characteristics are reported as n (%), mean +/‐ standard deviation, or median and 25^th^ and 75^th^ percentiles. Statistical analyses included t‐test, Wilcoxon rank sum, chi‐square and logistic regression, as appropriate.

**Results**: The study included 132 LGA infants born to 321 women with T1D (41%) and 26 LGA infants born to 55 women with T2D (47%). The incidences of aLGA and sLGA in both groups and patient characteristics are presented in Table 1. In T2D, aLGA was associated with higher 2^nd^ and 3^rd^ trimester HbA1 compared to sLGA. There was a trend for a similar association in T1D. Most infant outcomes did not differ between groups nor did overall composite outcomes (Table 2).

**Conclusion**: There is a high rate of LGA, and particularly aLGA, among infants of mothers with both T1D and T2D. However, we identified few differences in infant outcomes between the groups, suggesting the need for further study into the clinical significance of aLGA as compared to sLGA.

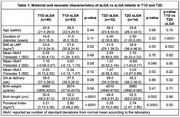

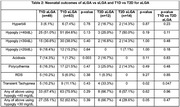

## 478 | Early Indicators and Clinical Characteristics of Preeclampsia in Pregnant Women with Insulin‐Treated Pregestational Diabetes

Ellen M. Murrin^1^; Barak Rosenn^2^; Shelley Ehrlich^3^; Jane Khoury^3^; Francis Mimouni^4^; Menachem Miodovnik^5^

*^1^Inova Fairfax Medical Campus, Falls Church, VA; ^2^Jersey City Medical Center, Jersey City, NJ; ^3^Cincinnati Children's Hospital Medical Center, Cincinnati, OH; ^4^Sackler School of Medicine Tel Aviv University, Tel Aviv, Tel Aviv; ^5^Inova Fairfax Medical Campus, Fairfax, VA*

4:00 PM ‐ 6:00 PM

**Objective**: Pregestational diabetes (PDM) is a known risk factor for preeclampsia (PE); however, various co‐existing factors can affect the risk of developing PE in this high‐risk population. The purpose of this study was to identify early indicators predictive of PE in pregnant women with insulin‐treated PDM.

**Study Design**: Pregnant women with PDM from a prospectively managed cohort were classified as with or without PE based on current ACOG criteria. Patients were seen every 1‐2 weeks during pregnancy and were managed by a dedicated team utilizing pre‐specified protocols. Individual clinical characteristics, diabetes‐related complications, blood pressure measurements, indices of glycemic control, and pregnancy outcomes were compared between patients who did and did not develop PE using t‐test, Wilcoxon rank sum test, or chi‐square, as appropriate.

**Results**: Patient characteristics are presented in Table 1 as n (%), mean ± standard deviation, or median and 25^th^ and 75^th^ percentiles. Of the 424 pregnancies, 87 (20.5%) developed PE. Duration of diabetes, microvascular complications, early pregnancy (8‐20 weeks) mean arterial blood pressure (MAP), and urine protein levels at 16 weeks were associated with an increased risk of developing PE. Weekly odds ratios for PE based on diastolic blood pressure were highest at 8‐10 weeks gestation. There was a trend towards higher Glycohemoglobin A1 (HgbA1) levels in the PE group during the first 20 weeks of pregnancy. PE was associated with earlier gestational age at delivery, lower birth weight, lower placental weight, and cesarean delivery. Live birth and neonatal death rates were similar in both groups (98% vs. 97% and 1% vs. 2% in the PE and non‐PE groups, respectively).

**Conclusion**: In PDM pregnancies, several factors may serve as early predictors for the risk of developing PE, including blood pressure and glycemic control early in pregnancy. These two modifiable risk factors suggest that there may be an opportunity to intervene early in pregnancy and possibly modify the risk of ultimately developing PE.

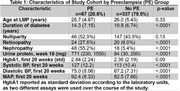

## 479 | Inequities in Postpartum Opioid Prescribing by Race‐Ethnicity and Insurance Status Across Statewide Quality Collaborative

Ellery G. Sarosi^1^; Joshua S. George^2^; Kyle Latack^1^; Steven Ater^3^; Michelle Moniz^2^; Lisa Kane Low^2^; Molly J. Stout^4^; Jourdan E. Triebwasser^2^; Jennifer Waljee^2^; Vidhya Gunaseelan^2^; Mark Bicket^2^; Courtney Townsel^5^; Alex Peahl^6^

*^1^University of Michigan Medical Center, Department of Obstetrics and Gynecology, Ann Arbor, MI; ^2^University of Michigan, Ann Arbor, MI; ^3^Central Michigan University, Central Michigan University, MI; ^4^University of Michigan Medical Center, Ann Arbor, MI; ^5^University of Maryland Department of Obstetrics, Gynecology and Reproductive Sciences, Baltimore, MD; ^6^Michigan Medicine, Ann Arbor, MI*

4:00 PM ‐ 6:00 PM

**Objective**: To describe differences in discharge opioid prescribing following vaginal (VB) and cesarean births (CB) across historically marginalized groups.

**Study Design**: Retrospective cohort study of nulliparous, term, singleton, vertex births from 1/1/2023‐10/13/2023 in a clinical registry from 71 hospitals participating in a quality improvement collaborative supported by Blue Cross Blue Shield of Michigan. Eligible patients had a VB or CB, were ≥ 18 years, and did not have a history of opioid use, postpartum length of stay ≥ 7 days, maternal transfer to another hospital, and invalid or incomplete opioid prescription data. Patients who underwent repair of a 3rd or 4th degree laceration, hysterectomy, or dilation and curettage were also excluded. We report adjusted opioid prescribing rates by race‐ethnicity and insurance status, stratified by mode of delivery.

**Results**: Of 14,690 VB, 87.4% (n = 12,837) had a spontaneous VB without additional procedures, of whom 1.1% (n = 146) received an opioid prescription. There was no significant difference in receipt of an opioid prescription based on race‐ethnicity or insurance after VB. Of 5,957 CB, 99.7% (n = 5,938) had a CB without additional procedures, of whom 89.2% (n = 5,298) received an opioid prescription. Following CB, NHB patients were more likely to receive an opioid prescription compared to NHW patients (Table 1; NHB: OR 1.57, 95% CI 1.16‐2.12). Patients with Medicaid were more likely and patients without insurance or self‐pay patients were less likely to receive an opioid prescription compared to privately insured patients (Table 1; Medicaid: OR 1.30, 95% CI 1.05‐1.62; Self‐pay/None: OR 0.43, 95% CI 0.20‐0.93).

**Conclusion**: Discharge opioid prescribing after VB without additional procedures was rare, and without inequities. In contrast, discharge opioid prescribing following CB was common, and higher among historically marginalized groups. 

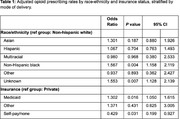

## 480 | Pregnancy Outcomes in Patients Living with Hiv Managed with Atazanavir Versus Darunavir‐Based Antiretroviral Therapy

Emily N. Adams^1^; George U. Eleje^2^; Ifeanyichukwu Ezebialu^3^; Ernie Shippey^4^; Muktar Aliyu^5^; Ahizechukwu C. Eke^6^

*^1^Johns Hopkins University, Baltimore, MD; ^2^Nnamdi Azikiwe University Teaching Hospital, Nnewi, Anambra; ^3^Chukwuemeka Odumegwu Ojukwu University Teaching Hospital, Amaku, Anambra; ^4^Vizient Center for Advanced Analytics, Chicago, IL; ^5^Vanderbilt University, Nashville, TN; ^6^Johns Hopkins University School of Medicine, Baltimore, MD*

4:00 PM ‐ 6:00 PM

**Objective**: Pregnant patients with HIV require effective antiretroviral therapy (ART) during pregnancy to prevent perinatal transmission and manage their own health, but the choice of regimen can impact pregnancy outcomes. Limited data are available comparing the safety and efficacy of second‐generation protease‐inhibitors (Atazanavir and Darunavir‐based ART) in pregnancy despite their associated risks of hyperbilirubinemia and cholestasis. Therefore, we compared the maternal and neonatal outcomes in pregnant patients living with HIV managed with Atazanavir versus Darunavir‐based ART.

**Study Design**: This multi‐center observational cohort study used data from 192 hospitals in 38 US states, sourced from the Vizient Clinical Database. We included pregnant patients with HIV on Atazanavir or Darunavir‐based ART, who delivered between January 2021‐ April 2024. The primary outcome was intrahepatic cholestasis of pregnancy (ICP), with secondary outcomes including hypertensive disorders, preterm birth, and neonatal outcomes. We computed adjusted odds ratios (aOR) using multivariable logistic regression to adjust for potential confounders (maternal age, gestational age, diabetes mellitus, hypertension, baseline viral load and CD4 cell count).

**Results**: There were 251 patients in the Atazanavir and 218 patients in the Darunavir group. In the overall cohort at study entry, the median (interquartile range, IQR) maternal age was 31 (IQR 27‐36) years with most patients identifying as Black (63.4%) . The frequency of ICP was similar in both groups (2.0% vs 2.8%; P = .59) ‐ *Table 1*. The adjusted odds ratios (aOR) of ICP (aOR 1.83; 95% CI 0.51, 6.58), preterm birth (aOR 0.93; 95% CI 0.41, 2.12), gestational hypertension (aOR 0.82; 95% CI 0.44, 1.54), pre‐eclampsia (aOR 1.07, 95% CI 0.58, 1.96), placental abruption (aOR 2.71; 95% CI 0.25, 28.93) and stillbirth (aOR 0.66; 95% CI 0.18, 2.25) were similar between both groups (*Table 2*).

**Conclusion**: Atazanavir and Darunavir‐based ART were safe for use during pregnancy and had similar pregnancy and neonatal outcomes.

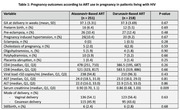

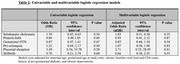

## 481 | Postpartum Hemorrhage Requiring Transfusion Increases Short‐Term Risk of Readmission for Cardiovascular Disease

Emily E. Daggett^1^; Rachel Lee^2^; Ruby Lin^2^; Morgan C. Dunn^3^; Julia Knypinski^2^; Cande V. Ananth^2^

*^1^Rutgers Robert Wood Johnson Medical School, Edison, NJ; ^2^Rutgers Robert Wood Johnson Medical School, New Brunswick, NJ; ^3^Rutgers Robert Wood Johnson, New Brunswick, NJ*

4:00 PM ‐ 6:00 PM

**Objective**: Postpartum hemorrhage (PPH) is a common obstetric complication that, in severe cases, may necessitate blood transfusion. We hypothesized that severe PPH constitutes a cardiovascular insult that confers risk beyond the immediate postpartum period and that necessitating blood transfusion serves as a marker of the severity of PPH. Severe PPH may plausibly be linked to cardiovascular disease (CVD) by large‐volume acute blood loss causing hypoperfusion and ischemia, leading to impaired cardiac function.

**Study Design**: We performed a retrospective cohort study of discharges from delivery hospitalizations in the United States, 2010‐2020, using the Healthcare Cost and Utilization Project's Nationwide Readmissions Database. ICD 9 and 10 coding were used to identify deliveries complicated by PPH with and without blood transfusion. Associations between PPH and non‐fatal CVD readmissions within the calendar year following delivery were derived from Cox proportional hazard models expressed in confounder‐adjusted hazard ratios (HR) with 95% confidence intervals (CI).

**Results**: Of 18,600,829 delivery admissions, 3.9% (n = 752,891) were complicated by PPH. Of these, 13.7% (n = 103,040) received a blood transfusion. The risk of CVD readmission was 170, 197, and 476 per 100,000 hospital deliveries for patients without PPH, PPH without transfusion, and PPH with transfusion, respectively. Compared to those without PPH, patients with PPH and no transfusion had an increased risk of CVD readmission (HR 1.19, CI 1.11‐1.28), with more than a doubling of risk among patients with PPH with transfusion (HR 2.20, CI 1.97‐2.47). The risks were highest after PPH with transfusion for atherosclerotic heart disease (HR 4.42, CI 3.04‐6.42), ischemic heart disease (HR 2.79, CI 2.09‐3.71), and acute myocardial infarction (HR 2.39, CI 1.58‐3.63)(Figure 1).

**Conclusion**: PPH, particularly when requiring blood transfusion, is associated with an increased risk of non‐fatal CVD readmission within the year following delivery. These findings underscore the burden of CVD risk with PPH and the need for active management to reduce CVD morbidity.

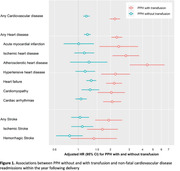

## 482 | Postpartum Hemorrhage Requiring Transfusion Increases Short‐Term Risk of Cardiovascular Disease Mortality

Emily E. Daggett^1^; Rachel Lee^2^; Ruby Lin^2^; Morgan C. Dunn^3^; Julia Knypinski^2^; Cande V. Ananth^2^

*^1^Rutgers Robert Wood Johnson Medical School, Edison, NJ; ^2^Rutgers Robert Wood Johnson Medical School, New Brunswick, NJ; ^3^Rutgers Robert Wood Johnson, New Brunswick, NJ*

4:00 PM ‐ 6:00 PM

**Objective**: Postpartum hemorrhage (PPH) is a common obstetric complication that, in severe cases, may necessitate blood transfusion. We hypothesized that severe PPH constitutes a cardiovascular insult that confers risk beyond the immediate postpartum period and that necessitating blood transfusion can serve as a marker of the severity of PPH. Severe PPH may plausibly be linked to cardiovascular disease (CVD) by large‐volume acute blood loss causing hypoperfusion and ischemia, leading to impaired cardiac function.

**Study Design**: Using the Healthcare Cost and Utilization Project's Nationwide Readmissions Database, we designed a retrospective cohort study of discharges from delivery hospitalizations in the United States, 2010‐2020. ICD 9 and 10 coding were used to identify deliveries  complicated by PPH with and without blood transfusion. Associations between PPH and CVD‐related mortality within the calendar year following delivery were derived from Cox proportional hazard models expressed in confounder‐adjusted hazard ratios (HR) with 95% confidence intervals (CI).

**Results**: Of 18,600,829 delivery admissions, 3.9% (n = 752,891) were complicated by PPH. Of these, 13.7% (n = 103,040) received a blood transfusion. The risk of CVD mortality was 3.3, 4.0, and 13.6 per 100,000 deliveries for patients without PPH, PPH without transfusion, and PPH with transfusion, respectively. Compared to those without PPH, patients with PPH and no transfusion had a similar risk of CVD mortality (HR 1.07, CI 0.71‐1.60). However, there was a three‐fold increased mortality risk among patients with PPH with  transfusion (HR 2.98, CI 1.65‐5.40), including heart disease mortality (HR 2.93, CI 1.53‐5.63)(Table 1).

**Conclusion**: While PPH without transfusion does not appear to increase the risk of CVD mortality in the year following delivery, PPH with transfusion (suggestive of disease severity) has a substantially higher risk of heart disease and stroke mortality. These findings underscore the significance of CVD risk with severe PPH and the need for active management to reduce CVD mortality.

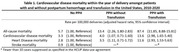

## 483 | Postpartum Hemorrhage is Associated with Reduced Rates of Breastfeeding in a Low‐Risk Nulliparous Population

Emma Trawick Roberts^1^; Sarah Heerboth^1^; Tracy A. Manuck^1^; Alison M. Stuebe^2^

*^1^University of North Carolina, Chapel Hill, NC; ^2^UNC School of Medicine, Chapel Hill, NC*

4:00 PM ‐ 6:00 PM

**Objective**: To examine the association of postpartum hemorrhage (PPH) with reduced rates of breastfeeding at 4‐8 weeks postpartum.

**Study Design**: Secondary analysis of a multicenter randomized controlled trial of elective induction of labor at 39 weeks’ gestation vs. expectant management in low‐risk nulliparous women from 2014‐2017 (MFMU ARRIVE trial). Participants with singleton, non‐anomalous pregnancies with no medical or obstetric indication for induction (as assessed at 38 weeks’ gestation/enrollment) were included. Those requiring ≥2 uterotonics in addition to oxytocin, transfusion, or surgical interventions to control bleeding (including peripartum hysterectomy) were considered to have PPH. At 4‐8 weeks postpartum, participants self‐reported infant feeding, categorized as exclusive breast (EBF), breast feeding in combination with formula (CF), or exclusive formula feeding (EFF). The primary outcome was any breastfeeding (EBF of CF) at 4‐8 weeks postpartum. Multivariable logistic regression was used to model the relationship between PPH and breastfeeding.

**Results**: Of 5454 participants that met inclusion criteria, 251 (5%) had a PPH, and 90/251 (36%) required blood transfusion. Clinical cohort characteristics are shown in table 1. At 4‐8 weeks postpartum, 33% were EBF, 31% were CF, and 35% were EFF. Of those with a PPH, 72 (28.7%) were EBF, 84 (33.5%) were CF, and 95 (37.9%) were EFF (p >0.05). In regression models, PPH was associated with a non‐statistically significant reduced odds of EBF or breastfeeding at all (Table 2). In regression models limited to those with PPH requiring blood transfusion, however, there was a significantly reduced odds of EBF or breastfeeding at all compared to those EFF (Table 2).

**Conclusion**: In a low‐risk nulliparous population, PPH requiring blood transfusion was associated with reduced odds of breastfeeding at 4‐8 weeks postpartum.  Women who have a PPH, especially if they require transfusion, may benefit from targeted breastfeeding support.

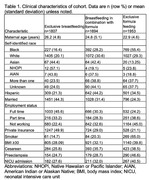

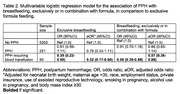

## 484 | First‐Trimester Virtual Placental Biopsy to Estimate Vascularization and Risk of Preeclampsia: a Prospective Study

Marie‐Laurence Côté^1^; Anne‐Sophie Lafortune^1^; Mario Girard^2^; Genevieve Marcoux^3^; Louise Ghesquiere^4^; Emmanuel Bujold^1^

*^1^Université Laval, PQ; ^2^CHU de Québec ‐ Research Center, CHU de Québec, PQ; ^3^CHU de Québec, CHU de Québec, PQ; ^4^Université de Lille, Lille, Nord‐Pas‐de‐Calais*

4:00 PM ‐ 6:00 PM

**Objective**: While preliminary studies have suggested its effectiveness (Dar et al. AJOG 2010), we aimed to assess whether 3‐dimensional power Doppler (3DPD) of the uteroplacental circulation space (UPCS) or of the whole placenta, measured in the first trimester was able to predict preeclampsia.

**Study Design**: A prospective observational study of singleton pregnancies between 11 and 14 weeks. The 3DPD indices, vascularization index (VI), flow index (FI), and vascularization flow index (VFI), were determined on a UPSC sphere biopsy with the virtual organ computer‐aided analysis (VOCAL) program. We also studied the vascularization of the entire placenta and the Doppler of the uterine arteries. Non‐parametric and ROC curve analyses were performed.

**Results**: Among 12,424 women recruited, 498 (4%) had preeclampsia, including 33 (0.3%) with early‐onset preeclampsia (< 34 weeks). We were able to determine that all vascularization indices, but particularly the VI and the VFI of the whole placenta and UPCS were largely decreased in early‐onset preeclampsia (all with p< 0.01). Taken alone, biopsy sphere VFI is faster to measure than uterine artery Doppler and has a sensitivity of 50% for early preeclampsia with a false‐positive rate of 15% (p< 0.001).

**Conclusion**: First‐trimester vascularization indices are decreased among women who will develop early‐osnet preeclampsia. Rapid assessment of placental vascularity by virtual UPCS biopsy, as proposed by Dar et al., is feasible on large populations, and is effective in predicting early‐onset preeclampsia.

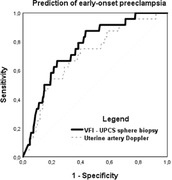

## 485 | Anticoagulation Preventive Therapy Versus no Therapy and the Risk for Venous Thromboembolism During the Postpartum

Enav Yefet^1^; Sally Hosari Mahamed^2^; Yael Skinez^2^; Zohar Nachum^3^

*^1^Tzafon Medical Center, Poriya, HaZafon; ^2^Emek medical center, Afula, HaZafon; ^3^Emak Medical Center, Afula, HaZafon*

4:00 PM ‐ 6:00 PM

**Objective**: Until 2011, enoxaparin was given for postpartum venous thromboembolism (VTE) prophylaxis in women with thrombophilia or previous VTE (pre‐protocol). Since 2012, VTE prophylaxis protocol was implemented and enoxaparin was given also to women with risk factors according to the RCOG. We aimed to compare the efficacy and safety of enoxaparin treatment postpartum before and after protocol implementation.

**Study Design**: Retrospective cohort study was conducted at Emek medical center in Israel. One delivery per woman was chosen randomly. The study included two periods; the pre‐protocol period between 2003 and 2011 (N = 19,783), and the protocol period from 2012 to 2020 (N = 20520). Enoxaparin was administered for 6 weeks in case of thrombophilia or past VTE and 7‐10 days in case of other risk factors. The primary outcome was the rate of VTE. Secondary outcomes were bleeding complications. Assuming that the protocol will reduce the rate for VTE from 1:1000 to 1:4000, 34,860 women were required (5% 2‐sided alpha, 80% power).

**Results**: Patients' characteristics and outcomes are presented in the table. In the pre‐protocol period, 531 women (2.7%) were treated with enoxaparin in the postpartum period. After protocol implementation, 964 (4.7%) women were treated due to known thrombophilia or past VTE event (P< 0.0001). Another 6.5% of the women received enoxaparin due to risk factors. The rate of VTE events in the postpartum period was 3 (0.02%) and 4 (0.02%) cases before and after the protocol implementation, respectively, (P = 1). Postpartum bleeding complications were comparable between the before and after protocol implementation groups, including postpartum hemorrhage (16 (0.08%) vs. 24 (0.12%), respectively, P = 0.25) or the need for blood transfusions (2 (0.01%) vs. 5 (0.02%), respectively, P = 0.45).

**Conclusion**: Protocol for postpartum VTE prophylaxis according to risk factors with enoxaparin treatment did not lower the rate of VTE events, which are rare to begin with. The guidelines for postpartum VTE prophylaxis should be re‐evaluated.

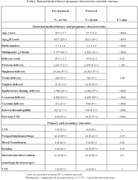

## 486 | Implementation of a Standardized Fetal Surveillance Protocol for Stillbirth Risk‐Reduction

Erik G. Holder^1^; Patrick S. Ramsey^2^; Camille B. Marquez^3^; John J. Byrne^4^

*^1^University of Texas Health Science Center at San Antonio, San Antonio, TX; ^2^University of Texas Health Science Center San Antonio, San Antonio, TX; ^3^University Health System, San Antonio, TX; ^4^University of Texas Health San Antonio, San Antonio, TX*

4:00 PM ‐ 6:00 PM

**Objective**: Antenatal surveillance is recommended by the American College of Obstetricians despite the lack of published data proving a direct reduction in fetal mortality. We sought to evaluate the impact of a standardized antenatal fetal surveillance protocol on stillbirth rates at our level IV maternal care, safety‐net hospital.

**Study Design**: Our institution implemented an updated, heightened antepartum surveillance protocol based on the ACOG Committee Opinion #828 in March of 2023. A retrospective cohort study was performed reviewing stillbirths one year pre‐ and one year post‐implementation of the protocol. Cases involving known, life‐limiting or lethal fetal anomalies, multi‐fetal gestations, and complete absence of prenatal care in the hospital system were excluded from the analysis. Stillbirth was defined as fetal death occurring between 20 0/7 weeks gestation until delivery. Secondary outcomes evaluated neonatal intensive care unit (NICU) admission rates, APGAR scores, and induction rates. Inductions were stratified into late preterm, early term, term, and late‐term groups. Chi‐square and Fisher's exact test were used for comparisons as appropriate to compare rates between cohorts.

**Results**: A total of 4078 deliveries occurred pre‐implementation (3/10/2022–3/10/2023) with 18 stillbirths identified (0.4%). A total of 4985 deliveries occurred post‐implementation (06/10/2023–06/10/2024), with 19 stillbirths identified (0.4%). Overall stillbirth rates did not change despite protocol implementation (p = 0.74), nor when evaluated by gestational age time epoch (p >0.05). Rates of induction, however, were significantly decreased for early term and term pregnancies (p = 0.02, p = 0.02, respectively) in the post‐implementation period. NICU admission rates and APGAR scores were not noted to have significant difference across groups.

**Conclusion**: Our data suggests that increased surveillance does not significantly impact stillbirth rates at our institution but does decrease incidence of early term and term inductions without adverse impact on NICU admission rates or APGAR scores.

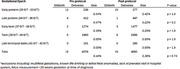

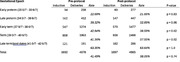

## 487 | Diagnostic Accuracy of Non‐Invasive Prenatal Test for Fetal Red Blood Cell Antigen Genotyping

Faezeh Aghajani^1^; Parisa Najjariasl^2^; Enaja Sambatur^3^; Alireza A. Shamshirsaz^4^; Asma Khalil^5^; Hiba J. Mustafa^6^

*^1^Maternal Fetal Care Center, Division of Fetal Medicine and Surgery, Boston Children's Hospital and Harvard School of Medicine, Boston, MA; ^2^Yas Hospital Complex, Tehran University of Medical Sciences, Tehran, Tehran; ^3^Fetal Care and Surgery Center, Division of Fetal Medicine and Surgery, Boston Children's Hospital and Harvard School of Medicine, Boston, MA; ^4^Fetal Surgeon Chief, Division of Fetal Medicine and Surgery Director of the Maternal Fetal Care Center Director of the Perinatal Surgery Fellowship Professor of Surgery, Boston Children's Hospital Harvard Medical School, Boston, MA; ^5^Fetal Medicine Unit, St George's Hospital, St George's University of London, London, England; ^6^Indiana University and Riley Children's Hospital, Indianapolis, IN*

4:00 PM ‐ 6:00 PM

**Objective**: To investigate non‐invasive prenatal testing (NIPT) diagnostic accuracy in identifying fetal red blood cell (RBC) antigen genotypes.

**Study Design**: A systematic search was conducted into four databases up to May 2024. We included studies implementing NIPT for fetal RBC antigen detection in pregnancies at risk of alloimmunization. We calculated sensitivity, specificity, diagnostic odds ratios, and area under the curve. Analysis was conducted per laboratory technique including polymerase chain reaction (PCR) and next‐generation sequencing (NGS) and subgroup analyses were performed per fetal antigen into RhC, Rhc, RhD, RhE, Kell, and Duffy (Fya) antigens.

**Results**: A total of 52 studies of which 48 (37228 maternal samples) used PCR and 4 (170 maternal samples) used NGS were included in the analysis. The diagnostic accuracy for the six antigens (RhC, Rhc, RhD, RhE, Kell, and Fya) in order are i) using PCR technique, the pooled sensitivity is 100% (95% C 99‐100), 98% (95% CI 95‐100), 100% (95% CI 99‐100), 100% (95% CI 98‐100), and 98% (95% CI 94‐100), respectively and the pooled specificity is 99% (95% CI 99‐100), 100% (95% CI 97‐100), 100%(95% CI 91‐100), 99% (95% CI 98‐99), 100% (95% CI 99‐100), and 100% (95% CI 97‐100), respectively, and ii) using NGS technique, the pooled sensitivity is 100% (95% CI 75‐100), 100% (95% CI 92‐100), 100% (95% CI 89‐100), 100% (95% CI 95‐100), 100% (95% CI 93‐100), 100% (95% CI 70‐100), respectively and the pooled specificity is 100% (95% CI 79‐100), 100% (95% CI 78‐100), 100% (895% CI 6‐100), 100% (95% CI 93‐100), 100% (95% CI 96‐100), 100% (95% CI 80‐100), respectively.

**Conclusion**: NIPT can accurately detect six fetal blood group antigens of which antibodies are associated with fetal anemia. Clinical adoption of NIPT for the detection of fetal antigens for both alloimmunized and RhD‐negative non‐alloimmunized pregnant individuals may streamline care and reduce unnecessary treatment, monitoring, and patient anxiety.

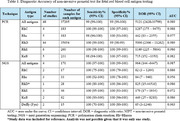

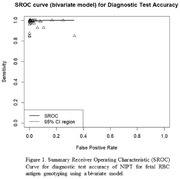

## 488 | Aspreo Secondary Analysis

Farah H. Amro^1^; Sean C. Blackwell^1^; Han‐Yang M. Chen^1^; Sandra Sadek^2^; Michal Fishel Bartal^3^; Baha M. Sibai^1^

*^1^McGovern Medical School at UTHealth Houston, Houston, TX; ^2^University of Texas Health Science Center in Houston, McGovern Medical School, Houston, TX; ^3^UTH Houston & Sheba Medical Center Israel, Houston, TX*

4:00 PM ‐ 6:00 PM

**Objective**: A recent meta‐analysis (4 trials) concluded that aspirin dose of 150‐162 mg vs 75‐81 mg initiated < 16 weeks gestational age (GA) decreased rates of preterm preeclampsia and preeclampsia with severe features (sPE). However, 3 of the 4 trials included were of low quality, and primary outcome was preeclampsia in only 1 of the trials. We conducted a secondary analysis of an RCT with primary outcome of sPE, and compared rates of sPE and preterm sPE between 162 vs 81 mg aspirin when initiated at < 16 weeks GA.

**Study Design**: Secondary analysis of a RCT in high‐risk obese gravidas comparing aspirin 162 (ASA 2) vs aspirin 81 mg (ASA 1) for preeclampsia prevention (ASPREO). Inclusion criteria were BMI >30 kg/m^2^ and > 1 high risk factor: PE in prior pregnancy, at least stage I hypertension in the index pregnancy, diabetes. Exclusion criteria were: delivery < 20 weeks GA or delivery outcome data not available.  For this analysis we focused on those enrolled < 16 weeks GA. The primary outcome was: sPE. Secondary outcomes were: preterm sPE, total PE, abruption, postpartum hemorrhage, severe maternal morbidity. Relative risks with 95% CIs were reported.

**Results**: A total of 107/209 (51%) were enrolled prior to 16 weeks GA (54 on ASA 2, 53 on ASA 1). Baseline characteristics were similar between groups (Table 1). There was no statistical difference in rates of sPE (28% vs 40%, RR: 0.7 (0.41‐1.21)), or preterm sPE (13% vs 24%, 0.53 (0.23‐1.22)) in those receiving ASA 2 vs ASA 1. Also, no differences were found in any of the analyzed secondary outcomes (Table 2).

**Conclusion**: When comparing aspirin 162 mg vs 81 mg started prior to 16 weeks, we found a trend towards reduced rates of preeclampsia with severe features and preterm preeclampsia with severe features in those receiving the 162 mg dose, although this was not statistically significant. A large RCT is needed to address the optimal dose and timing of initiation of aspirin for preeclampsia prevention.

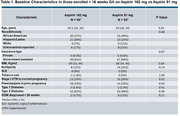

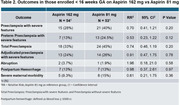

## 489 | Prediction of Cesarean Delivery Using Maternal Measurements–Secondary Analysis of a Prospective Multicenter Study

Rachel Malone^1^; Patrick Dicker^1^; Naomi Burke^1^; Ronan Daly^1^; Elizabeth Tunney^1^; Rebecca Boughton^1^; Alex Dakin^1^; Fergal D. Malone^2^

*^1^Royal College of Surgeons in Ireland, Dublin, Dublin; ^2^Royal College of Surgeons in Ireland, Dubiln, Dublin*

4:00 PM ‐ 6:00 PM

**Objective**: Prediction of intrapartum cesarean delivery (CD) would be of significant benefit in reducing labor‐associated morbidity. While different fetal sonographic biometric parameters have been studied to develop individual patient CD risks, they are subject to ultrasound resource limitations, as well as interobserver variability in ultrasound precision. Our objective was to evaluate whether simple, easily repeatable measurements of a pregnant woman's head circumference and her height could provide an easy method of CD risk prediction in routine antenatal clinics.

**Study Design**: A multi‐center prospective study recruited 2,336 nulliparous women with a vertex presentation between 39+0 and 40+6 weeks’ gestation at seven hospitals to examine a range of CD predictors. Maternal head circumference (MHC) and maternal height (MH) were recorded at the first antenatal visit, with data entered into a prospective database at study enrolment at 39+0 to 40+6  weeks’ gestation. Maternal proportionality of MHC to MH was assessed using the ratio MHC:MH.

**Results**: From a total enrolled cohort of 2,336 nulliparous patients, 491 (21%) had an unplanned CD. Mean MHC was 56.2cm (SD = 1.8cm, range = 50.0‐65.5cm). Mean MH was 165.5cm (SD = 6.3cm, range = 142‐188cm). The mean MHC:MH ratio was 0.346 (SD = 0.015), and this was used to standardize the MHC:MH ratio (sMHC:MH) for risk calculation. Table 1 shows that the sMHC:MH ratio was more predictive of emergency intrapartum CD than any maternal or fetal measurement individually (OR = 1.65, 95%CI 1.49‐1.83, p< 0.001, AUC = 0.64). Table 2 shows delivery outcomes based on quartile of sMHC:MH.

**Conclusion**: We have demonstrated that a simple ratio of MHC to MH is a useful predictor of CD, showing that the shortest patients with the largest maternal head measurements have a 1 in 3 chance of requiring CD, with CD at full dilation and failure to progress being significantly more likely. In particular when late pregnancy ultrasound resources may be limited, a simple maternal biometry ratio can be a powerful predictor of emergency intrapartum CD, at no cost.

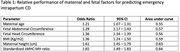

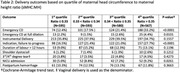

## 490 | Cost‐effectiveness of External Fetal and Maternal Heart Rate Monitoring vs. External Fetal Heart Rate Alone

Gabriela Hernandez Duran; Megha Arora; Aaron B. Caughey

*Oregon Health & Science University, Portland, OR*

4:00 PM ‐ 6:00 PM

**Objective**: Standard observation of a fetus during labor involves the use of external fetal monitoring to track fetal status. Recent evidence suggests that dual monitoring of external fetal heart rate (FHR) with concurrent maternal heart rate (MHR) can reduce the incidence of adverse neonatal outcomes. This study examined the cost‐effectiveness of concurrent MHR and FHR monitoring during labor.

**Study Design**: We constructed a decision‐analytic model using TreeAge Pro to compare neonatal outcomes between external FHR monitoring alone and dual external FHR and MHR monitoring. Outcomes examined included cost, neurodevelopmental delay (NDD), encephalopathy, death, NICU admission, and quality‐adjusted life years (QALYs). The theoretical cohort was 2,512,945 patients, the estimated annual singleton vaginal births at term. We applied a willingness‐to‐pay threshold of $100,000 per QALY for the incremental cost‐effectiveness ratio (ICER). The model inputs were sourced from literature and the QALYs were calculated using a 3% discount rate.

**Results**: In our theoretical cohort of 2,512,945 patients, dual MHR and FHR saved $843,454,354 and increased QALYs by 37,644. There would be 223 fewer patients with NDD, 1,131 fewer patients with encephalopathy, 8,325 fewer NICU admissions, and 291 fewer neonatal deaths. The negative ICER of $‐22,406/QALY suggests that not only is this strategy more effective, but also cost‐saving. Univariate sensitivity analysis of neonatal encephalopathy indicates that the dual monitoring would be cost‐saving once the probability of encephalopathy exceeds 0.06% (Figure 1).

**Conclusion**: In our study, the use of dual external FHR and MHR monitoring during labor is a cost‐saving strategy when compared to external FHR monitoring alone. Standardizing labor protocols to include external MHR monitoring may reduce dire neonatal outcomes.

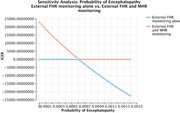

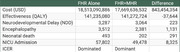

## 491 | Interpregnancy Interval and Uterine Rupture Risk in Women Undergoing Trial of Labor After Cesarean

Gal Bachar^1^; Naphtali Justman^1^; Ron Beloosesky^2^; Nira Gridish^2^; Dalia Jabarin^2^; Dana Vitner^1^; Zeev Weiner^2^; Yaniv Zipori^1^; Nizar Khatib^2^

*^1^Rambam Medical Center, Haifa, Hefa; ^2^Rambam Medical Health Center, Haifa, Hefa*

4:00 PM ‐ 6:00 PM

**Objective**: The interpregnancy interval (IPI) is the time between the end of one pregnancy and the start of the next. A short IPI, defined as 18 months or less, linked to negative outcomes like uterine rupture and preterm delivery. This study examines how the length of IPI affects the risk of uterine rupture and other major maternal and neonatal complications in women attempting a trial of labor after cesarean (TOLAC).

**Study Design**: A retrospective cohort study was conducted at a single tertiary care center between 2010 and 2024. The study population included pregnant women with a history of one previous cesarean delivery (CD) who attempted a TOLAC. Participants were stratified based on interpregnancy interval (IPI) of less than or greater than 18 months. The primary outcome was the incidence of uterine rupture. Secondary outcomes encompassed other maternal and neonatal complications.

**Results**: A total of 2386 women attempting TOLAC were included in the study, with 2151 (90.1%) in the IPI > 18 months group and 198 (9.9%) in the IPI < 18 months group. Baseline characteristics, including indication for previous CD, were comparable between the two groups (Table 1).

When attempting TOLAC, there was no significant difference in the incidence of uterine rupture between the two groups (0.5% vs. 0.8%, p = 0.235; Table 2). Vaginal delivery rate was also similar between group, with failed TOLAC rate was ∼38%. Other maternal and neonatal adverse outcomes did not differ significantly between the groups (Table 2).

**Conclusion**: Our study found no association between short IPI and an increased risk of uterine rupture or other maternal and neonatal complications in women attempting TOLAC. These findings suggest that IPI may not be a strong predictor of adverse maternal or neonatal outcomes in women undergoing TOLAC. However, the small number of women in the short IPI group, limits the strength of these conclusions.  Further research with larger sample sizes is needed to confirm these findings and to explore other factors that may influence outcomes in this population.

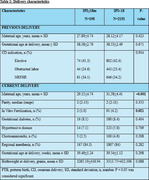

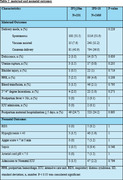

## 492 | Cost Analysis of Permissive Intrapartum Glucose Control for Diabetes: The PERMIT Randomized Controlled Trial

Ghamar Bitar^1^; Elenir B. Avritscher^2^; Kendra Folh^3^; Sarah A. Nazeer^4^; Sean C. Blackwell^1^; Baha M. Sibai^1^; Michal Fishel Bartal^5^

*^1^McGovern Medical School at UTHealth Houston, Houston, TX; ^2^University of Texas Health Science Center at Houston, Houston, TX; ^3^Children's Memorial Hermann Hospital, Houston, TX; ^4^The University of Texas Health Science Center at Houston (UTHealth), Houston, TX; ^5^UTH Houston & Sheba Medical Center Israel, Houston, TX*

4:00 PM ‐ 6:00 PM

**Objective**: In PERMIT randomized controlled trial (RCT), permissive intrapartum glucose control led to reduced need for maternal insulin drip and equivalent neonatal blood glucose when compared to usual care for patients with diabetes (Bitar, AJOG 2024). We aimed to evaluate its economic impact.

**Study Design**: This is a pre‐planned cost analysis of a multisite RCT of permissive intrapartum glucose control (target maternal blood glucose 70–180mg/dL) or usual care (70–110 mg/dL) in patients with diabetes. Costs were assessed from the hospital perspective and included the facility costs for all the inpatient services provided to all enrolled parturients and their neonates during the delivery and birth admissions, respectively, as recorded in the cost‐accounting system of the participating sites. Costs were inflated to 2024 based on the medical Consumer Price Index. We assessed differences in total combined maternal and neonatal costs (primary economic outcome), as well as individual maternal and neonatal costs, using frequentist generalized linear models (GLMs). We assessed the probability of reduction in costs using a Bayesian GLM model with a neutral prior assuming no benefit (rate ratio [RR] = 1.0; 95% credible interval, 0.3–3.3). All frequentist and Bayesian models used gamma distribution with log link and adjusted for the stratifying variables (site and type of diabetes).

**Results**: Costs were determined for all 96 maternal‐infant pairs. In intent‐to‐treat analysis, the total maternal and neonatal costs for permissive care were similar to those of usual care ($20,135 vs. $20,654; RR = 1.00 [95% CI, 0.77–1.30], p = 0.99; 49% probability of reduction).  Costs between permissive care and usual care were similar for both the parturients ($13,480 vs. $14,119; RR = 0.96 (0.78–1.18), p = 0.70; 69% probability of reduction) and the neonates ($6,655 vs. $6,535; 1.04 (0.63–1.73), p = 0.87; 41% probability of reduction).

**Conclusion**: Permissive care is clinically equivalent and cost‐neutral for both total maternal‐neonatal costs and individual costs. These findings require validation in additional sites.

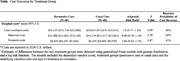

## 493 | Maternal Cardiac and Perinatal Outcomes Among Pregnant Individuals with Ebstein's Anomaly

Gianna L. Wilkie^1^; Rahi Patel^2^; Lara Kovell^3^; Anna R. Whelan^2^

*^1^University of Massachusetts Chan Medical School, Worcester, MA; ^2^University of Massachusetts Chan School of Medicine, Worcester, MA; ^3^UMass Memorial Health, Worcester, MA*

4:00 PM ‐ 6:00 PM

**Objective**: Ebstein's anomaly, a congenital cardiac condition, is defined by apical displacement of the tricuspid valve and atrialization of the inlet portion of the right ventricle. Advancement in cardiac intervention over time has led to an increase in people with Ebstein's anomaly surviving into the reproductive years. Due to its historical early lethality, the impacts of this condition are not well known in pregnancy. The objective of this study was to describe the maternal cardiac and perinatal outcomes associated with maternal Ebstein's anomaly in pregnancy through use of a large, contemporary dataset.

**Study Design**: Utilizing Epic Cosmos, an integrated dataset from over 1500 hospitals across the United States and includes 250 million patients, we identified a cohort of pregnancies among individuals with maternal Ebstein's anomaly. Individuals with simultaneous diagnoses codes for maternal Ebstein's anomaly and pregnancy between July 2021 and July 2024 were identified. Descriptive statistics, including demographics, cardiac and perinatal outcomes were obtained.

**Results**: During the study period, 487 individuals with a maternal Ebstein's anomaly and pregnancy were identified. Subjects were a mean age of 32.0 years old (±9 years) and identified as predominately White (78.9%). Approximately 30.8% of patients experienced a cardiac arrhythmia, while 14.8% developed heart failure. The most common arrhythmias were supraventricular tachycardias (SVT) (52%) and atrial fibrillation/flutter (36%). There was complete obstetric outcome data available for 235 individuals (48.2%). The average gestational age at delivery was 35.4 weeks (± 9.1 weeks) with 13.2% experiencing a preterm birth less than 37 weeks. Regarding mode of delivery, 77.0% had a vaginal delivery, while 23.0% had a cesarean delivery.

**Conclusion**: Ebstein's anomaly is associated with significant cardiac risks in pregnancy, specifically cardiac arrhythmia. Further prospective studies are needed in modern cohorts to guide optimal management of pregnant patients with Ebstein's anomaly and to determine the highest risk time period for cardiac complications.

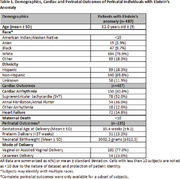

## 494 | Exposure to Maternal Chronic Hypertension (UNRELATED to PREECLAMPSIA) and Offspring Risk for Long‐Term Cardiovascular Morbidity

Gil Gutvirtz^1^; Tamar Wainstock^2^; Eyal Sheiner^2^

*^1^Soroka University Medical Center, Metar, HaDarom; ^2^Department of Obstetrics and Gynecology, Soroka University Medical Center, Ben‐Gurion University of the Negev, Beer‐Sheva, Israel., Beer Sheva, HaDarom*

4:00 PM ‐ 6:00 PM

**Objective**: Women with chronic hypertension are at an increased risk for adverse perinatal outcomes, such as superimposed preeclampsia and preterm labor. Both complications are associated with the maternal preexisting vascular disease that alters maternal adaptation to pregnancy and may expose the offspring for both short and long‐term adverse consequences. However, most studies regarding offspring long‐term outcomes included preeclampsia and chronic hypertension together. In this study we  investigate the long‐term cardiovascular morbidity of children exposed to maternal chronic hypertension, unrelated to preeclampsia.

**Study Design**: We conducted a population‐based retrospective cohort study of singleton deliveries occuring between the years 1991‐2021 at a tertiary center. Deliveries of women with chronic hypertension were compared with deliveries of women without chronic hypertension or any other hypertensive disorders of pregnancy. Offspring long‐term (up to 18 years) cardiovascular morbidity was compared using diagnoses from hospitalizations of the offspring involving a cardiovascular morbidity. A Kaplan–Meier survival curve was used to compare the cumulative incidence of cardiovascular morbidity and a Cox regression model was constructed to control for confounders.

**Results**: During the study period, 342,365 singleton deliveries occured, among them 3,097 (0.9%) were in mothers with chronic hypertension. Offspring total cardiovascular morbidity rate was significantly higher in children of mothers with chronic hypertension as compared with children from pregnancies uncomplicated by hypertensive disorders (**Table**). Also, the cumulative incidence of cardiovascular morbidity was higher for children exposed to maternal chronic hypertension (**Figure**). The Cox regression model, controlling for maternal and gestational age, found that maternal chronic hypertension is an independent risk factor for long‐term cardiovascular morbidity of the offspring (**Table**).

**Conclusion**: Maternal chronic hypertension, unrelated to preeclampsia, is an independent risk factor for offspring long‐term cardiovascular morbidity.

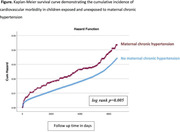

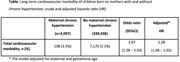

## 495 | Fertility Treatments Resulting in Twin Pregnancy and the Risk for Childhood Respiratory Morbidity

Gil Gutvirtz^1^; Tamar Wainstock^2^; Eyal Sheiner^2^

*^1^Soroka University Medical Center, Metar, HaDarom; ^2^Department of Obstetrics and Gynecology, Soroka University Medical Center, Ben‐Gurion University of the Negev, Beer‐Sheva, Israel., Beer Sheva, HaDarom*

4:00 PM ‐ 6:00 PM

**Objective**: The increasing use of fertility treatments in the last decades has led to a significant rise in multiple pregnancies. While singletons born using fertility treatment were found to have an increased risk for perinatal and long‐term adverse outcomes as compared with spontaneous pregnancies, twin pregnancies achieved using fertility treatments were scarcely investigated. We investigated the long‐term respiratory morbidity of twins born following fertility treatments as compared with twins of spontaneous pregnancies

**Study Design**: A population‐based retrospective cohort study of twin deliveries born between the years 1991‐2021 at a tertiary center was conducted. Twins born using fertility treatments including ovulation induction (OI) and in‐*vitro* fertilization (IVF) were compared with twins of spontaneous pregnancies. Respiratory morbidity was collected from community clinics and hospitalizations of offspring up to the age of 18 years. A Kaplan–Meier survival curve was used to compare the cumulative incidence of respiratory morbidity and a Cox regression model was used to control for confounders

**Results**: A total of 7790 twins were included: 696 (8.9%) were born after OI and 1380 (17.7%) following IVF. The total respiratory morbidity rate was significantly higher in twins born following fertility treatments and specifically in IVF twins (*p*< 0.01; using the chi‐square test for trends), as compared with twins from spontaneous pregnancies (**Table**). Likewise, the cumulative incidence of respiratory morbidity was higher for twins of fertility treatments, with IVF showing the highest cumulative incidence (**Figure**). The Cox model, controlling for maternal and gestational age, hypertensive disorders, diabetes mellitus and ethnicity, found that IVF, but not OI, was an independent risk factor for long‐term respiratory morbidity of the twin offspring (adjusted HR (aHR) for IVF = 1.22, 95%CI 1.1‐1.3, *p*< 0.001 and aHR for OI = 1.04, 95%CI 0.9‐1.1, *p* = 0.585; compared with spontaneous twins)

**Conclusion**: IVF treatments resulting in twin pregnancies are independently associated with long‐term respiratory morbidity of the offspring

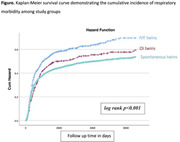

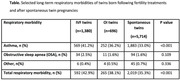

## 496 | Reproductive Planning in Kidney Disease: Survey of Patients with Chronic Kidney Disease and Preconception Counseling

Gwendolyn Lee^1^; Niloofar Nobakht^2^; Yaquelin A. Arevalo Iraheta^3^; Lorna Kwan^4^; Christina S. Han^4^

*^1^UCSF, San Francisco, CA; ^2^UCLA David Geffen School of Medicine, Los Angeles, CA; ^3^University of California, Los Angeles, Los Angeles, CA; ^4^UCLA, Los Angeles, CA*

4:00 PM ‐ 6:00 PM

**Objective**: Chronic kidney disease (CKD) is associated with greater rates of adverse maternal and fetal outcomes in pregnancy. Although preconception counseling (PCC) is recommended by the Centers for Disease Control and Prevention as primary prevention for pregnancy complications, the utilization and impact of PCC among patients with CKD is unknown.

**Study Design**: Patients assigned female sex at birth and aged 18‐45 years with CKD who received care at a nephrology clinic in an urban academic setting received a survey between January and May 2024. In this cross‐sectional analysis, data was analyzed using Chi‐square test or Fisher's exact test. Uni‐ and multivariable logistic regressions were performed to identify characteristics associated with inadequate PCC.

**Results**: Of 122 surveys distributed, 108 (88%) were completed. Overall, 74% of respondents received PCC, including 17% who received PCC from a maternal‐fetal medicine (MFM) provider. Respondents who received PCC from an MFM provider were significantly more likely to report discussing medication safety, maternal complications, and fetal complications, compared to those who received PCC without an MFM provider (Table 1). PCC recipients also reported greater concern about the effect of CKD on fertility and less knowledge about preconception and pregnancy with CKD. Respondents who reported a loss of libido were more likely to report PCC (OR 3.6, 95% CI 1.2‐10.5).

**Conclusion**: PCC for patients with CKD remains suboptimal, with 26% of respondents reporting no receipt of PCC. Our finding that topics discussed varied between MFM and non‐MFM providers suggest the opportunity for PCC standardization and equitable access to MFM providers. Furthermore, our finding that patients who did not receive PCC reported lower concern and greater knowledge than PCC recipients suggests false reassurance in the setting of lack of education and reaffirms the need for PCC.

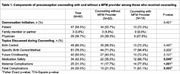

## 497 | Predicting Early Onset Preeclampsia (EOPE): Leveraging Demographics, Biophysical, and Biochemical Markers for Effective Aspirin Intervention

Hameeda A. Naimi^1^; Siddharth Hariharan^2^; Eric K. BRONI^3^; Shifa Turan^4^; Ozhan M. Turan^5^

*^1^Virginia Women's Center, Inc., Richmond, VA; ^2^University of Rochester, Rochester, NY; ^3^University of Pennsylvania Perelman School of Medicine, Philadelphia, PA; ^4^University of Maryland Medical System, Baltimore, MD; ^5^University of Maryland Medical Center, Baltimore, MD*

4:00 PM ‐ 6:00 PM

**Objective**: We hypothesized that combined screening using maternal demographics with biophysical and biochemical markers is a reliable method of predicting early onset preeclampsia (EOPE), requiring delivery prior to 34 weeks. Our secondary objective was to evaluate the effect of aspirin (ASA) on the performance of our prediction algorithm.

**Study Design**: This prospective cohort study assessed the risk of EOPE in pregnant patients between 11‐13 weeks of gestation using the EOPE risk calculator (published by the Fetal Medicine Foundation). Risk assessment included maternal demographics, biophysical data (BMI, blood pressure, uterine artery pulsatility index), and biochemical markers (PAPP‐A and PlGF). Patients were categorized as high risk (HR, ≥1:50) or low risk (LR, < 1:50). HR patients were advised to take 162mg of ASA, with compliance monitored via chart review. Logistic regression models considered factors such as history of preeclampsia, chronic hypertension, renal disease, and maternal age. The primary outcome was the development of EOPE, with secondary outcomes evaluating the impact of ASA on preventing preeclampsia.

**Results**: A total of 2,245 patients were screened, with 532 excluded (Figure 1). Among the remaining 1,713 patients, 141 (8.2%) were HR and 1,572 (91.8%) were LR. HR patients were more likely to be younger, Black, and have a history of hypertension and preeclampsia. HR individuals had a higher likelihood of developing EOPE (aOR: 4.38, 95% CI: 1.68‐11.42, p< 0.001) despite ASA recommendations. Among HR patients, 9 (6%) did not use ASA, and all developed preeclampsia, with 6 cases being EOPE. ASA significantly reduced development of EOPE (OR: 0.08, 95% CI: 0.008‐0.76, p = 0.03). Those using ASA had a higher median gestational age at delivery compared to non‐users (36 weeks vs. 33.6 weeks, p = 0.03) (Figure 2). 

**Conclusion**: Preeclampsia screening which combines maternal factors with biophysical and biochemical markers effectively identifies high risk individuals. ASA use in high risk individuals significantly reduces the risk of early‐onset disease and delays gestational age at delivery.

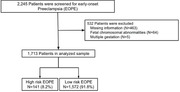

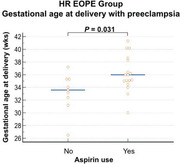

## 498 | Antenatal Fetal Surveillance: Impact on Pregnancy Outcomes

Hannah S. Foster^1^; Jessica Wu^1^; Markolline Forkpa^2^; Jesse Chittams^3^; Nehemiah Weldeab^3^; Jennifer Mccoy^1^; Nadav Schwartz^4^

*^1^Department of Obstetrics and Gynecology, University of Pennsylvania, Perelman School of Medicine, Philadelphia, PA; ^2^Pennsylvania Medicine Women's Health Clinical Research Center, Philadelphia, PA; ^3^University of Pennsylvania School of Nursing, Philadelphia, PA; ^4^University of Pennsylvania, Perelman School of Medicine, Philadelphia, PA*

4:00 PM ‐ 6:00 PM

**Objective**: Serial antenatal testing is often recommended for high‐risk pregnancies to reduce the stillbirth risk. However, the impact of testing recommendations on delivery outcomes is not well‐studied. We explored how fetal testing impacts delivery outcomes to better inform patients. 

**Study Design**: In this retrospective cohort, we identified all singleton pregnancies that delivered at 1 of 3 hospitals within an academic health system between 1/2022 and 5/2023. We then identified those that had undergone serial outpatient antenatal testing and collected testing indication and recommendations for the final testing visit prior to delivery. Primary outcomes included iatrogenic delivery < 39 weeks, iatrogenic preterm birth < 37 weeks (PTB), cesarean delivery (CS), and NICU admission. Stepwise regression models were used to assess the association between demographic variables and testing indications and outcomes.

**Results**: A total of 3992 patients were included, with 480 (12.1%) recommended for delivery based on their final test. Overall, 370 (9.3%) were sent for delivery < 39 weeks and 161 (4.0%) patients were sent for iatrogenic PTB. Testing patients sent for delivery had a 50.4% CS rate, which was significantly higher than the CS rate amongst those not sent for delivery due to a testing visit (37.4%, P< 0.001). Overall NICU admission rate was 12.6% (N = 501). Nulliparity was only associated with increased likelihood of CS (aOR: 1.2; 1.0‐1.4); whereas race was not associated with any of the outcomes. There were significant differences in NICU admission by hospital, likely related to NICU protocols. Several testing indications were significantly associated with each outcome (Table 1).

**Conclusion**: For patients undergoing antenatal testing, there is an overall low risk of iatrogenic preterm delivery or delivery prior to the 39‐week elective induction window. Certain testing indications are associated with increased risk of adverse pregnancy outcomes. These data can be used to help high‐risk patients understand their pregnancy risks when being recommended for serial fetal testing.

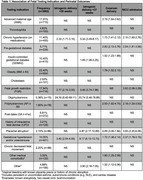

## 499 | The Risk of ICP Recurrence in the Subsequent Pregnancy: a Retrospective Analysis

Henri M. Rosenberg^1^; Minhazur R. Sarker^2^; Daniel L. Kuhr^3^; Sara E. Edwards^3^; Allison Perelman^4^; Leslie J. Warren^4^; Gladys (Sandy) A. Ramos^2^; Angela T. Bianco^4^; Lauren A. Ferrara^4^; Chelsea A. DeBolt^4^

*^1^Icahn School of Medicine at Mount Sinai, Division of Maternal‐Fetal Medicine, Icahn School of Medicine at Mount Sinai, NY; ^2^University of California, San Diego, San Diego, CA; ^3^Division of Maternal‐Fetal Medicine, Icahn School of Medicine at Mount Sinai, New York, NY; ^4^Icahn School of Medicine at Mount Sinai, New York, NY*

4:00 PM ‐ 6:00 PM

**Objective**: The recurrence risk of intrahepatic cholestasis of pregnancy (ICP) has been reported to be between 40‐90% based on small studies with homogeneous populations. Our aim was to determine the recurrence risk of ICP and identify variables associated with recurrence in a larger, more diverse population.

**Study Design**: Retrospective cohort study of singleton, non‐anomalous gestations complicated by ICP at a single academic center between 2015‐2024. The EMR was used to identify patients with a first delivery complicated by ICP (initial) and a subsequent pregnancy with or without ICP (subsequent), both delivering at the same institution. The primary outcome was the recurrence of ICP in the subsequent pregnancy; secondary outcomes include identifying variables associated with ICP recurrence. Univariable and multivariable logistic regression were used to calculate the odds ratio and 95% confidence interval (OR [95% CI]) adjusting for advanced maternal age, history of hepatobiliary disease, and gestational diabetes.

**Results**: A total of 104 patients with ICP were identified, 46 (44%) of whom developed ICP in the subsequent pregnancy [Table 1]. In the group that had recurrent ICP, more patients self‐identified as Hispanic (84.8% vs. 65.5%), but there were no differences in age or comorbidities in the initial pregnancy. The recurrent group had more patients with peak total bile acids (TBA) >40 (31. % vs. 7.6%, p = 0.01) and alkaline phosphatase (ALP) twice the upper limit of normal (37.0% vs. 19.0%, p = 0.04) in the initial pregnancy [Table 1]. In a multivariable regression, index pregnancy peak TBA >40 was associated with recurrence (aOR 6.03 Cl 1.81‐20.07) and short‐interval pregnancy was associated with decreased recurrence (aOR 0.40 Cl 0.17‐0.96) [Table 2].

**Conclusion**: In this ICP cohort, the risk of recurrence in the subsequent pregnancy was 44%. Only peak TBA >40 in the initial pregnancy was associated with recurrence.  Given the wide range of reported ICP recurrence rates in the literature, larger studies are warranted to improve pre‐conception counseling.

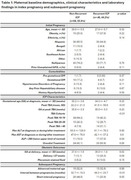

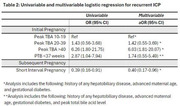

## 500 | Predictors of Pregnancy Duration and Gestational Age at Delivery after Laser Photocoagulation for Twin‐Twin‐Transfusion Syndrome

Henry L. Galan^1^; Michael V. Zaretsky^2^; Stephen P. Emery^3^; Zhaoxing Pan^4^; Nicholas Behrendt^4^; Sarkis C. Derderian^2^; Anthony Johnson^5^; Greg Ryan^6^; William H. Goodnight^7^; On behalf of the NAFTNet

*^1^University of Colorado, School of Medicine, Children's Hospital of Colorado, Aurora, CO; ^2^University of Colorado, School of Medicine, Aurora, CO; ^3^University of Pittsburgh, Pittsburgh, PA; ^4^University of Colorado, Aurora, CO; ^5^McGovern Medical School University of Texas Health Science Center at Houston (UThealth), Houston, TX; ^6^University of Toronto, Toronto, ON; ^7^University of North Carolina at Chapel Hill, Chapel Hill, NC*

4:00 PM ‐ 6:00 PM

**Objective**: Limited data exist regarding the impact of perioperative factors on pregnancy duration after fetoscopic laser photocoagulation (FLP) for twin‐twin transfusion syndrome (TTTS). The objective of this study was to determine the best predictors of duration of pregnancy (latency) and gestational age (GA) at delivery following FLP for TTTS.

**Study Design**: TTTS cases treated with FLP between 2001‐2023 were identified through the North American Fetal Therapy Network monochorionic twin registry containing perioperative variable and pregnancy outcomes. Higher order multiples, fetal anomalies and karyotypic abnormalities were excluded. Multiple perioperative variables were analyzed to determine the best predictors of latency and GA at delivery. Kaplan‐Meier and COX regression were used in univariate analysis to identify significant factors that then had interplay evaluation with multiple Cox regression to determine the best predictors.

**Results**: Of 2317 TTTS cases treated with FLP, 1740 met inclusion criteria with sufficient information for analysis. Shown as Mean ± S.D, the GA at time of laser was 20.7±2.7 wks, the procedure time was 52.5±25.5 min, the latency period was 10.2±5.3 wks and GA at delivery was 30.8±4.9 wks. While univariate analysis found multiple factors significantly associated with both reduced latency (Table) and earlier delivery GA, Cox regression analysis (N = 865) showed the best predictors to be cervical length (CL) of < 25mm, amnioinfusion volume, laser time and earlier GA at time of FLP (Table). Early GA at time of surgery carried longer latency to delivery (Figure) but resulted in earlier delivery. The longer latency with early surgery persists regardless of whether CL is < or ≥ 25mm (Figure). The hazard to early delivery GA decreased by 4.3% if surgery occurs a week later as opposed to a week earlier.

**Conclusion**: From this large registry, the best predictors of both latency and GA at delivery after FLP for TTTS are the GA at time of the procedure, laser time, a short cervix and volume of amnioinfusion during FLP.  These are factors to consider in the management of TTTS.

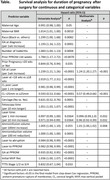

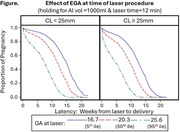

## 501 | Prevention of Preterm Birth in Twin Pregnancies: International Delphi Consensus

Hiba J. Mustafa^1^; Jameela Sheikh^2^; Vincenzo Berghella^3^; William A. Grobman^4^; Alireza A. Shamshirsaz^5^; Sanne J. Gordijn^6^; Wessel Ganzevoort^7^; Amanda Roman^8^; Asma Khalil^9^; On behalf of the PTB in the Twins Working Group

*^1^Indiana University and Riley Children's Hospital, Indianapolis, IN; ^2^Guy's and St Thomas’ Hospital NHS Foundation Trust, London, England; ^3^Sidney Kimmel Medical College of Thomas Jefferson University, Philadelphia, PA; ^4^The Ohio State University, Columbus, OH; ^5^Fetal Surgeon Chief, Division of Fetal Medicine and Surgery Director of the Maternal Fetal Care Center Director of the Perinatal Surgery Fellowship Professor of Surgery, Boston Children's Hospital Harvard Medical School, Boston, MA; ^6^University Medical Center Groningen, Groningen, Groningen; ^7^Amsterdam University Medical Centers, Amsterdam, Groningen; ^8^Department of Maternal‐Fetal Medicine, Thomas Jefferson University Hospital, Philadelphia, PA; ^9^Fetal Medicine Unit, St George's Hospital, St George's University of London, London, England*

4:00 PM ‐ 6:00 PM

**Objective**: To use a Delphi process to gain insight into approaches to the management of preterm birth (PTB) in twin pregnancies, including those with twin‐to‐twin transfusion (TTTS).

**Study Design**: A three‐round Delphi procedure was conducted among an international panel of experts to assess their approach to PTB prevention, monitoring, and management strategies in twins. Experts were chosen based on expertise, relevant publications, or affiliations. Response options included multiple‐choice answers or a 5‐point Likert scale. A priori, a cut‐off of ≥70% was used to define “consensus”.

**Results**: 115 experts joined the first round and 94/115 (82%) completed the subsequent rounds. There was representation from 22 countries on five continents. At least 70% performed routine screening of cervical length (CL) via transvaginal (TV) scan between 18 and 23 weeks with CL≤25 mm to diagnose a short cervix in twin pregnancies. In twin pregnancies with short CL, most experts offered vaginal progesterone and not pessary or expectant management. There was significant agreement but no consensus to offer cerclage for CL≤10. In twin pregnancies with asymptomatic dilated cervix, consensus was reached for cerclage placement as well as adjunctive practices surrounding the cerclage placement procedure. Similarly, at least 70% of experts agreed that serial TV CL assessment was warranted in those who had a current singleton pregnancy with a previous twin pregnancy requiring physical exam‐indicated cerclage. In those with TTTS, a laser procedure generally was offered by most regardless of whether the cervix was short or dilated. For those with TTTS and a short CL, most experts would recommend cerclage or progesterone but not pessary or expectant management. However, no consensus was reached on PTB prevention measures in TTTS with cervical dilation.  

**Conclusion**: This Delphi shows the practice variations among obstetric providers worldwide in evaluations and management of PTB in twin pregnancies, which often differs from what is recommended by national and international societies.

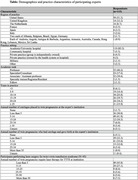

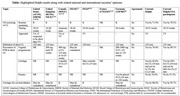

## 502 | Elevated Levels of Circulating Endothelial Cells in Pregnancies Complicated by Preeclampsia And/OR Fetal Growth Restriction

C Helmicki^1^; J Ladd^1^; A Heck^1^; J Wu^2^; A Coffer^1^; M Hart^1^; H Gammill^1^; J Vanderhoeven^2^; S Peterson^2^; A Ramirez^1^; E Kaldjian^1^

*^1^RareCyte, Inc., Seattle, WA; ^2^Swedish Medical Center, Seattle, WA*

4:00 PM ‐ 6:00 PM

**Objective**: To develop a circulating endothelial cell (CEC) assay to measure maternal blood CEC numbers in pregnancy and begin exploratory studies comparing pregnancies complicated by preeclampsia (PE) and fetal growth restriction (FGR). 

**Study Design**: We used model human umbilical vein endothelial cells (HUVECs) to develop a 4‐parameter immunofluorescence assay (IF) on the established RareCyte liquid biopsy platform with the following markers: Sytox (nucleus), VE‐Cadherin (CD144, positive CEC marker #1), MCAM (CD146, positive CEC marker #2), and CD45 (white blood cell exclusionary marker). CECs were defined as nucleated cells, negative for CD45, and positive for either or both CD144 and CD146. Using the HUVEC spike‐in model system, we analytically validated assay performance by evaluating specificity, sensitivity and limit of detection across clinically relevant CEC count ranges. In clinical studies, CEC numbers in healthy, non‐pregnant female subjects of child‐bearing age (20‐40 years old; N = 10) were compared to third trimester pregnancy samples (N = 17 total, including N = 9 uncomplicated, N = 8 FGR, N = 5 PE, N = 3 FGR + PE).  All pregnant subjects were enrolled after informed consented at Swedish Medical Center, Seattle, WA.

**Results**: Analytic validation studies demonstrated sensitivity and specificity of CD144 and CD146 of 99–100% and lower limit of detection of 1 cell in >1 million.  CEC count was higher in pregnancy compared to non‐pregnant reproductive age subjects and was higher in pregnancies with PE +/‐ FGR compared to those with uncomplicated outcomes.  Results are shown in Figure 1A and B.

**Conclusion**: We have developed a robust assay for enumeration of CECs in pregnancy.  Elevated levels of CECs in third trimester pregnant patients were observed in both PE and FGR relative to healthy third trimester pregnancies.  These preliminary studies support further investigation of CEC enumeration as a predictive biomarker of pregnancy complications.

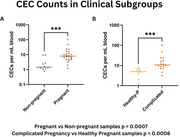

## 503 | Project PEN‐FAST: Unmasking Allergies for Antibiotic Optimization

Hope Brandon^1^; Noelle Leung^2^

*^1^University of Kentucky College of Pharmacy, Lexington, KY; ^2^University of Kentucky HealthCare, Lexington, KY*

4:00 PM ‐ 6:00 PM

**Objective**: Assess the clinical impact of the PEN‐FAST scoring tool within the obstetric group at a single academic medical center.

**Study Design**: This was a single center, retrospective, observational analysis of pregnant patients with a documented penicillin or cephalosporin allergy admitted to the birthing center between January and June 2023 who also received an antibiotic and/or had a PEN‐FAST assessment completed.

**Results**: Sixty‐four patients were included in the final analysis. Within the analyzed cohort, 53 (83%) of patients had a PEN‐FAST assessment completed, 17 (32%) of whom had their penicillin allergy successfully de‐labeled. Among patients with a penicillin allergy that was not de‐labeled, 11 (31%) had a score or assessment indicating that a cephalosporin could be safely utilized, nine (25%) had a score of three or greater, indicating a non‐beta lactam antibiotic should be employed, and seven (19%) patients declined de‐labeling despite a PEN‐FAST assessment indicating a low‐risk penicillin allergy, and the remaining nine (25%) were either referred for further workup or not de‐labeled due to other reasons. Of the patients who had a PEN‐FAST assessment completed, 38 (72%) received a penicillin or cephalosporin and 15 (28%) received alternate or no antibiotics. Comparatively, of the 11 patients who did not have a PEN‐FAST assessment completed, two (18%) received a penicillin or cephalosporin and nine (82%) received an alternate antibiotic. Among all patients analyzed, there were no identified adverse reactions to penicillins or cephalosporins.

**Conclusion**: Within our cohort, 32% of patients had their penicillin allergy successfully de‐labeled using the PEN‐FAST assessment. Even without de‐labeling due to factors such as patient preference, utilization of the PEN‐FAST assessment resulted in a dramatic increase in the use of pencillins and cephalosporins as opposed to alternate antibiotics. Overall, the PEN‐FAST assessment was an efficient and safe tool for stratifying risk of penicillin allergies within this cohort of patients and did impact antibiotic choice for the patients included.

## 504 | The Perinatal Effects of a Mindfulness Phone App ‐ a Pilot Randomized Controlled Trial

Noa Gilad^1^; Swati Agrawal^2^; Klaudia Szczech^3^; Alexandra Berezowsky^1^; Amir Naeh^4^; Howard Berger^5^

*^1^University of Toronto, Toronto, ON; ^2^McMaster University, Hamilton, ON; ^3^Saint Michael's Hospital, Toronto, ON; ^4^Hillel Yaffe Medical Center, Hillel Yaffe Medical Center, HaMerkaz; ^5^St. Michael's Hospital, University of Toronto, OR*

4:00 PM ‐ 6:00 PM

**Objective**: Sleep disturbances are commonly reported during pregnancy, are difficult to treat and are associated with adverse perinatal outcomes. We aimed to assess the feasibility of the use of a mobile mindfulness application (App) in pregnant people with self‐reported sleep disturbance to improve sleep quality and perinatal outcomes.

**Study Design**: Pilot randomized controlled trial (RCT). Pregnant people with a singleton pregnancy, between 20‐30 weeks gestation and self‐reporting sleep disturbances were assessed for recruitment. Participants were randomized to either the use of a standard pregnancy sleep leaflet or to the addition of funded access to a commercially available mindfulness App.  Both groups received and were instructed to use a sleep actigraph. The co‐primary outcomes were: 1) recruitment rate and quantitative use of the mindfulness app. 2) Sleep quality actigraph measures and the change in PSQI score. Perinatal outcomes were collected and reported.  

**Results**: Of 1576 potential participants,629 (39.9%) declined, 706 were not assessed and 113 were excluded leaving 128 randomized participants, 64 to each group. Baseline characteristics did not differ between groups. In the mindfulness group 35/64 (54.7%) used the App for an average of 9.9 (0.02–90) minutes per day and a mean of 8 (1‐47) sessions per pregnancy. Actigraph use was similar with 30/64 (46.9%) and 32/64 (50%) providing data in the mindfulness and control groups respectively.  No significant reduction was found in PSQI scores, actigraph measures or measures of stress, anxiety or depression (Table 1). Perinatal outcomes did not differ between groups except for the rate of post‐partum hemorrhage which was increased in the intervention group (11.9% vs 1.6%, P = 0.03). 

**Conclusion**: Performing a RCT using a mindfulness App is feasible, but recruitment and usage rates were lower than expected. Although exploratory, the use of a mindfulness App did not appear to improve sleep parameters when compared to routine sleep education.

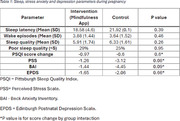

## 505 | The Association Between Hepatitis C Virus Infection During Pregnancy and Risk of Infant Death

Ilish Gedestad^1^; Kriti N. Vedhanayagam^2^; Sergio Karageuzian^3^; Stephen Contag^4^; Synia Chunn^2^; Megan Marquez^5^; Rang Kim^5^; Ruofan Yao^2^

*^1^Loma Linda University School of Medicine, Redlands, CA; ^2^Loma Linda University School of Medicine, Loma Linda, CA; ^3^Loma Linda University School of Medicine, Pasadena, CA; ^4^University of Minnesota, Minneapolis, MN; ^5^Loma Linda University School of Medicine, Loma Linda University, CA*

4:00 PM ‐ 6:00 PM

**Objective**: To investigate the association between Hepatitis C Virus (HCV) status and the likelihood of infant death.

**Study Design**: This retrospective cohort study used data from the National Center for Health Statistics Vital Statistics database. The cohort linked birth and infant death data files from 2015 to 2021 that were used for this study. The study included all births in the United States during the study period with known HCV status. The outcome of interest was infant death. Univariate analysis was performed to determine the association between HCV and infant death. Multivariate logistic regression was then performed to estimate the effect of HCV status on infant death adjusting for potential confounding variables. Interaction analysis was then performed to determine if the effect of HCV on infant death is modified by race and obesity.

**Results**: Of the 26,456,956 women, 122,485 (0.46%) were identified with HCV infection. HCV infection was associated with high rate of infant mortality (1.0% vs 0.5%, p< 0.001; aOR 2.34 [2.21‐2.47]). Compared to White, both Black and Asian race demonstrated a protective effect on infant mortality among individuals with HBV infection (aOR 0.55; 95% CI [0.39 ‐ 0.77] and aOR 0.54 95% CI [0.37‐0.78]). Compared to underweight group, individuals with class III obesity also had lower infant death (aOR 0.48; 95% CI [0.28‐0.82]).

**Conclusion**: This study demonstrates that HCV infection is associated with a significant increase in the risk of infant death, which is modified by other risk factors.

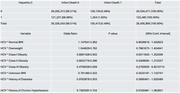

## 506 | The Association Between Hepatitis B Virus Infection During Pregnancy and Risk of Infant Death

Ilish Gedestad^1^; Sergio Karageuzian^2^; Kriti N. Vedhanayagam^3^; Stephen Contag^4^; Synia Chunn^3^; Megan Marquez^5^; Rang Kim^5^; Ruofan Yao^3^

*^1^Loma Linda University School of Medicine, Redlands, CA; ^2^Loma Linda University School of Medicine, Pasadena, CA; ^3^Loma Linda University School of Medicine, Loma Linda, CA; ^4^University of Minnesota, Minneapolis, MN; ^5^Loma Linda University School of Medicine, Loma Linda University, CA*

4:00 PM ‐ 6:00 PM

**Objective**: To investigate the association between Hepatitis B Virus (HBV) status and the likelihood of infant death.

**Study Design**: This retrospective cohort study used data from the National Center for Health Statistics Vital Statistics database. The cohort linked birth and infant death data files from 2015 to 2021 that were used for this study. The study included all births in the United States during the study period with known HBV status. The outcome of interest was infant death. Univariate analysis was performed to determine the association between HBV and infant death. Multivariate logistic regression was then performed to estimate the effect of HBV status on infant death adjusting for potential confounding variables. Interaction analysis was then performed to determine if the effect of HBV on infant death is modified by race and obesity.

**Results**: Of the 26,456,956 women, 56,299 (0.21%) were identified with HBV infection. HBV infection was associated with higher but not statically significant rate of infant mortality (0.52% vs 0.49%, p = 0.757; aOR 0.98 [0.87‐1.10]). Compared to White, both Black and Asian races demonstrated a protective effect on infant mortality among individuals with HBV infection (aOR 0.55; 95% CI [0.39 ‐ 0.77] and aOR 0.54; 95% CI [0.37 ‐ 0.78]).

**Conclusion**: HBV infection is not significantly associated with an increased risk of infant death, however other risk factors may modify this outcome.

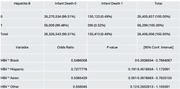

## 507 | The Use of a Novel Point‐Of‐Care (POC) IgM Test for the Diagnosis of Congenital Syphilis

Irene A. Stafford^1^; Sabrina DaCosta^2^; Kaitlyn Stark^3^; Diana Villarreal^4^; Jeffrey K. Klausner^5^; Leandro Mena^6^; Gary Lehnus^7^; Sean C. Blackwell^1^

*^1^McGovern Medical School at UTHealth Houston, Houston, TX; ^2^Division of Maternal‐Fetal Medicine, Department of Obstetrics, Gynecology and Reproductive Sciences, McGovern Medical School at UTHealth Houston, Houston, TX; ^3^University of Texas Health Science Center at Houston ‐ McGovern Medical School, Houston, TX; ^4^The University of Southern California, Los Angeles, CA; ^5^University of Southern California, Los Angeles, CA; ^6^All‐In Health Solutions LLC, Atlanta, GA; ^7^Lehnus & Associates Consulting, East Stroudsburg, PA*

4:00 PM ‐ 6:00 PM

**Objective**: The only hematologic test used for congenital syphilis (CS) diagnosis is the neonatal nontreponemal (NT) serologic combined antibody test directed towards *T. pallidum* antigens. This includes IgG antibodies which cross the placenta and may reflect maternal antibodies rather than neonatal infection. In contrast, any syphilis‐specific IgM immunoglobulins detected in the neonate are fetal in origin and may reflect CS. This pilot study aims to determine the test performance of a POC IgM test for the diagnosis of CS.

**Study Design**:  

After institutional review board approval, serum from 2 controls and 23 pregnant patients with syphilis and their newborns was collected between 05/24‐06/24. Duplicate 20 µL aliquots were tested using the research‐use only rapid lateral flow test developed by Diagnostics Direct, LLC for detection of syphilis IgM. This test uses a purified specific anti‐IgM polyclonal antibody coupled with colloidal gold in the sample pad to form rose colored visually read test and control lines.  A proprietary Protein is used to bind all IgG from reacting with the antigen test line, producing only IgM for visual results detection. Duplicate 50 µL aliquots from the samples were tested for IgM using two CE‐marked Western Blot (WB) tests with different treponema targets as comparators (ViraBlot, Germany and Euroimun, Germany). Mother‐baby dyads were staged for syphilis according to the Centers for Disease Control and Prevention guidelines and were also considered for test performance analytics.

**Results**: The positive and negative percent agreement (PPA, NPA) between the POC IgM test and the Virablot WB was 86% and 78% and for Euroimun, PPA and NPA were 80% and 86% respectively (Table 1). Clinical correlates were similar, demonstrating a 90% PPA and a 93.3% NPV between the POC IgM test and syphilis diagnosis for dyads (Table 2).

**Conclusion**: The syphilis POC IgM test demonstrated excellent test performance when compared to reference WB tests and clinical correlates. Given poor sensitivity of current lab tests for CS, further studies evaluating this POC IgM test for at‐risk neonates are warranted.

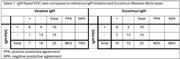

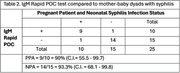

## 508 | Sibling Analysis of Long‐Term Morbidities in Preterm Twins: Vaginal versus Cesarean Delivery

Itamar Ben Shitrit^1^; Eyal Sheiner^2^; Gali Pariente^2^; Ruslan Sergienko^3^; Tamar Wainstock^2^

*^1^Soroka University Medical Center/ Ben Gurion University, Beer Sheva, HaDarom; ^2^Department of Obstetrics and Gynecology, Soroka University Medical Center, Ben‐Gurion University of the Negev, Beer‐Sheva, Israel., Beer Sheva, HaDarom; ^3^Ben‐Gurion University of the Negev, Beer Sheva, HaDarom*

4:00 PM ‐ 6:00 PM

**Objective**: Given the gap in knowledge regarding mode of delivery's impact on the premature twin population, we aimed to investigate the association between mode of delivery and long‐term respiratory, neurologic, infectious, and gastrointestinal (GI) morbidities in preterm twin siblings, with the first delivered vaginally (VD) and the second by cesarean delivery (CD).

**Study Design**: A retrospective population‐based cohort study was conducted at a tertiary medical center between 1991‐2021. The study compared preterm twins where the first‐born twin was vaginally delivered, and the second‐born twin was delivered via CD, excluding cases with congenital malformations or perinatal deaths. Follow‐up ended at the age of 18, end of the study period, or diagnoses of any morbidity from the studied outcomes. Kaplan‐Meier survival curves were used to compare cumulative incidences, and Cox proportional hazards models were applied to adjust for potential confounders.

**Results**: The cohort included 71 offspring born via CD and 71 via VD, representing 3.5% of all preterm twin births at this center. No difference in the cumulative incidence of respiratory, neurologic, infectious, and gastrointestinal morbidity was noted between the groups (respiratory: 24% VD vs. 23% CD, p = 0.85; neurologic: 12% VD vs. 12% CD, p = 1.00; infectious: 52% VD vs. 45% CD, p = 0.20; GI: 20% VD vs. 17% CD, p = 0.56, Table, Figure). Similarly, the Cox model, adjusted for maternal age, ethnicity, gestational age group, repeated pregnancies, offspring's birth year, gestational diabetes mellitus, preeclampsia, weight group, and clustering within pregnancy, showed no association between delivery mode and long‐term morbidity (respiratory: aHR 0.93, 95% CI 0.56–1.55; neurologic: aHR 1.16, 95% CI 0.47–2.27; infectious: aHR 0.81, 95% CI 0.59–1.12; GI: aHR 0.86, 95% CI 0.47–1.59, Table).

**Conclusion**: No significant differences in long‐term respiratory, neurologic, infectious, and GI morbidities exist between preterm twin siblings when the first‐born was delivered vaginally and the second‐born by CD. Further research on larger cohorts is needed to confirm these findings.

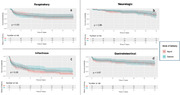

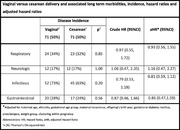

## 509 | Elective Inductions of Labor at 39 Weeks versus Expectant Management in Multiparous Patients

James D. Doss^1^; Elise Requadt^2^; Claire Novack^2^; Anupama Balasubramanian^3^; Carly Duncan^4^; Dong Wang^5^; Antonina I. Frolova^6^; Jeannie C. Kelly^7^; Nandini Raghuraman^7^

*^1^Washington University In St. Louis, St. Louis, MO; ^2^Washington University at St. Louis, St. Louis, MO; ^3^Larner College of Medicine at the University of Vermont, Larner College of Medicine at the University of Vermont, VT; ^4^University of Alabama at Birmingham, Birmingham, AL; ^5^Washington University at St. Louis, Department of Ob/Gyn, St. Louis, MO; ^6^Washington University School of Medicine, St. Louis, MO; ^7^Washington University School of Medicine in St. Louis, St. Louis, MO*

4:00 PM ‐ 6:00 PM

**Objective**: The ARRIVE trial demonstrated a reduction in cesarean section with similar perinatal outcomes in low risk nulliparas undergoing induction of labor (IOL) compared to expectant management (EM). We compared outcomes in low‐risk multiparous patients undergoing IOL vs EM. 

**Study Design**: We performed a retrospective cohort study of multiparous patients with low‐risk singleton pregnancies at 39 weeks gestation from 2020‐2022. All multiparous patients who delivered after 39 weeks were included, and patients undergoing IOL for medical or fetal indications were excluded. The primary outcome was composite neonatal morbidity. The secondary outcomes were cesarean section, postpartum hemorrhage (defined as estimated blood loss ≥1000 ml), and chorioamnionitis. These outcomes were compared between patients undergoing scheduled IOL between 39w0d and 39w6d and those undergoing expectant management at 39 weeks. Multivariable Firth's penalized logistic regression was used to adjust for potential confounders.  

**Results**: Of the 841 multiparous patients during the study period, 454 (54%) underwent IOL and 387 (46%) were EM. Among EM, 54 (14%) ultimately underwent an IOL. There were no significant differences in odds of having composite neonatal morbidity (aOR 1.04, 95% CI 0.63‐1.71, p = 0.88), cesarean delivery (aOR 2.09, 95% CI 0.9‐4.79, p = 0.09), or chorioamnionitis (aOR 0.58, 95% CI 0.13‐2.57, p = 0.48) in IOL group as compared to EM group. The odds of having postpartum hemorrhage were 2.6 times higher in IOL as compared to EM patients (aOR 2.59, 95% CI 1.24‐5.42, p = 0.011.) 

**Conclusion**: Multiparous patients undergoing elective IOL have similar odds of cesarean section and neonatal morbidity as those undergoing EM. IOL is associated with an increased risk of hemorrhage compared to EM.  

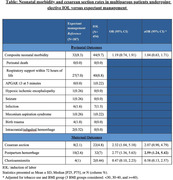

## 510 | Dysglycemia Phenotypes in Pregnancy and the Risk of Fetal Shoulder Dystocia at Birth

Jasmine Hitt^1^; Eric K. BRONI^2^; Maria Som^3^; Cassandra Seifert^3^; May Blanchard^1^

*^1^University of Maryland Department of OBGYN, Baltimore, MD; ^2^University of Pennsylvania Perelman School of Medicine, Philadelphia, PA; ^3^University of Maryland School of Medicine, Baltimore, MD*

4:00 PM ‐ 6:00 PM

**Objective**: Maternal diabetes is a well‐established risk factor for fetal shoulder dystocia (ShD). However, data on the differential risk of ShD among women with dysglycemia subtypes in pregnancy are scarce. We evaluated whether the risk of fetal ShD differs by maternal dysglycemia subtype.

**Study Design**: We investigated 202 mothers who delivered infants with ShD and 747 randomly selected controls from a cohort of women who received antenatal care at an urban, tertiary medical center from 2012‐ 2023. Maternal dysglycemia phenotypes include [Type 1 diabetes (T1D), Type 2 diabetes (T2D), Gestational diabetes controlled by diet and exercise (GDMA1), Gestational diabetes requiring hypoglycemic agents (GDMA2)], Elevated 1‐hour GCT without 3‐hour GTT (E1GCT) and Elevated 1‐hour GCT with normal 3‐hour GTT (E1N3GTT). We used adjusted logistic regression models to estimate the risk of infant ShD among women in the different dysglycemia subgroups.

**Results**: Pregnant women who had infants with ShD were younger (27 vs 31 years, p< 0.001), more likely to be Black (72.8% vs 46.5%), delivered at later gestational age (39.3 vs 38.4 wks, p< 0.001), were more likely to have vaginal delivery (96% vs 53%, p< 0.001) and a history of smoking (17.3% vs 7.4%) **Table 1**. Compared to pregnant women with normoglycemia, those with E1GCT had a 4.4‐fold [aOR, 4.35 (1.23–15.45)] higher risk of delivering an infant with ShD, even after controlling for maternal age, gestational age at delivery, race/ethnicity, parity, mode of delivery, prior delivery with ShD. Similarly, those with T2D had a 2.6‐fold [aOR, 2.62 (1.19–5.75)] higher odds of delivering an infant with ShD. Additionally, women with E1N3GTT had a 3.4‐fold [aOR, 3.36 (1.33‐8.44)] higher odds of delivering an infant with ShD. **Table 2**.

**Conclusion**: Different dysglycemia phenotypes in pregnancy are independently associated with a higher risk of shoulder dystocia at birth. Continuous surveillance of pregnant women with dysglycemia, especially those with T2D and elevated 1‐hour GCT (± 3‐hour GTT), will reduce the risk of fetal shoulder dystocia and concomitant perinatal complications.

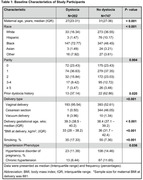

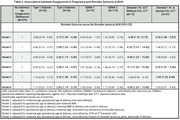

## 511 | Impact of Weight Discordance on Short‐term Postnatal and Neurodevelopmental Outcomes in Triplet Pregnancies

Jeesun Lee^1^; Joon Hyung Lee^2^; JI HOI KIM^3^; Hee Young Cho^4^; Seung Mi Lee^1^; Chan‐Wook Park^1^; Joong Shin Park^1^; Jong Kwan Jun^5^

*^1^Seoul National University College of Medicine, Seoul, Seoul‐t'ukpyolsi; ^2^The Nature Obstetrics and Gynecology, Pyeongtaek, Kyonggi‐do; ^3^Seoul National University Hospital College of Medicine, Seoul, Seoul‐t'ukpyolsi; ^4^Seoul National University, Seoul, Seoul‐t'ukpyolsi; ^5^Ewha Womans University College of Medicine, Seoul, Seoul‐t'ukpyolsi*

4:00 PM ‐ 6:00 PM

**Objective**: Weight discordance (WD) in multifetal pregnancies is a key concern due to its association with selective fetal growth restriction (sFGR). Although much research has focused on twins, studies on triplets are limited. This study evaluates the impact of WD on short‐term postnatal and neurodevelopmental outcomes in triplets.

**Study Design**: We analyzed triplets delivered at Seoul National University Hospital from November 1997 to December 2022. WD was calculated as: ((birthweight of the largest fetus–birthweight of the smallest fetus) × 100) / birthweight of the largest fetus. Short‐term outcomes included neonatal death, respiratory distress syndrome, bronchopulmonary dysplasia, persistent pulmonary hypertension, apnea of prematurity, intraventricular hemorrhage, periventricular leukomalacia, retinopathy of prematurity, necrotizing enterocolitis, and early‐onset sepsis. Neurodevelopmental outcomes were assessed up to 60 months using the Korean Ages and Stages Questionnaires and the Bayley Scales of Infant and Toddler Development, 3rd Edition. ROC curves and Generalized Estimating Equations (GEE) were used to analyze the predictive value.

**Results**: A total of 436 triplets were analyzed. Short‐term outcomes were linked to gestational age but not to WD or birthweight order. ROC curve analysis showed an AUC of 0.550 for short‐term outcomes, indicating limited predictive value. GEE analysis found adjusted hazard ratios of 1.08 (95% CI 0.74‐1.59) for the middle‐sized fetus and 1.48 (95% CI 0.99‐2.22) for the smallest fetus, suggesting minimal impact of birthweight order. Neurodevelopmental outcomes also showed no significant correlation with WD, with an AUC of 0.645. No differences in outcomes based on WD were seen when analyzed by chorionicity.

**Conclusion**: This study found no strong association between WD and short‐ or long‐term complications in liveborn triplets. WD does not appear to be a significant predictor of adverse outcomes in triplet pregnancies, though further research is needed for a thorough understanding.

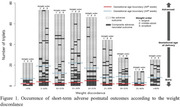

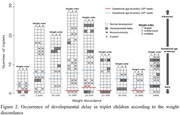

## 512 | Azithromycin to Improve Latency in Exam‐Indicated Cerclage: a Multicenter Randomized Control Trial (ALEC)

Jenani S. Jayakumaran^1^; Kavisha Khanuja^2^; Stephanie A. Fisher^3^; Emily S. Miller^4^; Emily B. Rosenfeld^5^; Justin S. Brandt^6^; Megan Piacquadio^7^; Adeeb Kalifeh^8^; Rupsa C. Boelig^9^

*^1^Virtua Medical Group, Moorestown, NJ; ^2^Sidney Kimmel Medical College at Thomas Jefferson University, Philadelphia, PA; ^3^Northwestern University Feinberg School of Medicine, Chicago, IL; ^4^Women & Infants Hospital of Rhode Island and Alpert Medical School of Brown University, Providence, RI; ^5^Rutgers Robert Wood Johnson Medical School, New Brunswick, NJ; ^6^NYU Langone Health, New York, NY; ^7^University of Miami, Miami, FL; ^8^Jefferson Einstein Philadelphia, Philadelphia, PA; ^9^Sidney Kimmel Medical College, Thomas Jefferson University, Philadelphia, PA*

4:00 PM ‐ 6:00 PM

**Objective**: While prophylactic azithromycin has been shown to increase latency in the setting of cervical shortening, its use in physical exam‐indicated cerclage (PEIC) has been understudied. Our objective is to determine whether adding azithromycin prior to PEIC increases gestational latency to usual care (cefazolin and indomethacin).

**Study Design**: This RCT included pregnant people with singleton gestations < 24 weeks gestational age (GA) planning a PEIC at one of four sites across the United States. Participants were randomized 1:1 to usual care (cefazolin 1‐2 g IV and indomethacin 50 mg IV preoperatively, then 2 additional doses at 8 and 16 hours postoperatively) or usual care plus azithromycin (azithromycin 1g IV once perioperatively). Primary outcome was gestational latency (days) from cerclage placement to delivery. Secondary outcomes included preterm birth (< 37, < 34, < 32, < 28, and < 24 weeks), GA at birth, chorioamnionitis, birthweight, NICU admission, and composite neonatal morbidity and mortality. Fifty participants needed to be randomized to demonstrate a 20±25 days improvement in latency with 80% power. Assuming a 10% loss to follow‐up, we aimed to recruit 55 individuals. Kaplan‐Meier survival curve, Mann Whitney U test, and chi squared analyses were conducted. The study was registered at clinicaltrials.gov (NCT05132829).

**Results**: A total of 55 participants were randomized from December 2021 to September 2023; 27 to usual care, 27 to adjunctive azithromycin, and 1 excluded from analysis when found not to meet study criteria after randomization. Protocol adherence was 100% for each arm. The median gestational latency after PEIC did not differ between the usual care and azithromycin groups (median difference ‐1 day, 95% CI ‐20 to 26 days; Figure). Additional secondary obstetric and neonatal outcomes were similar between groups (Table).

**Conclusion**: A single perioperative dose of azithromycin in addition to usual care (cefazolin and indomethacin) does not improve latency to delivery or other obstetric or neonatal outcomes for individuals undergoing PEIC.

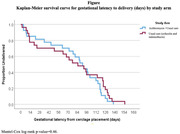

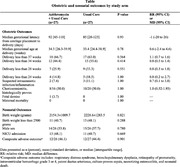

## 513 | Characteristics Associated with Compliance Within a Postpartum Hypertension Remote Monitoring Program

Jenny Y. Mei^1^; Audra Fain^2^; Kate Corry‐Saavedra^3^; Tina A. Nguyen^2^; Thalia Mok^2^; Aisling M. Murphy^2^

*^1^Stanford University, Mountain View, CA; ^2^University of California, Los Angeles, Los Angeles, CA; ^3^University of California, Irvine, University of California, Irvine, CA*

4:00 PM ‐ 6:00 PM

**Objective**: Remote blood pressure (BP) monitoring has been shown to decrease postpartum Emergency Department (ED) visits and readmissions. We evaluate characteristics associated with compliance within a remote BP monitoring program.

**Study Design**: A retrospective cohort study of birthing patients with peripartum hypertension (HTN) at a quaternary care center over 2 years. This study is part of an ongoing postpartum quality improvement project that entails lower BP targets and universal remote BP monitoring. Inclusion criteria were delivery at the study institution and diagnosis of HTN disorder of pregnancy at time of discharge. Primary outcome was compliance with utilizing remote BP monitoring, defined as logging at least one BP within the program. We compared maternal and hypertensive characteristics between groups.

**Results**: Out of 6410 deliveries between April 2022‐April 2024, 2019 (31.5%) pregnancies met inclusion criteria, of which 1509 (74.7%) were compliant with utilizing remote BP monitoring. There were higher rates of compliance with higher maternal age (in years, 34.2±5.0 vs 33.0±6.3, p< 0.001), nulliparity (71.2% vs 56.5%, p< 0.001), lower BMI (in kg/m^2^, 30.2±6.1 vs 31.6±7.0, p< 0.001), prenatal aspirin use (41.9% vs 33.1%, p< 0.001), IVF pregnancy (12.3% vs 7.8%, p = 0.006), and private insurance (84.9% vs 59.4%, p< 0.001). Patients discharged on anti‐hypertensive medication were more likely to be compliant (36.0% vs 24.1%, p< 0.001) as were those with longer postpartum lengths of stay (in days, 2.4±1.3 vs 2.3±1.2, p = 0.027). Compliance was associated with lower rate of ED visit or readmission (1.8% vs 3.3%, p = 0.039).

**Conclusion**: Characteristics that are associated with higher rates of compliance with remote BP monitoring include nulliparous, older, and privately insured patients. Compliance was associated with lower rates of postpartum ED visit or readmission. Continued work is needed to identify barriers to remote BP monitoring and target these areas for improvement within programs to improve compliance and further decrease postpartum readmission rates.

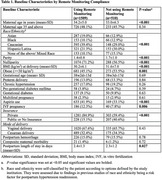

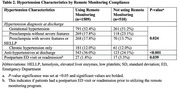

## 514 | Risk Factors for Discharge on Anti‐Hypertensive Medication with Lower Blood Pressure Targets

Jenny Y. Mei^1^; Audra Fain^2^; Kate Corry‐Saavedra^3^; Tina A. Nguyen^2^; Thalia Mok^2^; Aisling M. Murphy^2^

*^1^Stanford University, Mountain View, CA; ^2^University of California, Los Angeles, Los Angeles, CA; ^3^University of California, Irvine, University of California, Irvine, CA*

4:00 PM ‐ 6:00 PM

**Objective**: With more institutions implementing lower blood pressure (BP) targets prior to postpartum discharge for patients with hypertensive disorders of pregnancy, a growing number of patients are discharged on anti‐hypertensive (anti‐HTN) medication. We aim to evaluate risk factors associated with being discharged on anti‐HTN medication with lower BP goals.

**Study Design**: A retrospective cohort study of birthing patients with peripartum hypertension (HTN) at a quaternary care center over 2 years. This study is part of an ongoing postpartum quality improvement project that entails lower BP targets and universal remote BP monitoring. Goal blood pressure was under 140/90 leading up to discharge. Inclusion criteria were delivery at the study institution and diagnosis of HTN disorder of pregnancy. Primary outcome was prescription of anti‐HTNs at time of discharge. Characteristics associated with discharge on anti‐HTNs were compared between groups.

**Results**: Out of 6410 deliveries between April 2022‐April 2024, 2019 (31.5%) pregnancies met inclusion criteria, of which 666 (33.0%) were discharged on anti‐HTN medication. Baseline characteristics associated with discharge on anti‐HTN included maternal age 40 and above (17.0% vs 11.8%, p = 0.001), non‐Hispanic Black race (13.7% vs 9.5%, p = 0.005), chronic HTN (28.4% vs 12.3%, p< 0.001), prenatal aspirin use (52.1% vs 33.6%, p< 0.001), and having public or no insurance (25.2% vs 19.7%, p = 0.005). Other risk factors for discharge on anti‐HTN medication include proteinuria (43.2% vs 23.6%, p< 0.001) and preeclampsia with severe features (40.4% vs 5.1%, p< 0.001). Patients discharged on anti‐HTNs were more likely to be compliant with remote BP monitoring (97% vs 94.2%, p = 0.013) and undergo outpatient medication adjustments (24.6% vs 14.5%, p< 0.001).

**Conclusion**: At an institution implementing lower BP targets aiming for normotension, certain traditional high‐risk patients remain at increased risk for discharge on anti‐HTN medication. Continued research is needed to identify ways of mitigating long‐term comorbidities for this high‐risk cohort.

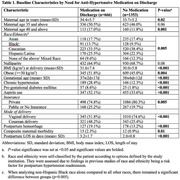

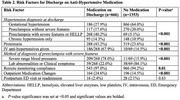

## 515 | Can the Supplementation of Ferric‐Carboxymaltose Improve Postpartum Depression After Birth? a Randomized, Controlled Clinical Trial

Jeong‐won Oh^1^; Eun Sil Lee^2^; Kyu Yeon Choi^2^; Subeen Hong^3^; Hyun Sun Ko^4^; Geun Joon Cho^5^; Jeong Jae Lee^2^

*^1^Department of Obstetrics and Gynecology, Soonchunhyang University Seoul Hospital, Soonchunhyang University College of Medicine, Soonchunhyang University Seoul Hospital, Soonchunhyang University College of Medicine/Seoul, Seoul‐t'ukpyolsi; ^2^Department of Obstetrics and Gynecology, Soonchunhyang University Seoul Hospital, Soonchunhyang University College of Medicine, Seoul, Soonchunhyang University Seoul Hospital, Soonchunhyang University College of Medicine, Seoul, Seoul‐t'ukpyolsi; ^3^Seoul St. Mary's Hospital, College of Medicine, The Catholic University of Korea, Seoul, Seoul‐t'ukpyolsi; ^4^Department of Obstetrics and Gynecology, Catholic University College of Medicine/ Seoul, Catholic University College of Medicine/ Seoul, Seoul‐t'ukpyolsi; ^5^Department of Obstetrics and Gynecology, Korea University College of Medicine/ Seoul, Seoul‐t'ukpyolsi*

4:00 PM ‐ 6:00 PM

**Objective**: This study aims to compare the efficacy of intravenous(IV) ferric‐carboxymaltose(FCM) administration versus conventional oral iron supplementation in improving postpartum depression(PPD) in postpartum women with iron‐deficiency anemia(IDA).

**Study Design**: A randomized, open‐label, controlled clinical trial was conducted at three centers in South Korea between 2021 and 2023. Postpartum women with (i)moderate IDA, diagnosed with hemoglobin(Hb) levels of 8≤ Hb< 11g/dL and ferritin< 30ng/mL or transferrin saturation(TSAT) < 20%, and with (ii)a high risk of PPD based on the Edinburgh Postnatal Depression Scale(EPDS) score of ≥ 8 within the first three days after delivery, were enrolled. Following randomization, participants received either IV FCM(500‐1000 mg) or oral iron(20‐400 mg). The primary endpoint was the change in EPDS score from baseline to 8 weeks postpartum. Hematologic parameters, presence of PPD (EPDS score > 11), and maternal fatigue were evaluated at ten days, 4, 6, and 8 weeks.

**Results**: Among 96 patients, 90 were analyzed, with 45 in each group. The aggregated change in EPDS scores over 8 weeks postpartum did not differ between the groups, while the mean EPDS score(±SD) for the entire study population significantly decreased from 11.5 ± 3.2 initially to 8.6±5.0 at 8 weeks(p‐value for trend < 0.001). The prevalence of PPD at 8 weeks was 22.2 %(10/45) in each group, with no significant difference. Variables such as Hb changes and type of iron therapy did not affect the risk of PPD, except for fatigue at 8 weeks (adjusted odds ratio 1.05, 95% confidence interval 1.01–1.09; P = 0.016). Hb levels increased well in both groups, but changes in Hb, ferritin, and TSAT levels at 8 weeks (p < 0.001) and fatigue scores at 6 weeks(p = 0.03) were significantly improved in the IV iron group compared to the conventional group.

**Conclusion**: While the administration of FCM did not show significant benefits over placebo in improving EPDS scores, the study`s findings demonstrated that IV iron was more effective than conventional oral iron in correcting postpartum IDA, iron stores and reducing fatigue.

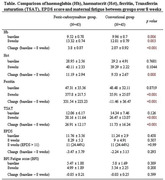

## 516 | Postpartum Healthcare Utilization and Morbidity Amongst Diabetic Versus Non‐Diabetic Individuals in Remote Hypertension Program

Jessica T. Chen^1^; Rodolfo Fernandez‐Criado^1^; Scott Machado^1^; Tracy L. Jackson^1^; Nicole Konecke^1^; William Hunt^1^; Danielle Simmons^1^; Maria Mejia Castillo^1^; Alisse Hauspurg^2^; Methodius G. Tuuli^3^; Adam K. Lewkowitz^1^

*^1^Women & Infants Hospital of Rhode Island / Alpert Medical School of Brown University., Providence, RI; ^2^Alpert Medical School of Brown University, Providence, RI; ^3^Women & Infants Hospital of Rhode Island / Alpert Medical School of Brown University, Providence, RI*

4:00 PM ‐ 6:00 PM

**Objective**: Individuals with diabetes (gestational or pregestational) are at increased risk of adverse outcomes, particularly in the setting of chronic hypertension (HTN) or new‐onset HTN in pregnancy. Though remote self‐measured blood pressure (SMBP) monitoring programs may reduce rates of HTN‐related hospital readmission, emergency department (ED) presentations, or severe maternal morbidity (SMM), it is unclear whether the extent of this reduction differs between those with and without diabetes. We aimed to compare outcomes between postpartum patients in our remote SMBP program with versus without diabetes.

**Study Design**: Postpartum patients with HTN at our tertiary care hospital are offered enrollment in our remote SMBP program, which includes BP monitoring for 6 weeks postpartum. For this analysis, participants were stratified by presence or absence of diabetes (pregestational or gestational). Those with unknown diabetes status were excluded. The primary outcome was a composite of postpartum readmission or ED presentation for HTN within 30 days of delivery hospitalization. Secondary outcomes included HTN‐related SMM. A generalized linear model was used to estimate relative risks (RR) after adjustment for differences in demographic, obstetric, and medical conditions from table 1.

**Results**: Of 2003 participants in the SMBP program, 17.2% had diabetes. Compared to those without, those with diabetes were older, less likely to identify as White, less likely to have gestational but more likely to have chronic hypertension, more likely to be delivered via cesarean, and had a lower median gestational age at delivery (**Table 1**). After adjusting for these factors, there was no difference in the risk of the composite outcome of HTN‐related postpartum readmission or ED presentation between those with and without diabetes [15.9% versus 16.6%; adjusted RR 0.9 (0.7, 1.2)]. Similarly, there were no differences in secondary outcomes (**Table 2**).

**Conclusion**: In our remote SMBP program for postpartum patients with HTN, both unplanned HTN‐related healthcare utilization and SMM were similar between those with and without diabetes.

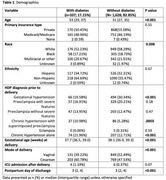

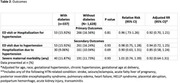

## 517 | Socioeconomic Status and Adverse Pregnancy Outcome Increase Risk of Long‐Term Cardiovascular Disease, Analyzing Long‐Term Cohort

JI HOI KIM^1^; Jeesun Lee^2^; Manu Shivakumar^3^; Dokyoon Kim^3^; Kue Hyun Kang^4^; Jin Yong Lee^1^; Haibin Bai^5^; Chan‐Wook Park^2^; Joong Shin Park^2^; Juwon Lim^1^; Jeehoon kang^1^; Soo Heon Kwak^1^; Seung Mi Lee^2^

*^1^Seoul National University Hospital College of Medicine, Seoul, Seoul‐t'ukpyolsi; ^2^Seoul National University College of Medicine, Seoul, Seoul‐t'ukpyolsi; ^3^University of Pennsylvania, Philadelphia, PA; ^4^Ain Hospital, Incheon, Inch'on‐jikhalsi; ^5^Seoul National University Hospital, Seoul, Seoul‐t'ukpyolsi*

4:00 PM ‐ 6:00 PM

**Objective**: Adverse pregnancy outcomes (APO) have been reported to increase the risk of long‐term atherosclerotic cardiovascular disease (ASCVD) later in life. Additionally, socioeconomic status (SES) is known to be associated with ASCVD risk. In the current analysis, we determined the interaction between SES and APO, specifically, if low SES further aggravates the ASCVD risk after APO.

**Study Design**: We conducted the current analysis using data from the UK Biobank, which is a large prospective cohort. This data includes participants in age 40 to 69 years recruited between 2006 and 2010, with ongoing follow‐up. APO included hypertensive disease during pregnancy (HDP), gestational diabetes mellitus (GDM), and low birth weight (< 2.5kg). SES was analyzed using the following indicators; household income, education, employment and Townsend Deprived Score. The study population were categorized into four main groups according to APO and SES: no APO/high SES, APO/high SES, no APO/low SES and APO/low SES groups. The incidence of long‐term ASCVD events and the cumulative hazard risk (HR) for ASCVD was analyzed according to the APO and SES categories.

**Results**: In total, 219,147 female participants were analyzed. The results indicated that the APO group had relatively lower SES in all indicators and higher incidence of ASCVD, hypertension, hyperlipidemia, diabetes mellitus, compared to the no APO group. After adjusting for the confounding factors, the risk of ASCVD was highest in the group with APO and low SES group. Specifically, the HR was 2.029 (95 % CI, 1.762‐2.338, p < 0.001) in those with APO and unemployment.

**Conclusion**: We found that the CVD risk varied according to SES and APO. This study suggests the importance for support policies tailored to individuals’ SES to prevent APO and improve long‐term cardiovascular disease.

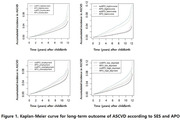

## 518 | The Risk of Long‐Term Cardiovascular Disease after Adolescent Pregnancies

JI HOI KIM^1^; Haemin Kim^2^; Manu Shivakumar^3^; Dokyoon Kim^3^; Kue Hyun Kang^4^; Minyoung Park^1^; Chan‐Wook Park^5^; Joong Shin Park^5^; Juwon Lim^1^; Jeehoon kang^1^; Seung Mi Lee^5^

*^1^Seoul National University Hospital College of Medicine, Seoul, Seoul‐t'ukpyolsi; ^2^Kyungpook National University Chilgok Hospital, Kyungpook National University, School of Medicine, Daegu, Ch'ungch'ong‐bukto; ^3^University of Pennsylvania, Philadelphia, PA; ^4^Ain Hospital, Incheon, Inch'on‐jikhalsi; ^5^Seoul National University College of Medicine, Seoul, Seoul‐t'ukpyolsi*

4:00 PM ‐ 6:00 PM

**Objective**: Previous studies have reported that adolescent pregnancy is associated with higher risks of adverse pregnancy outcomes (APO) compared to pregnancies after the age of 20. Although there have been accumulating evidence regarding age at reproductive event, such as menarche or menopause, and long‐term cardiovascular (CVD) risk, there aren't enough research on the effect of adolescent pregnancy on long‐term outcome. This study aimed to evaluate the risk of long‐term CVD after adolescent pregnancy using large cohort data.

**Study Design**: This study was based on the UK biobank database. Participants aged 40 to 69 years, who were recruited between 2006 and 2010, were analyzed. Clinical events were collected from the time of enrollment. The study population was divided into groups of first delivery before and after the age of 20. The risk of atherosclerotic cardiovascular disease (ASCVD) was evaluated according to adolescent pregnancy (< 20 years) and adult pregnancy ( ≥ 20 years).

**Results**: Among 182,843 women in the UK biobank, 28,090 women had their first delivery at adolescence with an average age at childbirth of 18.69 years. The adolescent pregnancy group had lower rates of hypertensive disease during pregnancy (HDP) compared to the adult pregnancy group. However, the incidence of ASCVD was 8.4 % (2269/28,090) in the adolescent pregnancy group, compared to the adult pregnancy group. Even after adjusting for confounding variables, the risk of ASCVD remained higher in adolescent pregnancy group (HR 1.320; 95 % CI 1.245 to 1.398, p < 0.001).

**Conclusion**: Adolescent pregnancy increases the risk of long‐term ASCVD. Healthcare providers should focus on both prenatal care and long‐term postpartum management for adolescent pregnancies. To improve potential risks and outcomes, appropriate care and comprehensive support are necessary throughout pregnancy and the postpartum period.

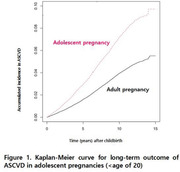

## 519 | Risk Factors Associated with Unexpected Neonatal Resuscitation Among Term Neonates in Uncomplicated Pregnancies

Nari Kim^1^; Eun Hee Ahn^1^; Sang Hee Jung^1^; Hyun Mee Ryu^1^; Ji Yeon Lee^2^

*^1^Department of Obstetrics and Gynecology, CHA Bundang Medical Center, CHA University School of Medicine, CHA Bundang Medical Center, CHA University School of Medicine / Seongnam, Kyonggi‐do; ^2^Department of Obstetrics and Gynecology, CHA Bundang Medical Center, CHA University School of Medicine, Seongnam, Kyonggi‐do*

4:00 PM ‐ 6:00 PM

**Objective**: Unexpected neonatal resuscitation (NR) in anticipated smooth deliveries is troubling without available neonatologists. This study evaluated risk factors for unexpected NR in singleton pregnancies with planned cesarean deliveries after 36 weeks gestation and no fetal abnormalities.

**Study Design**: This retrospective cohort study included singleton pregnant women at our hospital who received prenatal care from the first trimester, had no fetal abnormalities, and delivered via planned cesarean section after 36 weeks of gestation, between 2013 and 2023. Only cases with stable vital signs and no significant blood loss from anesthesia to delivery were included. Women with medical conditions requiring hospitalization were excluded. Cases were divided into the NR group (5‐minute Apgar score < 7) and the control group. Maternal characteristics, medical history, and laboratory tests from the first trimester and the last visit before delivery were reviewed. We formulated a scoring model after performing multivariable analysis using adjusted odds ratios (aOR).

**Results**: A total of 5,324 cases were included, with the NR group comprising 78 cases (1.5%). Five variables were associated with unexpected NR: prior preterm birth (aOR 5.6, 95%CI 1.9‐16.2, P = 0.002), psychiatric medication during pregnancy (aOR 10.3, 95%CI 1.8‐59.2, P = 0.009), leukocytosis (WBC count ≥15,000/mm^3^) in the first trimester (aOR 4.1, 95%CI 1.5‐11.2, P = 0.007), leukocytosis just before delivery (aOR 3.1, 95%CI 1.3‐7.5, P = 0.010), and increased HbA1c (≥6.0%) just before delivery (aOR 2.7, 95%CI 1.1‐7.0, P = 0.048). We formulated a scoring model, assigning each factor a score of 1 to 4 based on the aOR. A score of 4 out of 13 had a sensitivity of 72% and a specificity of 82% for predicting unexpected NR. The AUC was 0.817 (P< 0.001).

**Conclusion**: Prior PTB, psychiatric medication, leukocytosis in the first trimester, leukocytosis just before delivery, and increased HbA1c just before delivery were identified as risk factors for unexpected NR in uncomplicated pregnancies. This scoring model may provide useful information for predicting unexpected NR.

## 520 | Association of Isolated Leukocytosis in Early Pregnancy with Adverse Pregnancy Outcomes

Ji Yeon Lee^1^; Nari Kim^2^; Yeomin E. Kang^3^

*^1^Department of Obstetrics and Gynecology, CHA Bundang Medical Center, CHA University School of Medicine, Seongnam, Kyonggi‐do; ^2^Department of Obstetrics and Gynecology, CHA Bundang Medical Center, CHA University School of Medicine, CHA Bundang Medical Center, CHA University School of Medicine / Seongnam, Kyonggi‐do; ^3^University of Maryland, Baltimore, MD*

4:00 PM ‐ 6:00 PM

**Objective**: Animal studies suggest that maternal systemic inflammation in early pregnancy can affect adverse outcomes. There is no clear standard for maternal leukocytosis, and there is limited research on how high white blood cell(WBC) counts affect outcomes. This study aims to investigate how high WBC counts in the first trimester relate to adverse pregnancy outcomes and identify the WBC count level linked to poor prognosis.

**Study Design**: This retrospective cohort study included healthy, singleton pregnant women who delivered between 2014 and 2023. Cases included only those without fetal anomalies and no fever at the time of the first trimester laboratory test. Women showing signs of infection/inflammation or with autoimmune diseases were excluded. Women with a WBC count of 13,000‐14,000 in the first trimester were classified as group I (n = 382), those with a WBC count of 14,000‐15,000 as group II (n = 151), and those with a WBC count of ≥15,000 as group III (n = 70). Meanwhile, those with a WBC count of 7,500‐8,500 were designated as the control group (n = 1,524). Multivariate analysis was performed considering maternal characteristics.

**Results**: Groups I/II did not show statistical differences in adverse outcomes compared to the control group. However, group III had significantly higher risks compared to the control group, including the risk of preterm birth(PTB) before 32 weeks (aOR 5.5, 95% CI 1.5‐20.6, P = 0.012), before 34 weeks (aOR 8.2, 95%CI 3.4‐20.3, P< 0.001), and before 36 weeks (aOR 2.9, 95%CI 1.4‐6.1, P = 0.006). Additionally, the risks of gestational diabetes(GDM) (aOR 2.1, 95%CI 1.2‐3.7, P = 0.013), cesarean delivery(CD) (aOR 1.8, 95%CI 1.1‐3.1, P = 0.029), transient tachypnea(TTN) (aOR 2.6, 95%CI 1.1‐6.3, P = 0.036), and neonatal jaundice (aOR 1.7, 95%CI 1.1‐2.8, P = 0.039) were also elevated in group III compared to the control group.

**Conclusion**: Associations between high WBC counts (≥15,000) in early pregnancy and increased risks of PTB, GDM, CD, TTN, and neonatal jaundice have been observed. High first‐trimester WBC counts may help predict adverse pregnancy outcomes.

## 521 | Opt‐Out and Rapid Syphilis Testing in Pregnant Patients Presenting to the Ed: a Pre‐Post Study

Joe Haydamous^1^; Ana Davie^1^; Jose C. Noriega Valdez^1^; Cassandra Igbe^1^; Aidan Blackwell^1^; Carrie Bakunas^2^; Jeffrey K. Klausner^3^; Leandro Mena^4^; Irene A. Stafford^5^

*^1^Division of Maternal‐Fetal Medicine, Department of Obstetrics, Gynecology and Reproductive Sciences, McGovern Medical School at UTHealth Houston, Houston, TX; ^2^Department of Emergency Medicine, McGovern Medical School at The University of Texas Health Science Center at Houston (UTHealth), Houston, Texas, Houston, TX; ^3^University of Southern California, Los Angeles, CA; ^4^All‐In Health Solutions LLC, Atlanta, GA; ^5^McGovern Medical School at UTHealth Houston, Houston, TX*

4:00 PM ‐ 6:00 PM

**Objective**: According to regional pilot data, 35% of pregnant patients with newborns diagnosed with congenital syphilis (CS) visited the emergency department (ED) during pregnancy but were not tested for syphilis. This represents a critical missed opportunity to identify and treat syphilis in pregnant patients and reduce the incidence of CS. This pre‐ and post‐implementation study aims to determine if implementing an opt‐out laboratory‐based and rapid syphilis testing program improves the detection and treatment of syphilis during pregnancy in the ED.

**Study Design**: This pre‐post implementation study was conducted at the University of Texas Health Science Center, Houston, TX after institutional approval. During the pre‐implementation phase (11/01/2023 ‐ 02/29/2024), eligible pregnant patients presenting to the ED underwent standard lab‐based syphilis testing only when clinically indicated. In the post‐implementation phase (03/01/2024 ‐ 06/25/2024), pregnant patients without a documented syphilis result or prenatal care received lab‐based opt‐out syphilis testing with or without the Syphilis Health Check (SHC) point‐of‐care test, with coordinators facilitating linkage to prenatal care for all patients.

**Results**: During the pre‐implementation period, 302 pregnant patients presented to the ED for care, with only 6 (2%) undergoing syphilis testing, yielding no positive results. In the post‐implementation period, 322 pregnant patients presented to the ED. Of these, 79 (24.5%) were tested for syphilis, representing a 12.5‐fold increase in screening (p < 0.001). Three patients tested positive for syphilis, indicating a 3.8% prevalence rate. Moreover, 70% of those who tested positive were scheduled for prenatal care by research coordinators dedicated to the project.

**Conclusion**: The implementation of an opt‐out and rapid syphilis testing program for underserved pregnant patients in the ED led to a significant increase in syphilis testing and referral for treatment. The public health impact of this program for adults and neonates is likely to demonstrate further substantial benefits, particularly in high‐prevalence regions.

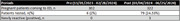

## 522 | The Texas Experience: The Impact of Levels of Care on Severe Maternal Morbidity

John J. Byrne^1^; Sangeeta Jain^2^; Kirsten Robinson^3^; David B. Nelson^4^; Carlos Anaya^5^; Mattie Mason^6^; Sadhana Chheda^7^; Patrick S. Ramsey^8^

*^1^University of Texas Health San Antonio, San Antonio, TX; ^2^University of Texas Medical Branch, Galveston, TX; ^3^Texas Tech University Health Sciences Center School of Medicine, Lubbock, TX; ^4^University of Texas Southwestern Medical Center, Dallas, TX; ^5^University of Texas Rio Grande Valley School of Medicine, Edinburg, TX; ^6^New Life Perinatal Health Care Services, Inc, Houston, TX; ^7^TTUHSC‐El Paso, El Paso, TX; ^8^University of Texas Health Science Center San Antonio, San Antonio, TX*

4:00 PM ‐ 6:00 PM

**Objective**: The State of Texas instituted Levels of Maternal Care (LoMC) Designation in 2018, with all hospitals that provide maternity services receiving a formal LoMC designation by August 31, 2021. We sought to assess the impact of instituting LoMC in Texas on the rates of severe maternal morbidity (SMM) across levels of care.

**Study Design**: We conducted a retrospective, cross‐sectional study for years 2018 and 2022 using Texas Medicaid Data, prior to‐ and after‐ LoMC designation occurred. Data extracted included total SMM rate, hysterectomy rate, and hospital level of care. Data related to total SMM (without transfusion), hysterectomy rate, and eclampsia rates were compared pre‐ and post‐implementation.

**Results**: There are 219 facilities that have achieved maternal designation (55–level I, 88–level II, 44–level III, 32–level IV) by 2021. The number of Medicaid births increased significantly from 2018 to 2022 in level I, II, III facilities (p< 0.001), and was not statistically different in the level IV facilities (p = 0.33). Of the 260,986 deliveries included in 2018 and 258,674 deliveries included in 2022, there were 4675 (1.79%) and 4533 (1.75%) SMM events, respectively (p = 0.29). There was a decrease of 0.5% in SMM rates in level I centers from 2018‐2022 (p = 0.001, Table). There were no statistically significant differences between SMM events in  level II, III, IV centers. SMM among hemorrhage cases was noted to be reduced from 2018 to 2022, and was statistically decreased in level I (p = 0.002) and level IV centers (p = 0.04). SMM among cases of patients with preeclampsia was significantly reduced in Level I‐IV centers (P< 0.01). There was a non‐statistically significant decrease in hysterectomies in Level I/II facilities from 2018 (64/204, 31.4) to 2022 (92/319, 28.8%), (p = 0.54).

**Conclusion**: Using Texas Medicaid data, which includes approximately 50% of births in Texas, there was a statistically significant decrease in SMM rates in Level 1 centers after implementation of LoMC. Additionally, there was a reduction in both hemorrhage‐ and preeclampsia‐related SMM during this timeframe.

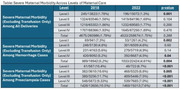

## 523 | Tranexamic Acid Prevents Postpartum Hemorrhage During Cesarean Delivery: A Meta‐Analysis

Jordan A. McKinney^1^; Gustavo Vilchez^2^; Luis Sanchez‐Ramos^3^

*^1^University of Florida College of Medicine, Gainesville, FL; ^2^UTHealth Houston, Houston, TX; ^3^University of Florida College of Medicine, Jacksonville, FL*

4:00 PM ‐ 6:00 PM

**Objective**: Postpartum hemorrhage remains a leading cause of maternal morbidity and mortality worldwide, particularly following cesarean delivery. Effective prophylactic interventions are critical to mitigate this risk and improve maternal outcomes. Tranexamic acid (TXA), an antifibrinolytic agent, has shown promise in reducing blood loss in various surgical contexts. This updated systematic review and meta‐analysis aimed to assess the impact of prophylactic tranexamic acid on preventing postpartum hemorrhage and the need for blood transfusion in patients undergoing cesarean delivery.

**Study Design**: We systematically searched multiple electronic databases and grey literature from their inception up to May 24, 2024, to identify randomized controlled trials (RCTs) that compared prophylactic TXA with placebo or no treatment in patients undergoing cesarean delivery. The primary outcome was postpartum hemorrhage. The need for a blood transfusion was the secondary outcome. We performed a meta‐analysis using random‐effects models to calculate odds ratios (OR) along with 95% confidence intervals (CI). We also performed an extensive literature review to investigate the cost‐effectiveness for the prophylactic TXA at the time of cesarean. 

**Results**: Data from 29 RCTs involving 22,003 participants was collected. The meta‐analysis showed that prophylactic TXA in cesarean delivery reduces the risk of postpartum hemorrhage (RR 0.54; 95% CI 0.43‐0.67; FI 84) and the need for blood transfusion (RR 0.45; 95% CI 0.34‐0.60; FI 63) compared to placebo/no treatment.

**Conclusion**: Our findings indicate that the use of prophylactic TXA in cesarean delivery reduces the risk of postpartum hemorrhage and the need for blood transfusion. These results support the routine use of prophylactic TXA in cesarean deliveries for individuals at high risk of postpartum hemorrhage.

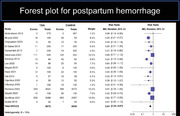

## 524 | Antenatal Corticosteroids Show Limited Benefit in Improving Neonatal Outcomes in Late‐Preterm Fetuses: A Meta‐Analysis

Jordan A. McKinney^1^; Gustavo Vilchez^2^; Lifeng Lin^3^; Luis Sanchez‐Ramos^4^

*^1^University of Florida College of Medicine, Gainesville, FL; ^2^UTHealth Houston, Houston, TX; ^3^University of Arizona, Tucson, AZ; ^4^University of Florida College of Medicine, Jacksonville, FL*

4:00 PM ‐ 6:00 PM

**Objective**: Recent large‐scale trials highlight the evolving landscape of antenatal corticosteroid (ACS) use in late preterm gestations (34–36 weeks), emphasizing the need for reassessment of current practices due to limited data on long‐term psychiatric and neurodevelopmental outcomes. This systematic review and meta‐analysis synthesize current evidence on the benefits and risks of ACS administration during this period.

**Study Design**: We conducted a search of electronic databases and gray literature from inception to February 1, 2024. We included randomized trials (RCTs) that assessed neonatal outcomes in newborns whose mothers received ACS during the late preterm period, compared to those whose mothers received a placebo or no treatment. Pooled unadjusted odds ratios (OR) with 95% confidence intervals (CI) were calculated using a random‐effects model. Primary outcomes included the need for respiratory support and neonatal hypoglycemia, with other adverse neonatal outcomes as secondary. We also calculated fragility indices, 95% prediction intervals, and performed subgroup analyses to evaluate the robustness of the results.

**Results**: Data from 12 RCTs were included in the meta‐analysis. ACS administration did not significantly reduce the likelihood of neonates requiring any form of respiratory support compared to placebo or no treatment (OR, 0.74; 95% CI, 0.54‐1.02). Neonates exposed to ACS did not have a significantly increased chances of hypoglycemia (OR, 1.37; 95% CI, 0.99‐1.90). However, ACS exposure was associated with reduced odds of requiring resuscitation at birth (OR, 0.54; 95% CI, 0.35‐0.83) and a lower probability of developing respiratory distress syndrome (OR, 0.63; 95% CI, 0.40‐0.99).

**Conclusion**: The findings of this meta‐analysis suggest that late preterm ACS administration may offer limited benefits in preventing neonatal respiratory morbidity and improving other neonatal outcomes. Current evidence highlights the importance of shared decision‐making in providing patient‐centered care and underscores the need for further research into the efficacy and safety of late preterm ACS.

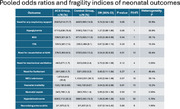

## 525 | Maternal Immune Microenvironment in Fetal Congenital Heart Disease

Jordan R. Lowe^1^; Rajeev Aurora^1^; Dana Alshekhlee^1^; Megan Krupp^1^; Amanda Criebaum^2^; Niraj R. Chavan^3^

*^1^Saint Louis University, St. Louis, MO; ^2^Saint Louis University, St. Louis, MT; ^3^Saint Louis University School of Medicine, St. Louis, MO*

4:00 PM ‐ 6:00 PM

**Objective**: This study was undertaken to evaluate the relationship between maternal immunological milieu (cytokines and chemokines) and complex fetal congenital heart disease (CHD).

**Study Design**: We performed a prospective, non‐interventional, case‐control study of pregnancies with complex fetal CHD (cases) at a single tertiary academic fetal care center matched to those with normal fetal cardiac anatomy (controls). Maternal serum was collected after CHD diagnosis based on fetal echocardiogram evaluation. Serum cytokine and chemokine levels were assessed using multiplex enzyme‐linked immunosorbent assay (ELISA) and compared across groups. Demographic (age, race, parity, insurance) and clinical characteristics (obesity, diabetes, hypertension, connective tissue / thyroid disorder, In‐vitro fertilization and history of CHD) were abstracted from electronic medical records and compared using student's t test and chi squared / Fisher's exact test for continuous and categorical data respectively. Statistical significance was set at p < 0.05.

**Results**: ELISA was performed for 23 cases with fetal CHD and 25 matched controls. The predominant CHD phenotypes were hypoplastic (left/right) heart syndrome (9/23, 39%), tetralogy of Fallot (4/23, 17%), and coarctation (3/23, 13%). Demographic and clinical characteristics were similar across both groups except gestational age at specimen collection [cases: 30.1±4.4 vs controls 27.5±2.8 weeks (mean ± SD), p = 0.02]. Multiplex analysis of 38 pro‐ and anti‐inflammatory cytokines and chemokines was performed. Of those assayed, IL‐2 was significantly overexpressed in cases vs control (positive fold change 1.58, p = 0.03). Other analytes–including IL‐8 (p = 0.03) fractalkine (p = 0.02), GM‐CSF (p = 0.01), IFNα2 (p = 0.04), IL‐17A (p = 0.01), and MCP‐1(p = 0.03) were significantly under expressed in cases compared to controls.

**Conclusion**: Significant differences were noted in immunoregulatory cytokine and chemokine expression among pregnancies with and without fetal CHD. Our findings suggest a predominant involvement of the Th1 cell line in fetal CHD pathogenesis.

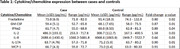

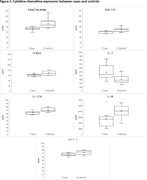

## 526 | Association Between Antibiotic Regimen and Adverse Outcomes in Pregnancies Complicated by Chorioamnionitis

Jordan R. Lowe^1^; Niraj R. Chavan^2^; Audrey Brechter^1^; Tracy M. Tomlinson^1^

*^1^Saint Louis University, St. Louis, MO; ^2^Saint Louis University School of Medicine, St. Louis, MO*

4:00 PM ‐ 6:00 PM

**Objective**: We aimed to assess the association between antibiotic regimen choice subsequent to a change in institutional treatment guidelines for management of chorioamnionitis and adverse maternal and neonatal outcomes.

**Study Design**: Passive prospective, single center, cohort study of pregnancies complicated by chorioamnionitis treated with either intravenous ampicillin, gentamicin, ± clindamycin (AGC, 2010‐2017) or piperacillin/tazobactam with clindamycin (PTC, 2017‐2021). Acute chorioamnionitis was defined based on accepted clinical and histopathological criteria. We assessed the univariate associations between pregnancy characteristics and antibiotic regimen administered in addition to our composite adverse outcome: maternal sepsis, atonic postpartum hemorrhage, and/or neonatal sepsis with antibiotic administration prior to delivery, readmission for endometritis, wound infection, and/or maternal temperature of 99.5 for >24 hours after administration of first antibiotic dose. Factors with P < 0.2 on one or both univariate analyses were considered for incorporation into a multivariable model predictive of the primary outcome.

**Results**: Of 403 pregnancies, 211 were treated with AGC vs 192 with PTC regimens. Most characteristics were similar between antibiotic regimen groups, however women receiving AGC were younger and fewer had a public payor source. The 88 (22%) pregnancies with the primary outcome had a longer median duration of rupture of membranes prior to delivery (20.6 vs 14.3 hours, *P* < 0.01), more underwent cesarean delivery (CD) (36% vs 11%, *P* < 0.0001), and more received the AGC regimen (27 % vs 16%, *P* 0.01). Factors associated with the primary outcome on multivariable analysis include BMI >30, substance use, delivery < 37 weeks, CD, and treatment with AGC regimen (aOR 2.15, 95% CI 1.08‐3.46). The 5‐covariate model had an optimism‐corrected bootstrapped (1000 replicates) AUROC of 0.76 (95% CI 0.69‐0.80).

**Conclusion**: Treatment of intrapartum acute chorioamnionitis with the PTC regimen has the potential to lead to improved maternal and neonatal outcomes.

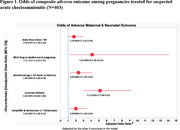

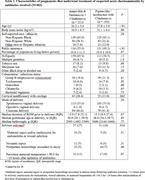

## 527 | Management and Referral Patterns for Opioid and Other Substance Use Disorder in Pregnancy

Joshua S. George^1^; Ellery G. Sarosi^2^; Harini Pennathur^3^; Rachel A. Clark^4^; Kenechukwu Odenigbo^4^; Shayla Parthasarathy^4^; Michelle Moniz^1^; Lisa Kane Low^1^; Maria Muzik^1^; Alex Peahl^5^

*^1^University of Michigan, Ann Arbor, MI; ^2^University of Michigan Medical Center, Department of Obstetrics and Gynecology, Ann Arbor, MI; ^3^Texas A&M University School of Engineering Medicine; ^4^University of Michigan Medical School, Ann Arbor, MI; ^5^Michigan Medicine, Ann Arbor, MI*

4:00 PM ‐ 6:00 PM

**Objective**: To explore how various outpatient clinic types providing obstetric services manage care for birthing people with opioid and other substance use disorder (OUD/SUD) in pregnancy.

**Study Design**:  Prenatal care providers from across the state of Michigan were identified through systematic web searches and snowball sampling. We conducted semi‐structured surveys supported by Blue Cross Blue Shield of Michigan on routine prenatal care practices including management of high‐risk conditions such as OUD/SUD. Specifically, we queried if clinics manage OUD/SUD in pregnancy, co‐manage with another provider, or refer patients to other providers. We assessed practices across clinic attributes using chi‐square tests. 

**Results**: Of the 18 clinics across 8 out of 10 designated prosperity regions in Michigan who completed the survey, 7 clinics self‐managed OUD/SUD, 6 clinics utilized co‐management, and 5 clinics utilized referrals for specialized care. Federally Qualified Health Centers and outpatient clinics associated with hospitals were more likely to self‐manage patients with OUD/SUD than private practices (p = 0.01). All respondents from private practices reported referring as their primary management strategy. Clinics with Epic or Cerner electronic health record (EHR) systems were more likely to manage or co‐manage OUD/SUD than clinics with local EHR systems (p = 0.01). Management strategy did not differ by the clinic population size, state designated prosperity regions, or insurance limitations. 

**Conclusion**: OUD/SUD is the leading cause of pregnancy associated mortality in the state of Michigan. Different practice patterns for managing birthing people with OUD/SUD warrant further exploration to define the most effective, efficient, and patient‐centered models of care to improve outcomes for this population.

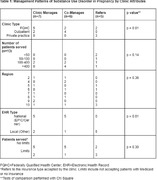

## 528 | Impact of Prolonged Induction of Labor on Maternal and Neonatal Morbidity

Jourdan E. Triebwasser^1^; Evan J. Keil^1^; Rebecca Wardrop^1^; Xilin Chen^1^; Rebecca F. Hamm^2^; Lisa Kane Low^1^; Abigail Ramseyer^3^; Molly J. Stout^4^; Michelle Moniz^1^

*^1^University of Michigan, Ann Arbor, MI; ^2^University of Pennsylvania Perelman School of Medicine, Philadelphia, PA; ^3^University of Michigan Health‐Sparrow, Lansing, MI; ^4^University of Michigan Medical Center, Ann Arbor, MI*

4:00 PM ‐ 6:00 PM

**Objective**: To evaluate the association of prolonged induction of labor (IOL) on severe maternal morbidity (SMM) and severe neonatal morbidity (SNM) among nulliparous, term, singleton, vertex (NTSV) births.

**Study Design**: Retrospective cohort study across 70 hospitals using clinically abstracted data from the Obstetrics Initiative, a quality improvement initiative supported by Blue Cross Blue Shield of Michigan and Blue Care Network. We included NTSV IOL from 01/2022 to 12/2023. Prolonged IOL was defined as duration > 75^th^ percentile for the cohort. Primary outcomes were SMM (Centers for Disease Control criteria) and SNM (unexpected complications in term newborns, PC‐06). Secondary outcomes were postpartum hemorrhage (PPH, blood loss > 1000 mL) and obstetric infection (chorioamnionitis or endometritis). A 1‐to‐1 propensity score‐matched analysis was performed between those with and without prolonged IOL within a hospital using greedy nearest neighbor matching. Adjusted odds ratios (aOR) were calculated adjusting for hospital characteristics (teaching status, higher level neonatal care) to account for hospital‐level clustering. Results were stratified by mode of delivery.

**Results**: Among 29,340 NTSV inductions, the 75^th^ percentile for IOL length was 31 h 47 min. In unadjusted analyses, prolonged IOL was associated with SMM (4.5% vs. 3.0%, p < 0.001), SNM (6.1% vs. 4.0%, p < 0.001), cesarean (50% vs. 28%, p < 0.001), PPH (19% vs. 10%, p < 0.001), and obstetric infection (10% vs. 5.2%, p < 0.001). After propensity score matching (**Table**), prolonged IOL was associated with SMM (aOR 1.43, 95% CI 1.06‐1.92) and SNM (aOR 1.37, 95% CI 1.07‐1.75) among vaginal, but not cesarean births (**Figure**). Prolonged IOL was associated with increased risk of PPH for vaginal (aOR 1.70, 95% CI 1.40‐2.07) and cesarean births (aOR 1.30, 95% CI 1.13‐1.50). Similarly, prolonged IOL was associated with obstetric infection for vaginal (aOR 2.07, 95% CI 1.68‐2.54) and cesarean births (aOR 1.48, 95% CI 1.20‐1.81).

**Conclusion**: Prolonged IOL is associated with maternal and neonatal morbidity, particularly for vaginal births.

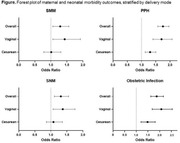

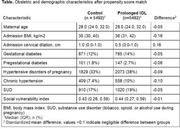

## 529 | Is There a Role for Maternal Oxygen for Fetal Resuscitation in High Risk Gestations?

Julia Burd^1^; Brianna Bradley^1^; Lori Atwood^1^; Nicole McCandless^1^; Paige Wilder^1^; Cookie Van^1^; Jeannie C. Kelly^2^; Antonina I. Frolova^3^; Alison G. Cahill^4^; Nandini Raghuraman^2^

*^1^Washington University in St. Louis, St. Louis, MO; ^2^Washington University School of Medicine in St. Louis, St. Louis, MO; ^3^Washington University School of Medicine, St. Louis, MO; ^4^Dell Medical School Health Transformation Research Institute, Dell Medical School, The University of Texas at Austin, Austin, TX*

4:00 PM ‐ 6:00 PM

**Objective**: Level 1 evidence indicates that maternal oxygen (O2) administration is ineffective for fetal resuscitation in low‐risk patient populations. We investigated whether intrapartum maternal O2 administration benefits patients at high risk of placental insufficiency in labor.

**Study Design**: This is a secondary analysis of two randomized controlled trials at a single center comparing O2 at 10L/min to room air (RA) in term patients with Category II fetal heart tracings in active labor. For this analysis, patients were categorized into high‐ and low‐ risk cohorts, with high‐risk defined as those with fetal growth restriction, chronic hypertension, hypertensive disorders of pregnancy, tobacco use, and/or gestational diabetes requiring medications. The primary outcome was umbilical artery (UA) pH < 7.20. Multivariable logistic regression was used to account for potential confounders. Interaction testing was performed to assess effect modification.

**Results**: 320 patients were recruited across both trials, among which 265 had validated UA pH values and were included. There were no statistically significant demographic or delivery differences between those receiving O2 versus RA in the total cohort or among high‐risk gestations (Table 1). There was no difference in rate of pH < 7.2 between O2 and RA groups in the entire cohort (28.9% vs 23.9%, aOR 1.27 (95% confidence interval 0.73, 2.21). In a stratified analysis assessing pH < 7.2 in high‐ and low‐risk groups, O2 was not associated with a significant reduction in pH < 7.2 in either group, with no evidence of effect modification (p for interaction = 0.97)

**Conclusion**: This secondary analysis suggests that there is no differential benefit for intrapartum maternal O2 supplementation among high risk gestations. This supports ACOG's recommendation to deimplement this practice.

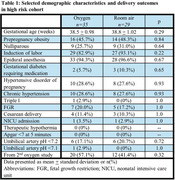

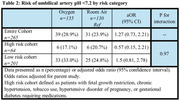

## 530 | Impact of Recipient Or Donor Velamentous Cord Insertion in Patients Undergoing Laser for TTTS

Juliana S. Gebb^1^; Alekhya Jampa^2^; Violetta Bakunina^1^; Shelly Soni^2^; Desiree Fiorentino^2^; Christina Paidas Teefey^2^; Beverly G. Coleman^3^; Julie S. Moldenhauer^2^; Nahla Khalek^2^

*^1^Richard D. Wood, Jr Center for Fetal Diagnosis and Treatment, Children's Hospital of Philadelphia, Philadelphia, PA; ^2^Richard D. Wood Jr. Center for Fetal Diagnosis and Treatment at CHOP, Philadelphia, PA; ^3^Richard D. Wood, Jr Center for Fetal Diagnosis and Treatment, The Children's Hospital of Philadelphia, Philadelphia, PA*

4:00 PM ‐ 6:00 PM

**Objective**: To determine whether recipient or donor velamentous cord insertion (VCI) is associated with perioperative complications and worse outcomes after laser for Twin‐Twin Transfusion Syndrome (TTTS).

**Study Design**: Retrospective analysis of prospectively collected data from a monochorionic (MC) pregnancy registry at a Level III fetal care center. The records of patients with MC twins that underwent laser for TTTS between 1/2013‐1/2024 were reviewed. Pregnancy characteristics and outcomes were then compared between patients that had recipient VCI versus recipient non‐VCI as well as donor VCI versus donor non‐VCI. Statistical analysis included Mann‐Whitney U, chi‐square, and Fisher's exact tests, as appropriate.  

**Results**: 373 patients underwent laser over the study period including 77 (20.6%) with recipient VCI, 92 (24.7%) with donor VCI, and 18 (4.9%) with both recipient and donor VCI. Although postoperative outcomes were similar between groups (Tables 1‐2), patients with recipient VCI had a lower estimated fetal weight (EFW) discordance (13 vs 18, p< 0.001), larger recipient deepest vertical pocket (10.4 vs 9.5, p = 0.004), later gestational age at laser (20.6 vs 19.6, p< 0.001) and lower need for amnioexchange (6.5 vs 17.9%, p = 0.014) compared to those with recipient non‐VCI. On the contrary, patients with donor VCI had higher EFW discordance (23 vs 16, p< 0.001) and earlier gestational age at laser (19.3 vs 20.2, p = 0.001) compared to those with donor non‐VCI.

**Conclusion**: In our cohort, although recipient and donor VCIs were associated with differences in baseline characteristics at laser, they were not associated with worse pregnancy outcomes.

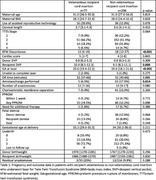

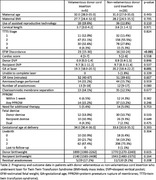

## 531 | Impact of Second Trimester Vaginal Bleeding on Outcomes in Patients Undergoing Laser for TTTS

Juliana S. Gebb^1^; Alekhya Jampa^2^; Violetta Bakunina^1^; Desiree Fiorentino^2^; Christina Paidas Teefey^2^; Shelly Soni^2^; Edward R. Oliver^1^; Julie S. Moldenhauer^2^; Nahla Khalek^2^

*^1^Richard D. Wood, Jr Center for Fetal Diagnosis and Treatment, Children's Hospital of Philadelphia, Philadelphia, PA; ^2^Richard D. Wood Jr. Center for Fetal Diagnosis and Treatment at CHOP, Philadelphia, PA*

4:00 PM ‐ 6:00 PM

**Objective**: To determine whether second trimester vaginal bleeding is associated with perioperative complications and worse outcomes after laser for Twin‐Twin Transfusion Syndrome (TTTS).

**Study Design**: Retrospective analysis of prospectively collected data from a monochorionic (MC) pregnancy registry at a Level III fetal care center. The records of patients with MC twins that underwent laser for TTTS between 1/2013‐1/2024 were reviewed to determine the incidence of second trimester vaginal bleeding prior to the laser procedure. Those without clear documentation of bleeding were excluded. Pregnancy characteristics and outcomes were then compared between patients that had second trimester bleeding versus those that did not using Mann‐Whitney U, chi‐square, and Fisher's exact tests, as appropriate.  

**Results**: Of 373 patients that underwent laser over the study period, 359 (96.2%) had clear documentation of whether they had second trimester vaginal bleeding including 30 (8.4%) that did and 329 (91.6%) that did not, respectively. Although demographic characteristics and survival outcomes were similar between groups, patients with second trimester vaginal bleeding had longer median operating room times (61 vs 50 minutes, p = 0.032), higher need for amnioexchange (33.3 vs 14.3%, p = 0.006), and higher incidence of preterm premature rupture of membranes within 1 week after the laser procedure (16.7 vs 3.3%, p = 0.001).

**Conclusion**: In our cohort, second trimester vaginal bleeding was associated with a higher incidence of perioperative complications surrounding laser for TTTS. This information can be utilized to guide future studies and in counseling patients prior to the laser procedure.

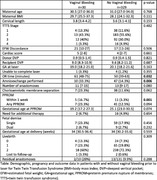

## 532 | Validation of the Delivery Trauma Scale for Evaluation of Emotional Burden Secondary to Birth

Katherine Lambert^1^; Irene A. Stafford^2^; Rafael Bravo Santos^3^; Alicia Lore^4^; Jennifer Tang^4^; Grace Ge^4^; Charles Green^4^

*^1^University of Texas Medical Branch Galveston, Galveston, TX; ^2^McGovern Medical School at UTHealth Houston, Houston, TX; ^3^The University of Texas Health Science Center at Houston (UTHealth), Houston, TX; ^4^University of Texas Health Science Center at Houston, Houston, TX*

4:00 PM ‐ 6:00 PM

**Objective**: The purpose of this study is to assess the validity of the Delivery Trauma Scale (revised City Birth Trauma Scale) in a U.S. population. The aim is to determine if this questionnaire is appropriate for use in assessing emotional burden secondary to birth (previously referred to as postpartum post‐traumatic stress disorder) in the target population and to propose a new name for the questionnaire that better suits the birth and postpartum stress response.

**Study Design**: The study conducted is a methodological non‐interventional study aimed at establishing the validity and reliability of a scale to assess delivery‐related trauma. In the first stage of the study, face validity and content validity were established via expert review of the individual items on the questionnaire and subsequent content validity index analysis. The questionnaire was then administered to women in the postpartum period at least 18 years of age who has delivered an infant within the past 12 months. The construct validity and reliability of the questionnaire was evaluated via statistical analysis using Cronbach's alpha coefficient and confirmatory factor analysis.

**Results**: The initial review of the questionnaire by experts resulted in a content validity index of 1. This can be interpreted as every question being appropriate for and relevant to the evaluation of emotional burden secondary to birth. The Cronbach's alpha coefficient for the questionnaire was calculated to be 0.898 (Bootstrap 95% CI 0.82‐0.93) indicating a very high degree of internal consistency within the questionnaire. Confirmatory factor analysis showed statistical significance of all symptom categories (p< 0.001) with the exception of the category of negative symptoms (Table 1).

**Conclusion**: The Delivery Trauma Scale is validated for use in assessment of emotional burden secondary to birth in a U.S. population, though it should be used cautiously in evaluation for negative symptoms. Overall, the DTS is a promising tool that can be used in the postpartum period to identify patients who may benefit from additional mental health resources and support.

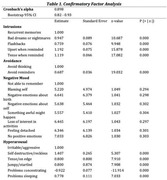

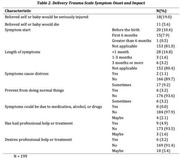

## 533 | Comparison of Fetal Abdominal Circumference References for the Prediction of Sga

Katherine Pressman^1^; Madeline Erwich^2^; Gustavo Vilchez^3^; Anthony O. Odibo^4^; Jose R. Duncan^5^

*^1^Department of Obstetrics and Gynecology, University of South Florida, Tampa, FL; ^2^University of South Florida, University of South Florida, FL; ^3^UTHealth Houston, Houston, TX; ^4^University of Missouri ‐ Kansas City, Leawood, KS; ^5^Department of Obstetrics and Gynecology, University of South Florida, Morsani College of Medicine, Tampa, FL*

4:00 PM ‐ 6:00 PM

**Objective**: Small for gestational age (SGA) infants are at increased risk for adverse neonatal outcomes. Fetal growth restriction (FGR) is defined as estimated fetal weight (EFW) < 10% or abdominal circumference (AC) < 10%.  In the United States, the Hadlock reference is the most widely used method for estimating the fetal AC, however, several other methods exist including the INTERGROWTH‐21st standard and the Chitty standard. We sought to compare the ability of three antenatal AC standards to predict SGA and adverse neonatal outcomes.

**Study Design**: In this secondary analysis of a cohort of singleton gestations that underwent fetal growth assessment between 26 and 36 weeks of gestation, fetuses with chromosomal or congenital malformations and those without delivery information were excluded. The Hadlock, Chitty, and INTERGROWTH‐21st fetal AC methods’ ability to detect SGA and adverse neonatal outcomes were compared by calculating the area under the receiver operating curve of clinical characteristics, sensitivity, specificity, positive predictive value, and negative predictive value.

**Results**: Of 1054 patients, 122 had an AC < 10% by Hadlock, 31 by Chitty, and 50 by INTERGROWTH‐21^st^ (Table 1). The Hadlock definition for AC < 10th was a better identifier for SGA. AUC and 95% CI for SGA for Hadlock, Chitty, and INTERGROWTH‐21^st^ were 0.73 [0.69 ‐0.77] vs 0.59 [0.55 ‐0.61] vs 0.62 [0.59‐0.66]) (p< 0.001), respectively (Figure 1). All AC standards had suboptimal performance for the prediction of adverse neonatal outcomes.

**Conclusion**: The Hadlock AC < 10% was a better predictor than Chitty or INTERGROWTH‐21st for SGA. All references had a suboptimal ability to predict composite adverse neonatal outcomes. The Hadlock reference chart should be utilize in obstetrical practice.

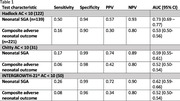

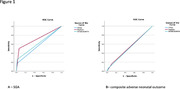

## 534 | Screening Analysis for Autism Spectrum Disorders in FNAIT‐Affected Children with and without Intra‐Cranial Hemorrhage

Katherine A. Knightly^1^; Margaret H. McKelvy^2^; Eleanore A. McFarland^1^; Stephanie V. Volpe^3^; Thea D. Palmer^3^; Stacy Corke^3^; James B. Bussel^1^; Emilie L. Vander Haar^1^

*^1^Weill Cornell Medicine, New York, NY; ^2^Renaissance School of Medicine at Stony Brook University, New York, NY; ^3^NAITbabies, Penzance, England*

4:00 PM ‐ 6:00 PM

**Objective**: Fetal/neonatal alloimmune thrombocytopenia (FNAIT) results from parental platelet antigen incompatibility. De Vos et al. identified increased neurodevelopmental issues in FNAIT‐affected children without ICH. Members of NAITbabies suggested increased autism in FNAIT‐affected children. Our survey evaluated neurodevelopmental issues (autism) in FNAIT‐affected children, with and without ICH.

**Study Design**: A de‐identified survey was distributed via Qualtrics to mothers in NAITbabies and assessed neurodevelopment using 4 age‐specific autism screening scales to identify FNAIT‐affected children at high risk: Q‐CHAT‐10 for 18‐24 months; M‐CHAT 2–4 yrs; AQ10‐Child 4 ‐11; and AQ10‐Adolescent for >12 with 20, 61, 173, and 70 children. Mothers reported if autism diagnosis was known prior to screening and responses were scored using algorithms.

**Results**: There were 324 children across the 4 age groups, 55 with ICH and 269 without. Autism diagnoses were more prevalent in the ICH groups across all ages, except the 18–24‐mo group (no reported autism) see Table 1. Most notably, for 2‐4yr olds, none of 49 children without ICH reported a diagnosis or screened at risk. Among 4‐11yr‐olds, screening showed 33% with ICH and 13% without ICH.  The highest levels were in the 12+ age group, reporting 36.4% with ICH and 11.8% without ICH, with screening identifying autism risk in 45% with ICH and 37% without ICH. Overall, while the ICH group showed more high‐risk screening compared to pre‐reported diagnoses across the 3 older ages, screening totals never exceeded 45% of ICH cases. In the non‐ICH groups, at‐risk screening was seen in > 1/3 of the 12+ age group.

**Conclusion**: Autism and other neurodevelopmental issues become more apparent with age in ICH and non‐ICH groups. The screening tool is NOT a specific diagnostic test for autism and identifies non‐specific neurologic damage. Children affected by FNAIT, especially adolescents, are at risk for “autism” even without ICH. These results indicate the need for periodic screening for autism and neurologic damage in general in children affected by FNAIT, regardless of ICH occurrence.

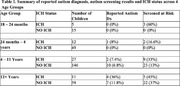

## 535 | Racial Disparities in Severe Maternal Morbidity: Identifying Predictors for Designing Interventions and Risk Reduction

Katherine L. Chen^1^; Mary McLennan^2^; Jennifer A. Bickhaus^2^; Andrew Storm^1^; Samantha Levine^1^; Rachel Hsu^3^; Jason Doherty^1^; Niraj R. Chavan^1^

*^1^Saint Louis University School of Medicine, St. Louis, MO; ^2^Saint Louis University School of Medicine, Department of Obstetrics and Gynecology, St. Louis, MO; ^3^Saint Louis University School of Medicine, St Louis, MO*

4:00 PM ‐ 6:00 PM

**Objective**: To evaluate differences in severe maternal morbidity (SMM) and identify key predictors of SMM among Non‐Hispanic Black (NHB) versus Non‐Hispanic White (NHW) pregnancies

**Study Design**: We undertook a retrospective cohort study of all patients receiving prenatal care and delivering at a single academic tertiary medical center from 2017–2013. The cohort was divided based on self‐identified race into 2 subgroups ‐ i.e. NHB vs NHW. Our primary outcome was SMM as defined by the Centers for Disease Control (CDC). Demographic and clinical characteristics were abstracted from electronic records and compared across study groups using Student's T test and chi2/Fischer's exact test for continuous and categorical data, respectively. The Kruskal Wallis test was used to compare nonparametric data. Multivariable regression analyses were used to identify the key predictors of SMM in each cohort.

**Results**: Of the 6403 patients, 4200 (66%) identified as NHB, while 2203 (34%) identified as NHW. NHB patients were significantly younger (p< .01), had governmental insurance (p< .01) with a higher BMI (p< .01), and presented at later gestational age at prenatal care initiation (p< .01). Overall, 417/6403 (7%) of the cohort had a SMM event with a higher incidence of SMM among NHB patients as compared to their NHW counterparts [307/4200 (7%) vs 110/2203 (5%), p< .05]. Rates of individual SMM events were similar across both groups except for higher rates of sickle cell disease with crisis (p = .02) and hemorrhage (p< .01) in NHB Patients. Among NHB patients–gestational hypertension [aOR: 1.61 (1.10–2.330], cesarean delivery [aOR: 2.82 (2.17–3.67)] and gestational age at delivery [aOR:1.87 (1.13–2.99)] were identified as key predictors of SMM. In NHW patients–gestational hypertension [aOR: 3.73 (1.88–7.16) and cesarean delivery [aOR: 3.71 (2.36–5.94)] had the highest impact on SMM risk.

**Conclusion**: Key clinical predictors of SMM were identified in our study with a differential risk impact among NHB and NHW patients as an opportunity for risk reduction and maternal mortality prevention.

## 536 | Epidural Placement Early in Cervical Ripening: is There a Negative Impact?

Katie Brow^1^; Nandini Raghuraman^2^; Alison G. Cahill^3^; Candice Woolfolk^4^; Sydney M. Thayer^5^; Jeannie C. Kelly^2^; Antonina I. Frolova^1^; Julia Burd^5^

*^1^Washington University School of Medicine, St. Louis, MO; ^2^Washington University School of Medicine in St. Louis, St. Louis, MO; ^3^Dell Medical School Health Transformation Research Institute, Dell Medical School, The University of Texas at Austin, Austin, TX; ^4^Washington University School of Medicine in Saint Louis, St. Louis, MO; ^5^Washington University in St. Louis, St. Louis, MO*

4:00 PM ‐ 6:00 PM

**Objective**: National guidelines recommend epidural anesthesia be offered at any point in labor. At our institution, patients undergoing induction of labor (IOL) often request “early” epidurals during cervical ripening or prior to mechanical ripening. Our objective was to evaluate if early epidural placement during IOL impacts labor duration.

**Study Design**: This is a secondary analysis of a prospective cohort study of term patients from a single institution. We included all patients who presented for IOL and received either a prostaglandin or mechanical cervical ripening. Patients with history of cesarean and those without an epidural were excluded. Patients were divided into two groups based on the exam prior to epidural placement: early epidural (EE) if < 2 cm and late epidural (LE) if > 2 cm. Analysis was stratified by parity. The primary outcome was length of IOL, defined as interval between first cervical exam and delivery. Secondary outcomes were mode of delivery, intraamniotic infection, 3^rd^/4^th^ degree lacerations, and neonatal outcomes. Time to event analysis was performed and the log‐rank test was used to compare the primary outcome between groups. Cox proportional hazards modeling was used to adjust for potential confounders.

**Results**: 1704 patients were included with 816 (48%) in the EE group and 888 (52%) in the LE group. Median Bishop score on admission was 1 for both groups. IOL duration was significantly longer in patients with EE (22.7 hours vs 19.9 hours in nulliparous and 15.9 hours vs 14.8 hours in multiparous, both P< 0.001) (Figure). There was no difference in second stage duration between groups. After adjusting for confounders, there was no association between timing of epidural and rates of cesarean delivery, operative vaginal delivery, intraamniotic infection, 3^rd^/4^th^ degree lacerations, or neonatal outcomes (Table).

**Conclusion**: Early epidural placement < 2 cm is associated with a statistically longer total duration of IOL with an absolute difference of no more than 2‐3 hours. Early epidural is not associated with adverse maternal or neonatal outcomes or a longer second stage of labor.

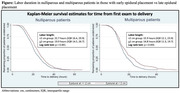

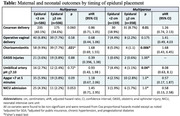

## 537 | Maternal Covid‐19 and Placental Expression of Senescence‐Associated Secretory Phenotype Genes

Kimen S. Balhotra^1^; Katherine B. Le^2^; Rachel Keuls^3^; Natalie N. Lanners^2^; Tina Findley^2^; Ron Parchem^3^; Jacqueline G. Parchem^2^

*^1^McGovern Medical School at UTHealth, Houston, TX; ^2^McGovern Medical School at UTHealth Houston, Houston, TX; ^3^Baylor College of Medicine, Houston, TX*

4:00 PM ‐ 6:00 PM

**Objective**: Maternal COVID‐19 increases the risk for adverse pregnancy outcomes related to placental dysfunction, including preeclampsia and stillbirth, however, the biologic basis remains underexplored. Based on recent work showing the association between placental senescence and preeclampsia, we hypothesized that senescence‐associated secretory phenotype (SASP) genes are dysregulated in COVID‐19 placentas.

**Study Design**: We analyzed a single‐nucleus RNA sequencing dataset of placentas from symptomatic COVID‐19 patients at birth (n = 4) and gestational age‐matched controls (n = 4) to characterize the response of different trophoblast populations to COVID‐induced stress. Differentially expressed gene (DEGs) in COVID‐19 v. controls were identified for villous syncytiotrophoblasts (STB), membrane extravillous trophoblasts (EVT‐M), and membrane cytotrophoblasts (CTB‐M) using the Seurat (v4.3.0) in R given that these populations are likely sources of circulating placental proteins. Our analysis focused on DEGs belonging to a previously defined set of SASP genes.

**Results**: The baseline characteristics for COVID‐19 patients and control groups were similar for maternal age (26 vs 28 years‐old), gestational age (36 vs 38 weeks) and BMI (37 vs 34 kg/ m2). DEG analysis showed upregulation of distinct SASP pathway genes in STB, EVT‐M, and CTB‐M populations. Follistatin‐like 3 (FSTL3) was significantly upregulated in COVID‐19 samples across all cell populations, but predominantly expressed in EVT‐M and CTB‐M(P ≤ 0.0001)(Figure 1). In contrast, activin A (INHBA) was only upregulated in the STB and EVT‐M populations, but predominantly expressed in STBs (P ≤ 0.0001)(Figure 2).

**Conclusion**: Compared with control placentas, trophoblast populations from COVID‐19 positive patients showed an increased expression of senescence genes. Our data reveals cell type‐specific expression of senescence genes, suggesting that SASP proteins in the maternal circulation have different sources. This increased expression of senescence genes among COVID‐19 positive patients may provide potential insight into the link between COVID‐19 and preeclampsia.

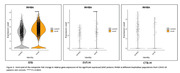

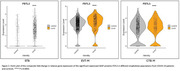

## 538 | The Association Between Hepatitis B Virus Infection During Pregnancy and Risk of Primary Cesarean Delivery

Kriti N. Vedhanayagam^1^; Stephen Contag^2^; Ilish Gedestad^3^; Sergio Karageuzian^4^; Rang Kim^5^; Synia Chunn^1^; Megan Marquez^5^; Ruofan Yao^1^

*^1^Loma Linda University School of Medicine, Loma Linda, CA; ^2^University of Minnesota, Minneapolis, MN; ^3^Loma Linda University School of Medicine, Redlands, CA; ^4^Loma Linda University School of Medicine, Pasadena, CA; ^5^Loma Linda University School of Medicine, Loma Linda University, CA*

4:00 PM ‐ 6:00 PM

**Objective**: To investigate the association between hepatitis B virus (HBV) status and the likelihood of primary cesarean section (C‐section) delivery compared to vaginal delivery. 

**Study Design**: This retrospective cohort study used data from the National Center for Health Statistics Vital Statistics database. The cohort linked birth and infant death data files from 2015 to 2021 was used for this study. The study included all births in the United States during the study period, with known HBV status and without prior Cesarean Delivery (CD). The outcome of interest was mode of delivery. Univariate analysis was performed to determine the association between HBV and CD rate. Multivariate logistic regression was then performed to estimate the effect of HBV status on CD adjusting for potential confounding variables. Interaction analysis was then performed to determine if the effect of HBV on CD is modified by race and obesity.

**Results**: The rate of CD was higher in pregnancies complicated by HBV infection compared to uninfected individuals (22.4% vs 21.9%, p = 0.004).The effect of HBV on CD is modified by both race and obesity. Specifically, HBV infection increases risk of primary CD (aOR 1.39; 95% CI [1.25‐1.54]). Compared to White, both Black and Asian race demonstrated a protective effect on primary CS among individuals with HBV infection (aOR 0.93; 95% CI [0.87 ‐ 0.99] and (aOR 0.74; 95% CI [0.85‐0.79]). Compared to underweight group, individuals in the higher weight groups complicated by HBV infection also demonstrated a protective effect on primary CD.

**Conclusion**: This study demonstrated that HBV infection increased the risk of primary CD in otherwise uncomplicated pregnancies. However, in pregnancies complicated by racial categories and high BMI groups with already increased risk of primary CD, HBV infection demonstrated a protective effect.

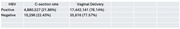

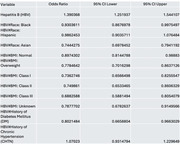

## 539 | How Much is Enough? Incomplete Treatment Course of Intravenous Iron to Prevent Postpartum Blood Transfusions

Lama R. Noureddine^1^; Miranda Hawthorne^1^; Chavi Eve Karkowsky^2^; Jessica Greenberg^3^; Lisa N. Gittens‐Williams^1^; Joseph J. Apuzzio^1^; Shauna F. Williams^1^

*^1^Rutgers New Jersey Medical School, Newark, NJ; ^2^Rutgers New Jersey Medical School, Rutgers NJMS, NJ; ^3^Rutgers New Jersey Medical School, South Orange, NJ*

4:00 PM ‐ 6:00 PM

**Objective**: To compare the benefit of partial and full doses of IV iron treatment in reducing postpartum blood transfusions.

**Study Design**: This was a retrospective cohort study of patients with Iron Deficiency Anemia (IDA) who presented for prenatal care at a tertiary urban academic center from June 2020 to June 2024. Women were included if they received IV iron antepartum and excluded if they had contraindications to blood transfusion. The primary outcome was postpartum blood transfusion. Secondary outcomes included hemoglobin (Hg) levels at admission and percent change in Hg (pre‐treatment to admission). Fisher's exact, chi square, parametric and non‐parametric tests were used. Median (interquartile range) and mean +/‐ standard deviation shown.

**Results**: Among 224 patients with IDA, 116(52%) completed the calculated IV iron dose based on the iron deficit (Full dose, Group 1) and 108(48%) initiated, but did not complete the prescribed treatment (Partial dose, Group 2). The median dose of IV iron was 900 mg (400‐1800) in Group 1 and 400 mg (200‐1200) in Group 2 (p< 0.0001). Pre‐treatment Hg was similar between Groups 1 and 2 (8.7 +/‐ 0.89 vs 8.6 g/dL +/‐ 0.92 respectively, p = 0.71). However, Group 2 had more short interval pregnancies [8(7%) vs 24(22%) in Groups 1 and 2, p = 0.001], later gestational age at treatment initiation [median 34(20‐38) vs 36 weeks (24‐40), p< 0.0001] and a higher median delivery blood loss [285(200‐543) vs 361 mL(200‐612), p = 0.04]. There was no difference in postpartum transfusions between Groups 1 and 2 [8(7%) vs 11(10%), p = 0.33]. However, Group 1 had a higher mean admission Hg than Group 2 (10.8 +/‐ 0.92 vs 9.7 g/dL +/‐ 0.98, p< 0.0001), a higher median percent increase in Hg [25% (12‐36) vs 11% (4‐21), p< 0.0001], and a higher mean postpartum day 1 Hg (9.8 +/‐ 1.18 vs 8.3 +/‐ 1.19 g/dL, p< 0.0001). 

**Conclusion**: A full course of IV iron improved Hg response and admission Hg compared to partial course but did not significantly decrease postpartum blood transfusions. Partial treatment still benefits pregnant patients with anemia, even when optimal treatment cannot be completed.

## 540 | Creating Optimal Pain Management FOR Tailoring (COMFORT) Guidelines for Pain Management: Postpartum Recommendations

Alex Peahl^1^; Laura Peyton Ellis^2^; Courtney Townsel^3^; Lisa Kane Low^4^; Jennifer Waljee^4^; Mark Bicket^4^; Dana Feldman^5^; Bryan L. Aaron^4^; Michelle Moniz^4^

*^1^Michigan Medicine, Ann Arbor, MI; ^2^UConn Health Obstetrics and Gynecology, Farmington, CT; ^3^University of Maryland Department of Obstetrics, Gynecology and Reproductive Sciences, Baltimore, MD; ^4^University of Michigan, Ann Arbor, MI; ^5^Wake Forest University, Winston‐Salem, NC*

4:00 PM ‐ 6:00 PM

**Objective**: To develop a new clinical practice guideline for postpartum pain management that promotes opioid stewardship, patient‐centeredness, and health equity.

**Study Design**: We developed the COMFORT (Creating Optimal Pain Management FOR Tailoring pain management after surgery in pregnancy and childbirth) guideline using a rigorous, evidence‐based, eDelphi process, the RAND‐UCLA Appropriateness Method. After a systematic review, we identified 19 procedures, 6 populations, and 5 postpartum pain management strategies. Interprofessional panelists (n = 17) and public members (n = 3) rated the appropriateness of pain management strategies in 3 eDelphi rounds. Two consensus conferences were held between rounds with panelists and public members to resolve discrepancies and ensure public contributions. Quantitative ratings were assessed using basic statistics and the RAM definitions for consensus. Key findings from the panel discussion were summarized using a qualitative content analysis and matched to quantitative findings.

**Results**: Key panel recommendations for postpartum patients include: 1) provide robust patient education on postpartum pain management and opioid risk‐reduction; 2) use scheduled non‐opioid medications (e.g., acetaminophen, Non‐steroidal Anti‐Inflammatory Drugs); 3) utilize non‐pharmacologic strategies as additive pain management; 4) utilize alternative inpatient strategies for individuals with complex pain; 5a) use judicious opioid prescribing as needed; 5b) utilize tailored prescription benchmarks to determine appropriate opioid prescription sizes for shared decision making (Table 1).

**Conclusion**: New guidelines for pain management after surgery in pregnancy and after childbirth may standardize care, reduce risks of opioid prescribing, and improve patient‐centeredness and equity.

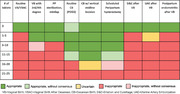

## 541 | Preconception and First Trimester Blood Pressure: Risks of Hypertensive Disorders in Pregnancy Using AHA Guidelines

Lior Heresco^1^; Tal Biron‐Shental^2^; Tzipi Hornik‐Lurie^3^; Ella Pardo^1^; Gil Shechter Maor^1^; Michal Kovo^1^

*^1^Meir Medical Center, Kfar Saba, HaMerkaz; ^2^Meir Medical Center, Meir Medical Center, HaMerkaz; ^3^Data research department at the Research Authority, Meir Medical Center, Kfar Saba, HaMerkaz*

4:00 PM ‐ 6:00 PM

**Objective**: Hypertensive disorders during pregnancy (HDP) significantly contribute to maternal morbidity. We aimed to assess the risk of HDP according to the 2017 American Heart Association AHA criteria based on preconception and early pregnancy blood pressure (BP) measurements.

**Study Design**: This retrospective study utilized the Clalit Health Services database, and included all singleton pregnancies from 2010 to 2023 with at least one BP measurement within one year before conception until 13 weeks of gestation. Participants were classified into three groups according to the AHA guidelines:normotensive, elevated BP (systolic BP [SBP] of 120‐129 mm Hg with diastolic BP [DBP] < 80 mm Hg), and stage 1 hypertension (SBP of 130‐139 mm Hg or DBP of 80‐89 mm Hg). Patients with chronic hypertension were excluded. Maternal, neonatal, and composite hypertensive outcomes were compared between the groups. Subgroup analyses were conducted for patients with obesity (BMI ≥ 30) and those with pre‐gestational diabetes mellitus (PGDM). A logistic regression model adjusted for maternal age, aspirin use, BMI, and PGDM was utilized.

**Results**: The study included 57,524 women. Of these, 46,924 (81.6%) were normotensive, 6,636 (11.5%) had elevated BP, and 3,964 (6.9%) had stage 1 hypertension. The incidence of composite hypertensive adverse outcome increased in a dose‐response manner: 2.8% in the normotensive group, 6.4% in the elevated BP group, and 8.2% in the stage 1 hypertension group (p< 0.001). Neonatal NICU admissions were more common in the elevated BP and stage 1 hypertension groups compared to the normotensive group (p< 0.001). Logistic regression revealed that elevated BP, stage 1 hypertension, maternal age ≥35 years, and PGDM were independently associated with increased odds of composite hypertensive outcome: OR 2.22 (95% CI 1.94‐2.55), OR 2.75 (95% CI 2.34‐3.24), OR 1.88 (95% CI 1.57‐2.27), and OR 1.55 (95% CI 1.29‐1.86), respectively (p< 0.001 for all).

**Conclusion**: Preconceptional and first‐trimester elevated BP and stage 1 hypertension, as defined by the AHA guidelines, are associated with an increased risk of maternal HDP.

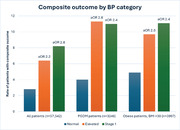

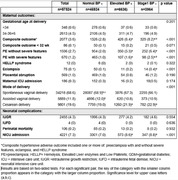

## 542 | Standardized Clinical Assessment and Management Plan Improves Neonatal Outcomes in prenatally suspected Congenital Heart Disease

Lior Kashani Ligumsky; Angela Desmond; Guadalupe Martinez; Joanne Newens; Deborah Krakow; Gary Satou; Yalda Afshar

*University of California, Los Angeles, Los Angeles, CA*

4:00 PM ‐ 6:00 PM

**Objective**: Prenatal diagnosis of congenital heart disease (CHD) has been associated with early‐term and cesarean births. We implemented a standardized clinical assessment and management plan (SCAMP) aimed at reducing early‐term and cesarean births. We aimed to evaluate the SCAMP's impact on neonatal outcomes focusing on survival at discharge rate and birthweight in pregnancies complicated by fetal CHD.

**Study Design**: Neonatal data from historical and intervention cohorts before and after SCAMP implementation were analyzed. Neonates with complete data on birth mode, birthweight, and survival at discharge were included. The primary outcome was survival at discharge. Secondary outcomes included birthweight and survival differences based on birth mode (vaginal birth, IOL, and cesarean birth). Statistical comparisons used t‐tests for continuous and chi‐square tests for categorical variables.

**Results**: A total of 424 pregnancies were included in the study, comprising 177 historical pregnancies and 247 intervention cohort pregnancies. Mean birthweight increased from 2808g in the historical cohort to 2967g in the post‐SCAMP intervention cohort (p = 0.02). The overall survival rate to discharge was higher in the intervention cohort (91.1%) compared to the historical cohort (83.1%) (p = 0.012). In the cesarean birth group, survival rates were higher in the intervention cohort (89.1%) compared to the historical cohort (76.1%) (p = 0.02). There were no significant differences in survival rates for induction of labor and spontaneous births between the historical and intervention cohorts (p = 0.8 and p = 0.1, respectively).

**Conclusion**: The implementation of a SCAMP focused on reducing early‐term and cesarean birth significantly improved neonatal outcomes, notably increasing neonates with CHD survival rates at discharge. This improvement was most pronounced in cesarean births. Clinical pathways focused on the prenatal course of pregnancies affected by CHD improve neonatal outcomes.

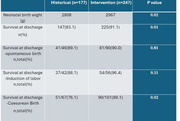

## 543 | Predictive Modeling for Antenatal Shoulder Dystocia Risk

Lior Heresco^1^; Noa Levy^2^; Omer Todress^2^; Tal Biron‐Shental^3^; Omer Weitzner^1^

*^1^Meir Medical Center, Kfar Saba, HaMerkaz; ^2^Department of Biomedical Engineering, Faculty of Engineering, Tel Aviv University, Israel, Tel Aviv, Tel Aviv; ^3^Meir Medical Center, Meir Medical Center, HaMerkaz*

4:00 PM ‐ 6:00 PM

**Objective**: Shoulder dystocia (SD) represents a significant complication during delivery, posing substantial risks. Despite its challenging predictability, assessing individual risk is crucial for informed counseling on optimal delivery methods. The objective of this study was to develop and validate a model prediction system for SD using fetal ultrasound and maternal data.

**Study Design**: Data was retrospectively obtained from deliveries in Meir Hospital between 2014 and 2023.The inclusion criteria were singleton pregnancies and vaginal deliveries. The features included in the tested models were those that had a small percentage of null values in the SD cases. In cases of null values in normally distributed features, we completed the data with mean values. The final model parameters included maternal age, BMI, obstetric history, maternal height, gestational or pre‐gestational diabetes, gestational age at delivery, clinical and sonographic estimated fetal weight, fetal gender, and mode of delivery (vaginal or operative vaginal). To address imbalanced data, we used repeated random sampling of the negative cases. The training and test sets were split in a 70:30 ratio and standardized.

Several classifiers, including logistic regression, decision tree, random forest, support vector machine, XGBoost, and CatBoost, were evaluated using cross‐validation and area under the ROC Curve (AUC). We chose the model based on the highest mean AUC. The analysis was conducted in Python using Pandas, Scikit‐learn, Numpy, and visualization libraries.

**Results**: A total of 51,628 deliveries were analyzed, including 94 SD cases. SD patients had higher rates of diabetes (13% vs. 4.8%) and obesity (23% vs. 5%) compared to non‐SD patients, and higher mean neonatal birthweight (3751 g vs. 3287 g). The highest mean AUC was achieved using the CatBoost model, which demonstrated high discriminatory ability in predicting SD (AUC = 0.83).

**Conclusion**: The SD prediction model presented in this study serves as a valuable adjunct for clinical decision‐making regarding the appropriate mode of delivery.

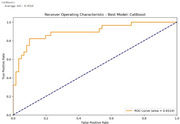

## 544 | Do Maternal Attributes Including Clinical Risk and Antenatal Services Predict Postpartum Care by 6 Months?

Lisbet S. Lundsberg; Caitlin Partridge; Olivia Paoletti; Jennifer F. Culhane

*Yale School of Medicine, New Haven, CT*

4:00 PM ‐ 6:00 PM

**Objective**: Adequate postpartum care (PPC) is critical to overall maternal health, however rates of PPC utilization in the United States are suboptimal.  To better understand factors related PPC engagement, we sought to broadly examine maternal attributes including clinical risk, antenatal services, labor and delivery outcomes, and their association with 6‐month PPC.

**Study Design**: Retrospective cohort analysis of patients delivering in a hospital system 2013‐2022 with at least 1 documented prenatal care (PNC) visit.  We identified PPC visits by examining electronic medical record (EMR) data for ICD codes for any PPC engagement within 6 months across all specialty departments, and traditional PPC defined by ICD codes Z39 and V24.  Patient socio‐demographics, antenatal clinical diagnoses, measures of PNC service utilization, and maternal/neonatal outcomes were examined by PPC engagement using chi‐square or ANOVA.  Multivariable logistic regression was performed including all variables p< 0.05 in bivariate tests.   Adjusted odds ratios (aOR) and 95% CI are presented in Table 1.

**Results**: Among 60,933 deliveries, 90.3% and 78.3% had any PPC and traditional PPC, respectively. PPC engagement at 6 months varied by many patient attributes.  Following multivariable adjustment, significantly reduced odds of any PPC and traditional PPC were observed among patients with the following attributes: multiparous, smoking, single relationship status, public insurance, gestational diabetes (GDM), and substance use disorder (SUD). In contrast, significantly increased likelihood of PPC was demonstrated among patients with planned or intrapartum cesarean, preterm birth, chronic hypertension (cHTN), depression or anxiety, influenza vaccination, and higher number of PNC visits.

**Conclusion**: Although the overall rate of PPC is high in this diverse cohort, patients with disparity‐related risk demonstrate significantly lower rates of PPC.  These data can inform targeted antenatal and delivery admission encounter interventions to optimize PPC with the goal of improving maternal health.

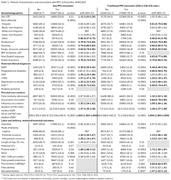

## 545 | Patient‐Reported Unmet Health‐Related Social Needs in Patients with and Without Gestational Diabetes

Logan Mauney^1^; Jonathan Y. Siden^2^; Kaitlyn E. James^1^; Chengbo Zeng^3^; Camille E. Powe^1^; Sarah N. Bernstein^4^

*^1^Massachusetts General Hospital, Boston, MA; ^2^Massachusetts General Hospital, Harvard Medical School, Boston, MA; ^3^Brigham & Women's Hospital, Boston, MA; ^4^Massachusetts General Hospital, Division of Maternal Fetal Medicine, Boston, MA*

4:00 PM ‐ 6:00 PM

**Objective**: Gestational diabetes (GDM) is a common pregnancy complication associated with social determinants of health (SDOH) including economic stability, social and community context and neighborhood environment. Little is known regarding the impact of patient‐level manifestations of SDOH, termed unmet health‐related social needs (HRSNs), on GDM. We sought to characterize the impact of patient‐reported HRSNs on GDM.

**Study Design**: Retrospective cohort study of pregnant patients that completed a validated electronic screening tool at least one time for HRSNs during pregnancy in a regional health‐care system from 5/2020 to 3/2024. Patients with pre‐existing diabetes or multiple gestation were excluded. HRSNs were compared between patients with and without GDM by ICD‐10 codes at delivery. Generalized estimating equations were generated to determine adjusted odds ratios (aOR) for the association between HRSNs and GDM, adjusting for patient characteristics known to be associated with GDM.

**Results**: 2,024 patients met inclusion criteria, of whom 174 (8.6%) had GDM. The GDM cohort was more likely to be older, Black or Asian, multiparous, and to have obesity. Compared to patients without GDM, patients with GDM were more likely to have food insecurity (8.6% vs. 3%) and transportation barriers (13.8% vs. 8.1). 77 patients with GDM (44.3%, p = 0.03) had ≥ 1 HRSN in any domain. In logistic regression models controlling for patient characteristics, transportation barriers (aOR 4.3, 95% CI 2.18‐8.50), food insecurity (aOR 1.95, 95% CI 1.14‐3.34), and ≥ 3 HRSN domains (aOR 1.93, 95% CI 1.19‐3.13) were significantly associated with GDM (Table 1). Other HRSNs represented in the cohort (Figure 1) were not significantly associated with risk of GDM (Table 1).

**Conclusion**: Multiple HRSNs are risk factors for GDM, and the effect size of transportation barriers was on par with that of obesity. Further research is needed to understand the impact of these HRSNs on the development of GDM so appropriate primary prevention tools on a patient‐ and community‐level can be developed to optimize birth outcomes.

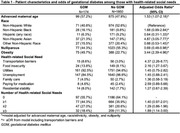

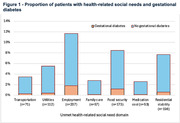

## 546 | Influence of Preconception and Pregnancy Exposure to Marijuana on Adverse Perinatal Outcomes

Madison Blount^1^; Neil B. Patel^2^; Emily A. DeFranco^3^

*^1^The University of Kentucky College of Medicine, Lexington, KY; ^2^Ascension Sacred Heart ‐ Pensacola, Pensacola, FL; ^3^Department of Obstetrics and Gynecology, University of Kentucky, Lexington, KY*

4:00 PM ‐ 6:00 PM

**Objective**: Marijuana consumption during pregnancy has been associated with adverse perinatal outcomes. However, whether exposure during the preconception period also poses a risk is unknown.  

**Study Design**: Retrospective cohort utilizing data from PRAMS Phase 8 Core Questionnaire and the Marijuana & Prescription Drug Supplement from 2017‐2021. The association between marijuana use 3 months prior to pregnancy, during pregnancy, and during both periods and pregnancy outcomes were observed. The primary outcome was fetal growth restriction (FGR) < 10%ile. Other outcomes examined were severe FGR, preterm birth <37 weeks, low birth weight, infant mortality, and NICU admission. Generalized linear modeling estimated the associations while adjusting for coexisting risks of maternal race, tobacco use, education status, obesity, and parity.  

**Results**: Of 17,882 postpartum individuals surveyed, 88.7% reported no marijuana consumption, 5.4% reported use prior to pregnancy only, 0.1% during pregnancy only, and 5.9% preconception and during pregnancy. Marijuana consumption prior to and during pregnancy was associated with a 30% increased risk of FGR and severe FGR, even after accounting for tobacco use and other risk factors (Table 2). The rate of infant mortality also increased but was not statistically significant. Cessation of marijuana use after the preconception period was not associated with an increased risk of adverse perinatal outcomes.

**Conclusion**: Marijuana use before and during pregnancy is associated with increased risk of adverse perinatal outcomes, even after adjusting for confounding risks. However, exposure during only the pregestational period was not. This highlights the importance of counseling patients regarding the risks of marijuana use during pregnancy and the importance of cessation

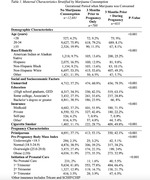

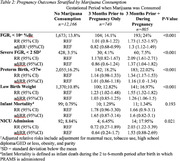

## 547 | Temporal Trends in Gonorrhea and Chlamydia Infections During Pregnancy: 2013‐2023

Marcia Des Jardin^1^; Christina T. Blanchard^2^; Victoria C. Jauk^1^; Lynda Ugwu^1^; Jodie A. Dionne^1^; Akila Subramaniam^2^

*^1^University of Alabama at Birmingham, Birmingham, AL; ^2^Center for Women's Reproductive Health, University of Alabama at Birmingham, Birmingham, AL*

4:00 PM ‐ 6:00 PM

**Objective**: Gonorrhea (GC) and chlamydia (CT) infection during pregnancy is increasing nationally. We evaluated the incidence of GC and/or CT infection in pregnancy as well as risk factors for infection over the past 11 years at single center.

**Study Design**: Retrospective study of all pregnant patients who delivered between 2013‐2023 at large urban tertiary care center in Alabama. The primary outcome was the incidence of infection with GC, CT, or both (GC/CT co‐infection) identified during pregnancy by nucleic acid amplification testing; secondary outcomes included rates of treatment, time to treatment, and reinfection rates. Outcomes were compared across the study period with linear regression to assess trends. We secondarily compared baseline characteristics between individuals with and without GC and/or CT infection using multivariable logistic regression. Tests of heterogeneity (interaction p‐values) were conducted by year to assess different effects in these risk factors over the study period.

**Results**: Over the study period, 40,139 patients were analyzed (no exclusions) ‐ 1.8% had GC, 6.6% CT, and 0.8% both. Rates of infection (GC, CT, and both) significantly decreased over time (Figure, all p< 0.001). Rates of treatment were high (range 58.4%‐71.7%), reinfection low (6.2%‐12.9%), and did not demonstrate trends across the study period (p >0.05). Of note, time to treatment significantly decreased over the study period from a median of 5 days (IQR 0‐10) to 2 days (0‐4) (p< 0.001).  Multiple risk factors were associated with infection in multivariable analyses (Table). However, over time, there were only increasing risks of infection based on non‐White/Black/Hispanic race/ethnicity (p‐interaction = 0.02) and government insurance (p‐interaction = 0.003).  

**Conclusion**: Contrary to national reports in pregnant individuals, at our urban institution, we describe decreasing rates of GC and/or CT infection. Risk factors in our population are consistent with the literature. Further larger studies to confirm or refute our findings, and identify risk factors in populations with rising infection rates should be performed.

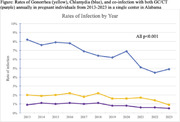

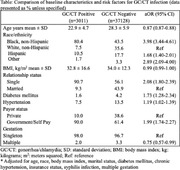

## 548 | Association of Perinatal Inflammation and Breastfeeding Intensity

Margaret S. Butler^1^; Lauren S. Keenan‐Devlin^2^; Ann EB Borders^3^; Britney P. Smart^4^; Janedelie Romero^5^

*^1^Center of Excellence in Maternal and Child Health, Chicago, IL; ^2^Endeavor Health/ University of Chicago Pritzker School of Medicine, Endeavor Health, IL; ^3^Endeavor Health, Evanston Hospital, Evanston, IL; ^4^Sinai Chicago, Chicago, IL; ^5^Endeavor Health, Evanston, IL*

4:00 PM ‐ 6:00 PM

**Objective**: Higher systemic inflammation has been associated with lower rates of breastfeeding, but this has not been measured across multiple perinatal timepoints. We aimed to identify whether elevated inflammatory cytokines in blood during pregnancy and postpartum were associated with lower breastfeeding intensity.

**Study Design**: The Stress, Pregnancy, and Health (SPAH) observational study included 605 participants enrolled mid‐gestation with singleton pregnancies. Participants completed surveys and antecubital blood draws between 20‐27 and after 32 weeks gestation. Clinical outcomes were abstracted from the medical record. A sub‐group of SPAH participants additionally completed surveys at 1 week, 6 weeks, 3 months, and 6 months postpartum as well as an additional blood draw at 6 weeks. Breastfeeding intensity was self‐reported at all 4 surveys, and cytokines IL‐1RA, IL‐6, IL‐10, and TNF‐α, and C‐reactive protein were measured in serum. Inflammation markers were log‐transformed for normality and z‐scored, and z‐scores were summed into composites. Stepwise regression identified key covariates, which were retained in multiple regression models.

**Results**: 155 individuals were included in the analysis. As indicated by the stepwise regression, we retained marital status, SES, and any diabetes in prenatal models and gestational weeks at delivery, any diabetes, and hypertensive disorders of pregnancy, last BMI measured during pregnancy, and resource composite score in postpartum models. Adjusted models indicated that lower circulating inflammatory markers during pregnancy were associated with higher breastfeeding intensity across week 6, month 3, and month 6 time points. The postpartum inflammatory composite was associated with breastfeeding intensity at point of blood draw (6 weeks).

**Conclusion**: Lower prenatal inflammatory markers were associated with higher rates of breastfeeding in the postpartum period, but postpartum inflammatory markers were not. Inflammation in pregnancy may influence lactogenesis and breastfeeding initiation, differentiating breastfeeding outcomes in the postpartum period.

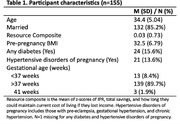

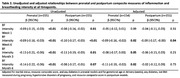

## 549 | Physiologic Treatment of Mild Chronic Hypertension and Pregnancy Outcomes

Mariam Ayyash^1^; Ayodeji Sanusi^2^; On behalf of The CHAP Study Consortium

*^1^Columbia University Irving Medical Center, New York, NY; ^2^Center for Women's Reproductive Health, University of Alabama at Birmingham, Birmingham, AL*

4:00 PM ‐ 6:00 PM

**Objective**: Among patients with hypertension (HTN) in pregnancy, emerging research suggests that improved blood pressure (BP) control can be achieved if the antihypertensive drug used accounts for the underlying maternal hemodynamic physiology. We evaluated whether physiologic treatment of mild chronic HTN is associated with a reduction in adverse pregnancy outcomes.

**Study Design**: We conducted a secondary analysis of CHAP, an open‐label, multicenter, randomized trial of antihypertensive treatment (vs. none) for pregnant patients with mild chronic HTN. Patients with BPs≥160/105 were excluded from CHAP trial. Eligible participants for this analysis had either hyperdynamic– a pulse pressure (PP)≥65, or vasoconstrictive– diastolic BP≥100 physiology. Three groups were compared based on treatment of BP at randomization: 1) Physiologic‐ hyperdynamic receiving Labetalol or vasoconstrictive receiving Nifedipine; 2) Non‐physiologic‐ hyperdynamic receiving Nifedipine or vasoconstrictive receiving Labetalol, and 3) control‐ not on medications. The primary outcome was a composite of superimposed preeclampsia with severe features, preterm birth before 35 weeks’ gestation, placental abruption, or fetal or neonatal death. Adjusted odds ratios (aORs) and 95% CIs were estimated with logistic regression.

**Results**: Of 2408 in CHAP, 542 (23.3%) participants met physiologic classification criteria and were analyzed: 136 (25.1%) had physiologic treatment, 125 (23.0%) had non‐physiologic treatment, with 281 (51.8%) untreated controls. The primary outcome did not differ among those who received physiologic treatment (44.9%), non‐physiologic (45.6%), or control (48.4%) with aOR (CIs) for physiologic vs control 0.85 (0.56‐1.30), non‐physiologic vs control 0.90 (0.59‐1.39), and physiologic vs non‐physiologic 0.95 (0.58‐1.56).

**Conclusion**: Physiologic treatment of mild chronic HTN was not associated with a significant reduction in primary composite adverse outcome. Most patients did not meet criteria for physiologic classification. Further research using different thresholds to account for dynamic physiology over a greater BP range is needed.

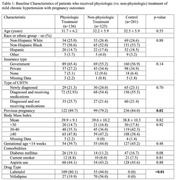

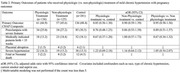

## 550 | Are Maternal Fever Height and Duration During Labor Associated with the Risk of Hypoxic‐Ischemic Encephalopathy?

Marie Cornet^1^; Michael W. Kuzniewicz^2^; Aditi Lahiri^2^; Yvonne Wu^3^

*^1^University of California San Francisco, San Francisco, CA; ^2^Kaiser Permanente Northern California, Kaiser Permanente Northern California, CA; ^3^University of California, San Francisco, San Francisco, CA*

4:00 PM ‐ 6:00 PM

**Objective**: To examine if the height of fever and/or duration between maternal fever onset and delivery modifies the risk of hypoxic‐ischemic encephalopathy (HIE).

**Study Design**: Population‐based cohort study of singleton neonates without congenital anomalies born > 35 weeks at 15 Kaiser Permanente Northern California hospitals between January 2012 and July 2019. Maternal fever was defined as at least one temperature >38°C before delivery. Maximal maternal temperature and timing of the first maternal fever were extracted from medical records. HIE was defined as the presence of both neonatal encephalopathy and perinatal acidosis (cord pH< 7 or base deficit >10 on any gas within 2 hours after birth). We evaluated the associations between maximal maternal temperature and duration between fever onset to delivery and neonatal outcomes, using Poisson regression adjusting for duration of labor as a spline.

**Results**: Among 280,347 neonates, exposure to maternal fever occurred in 27,317 (9.8%) and HIE occurred in 490 (0.17%). The incidence of HIE was higher among infants of febrile compared to afebrile mothers (Relative risk ‐ RR 4.4 [95% CI 3.6‐5.3]). Both the height (Figure 1) and duration (Figure 2) of fever were associated with an increased risk of HIE. After adjusting for duration of labor, the incidence of HIE increased with each 0.1°C increase in maximal maternal temperature (Incidence rate ratio‐ IRR 1.09; 95% CI 1.08‐1.11) and for each hour between fever onset and delivery (IRR 1.06 95%CI 1.05‐1.07). 

**Conclusion**: The higher the maternal fever and the longer the duration between fever onset and delivery, the higher the risk of developing HIE. Novel strategies designed to predict the risk of adverse neonatal outcomes during labor and delivery should account for these factors.

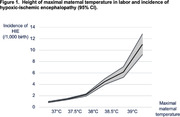

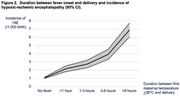

## 551 | Obstetric, Maternal and Neonatal Outcomes Associated with Attempted Trial of Labor after Cesarean Delivery

Marlee Hirsch^1^; Erez Lenchner^2^; Ashley Zimmermann^3^; Moti Gulersen^4^; Eran Bornstein^5^

*^1^Northwell Lenox Hill Hospital, Lenox Hill Hospital, NY; ^2^New York University Rory Meyers College of Nursing, New York, NY; ^3^Northwell Lenox Hill Hospital, New York, NY; ^4^Sidney Kimmel Medical College of Thomas Jefferson University, Philadelphia, PA; ^5^Northwell Lenox Hill Hospital, New Hyde Park, NY*

4:00 PM ‐ 6:00 PM

**Objective**: To examine the obstetric, maternal, and neonatal outcomes associated with attempted trial of labor after cesarean delivery (TOLAC) in the United States (US).

**Study Design**: A retrospective analysis of the Centers for Disease Control and Prevention's Natality live birth database (2016‐2022). The inclusion criteria consisted of term (37 0/7 ‐ 40 6/7 weeks), singleton pregnancies with a history of cesarean delivery. The study group comprised of individuals who attempted TOLAC, while the comparison group consisted of those undergoing repeat cesarean section without TOLAC. Obstetric, maternal, and neonatal outcomes were compared between the two groups using Pearson's chi‐square test. Results were presented as odds ratios (OR) with 95% confidence intervals (CI). Statistical significance was defined as p‐value < 0.05.

**Results**: Of the 7,250,494 live births with previous cesarean sections analyzed, 2,313,494 (31.9%) individuals attempted TOLAC. The prevalence of attempted TOLAC and the ORs based on different maternal and neonatal outcomes are presented in Tables 1 and 2, respectively.  

**Conclusion**: Based on this large contemporary US database, we provide an update on obstetric, maternal, and neonatal outcomes associated with attempted TOLAC. Attempted TOLAC was associated with a higher risk of maternal intensive care unit (ICU) admission, maternal antibiotic use, uterine rupture, immediate neonatal assisted ventilation, and 5‐minute APGAR scores < 7. However, attempted TOLAC was associated with lower odds of assisted neonatal ventilation after six hours and neonatal ICU admissions, suggesting that attempted TOLAC has a transient impact on the neonatal status. These findings can be used to help counsel patients on risks of attempting TOLAC.

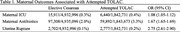

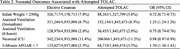

## 552 | Does Day of Delivery Matter? Obstetric, Maternal and Neonatal Outcomes in Term, Singleton Weekend Births

Marlee Hirsch^1^; Erez Lenchner^2^; Ashley Zimmermann^3^; Moti Gulersen^4^; Eran Bornstein^5^

*^1^Northwell Lenox Hill Hospital, Lenox Hill Hospital, NY; ^2^New York University Rory Meyers College of Nursing, New York, NY; ^3^Northwell Lenox Hill Hospital, New York, NY; ^4^Sidney Kimmel Medical College of Thomas Jefferson University, Philadelphia, PA; ^5^Northwell Lenox Hill Hospital, New Hyde Park, NY*

4:00 PM ‐ 6:00 PM

**Objective**: To determine whether obstetric, maternal, and neonatal outcomes differ between weekend and weekday deliveries in the contemporary United States (US).

**Study Design**: A retrospective analysis of the Centers for Disease Control and Prevention's Natality live birth database (2016‐2022). The inclusion criteria consisted of term (37 0/7 ‐ 40 6/7 weeks), singleton pregnancies. The study group comprised of births occurring on the weekend (Saturday or Sunday) while the comparison group consisted of those occurring during the weekdays (Monday through Friday). Obstetric, maternal, and neonatal outcomes were compared between the two groups using Pearson's chi‐square test. Results were presented as odds ratios (OR) with 95% confidence intervals (CI). Statistical significance was defined as p‐value < 0.05.

**Results**: Of the 22,355,756 live births analyzed, 4,598,349 (20.6%) births occurred on weekends. The prevalence of weekend births and the ORs based on different maternal and neonatal outcomes are presented in Tables 1 and 2, respectively.

**Conclusion**: Based on this large contemporary US database, weekend births are associated with higher rates of maternal intensive care unit (ICU) admission, 3rd and 4th degree perineal lacerations, maternal antibiotic use, uterine rupture, neonate less than 2500g, assisted ventilation, 5‐minute APGAR score < 7, and neonatal ICU admission. However, there was a lower risk of primary cesarean section on weekends than weekdays. This information can be used to guide providers into stratifying higher risk deliveries on weekdays when planning is possible.

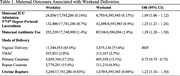

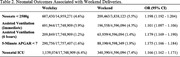

## 553 | Burdened by Prevention? Factors Associated with High Burden of Attending Antenatal Fetal Surveillance

Marta J. Perez^1^; Miriam Alvarez^1^; Sarah Lasater^1^; Lorie M. Harper^1^; Alison G. Cahill^2^

*^1^Dell Medical School, Austin, TX; ^2^Dell Medical School Health Transformation Research Institute, Dell Medical School, The University of Texas at Austin, Austin, TX*

4:00 PM ‐ 6:00 PM

**Objective**: Antenatal fetal surveillance (AFS) is recommended to screen for acidemia and prevent stillbirth in pregnancies with a maternal or fetal condition that increase these risks. AFS visits are in addition to prenatal care and factors associated with patient's experience of burdens of attending this care is lacking.

**Study Design**: We performed a cross‐sectional survey of patients attending AFS in a maternal fetal medicine ultrasound unit, querying patients regarding their burdens in cost, childcare, employment, and time‐related domains. Patients eligible for inclusion were those undergoing any type or frequency of AFS and were English or Spanish speaking. The primary aim was to describe factors associated with experiencing a high burden of AFS visits. Patients were categorized as “high burden” if they agreed with experiencing burden in any domain and “low burden” if they universally disagreed with experiencing burden. Chi square and t‐test as appropriate were used to determine associations between demographic and patient characteristics. 

**Results**: Of 186 patients, 82 (44.1%) experienced high burden and 104 (55.9%) were low burden. The high burden group was older (32.4 years vs 28.6 years, p< 0.01) and more likely to attend twice weekly testing (31.7% vs 15.4%, p< 0.01). There was no difference between high and low burden experience groups in gestational age at start of AFS, number of visits scheduled, having children at home, or work outside the home. The high burden group were more likely to have gestational diabetes as the indication for AFS (24.4% vs 9.6%, p< 0.01) though other indications for AFS were similar between high and low burden groups.

**Conclusion**: Almost half of patients experienced a high burden of recommended AFS, which was associated with a slightly older patient age, twice‐weekly frequency of AFS, and a diagnosis of gestational diabetes, which requires daily intensive blood glucose monitoring. Providers should attempt to query patients about burdens to develop a regimen of AFS that balances burden and stillbirth prevention.

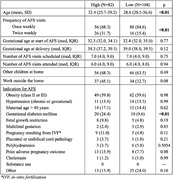

## 554 | Predictors of VBAC Success After Two Previous Cesarean Sections

Mary C. Gallo; Shaun R. Wesley; Siddharth Hariharan; Sarah Crimmins

*University of Rochester, Rochester, NY*

4:00 PM ‐ 6:00 PM

**Objective**: This study aimed to identify key predictors of a successful vaginal birth after cesarean (VBAC) among women with two previous cesarean sections and no prior spontaneous vaginal deliveries (SVD) using US vital statistics natality birth data, and to determine the uterine rupture rate in this population.

**Study Design**: A retrospective cohort study used data from the US Vital Statistics natality birth dataset. The analysis included women with two previous cesarean sections and no prior SVD. Descriptive statistics were performed on all variables. Logistic regression was used to identify predictors of VBAC success. Adjusted odds ratios (aOR) and 95% confidence intervals (CI) were calculated, and the uterine rupture rate was determined.

**Results**: The study included 537,852 women, with an overall VBAC success rate of 3.0%. Native Hawaiian or Other Pacific Islander (NHOPI) women had higher odds of VBAC success compared to White women (aOR = 1.38, CI: 1.09‐1.75). Women with Class II (aOR = 0.53, 95% CI: 0.46‐0.60) and Class III (aOR = 0.43, 95% CI: 0.38‐0.49) obesity had significantly lower odds of VBAC success. Women aged 40+ years exhibited lower odds of VBAC compared to women aged under 20 years (aOR = 0.41, 95% CI: 0.31‐0.55). Privately insured had higher odds of VBAC success than those with public insurance (aOR = 1.97, 95% CI: 1.84‐2.12). There were 536 cases of uterine rupture identified, corresponding to a rate of 0.10%.

**Conclusion**: This study highlights predictors associated with VBAC success. The overall VBAC success rate was low at 3.0%, a result potentially limited by using a retrospective dataset with many covariates. The finding that privately insured women had better outcomes than those with public insurance may reflect disparities in access to healthcare resources. The finding of increased risk of failure in the highest BMI and age categories continues to support these variables as independent risk factors for cesarean delivery. The reported uterine rupture rate of 0.10% is lower than previous studies, supporting VBAC as a viable option for delivery mode as part of shared decision making.

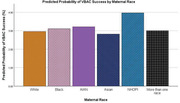

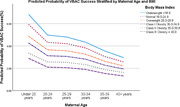

## 555 | Influence of French Labor‐management Guidelines on Incidence of Severe Postpartum Hemorrhage: A National Population‐Based Study

Mathilde Lepelletier^1^; Camille Le Ray^1^; Aude Girault^2^

*^1^INSERM UMR 1153, Obstetrical, Perinatal and Pediatric Epidemiology Research Team (Epopé), Center for Epidemiology and Statistics, FHU PREMA, Université de Paris, Paris, Ile‐de‐France; ^2^FHU PREMA, Paris, France. FHU PREMA, Paris, France; Université Paris Cité, Inserm, Centre for Research in Epidemiology and StatisticS (CRESS), Obstetrical Perinatal and Pediatric Epidemiology Research Team (EPOPé), Paris, France., Paris, Ile‐de‐France*

4:00 PM ‐ 6:00 PM

**Objective**: The 2021 French National Perinatal Survey (FNPS) revealed an increased rate of severe postpartum hemorrhage (PPH) since the previous survey in 2016. This rise might be attributed not only to the evolution of maternal risk factors but also to the publication of the 2017 guidelines that aimed at restricting labor interventions, such as oxytocin administration, for women in spontaneous labor. Our aim was to explore the association between changes in medical practice between 2016 and 2021 and incidence of severe PPH.

**Study Design**: Women who participated to the 2016 and 2021 FNPS and delivered of a term live‐born cephalic singleton after a spontaneous labor were included. We described the evolution of severe PPH rate, oxytocin use, and labor duration. We then assessed the determinants of severe PPH using univariate and multivariable analyses adjusting on maternal characteristics. Finally, we investigated the potential mediation effect of oxytocin use and prolonged labor on the relationship between the year of delivery and the occurrence of severe PPH.

**Results**: A total of 14,168 women were included, 7,529 in 2016 and 6,639 in 2021. The incidence of severe PPH increased from 1.2% in 2016 to 2.0% in 2021, p< 0.01. The use of oxytocin during labor decreased from 45.0% in 2016 to 30.2% in 2021, p< 0.01. Labor duration were significantly longer in 2021, regardless of parity (p < 0.01) After adjusting for maternal, obstetrical and maternity unit characteristics, giving birth in 2021 (versus 2016) remained associated with an increased risk of severe PPH (OR 1.7 [1.3‐2.2]). While oxytocin use did not appear as a mediating factor, prolonged labor mediated only 9% of the observed association between the year of delivery and the risk of severe PPH.

**Conclusion**: While the French Labor management Guidelines could partially explain the increase in severe PPH rates observed between 2016 and 2021, the decrease in the use of oxytocin does not appear to be associated with this increase.

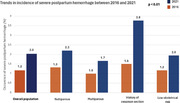

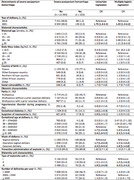

## 556 | Trajectory Analysis of Prenatal Urinary Tract Dilation and Risk of Postnatal Surgery

Matthew K. Janssen^1^; Markolline Forkpa^2^; Dana Weiss^3^; Nadav Schwartz^4^

*^1^University of Pennsylvania, Pittsburgh, PA; ^2^Pennsylvania Medicine Women's Health Clinical Research Center, Philadelphia, PA; ^3^Children's Hospital of Philadelphia, Philadelphia, PA; ^4^University of Pennsylvania, Perelman School of Medicine, Philadelphia, PA*

4:00 PM ‐ 6:00 PM

**Objective**: A prenatal urinary tract dilation (UTD) classification system is associated with postnatal urologic outcomes and assigns a score (UTDA1 or UTDA2‐3) depending on anterior‐posterior renal pelvis diameter (APRPD) and other markers of renal dysfunction. Postnatal management is dictated by a maximum peri‐delivery UTD score and there is limited data assessing the trajectory of UTD across gestation with postnatal outcomes. We aimed to identify trajectories of UTD and describe the association with postnatal surgery.

**Study Design**: We conducted a retrospective cohort study of infants with congenital UTD, via ICD‐10 codes, in visits with pediatric urology at an academic health system and born between 2015‐2020 with at least 2 years of follow up. We matched infants to maternal charts and delivery records in the health system. We obtained demographic, obstetric, prenatal ultrasounds, and infant surgical outcomes. We excluded patients without sonographic evidence of UTD prior to delivery as this would not have prompted postnatal follow up, or if there was only a single prenatal ultrasound. Postnatal management followed a standardized algorithm. We performed a trajectory analysis with UTD score and APRPD at multiple gestational ages and assessed their association with postnatal surgery.

**Results**: There were n = 243 matched dyads and n = 54 were excluded for missing prenatal ultrasound data, leaving n = 189 dyads for analysis. There were no demographic differences between infants with or without postnatal surgery. Three trajectories of UTD score (Figure 1) were identified: stable‐high, stable‐medium, and escalating groups with postnatal surgery in 27.7% (n = 23/83), 4.4% (n = 3/68), and 15.8% (n = 6/38), respectively, (p< 0.001). Two trajectories for APRPD (Figure 2) were identified with postnatal surgery in 46.2% (n = 18/39) vs 9.3% (n = 14/150, p< 0.001).

**Conclusion**: UTD score trajectories demonstrated an increased risk of postnatal surgery in stable‐high and escalating groups, while a stable‐medium risk UTD group conferred a low risk of surgery. ARPRD measures had two trajectories with differing risks of postnatal surgery.

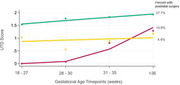

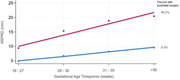

## 557 | Accuracy of ICD‐10 Codes for Placenta Accreta Spectrum: Frequency and Reasons for Miscoding

Matthew Givens^1^; Savvy Benipal^2^; Amanda A. Allshouse^2^; Ibrahim Hammad^3^; Robert M. Silver^2^; Jason D. Wright^4^; Koji Matsuo^5^; Bret D. Einerson^6^

*^1^Intermountain Health, Salt Lake City, UT; ^2^University of Utah, Salt Lake City, UT; ^3^Intermountain Health, Intermountain Health, UT; ^4^Columbia University Irving Medical Center, New York, NY; ^5^University of Southern California/Los Angeles General Medical Center, Los Angeles, CA; ^6^University of Utah Health, Salt Lake City, UT*

4:00 PM ‐ 6:00 PM

**Objective**: The ICD‐10 codes for placenta accreta spectrum (PAS) were introduced in 2015, facilitating improved research and quality tracking of this important source of maternal morbidity. However, the validity of these codes has not been thoroughly evaluated. We aimed to identify the frequency and reasons for PAS miscoding.

**Study Design**: We conducted a case series analysis of all deliveries associated with an ICD‐10 code for PAS in the two large healthcare systems (17 hospitals, including 2 PAS referral centers) in our state between September 2015 and December 2023. Medical records were reviewed by three reviewers to determine whether PAS was present using the FIGO clinical and/or histopathologic criteria. If PAS was excluded, the reason for misapplication of the code was assigned.

**Results**: There were 394 deliveries associated with ICD‐10 codes for PAS during the study period, with most (n = 156, 68%) occurring at a PAS referral center; 40% (95% CI 35‐45%) were false positive PAS. Many demographic and delivery factors were associated with a false PAS diagnosis including younger maternal age at delivery, a lower number of prior cesarean deliveries, absence of placenta previa, and delivery without hysterectomy, and later gestational age at delivery (Table). The most common cause (42.9%) for a false positive was hemorrhage due to another etiology not meeting clinical or histopathologic criteria for PAS (Figure). In 22.4% of false positives, there was antenatal suspicion of PAS, but PAS was not seen at delivery. In 22.4% of false positives, the code was erroneously applied.

**Conclusion**: In this study, 40% of cases with an ICD‐10 code for PAS did not meet clinical and/or histopathologic criteria for PAS. This underscores the need for improved clinician understanding of the clinical PAS criteria and improved coding practices for PAS. Additionally, studies using ICD‐10 to identify cases of PAS should consider the possibility of bias introduced by a significant false positive rate.

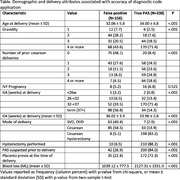

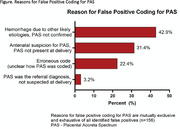

## 558 | Cardiovascular Disease and Hypertensive Disorders of Pregnancy Increase Severe Maternal Morbidity Risk

Maura Jones Pullins^1^; Johanna Quist‐Nelson^1^; Jerome J. Federspiel^2^; Kim Boggess^3^; Matthew Fuller^4^; Marie‐Louise Meng^2^

*^1^University of North Carolina, Chapel Hill, NC; ^2^Duke University School of Medicine, Durham, NC; ^3^University of North Carolina at Chapel HIll, Chapel Hill, NC; ^4^Duke University, Durham, NC*

4:00 PM ‐ 6:00 PM

**Objective**: Cardiovascular disease (CVD) and hypertensive disorder of pregnancy (HDP) both confer risk of severe maternal morbidity (SMM). Our objective was to examine rates of SMM by CVD and HDP type during delivery hospitalization and 90 days postpartum compared to births without CVD.

**Study Design**: Retrospective cohort study of pregnant patients > 18 years of age delivering between 2015 and 2020 in the Premier Database. Patients without CVD were compared to patients with six categories of CVD: congenital, ischemic, aortic, pulmonary hypertension (pHTN), cardiomyopathy, and valvular. Patients with multiple types of CVD were included in the analyses for each category. The exposure of interest was any SMM during delivery hospitalization and < 90 days postpartum. Patients were stratified by HDP type: gestational hypertension (GHTN), preeclampsia (PEC), and preeclampsia with severe features (sPEC). Adjusted predicted SMM rate (%) was calculated during delivery and within 90 days postpartum by CVD type stratified by HDP type, adjusted for age, payor status, race, ethnicity, hospital characteristics, and OB comorbidities.  

**Results**: Of 4,606,247 deliveries, 22,366 (0.5%) had CVD. The prevalence of HDP in this population was 11.9%. Patients with PEC had higher adjusted predicted SMM rates, independent of CVD. Those with CVD had significantly higher SMM rates across all HDP types. Patients with cardiomyopathy and pHTN who developed sPEC had the highest rates of SMM (26% and 33%) during delivery hospitalization (Figure 1). Acute respiratory distress syndrome (ARDS) and mechanical ventilation were the most common types of SMM during delivery hospitalization, while heart failure/pulmonary edema were the most common < 90 days postpartum (Figure 2).

**Conclusion**: Patients with CVD and PEC have a substantial increase in risk of SMM. The indications for the high rates of mechanical ventilation and etiologies of ARDS during delivery should be explored further. Heart failure is the most common postpartum morbidity and care should be targeted to improve surveillance and prevention postpartum.

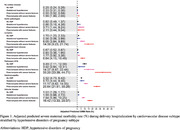

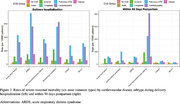

## 559 | Risk of Hemorrhage and Severe Maternal Morbidity (SMM) with Low‐lying Placenta: A Nationwide Analysis

May Abiad^1^; Giulia Bonanni^2^; Anna M. Modest^3^; Ali Javinani^4^; Nikan Zargarzadeh^4^; George R. Saade^5^; Scott A. Shainker^6^; Kjersti M. Aagaard^7^; Alireza A. Shamshirsaz^8^

*^1^Maternal Fetal Care Center, Boston Children's Hospital, Boston, MA; ^2^Postdoctoral Research Fellow Fetal Care and Surgery Center Division of Fetal Medicine and Surgery Boston Children's Hospital, Harvard Medical School, Boston, MA; ^3^Beth Israel Deaconess Medical Center, Boston, MA; ^4^Boston Children's Hospital, Harvard Medical School, Boston, MA; ^5^Macon & Joan Brock Virginia Health Sciences Eastern Virginia Medical School at Old Dominion University, Norfolk, VA; ^6^Maternal Fetal Care Center Associate Professor of Surgery Associate Professor of Obstetrics, Gynecology and Reproductive Biology Harvard Medical School, Boston, MA; ^7^Boston Children's Hospital, Division of Fetal Medicine and Surgery, Boston, MA; HCA Healthcare and HCA Healthcare Research Institute, Nashville, TN; HCA Texas Maternal Fetal Medicine, Houston, TX; Baylor College of Medicine and Texas Children's Hospital, Houston, TX, Boston, MA; ^8^Fetal Surgeon Chief, Division of Fetal Medicine and Surgery Director of the Maternal Fetal Care Center Director of the Perinatal Surgery Fellowship Professor of Surgery, Boston Children's Hospital Harvard Medical School, Boston, MA*

4:00 PM ‐ 6:00 PM

**Objective**: To evaluate and compare rates of hemorrhage and the CDC‐defined severe maternal morbidity (SMM) associated with persistent low‐lying placenta (LLP) and placenta previa (PP) using a national database.

**Study Design**: We conducted a retrospective analysis using the Nationwide Readmissions Database (NRD) from 2017 through 2019, reflecting the initial 3 years of coding for LLP. Patients with multifetal gestation, ectopic/molar pregnancies, and other placental abnormalities were excluded. Hemorrhage was defined as ante‐, intra‐, and post‐partum hemorrhage. Baseline characteristics and maternal outcomes were compared using chi‐square tests for categorical variables and Kruskal‐Wallis tests for continuous variables. Multivariable regression analysis was used to evaluate the risk ratios (RR) of adverse outcomes across these groups.

**Results**: Among 5,644,185 singleton deliveries, 11,755 were diagnosed with LLP & 23,817 with PP.  Compared to normal placentation, LLP was associated with a higher prevalence of hemorrhage (21.1% vs. 4.1%, p < 0.001), and increased RR of hemorrhage (RR: 5.19; 95% CI: 5.01‐5.38), SMM (RR: 2.55, 95% CI: 2.35‐2.78), blood transfusion (RR: 3.08; 95% CI: 2.79‐3.40), and hysterectomy (RR: 4.69; 95% CI: 2.95‐7.45). Additionally, delivery admission with LLP was found to have a longer inpatient stay (3 vs. 2 days, p < 0.001) and a higher cost ($21,451 vs. $17,025; p < 0.001). Patients with PP had significantly worse outcomes than those with normal placentation as well, with a 5.28‐fold RR of SMM, 7.11‐fold RR of blood transfusion, and 10.49‐fold RR of hemorrhage.

**Conclusion**: With the institution of distinct coding for LLP in 2017, we were able to distinguish between patients with LLP and PP. While patients with PP are known to have poor maternal outcomes, patients with LLP also have an increased risk of hemorrhage, SMM, and prolonged inpatient care relative to normal placentation. These findings should be considered during the prenatal counseling and management of patients with LLP.

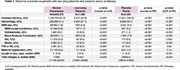

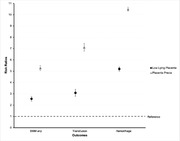

## 560 | Association between a Healthy Plant‐Based Diet and Adverse Pregnancy Outcomes

McKenzie K. Jancsura^1^; William A. Grobman^2^; Jiqiang Wu^2^; Michael Wirth^3^; David M. Haas^4^; Robert M. Silver^5^; George R. Saade^6^; Judith H. Chung^7^; Lynn M. Yee^8^; Kartik K. Venkatesh^2^

*^1^The Ohio State University College of Nursing, Columbus, OH; ^2^The Ohio State University, Columbus, OH; ^3^University of South Carolina, Columbia, SC; ^4^Indiana University School of Medicine, Indianapolis, IN; ^5^University of Utah, Salt Lake City, UT; ^6^Macon & Joan Brock Virginia Health Sciences Eastern Virginia Medical School at Old Dominion University, Norfolk, VA; ^7^University of California‐ Irvine, Irvine, CA; ^8^Northwestern University Feinberg School of Medicine, Chicago, IL*

4:00 PM ‐ 6:00 PM

**Objective**: We determined whether a healthy plant‐based diet in early pregnancy was associated with adverse pregnancy outcomes (APOs).

**Study Design**: This is a secondary analysis of data from the prospective Nulliparous Pregnancy Outcomes Study‐Monitoring Mothers‐to‐Be (nuMoM2b) cohort. We calculated Healthy Plant‐Based Diet Index (hPDI) scores from a validated food‐frequency questionnaire in the first trimester. Healthy plant‐based foods (e.g., whole grains, seed oils, vegetables) were assigned positive scores and unhealthy plant foods (e.g., refined grains, sugar sweetened beverages) and animal foods (e.g., meat, dairy) received negative scores. The hPDI was categorized in quartiles from quartile 1 (Q1, least plant‐based) to Q4 (most plant‐based). The outcomes were: small‐for‐gestational‐age [< 10^th^ percentile, SGA], large‐for‐gestational‐age [> 90^th^ percentile, LGA]), hypertensive disorder of pregnancy (HDP), gestational diabetes (GDM), and preterm birth (PTB) < 37 weeks. APOs were evaluated individually. Modified Poisson regression models adjusted for age, income, education, and health insurance. Secondarily, we assessed whether the above associations varied by adequate neighborhood food access, determined using the USDA *Food Atlas)*.

**Results**: Among 7,981 nulliparous individuals with dietary and outcome data, those who were in the highest quartile of a healthy plant‐based diet were at a lower risk of developing HDP (11.0% vs 15.1%; aRR 0.76; 95% CI 0.62 to 0.94) and GDM (3.4% vs 4.1%; aRR 0.64; 95% CI 0.44 to 0.92) compared with those in the lowest quartile. The above associations did not vary by neighborhood food access (interaction p‐value = 0.31 for HDP and 0.52 for GDM in adjusted models). A healthy plant‐based diet was not associated with SGA, LGA, or PTB (TABLE).

**Conclusion**: A healthy plant‐based diet in early pregnancy was associated with a lower risk of developing HDP and GDM in nulliparous individuals, after accounting for potential socioeconomic confounders. Findings suggest the potential benefits of a plant‐based diet in early pregnancy and warrant further study.

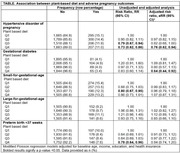

## 561 | Effect of Oxytocin Rest on Labor Outcomes and Uterine Contractility During Induction of Labor

McKenzie Barber^1^; Nandini Raghuraman^2^; Katherine H. Bligard^2^; Jeannie C. Kelly^2^; Antonina I. Frolova^3^

*^1^Washington University in Saint Louis, St. Louis, MO; ^2^Washington University School of Medicine in St. Louis, St. Louis, MO; ^3^Washington University School of Medicine, St. Louis, MO*

4:00 PM ‐ 6:00 PM

**Objective**: For patients with inadequate contractions despite high‐dose continuous oxytocin (OT), a period of OT rest is often trialed to resensitize uterine smooth muscle to OT; however, the utility of this is unknown. Our aim was to determine the effect of OT rest on labor outcomes and uterine contractility compared to continuous OT during induction of labor (IOL).

**Study Design**: A secondary analysis of a prospective cohort study of singleton, term pregnancies admitted in labor or for IOL from January 2020 to July 2022 was performed. Inclusion criteria were nulliparity, IOL, and OT use. All patients had an intrauterine pressure catheter placed within 30 minutes of membrane rupture, regardless of labor progress. OT rest was compared to continuous OT. Primary outcomes were completion of first stage, length of active phase, and length of second stage. We used multivariable logistic regression to adjust for potential confounders. Secondary outcomes were Cesarean delivery and Montevideo units (MVUs) at three time points: active labor (cervix ≥ 6cm), max OT dose, and first stage completion. Subgroup analysis was performed to exclude OT rest for non‐reassuring fetal status (NRFS).

**Results**: Of the 120 patients included, 55 (45.8%) had OT rest and 65 (54.1%) had continuous OT. Indications for OT rest were NRFS (73.7%), protracted labor (12.3%), and other (14.0%). Those with OT rest had significantly lower risk of completing the first stage (aRR 0.81, 95% CI 0.52‐0.97). This association persisted when OT rest for NRFS was excluded (aRR 0.52, 95% CI 0.15‐0.90). There were no differences in max OT dose (19.1 vs 16.7, p = 0.24), length of active phase, or length of second stage (Table 1). Those with OT rest were more likely to deliver via Cesarean (Table 2). MVUs did not differ between groups at any time (Table 2).

**Conclusion**: Nulliparous patients who had OT rest for protracted labor were 48% less likely to complete the first stage of labor compared to those who had continuous OT; a higher proportion also delivered via Cesarean. Further research is needed to determine the utility of OT rest after diagnosis of protracted labor.

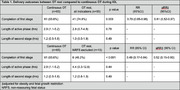

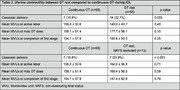

## 562 | Baby Got #Vbac: A Content Analysis of Trending Videos on Tiktok

Megan N. Happ^1^; Allison Chu^2^; Anissa Cervantes^3^; Jenny Wu^4^; Jonas J. Swartz^4^

*^1^Duke University, Durham, NC; ^2^Duke University School of Medicine, Durham, NC; ^3^Howard University, Washington DC, DC; ^4^Duke University Medical Center, Durham, NC*

4:00 PM ‐ 6:00 PM

**Objective**: Obstetric providers may be able to combat disinformation and strengthen patient‐provider trust by understanding health information many of their patients consume on social media. We aimed to characterize and evaluate vaginal birth after Cesarean (VBAC) video content presented on TikTok.

**Study Design**: We used the web‐scraping tool Apify to compile the 100 top “#vbac” videos.  Unrelated, unavailable, or non‐English videos were excluded. Two independent reviewers collected twenty‐six descriptive data points for each video; a third arbitrated differences. Medical facts were appraised using VBAC guidelines from American College of Obstetricians and Gynecologists and Society of Maternal Fetal Medicine. Two validated measures were used: a modified 5‐point DISCERN scale assessed information quality and Patient Education Materials Assessment Tool (PEMAT) assessed understandability and actionability.

**Results**: The top 100 videos had approximately 156 million views, 12 million likes, and 317,000 shares. A majority (81.0%) were made by non‐healthcare professionals. The most common primary video theme was a personal experience or opinion (36.0%); of these, 13 (36.0%) mentioned distrust of or dismissal by providers. Twenty percent of all videos addressed home birth after Cesarean (HBAC), including 16 promoting HBAC by a single creator. Twenty‐four videos presented medical facts; of these, videos by healthcare providers (n = 11) and non‐healthcare providers (n = 13) included evidence‐based facts 64.0% (n = 7) and 46.0%(n = 5) of the time, respectively. Overall videos exhibited high understandability (PEMAT median 85.5%, SD 15.7%), but low information quality (DISCERN mean 1.5, SD 0.9).

**Conclusion**: Videos with #vbac had low information quality, a finding consistent with previous work evaluating videos on contraception and abortion. Our study shows that top #vbac videos often lack health information. Of videos that include medical facts, many are incomplete or inaccurate. Obstetric providers can use the findings of this study to engage with patients they know are using TikTok for health education.

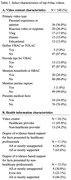

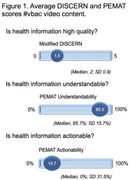

## 563 | Second Stage of Labor Duration Among Patients with Obesity: How Likely is Vaginal Delivery?

Xiang Yu Feng^1^; Christina Frasik^1^; Megan C. Oakes^2^

*^1^University of California, Irvine Division of Maternal‐Fetal Medicine, University of California, Irvine, CA; ^2^MemorialCare Miller Children's and Women's Hospital Long Beach, Miller Children's and Women's Hospital, Long Beach, CA*

4:00 PM ‐ 6:00 PM

**Objective**: ACOG recently revised guidelines defining an abnormally prolonged second stage labor as ≥ 3 hours. We sought to evaluate the difference in vaginal delivery rate for nulliparous patients with obesity and a singleton, term, vertex fetus (NTSV) with a normal (< 3 hour) versus prolonged (≥ 3 hour) second stage of labor. 

**Study Design**: A retrospective cohort study of NTSV patients with obesity who achieved the second stage of labor, delivered at a tertiary care center hospital from 6/1/2023‐12/31/2023. Obesity was defined as BMI ≥ 30 kg/m^2^ on admission. The primary outcome was vaginal delivery. Secondary outcomes included select maternal and neonatal morbidities. Categorical variables were compared using Chi square and Fisher's exact. Normally distributed continuous variables were assessed using Student's T‐test. Multivariable logistic regression was used to adjust for confounders.

**Results**: Of 348 included patients, 274 (78.7%) had a second stage < 3 hours and 74 (21.3%) had a second stage ≥ 3 hours. Those with a prolonged second stage were older (p = 0.04), more likely to be admitted for induction of labor (p< 0.01) and utilize oxytocin in the second stage (p = 0.02). After adjusting for confounders, a prolonged second stage was associated with a 22% lower likelihood of vaginal delivery (aRR 0.78, CI 0.41‐0.95). Patients with a prolonged second stage were more than 4 times as likely to have an operative vaginal delivery (aRR 4.54, 95% CI 2.97‐6.29). 3^rd^ degree lacerations were more likely in a prolonged second stage (aRR 10.25, 95% CI 2.02‐41.55); however, the overall frequency was low. There were no other differences in maternal or neonatal morbidities between the groups. 

**Conclusion**: While NTSV patients with obesity and a prolonged second stage of labor had a lower likelihood for vaginal delivery compared to those with a normal second stage duration, both groups had high rates of success. In patients with a prolonged second stage, 40% had an operative vaginal delivery, highlighting the importance of this obstetric skill in preventing the first cesarean section in patients with a protracted labor course.  

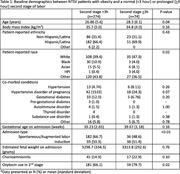

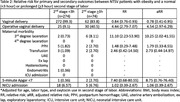

## 564 | Prolonged Second Stage: What is the Likelihood of a Safe, Vaginal Delivery?

Christina Frasik^1^; Xiang Y. Feng^1^; Megan C. Oakes^2^

*^1^University of California, Irvine Division of Maternal‐Fetal Medicine, University of California, Irvine, CA; ^2^MemorialCare Miller Children's and Women's Hospital Long Beach, Miller Children's and Women's Hospital, Long Beach, CA*

4:00 PM ‐ 6:00 PM

**Objective**: ACOG has recently revised a clinical practice guideline defining a prolonged second stage labor as >3 hours, citing increased risk for adverse maternal and neonatal outcomes and lower likelihood of vaginal delivery at longer second stage durations. We aimed to measure how second stage duration is associated with vaginal delivery rate and adverse maternal and neonatal outcomes.

**Study Design**: This was a retrospective cohort study of nulliparous, term patients with a singleton, vertex fetus (NTSV) delivered at a tertiary care center hospital from 6/1/2023‐12/31/2023. Patients who did not achieve the second stage of labor were excluded. Patients were stratified into three groups based on second stage duration: < 3 hours, >3 hours but < 4 hours, or > 4 hours. The association between second stage duration and all outcomes was tested using the Cochran‐Armitage test of trend.

**Results**: 700 patients were included. There was a significant decrease in vaginal delivery rate with increasing duration of the second stage (P‐trend < 0.01). In contrast, there was a significant increase in operative vaginal delivery rate with increasing second stage duration (P‐trend < 0.01). The rate of neonatal 5‐minute Apgar < 7 increased with increasing second stage duration (P‐trend 0.04); however, no other significant trends were noted for other markers of maternal morbidity or neonatal ICU admission.

**Conclusion**: Patients who reached the second stage of labor were significantly less likely to achieve a vaginal delivery as the second stage duration increased. However, the overall vaginal delivery rate remained high, with a significant proportion of operative vaginal deliveries among those with a prolonged second stage.

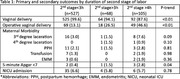

## 565 | Longitudinal Trends in Symptom and Mood During Pregnancy: a Retrospective Study from Digital Health App

Mia Charifson^1^; Timothy Wen^2^; Bonnie Zell^1^; Isabel Fulcher^1^

*^1^Delfina Care Inc, Delfina Care Inc, CA; ^2^University of California, San Diego, Irvine, CA*

4:00 PM ‐ 6:00 PM

**Objective**: Pregnancy symptomatology and mood are routinely captured during prenatal care, however, little data exists on how their trajectories vary throughout pregnancy. The use of mobile pregnancy trackers presents new opportunities to study symptom and mood trajectories throughout pregnancy.

**Study Design**: Pregnant patients from three clinics enrolled in a pregnancy mobile application between January 2023 and July 2024 and were instructed by their care team to track weekly pregnancy symptoms (100 unique symptoms available to select) and daily mood rating (1 to 5) in the application. We plot the trajectories of the top five symptom groups and average mood throughout pregnancy using cubic splines. We perform statistical tests for linear and quadratic terms in regression models to describe trends by gestational week.

**Results**: By July 2024, there were n = 1445 patients using the application. 1073 users reported a total of 24,273 symptoms (average 22.62 symptoms per user) and 1100 reported a total of 29,148 mood measures (average 26.5 moods per user). The top five symptom groups ever reported in our cohort were constitutional, musculoskeletal, gastrointestinal, obstetric, and genitourinary symptoms (Table 1). Each symptom group had a unique trajectory throughout pregnancy (Figure 1). Musculoskeletal symptoms increased linearly (p< 0.01) and gastrointestinal symptoms decreased linearly (p< 0.01). Constitutional (p< 0.01), obstetric (p< 0.01), and genitourinary (p< 0.01) all had U‐shaped curves throughout pregnancy. Average mood also exhibited a slight inverted U‐shaped curve (p< 0.01), although variation throughout pregnancy was low overall (SD = 0.15).

**Conclusion**: We observed distinct trajectories of symptoms and mood throughout pregnancy. Such information could improve personalized pregnancy care, use evidence‐based knowledge to normalize pregnancy experiences, and may be potentially informative for early detection of adverse outcomes.

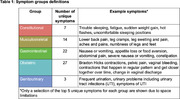

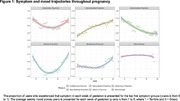

## 566 | Oral Presentation Conversion to Manuscript Publication Among Obstetrics and Gynecology Subspecialty Meetings

Minhazur R. Sarker^1^; Maha Pasha^2^; Dana R. Canfield^3^; Blair Thompson^4^; Dylan Hutson^4^; Megan King^4^; Harriet Rothschild^4^; Rachel L. Wiley^1^; Gladys (Sandy) A. Ramos^1^; Cynthia Gyamfi‐Bannerman^1^

*^1^University of California, San Diego, San Diego, CA; ^2^N/A, San Diego, CA; ^3^UC San Diego Health, San Diego, CA; ^4^University of California San Diego, San Diego, CA*

4:00 PM ‐ 6:00 PM

**Objective**: We aimed to evaluate the oral presentation to full manuscript conversion rate of Obstetrics and Gynecology subspecialty meetings.

**Study Design**: We evaluated the 2019 meeting supplements from four subspecialty societies: 1) Society for Maternal‐Fetal Medicine (SMFM), 2) Society of Gynecologic Oncology (SGO), 3) American Urogynecologic Society (AUGS), and 4) American Society for Reproductive Medicine (ASRM). Abstracts awarded an oral presentation were included and queried on PubMed using a multimodal method incorporating abstract keywords and first and last author's names to determine publication status. An abstract was classified as a published manuscript if the first author of the meeting abstract was credited in the final publication. The primary outcome was publication status. Secondary outcomes included first author consistency, manuscript journal impact factor, and time to publication. All analyses were performed on STATA IC 15.1 with Chi‐square, ANOVA, and Kruskal‐Wallis to determine statistical significance at a p‐value < 0.05.

**Results**: There were differences in the proportion of abstracts presented as oral presentations with SMFM 109/1059 (10.3%), SGO 42/629 (6.7%), AUGS 153/704 (21.4%), and ASRM 276/1099 (25.1) (p‐value < 0.01, Table 1). SMFM and SGO had a greater proportion of randomized controlled trials and SMFM had the greatest proportion of basic science studies. Among oral presentations, the publication rate was greatest for SGO (78.6%) and SMFM (74.3%), lower in AUGS (66.7%), and lowest in ASRM (51.1%) (p‐value < 0.01, Figure 1). Among published manuscripts, SMFM had the highest median impact factor and quickest median time to publication (p‐value < 0.01, Table 1). There were no differences noted in first author consistency (Table 1).

**Conclusion**: We found that the societies with the lowest proportion of oral presentations had the highest publication rate. More importantly, 21‐49% of all oral presentations at subspecialty meetings are unpublished suggesting the need to improve the mechanism for or access by which highly regarded research translates to publication.

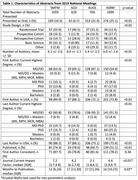

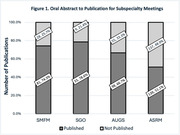

## 567 | Practice Patterns in the Administration of Antenatal Corticosteroids in Patients at Risk of Preterm Birth

Moti Gulersen^1^; Frank I. Jackson^2^; Ashley N. Battarbee^3^; Vincenzo Berghella^1^; Matthew J. Blitz^2^

*^1^Sidney Kimmel Medical College of Thomas Jefferson University, Philadelphia, PA; ^2^Northwell, New Hyde Park, NY; ^3^Center for Women's Reproductive Health, University of Alabama at Birmingham, Birmingham, AL*

4:00 PM ‐ 6:00 PM

**Objective**: Although suboptimal timing of antenatal corticosteroid (ACS) administration in patients at risk of preterm birth (PTB) is well known, studies stratifying timing by GA at administration are limited. We aimed to evaluate whether ACS timing differs by gestational age (GA) of administration in patients at risk of PTB.

**Study Design**: Multicenter retrospective cohort of all singleton pregnancies exposed to ACS between 22 0/7‐36 6/7 weeks of gestation and delivered from 2019‐2023. Patients who had neonates that were not actively resuscitated at birth and intrauterine fetal demise were excluded. The primary outcome of time interval from ACS administration to delivery, stratified by < 2, 2‐7, and >7 days, was compared among 4 gestational age groups: 22 0/7‐25 6/7, 26 0/7‐29 6/7, 30 0/7‐33 6/7, and 34 0/7‐36 6/7 weeks of gestation. The secondary outcome was delivery at term (37 0/7 weeks of gestation or later). Statistical analysis included use of Chi‐squared test. Statistical significance was set *P* < 0.05.

**Results**: Of the 116,803 patients who delivered during the study period, 5,003 (4.3%) received ACS prior to delivery. Primary and secondary outcomes are displayed in the Table.

**Conclusion**: Data from this large cohort suggest that optimal timing of ACS in patients at risk of PTB is low, regardless of gestational age of ACS administration. The likelihood of optimal timing is significantly decreased in the late preterm period compared to early preterm periods. A significant proportion of patients exposed to ACS in the periviable period deliver at term. These data highlight the importance of developing PTB prediction tools to aid practitioners in optimizing the timing of ACS.

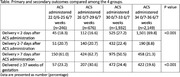

## 568 | Should Antenatal Corticosteroids Be Considered in Singletons Undergoing Cerclage at 22 0/7‐23 6/7 Weeks’ Gestation?

Moti Gulersen^1^; Frank I. Jackson^2^; Rupsa C. Boelig^3^; Eran Bornstein^4^; Vincenzo Berghella^1^; Matthew J. Blitz^2^

*^1^Sidney Kimmel Medical College of Thomas Jefferson University, Philadelphia, PA; ^2^Northwell, New Hyde Park, NY; ^3^Sidney Kimmel Medical College, Thomas Jefferson University, Philadelphia, PA; ^4^Northwell Lenox Hill Hospital, New Hyde Park, NY*

4:00 PM ‐ 6:00 PM

**Objective**: The American College of Obstetricians and Gynecologists (ACOG) suggests that antenatal corticosteroids (ACS) can be considered as early as 22 0/7 weeks’ gestation in patients at increased risk of preterm (PTB) within 7 days of presentation. We aimed to evaluate the likelihood of delivery within 7 days of cerclage placement to determine whether ACS should be considered in this high‐risk population.

**Study Design**: Multicenter retrospective cohort of all singletons undergoing an ultrasound‐indicated cerclage (UIC) or physical exam‐indicated cerclage (PEIC) between 22 0/7‐23 6/7 weeks’ gestation and delivered from 01/2018‐06/2023. The primary outcome was delivery < 7 days after cerclage placement. Secondary outcomes included delivery < 14 days after cerclage placement and PTB. We planned to compare baseline characteristics at the time of cerclage placement between those who delivered < 7 days versus 7 days or later after cerclage. Statistical analysis included use of Pearson's chi‐square for categorical variables and Wilcoxon rank sum test for continuous variables. Statistical significance was set *P* < 0.05.

**Results**: Of the 83 patients included, 61 (73.5%) underwent an UIC and 22 (26.5%) underwent a PEIC. There were no patients who delivered < 7 days after cerclage placement; thus, we primarily compared those who delivered < 14 days versus 14 days or later. Three (3.6%) of patients delivered < 14 days after cerclage placement (12, 13, and 13 days). The prevalence of PTB < 37 weeks was 70.0%. Baseline characteristics compared between the two groups are displayed in the Table. Patients who delivered < 14 days after cerclage more commonly had a BMI > 40 kg/m^2^  compared to those who delivered later (Table).

**Conclusion**: Although patients undergoing cerclage at 22 0/7‐23 6/7 weeks’ gestation are at high risk of PTB, none of the patients in this cohort delivered < 7 day after placement. These data suggest that ACS administration may be deferred in this patient population unless additional indications are present.

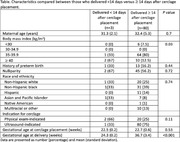

## 569 | The Impact of Peripartum Fever on Long‐Term Neurological Morbidity in Offspring

Narkis Hermon^1^; Omri Zamstein^2^; Tamar Wainstock^3^; Eyal Sheiner^3^

*^1^Soroka Medical Hospital, Soroka Medical Center, HaDarom; ^2^Soroka University Medical Center, Soroka University Medical Center, HaDarom; ^3^Department of Obstetrics and Gynecology, Soroka University Medical Center, Ben‐Gurion University of the Negev, Beer‐Sheva, Israel., Beer Sheva, HaDarom*

4:00 PM ‐ 6:00 PM

**Objective**: Peripartum fever, resulting from various infectious and non‐infectious causes, can lead to adverse maternal and neonatal outcomes. This study explores the potential long‐term neurological effects of peripartum fever on the offspring.

**Study Design**: A population‐based cohort analysis was conducted at a tertiary referral hospital to assess the incidence of neurological conditions in the offspring—encompassing both hospital and community‐based diagnoses—based on the presence of peripartum fever. Kaplan‐Meier survival curves evaluated the cumulative incidence of morbidity, while Cox proportional hazards models adjusted for confounding factors.

**Results**: In this study involving 232,476 deliveries, 1,728 (0.7%) were associated with peripartum fever. Compared to the group without fever, those with peripartum fever exhibited significantly higher incidences of hypertensive disorders (9.5% vs. 4.6%), preterm births (15.7% vs. 7.0%), non‐reassuring fetal heart rate patterns (17.7% vs. 5.4%), and cesarean deliveries (47.0% vs. 13.8%; Table). Although overall neurological morbidity in the offspring was somewhat higher in the fever group (19.6% vs. 17.4%, p = 0.018), there was no notable difference in the occurrence of autism spectrum disorders (0.3% vs. 0.5%, p = 0.62). Additionally, the Kaplan‐Meier analysis revealed no significant difference in the overall neurological morbidity (Log‐rank p‐value = 0.899; Figure). Following adjustments for maternal age and gestational age at birth, there was no significant association between peripartum fever and long‐term neurological outcomes in the offspring (adjusted HR = 0.95, 95% CI 0.85–1.06, p = 0.35; Table).

**Conclusion**: Peripartum fever is linked to increased rates of obstetric complications; however, it does not significantly impact long‐term neurological outcomes in offspring.

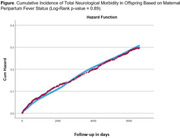

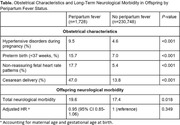

## 570 | Maternal and Neonatal Outcomes: Ampicillin Plus Gentamicin vs. Ampicillin Alone for Intrapartum Fever

Natav Hendin; Yarin Mash; Or Bercovich; Yossi Geron; Mor Ginat Shaked; Eran Hadar; Ohad Houri

*Rabin Medical Center, Petach Tikva, HaMerkaz*

4:00 PM ‐ 6:00 PM

**Objective**: To evaluate the effectiveness of ampicillin plus gentamycin versus ampicillin alone in treating women with intrapartum fever.

**Study Design**: 10‐year retrospective study at a single tertiary hospital, including term singleton vaginal deliveries who developed isolated intrapartum fever (≥38°C) without any concurrent evidence of infection. The department's treatment protocol changed in 2016 from ampicillin alone to ampicillin plus gentamicin. Primary outcome was puerperal endometritis. Secondary outcomes included microbiological studies, such as maternal urine or blood cultures, and neonatal outcomes.

**Results**: A total of 389 women met inclusion criteria and were treated with either ampicillin alone (n = 128, 33%) or ampicillin plus gentamycin (n = 261, 67%). The incidence of endometritis was higher in the ampicillin group compared to the ampicillin plus gentamycin group, although not statistically significant (6.25% vs. 2.30%, P = 0.08). Blood and urine culture positivity rates were similar (6.25% vs. 4.60%, p = 0.47, 2.34% vs. 5.36%, p = 0.20 respectively). Maternal hospitalization was longer for the ampicillin group (3.36 ± 1.1 days vs. 3.03 ± 1.04 days, P< 0.01).

Neonatal intensive care unit (NICU) admission rates were significantly higher in the ampicillin group (36.7% vs. 11.9%, P< 0.01). Conversely, among the neonates admitted to the NICU, the incidence of respiratory pathologies, such as respiratory distress syndrome or transient tachypnea of the newborn, and the need for ventilation were significantly higher in the ampicillin plus gentamycin group (2.1% vs. 25.8%, P< 0.01, 4.3% vs. 38.7 %, p< 0.01 respectively) (Table 1).

**Conclusion**: Women treated with ampicillin alone for intrapartum fever had higher rates of adverse maternal outcomes compared to those treated with ampicillin plus gentamycin. Although NICU admission rates were higher in the ampicillin group, respiratory diseases were more prevalent in the ampicillin plus gentamycin group.

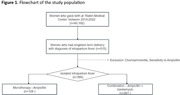

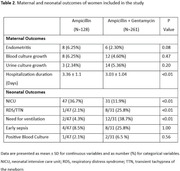

## 571 | Neonatal Outcomes After Expedited Delivery Versus Expectant Management for Preeclampsia with Severe Features

Frank I. Jackson; Nathan A. Keller; Sarah H. Abelman; Luis A. Bracero; Matthew J. Blitz

*Northwell, New Hyde Park, NY*

4:00 PM ‐ 6:00 PM

**Objective**: To determine if expectant management versus expedited delivery for preeclampsia with severe features (sPEC) improves neonatal outcomes.

**Study Design**: Retrospective cohort study of pregnancies complicated by sPEC within a large health care system in New York between 2019 and 2023. Patients who delivered < 24 hours after diagnosis (immediate delivery), without receiving two doses of betamethasone were excluded. Deliveries were classified as expectant management (EM) if they occurred > 72 hours after diagnosis and as expedited delivery (ED) if they occurred within 24‐72 hours after diagnosis. The primary outcome was severe neonatal morbidity (SNM), a composite neonatal adverse outcome indicator which includes diagnoses and procedures. Secondary outcomes included respiratory distress syndrome (RDS), bronchopulmonary dysplasia (BPD), interventricular hemorrhage (IVH), and sepsis. Analysis was performed separately for deliveries at 28‐34 weeks and < 28 weeks due to presumed variation in clinical management strategies. A logistic regression was performed. All statistical analyses were performed in R 4.3.1.

**Results**: In total, 218 patients were included for analysis: 178 (81.7) delivered between 28 and 34 weeks and 40 (18.3) delivered at < 28 weeks. Overall, the time interval from diagnosis to delivery had a median of 4.0 days [IQR: 2.4‐8.0]. There were no significant differences in SNM, RDS, BPD, IVH, or sepsis between the ED and EM groups in either gestational age grouping (Table 1).

**Conclusion**: Pregnancies complicated by sPEC did not show improvement in SNM, RDS, BPD, IVH, or sepsis whether the delivery was expedited or occurred after expectant management. Significant maternal risks without clear neonatal benefit of expectant management may favor expedited delivery.

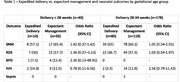

## 572 | Racial and Ethnic Disparities in Severe Maternal Morbidity and Icu Admission by Bariatric Surgery Status

Nathan A. Keller; Frank I. Jackson; Sarah H. Abelman; Luke Pierce; Sarika Arora; Alexis Palmer; Luis A. Bracero; Matthew J. Blitz

*Northwell, New Hyde Park, NY*

4:00 PM ‐ 6:00 PM

**Objective**: To assess racial and ethnic disparities in severe maternal morbidity (SMM) and ICU admission in patients with and without a history of bariatric surgery.

**Study Design**: Retrospective cohort study of pregnancies within a large health care system in New York from 2019‐2023. The primary exposure was bariatric surgery, which included: gastric sleeve, gastric band, Roux‐en‐Y gastric bypass, and biliopancreatic diversion with duodenal switch. Race and ethnicity were self‐identified from pre‐specified categories. The primary outcome was SMM during delivery hospitalization. SMM was defined using the Centers for Disease Control and Prevention (CDC) criteria which includes 21 indicators. A multivariable logistic regression was used to estimate the strength of association between prior bariatric surgery and SMM and ICU admission, adjusting for Obstetric Comorbidity Index (OBCMI) and race and ethnicity group. All statistical analyses were performed in R 4.3.1.

**Results**: In total, 127,959 pregnancies with delivery outcomes were identified with 493 (0.4%) with and 127,466 (99.6%) without a history of bariatric surgery. SMM occurred in 43 (8.7%) of pregnancies with a history of bariatric surgery and in 5,333 (4.2%) of pregnancies without such a history [unadjusted OR: 2.19, 95% CI 1.58‐2.96]. After adjustment for OBCMI and race and ethnicity groups, no significant differences among SMM in patients with a history of bariatric surgery was observed [aOR: 1.37, 95% CI 0.98‐1.88]. ICU admission occurred in 12 (2.4%) pregnancies with a history of bariatric surgery and in 797 (0.6%) pregnancies without such a history [aOR: 2.13, 95% CI 1.11‐3.71]. All other race and ethnicity groups were at increased risk for SMM compared to Non‐Hispanic White patients (Table 1).

**Conclusion**: A history of bariatric surgery was associated with increased risks of SMM before, but not after, adjustments for OBCMI and race and ethnicity group. ICU admission was significantly increased even after adjustments. Significant differences in SMM were seen by race and ethnicity group.

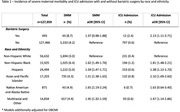

## 573 | Ultrasonographic Fetal Lung Echotexture Analysis in the Prediction of Neonatal Respiratory Distress Syndrome

Nathan A. Keller^1^; Luis A. Bracero^1^; Frank I. Jackson^1^; Insaf Kouba^2^; Christina Karras^1^; Sarah H. Abelman^1^; Wassil Kouba^1^; Matthew J. Blitz^1^; Sleiman R. Ghorayeb^1^

*^1^Northwell, New Hyde Park, NY; ^2^Northwell, St. Petersburg, FL*

4:00 PM ‐ 6:00 PM

**Objective**: To determine if ultrasonographic fetal lung echotexture analysis is predictive of neonatal respiratory distress syndrome (RDS).

**Study Design**: Prospective cohort study of singleton gestations with medically‐indicated ultrasound examinations from October 2017 through September 2021. Grayscale transverse fetal lung images were obtained at the level of the four‐chamber heart view with GE Voluson E8 and E10 ultrasound machines with convex array transducers. A region of interest was selected in each fetal lung image. Fetal lung heterogeneity index (HI) was determined with MATLAB software using a dithering technique with ultrasound image pixels transformed into a binary map from which a dynamic range value was determined. The fetal lung HI ultrasound occurred < 7 days prior to delivery. Pregnancies were categorized by delivery timing: late preterm (34+0 to 36+6 WGA), early term (37+0 to 38+6 WGA), and full‐term (39+0 to 40+6 WGA). Pregnancies with delivery occurring > 7 days from HI measurement were excluded from the analysis.

**Results**: In total, 468 singletons were delivered < 7 days from the HI ultrasound examination. Of these, 94 (20.1%), 276 (59.0%), and 98 (20.9%) delivered preterm, early term, and full term, respectively. RDS occurred in 20 (21.3%) of late preterm, 3 (1.0%) of early term, and 1 (1.0%) of full term deliveries. Figure 1 shows the receiver operating characteristic (ROC) curve for fetal lung HI as a predictor of RDS in the preterm delivery cohort. HI was modestly predictive of RDS in the late preterm cohort (AUC: 0.632), but not in the early or full term cohort, potentially from low incidences of RDS in these time frames.

**Conclusion**: Fetal lung HI had a modest association with RDS in the late preterm time period which is the time of greatest fetal lung HI change and greatest risk of RDS. While fetal lung HI alone may not be a great predictor of RDS, it may be a useful adjunct when combined with other pregnancy risk factors.

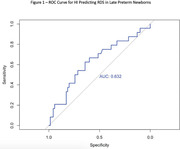

## 574 | Risk Factors for Maternal Abruption after the Fetoscopic Laser Surgery for Twin Twin Transfusion Syndrome

Neha Agarwal^1^; Ramesha Papanna^2^; Dejian Lai^3^; Anthony Johnson^4^; Sami Backley^5^; Eric P. Bergh^6^; Edgar A. Hernandez‐Andrade^2^; Gustavo Vilchez^7^; Felicia V. LeMoine^5^; Alexandra Garcia^5^; Tania clarete^5^; Elisa Garcia^5^; Ashley Salazar^8^; Jimmy Espinoza^5^

*^1^Division of Fetal Intervention, Department of Obstetrics, Gynecology and Reproductive Sciences, UTHealth McGovern Medical School, Houston, TX, USA, Houston, TX; ^2^McGovern Medical School at UTHealth Houston, Houston, TX; ^3^UT School of Public Health, Houston, TX; ^4^McGovern Medical School University of Texas Health Science Center at Houston (UThealth), Houston, TX; ^5^Division of Fetal Intervention, Department of Obstetrics, Gynecology and Reproductive Sciences, McGovern Medical School at UTHealth Houston, Houston, TX; ^6^UT Health, Houston, TX; ^7^UTHealth Houston, Houston, TX; ^8^Division of Fetal Intervention, Department of Obstetrics, Gynecology and Reproductive Sciences, McGovern Medical School at UT Health Houston, Houston, TX*

4:00 PM ‐ 6:00 PM

**Objective**: To evaluate the risk factors associated with maternal abruption in twin‐twin transfusion syndrome (TTTS) after fetoscopic laser surgery (FLS).

**Study Design**: This retrospective study included TTTS cases that underwent FLS between 2011 and 2024 at a single institution. Maternal demographics and preoperative, intraoperative and postoperative outcomes were collected. Patients were subsequently grouped by abruption status. Placental abruption was diagnosed based on documentation in the medical record. Multiple logistic regression analysis was performed to identify risk factors, adjusted for maternal ethnicity, placental location, gestational age at FLS, amnioinfusion, amnioreduction, number of uterine entries, Solomonization, laser time and kilojoules, chorioamnion separation (CAS) observed on the first postoperative day, and preterm prelabor rupture of membrane (PPROM).

**Results**: 749 patients were enrolled in the study. Among them, 88 (11.7%) were complicated by maternal abruption. Patients with abruption delivered earlier than those without abruption (29.2 [26.3, 31.5] wks. vs 32 [28.5, 34.2] wks., p < 0.001). There were no significant statistical differences in maternal age, race, BMI, smoking status or other demographics or intraoperative variables between groups (Table 1). Rates of CAS (18.1% [16/88] vs. 9.6% [64/661], p = 0.04) and PPROM (61.3% [54/93] vs 37% [245/655], p = 0.001) were statistically significantly higher among the abruption group than the no abruption group. Multiple logistical regression demonstrated that CAS and PPROM are significant risk factors for placental abruption ([aOR, 2.04; 95% CI 1.01–4.11, p = 0.04] and [aOR, 2.16; 95% CI 1.29‐3.61, p = 0.003], respectively).

**Conclusion**: CAS and PPROM are significant risk factors for placental abruption. After FLS, patients with CAS and PPROM should be advised of the increased risk for placental abruption.

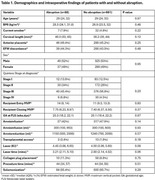

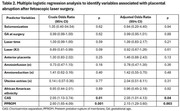

## 575 | Evaluation Of Severity Determination Using Transvaginal Ultrasound In Placenta Accreta Spectrum Scoring (TUPAS)

Neha Agarwal^1^; Sarah T. Mehl^2^; Edgar A. Hernandez‐Andrade^2^; Dejian Lai^3^; Gustavo Vilchez^4^; Eleazar E. Soto^2^; Farah H. Amro^2^; Rosa Guerra^5^; Sen Zhu^6^; Baha M. Sibai^2^; Sean C. Blackwell^2^; Ramesha Papanna^2^

*^1^Division of Fetal Intervention, Department of Obstetrics, Gynecology and Reproductive Sciences, UTHealth McGovern Medical School, Houston, TX, USA, Houston, TX; ^2^McGovern Medical School at UTHealth Houston, Houston, TX; ^3^UT School of Public Health, Houston, TX; ^4^UTHealth Houston, Houston, TX; ^5^Division of Gynecologic Oncology, Department of Obstetrics, Gynecology and Reproductive Sciences, McGovern Medical School at UTHealth Houston, Houston, TX; ^6^McGovern Medical School at the University of Texas, Houston, TX*

4:00 PM ‐ 6:00 PM

**Objective**: We showed that TUPAS, based on loss of uterine wall interface (LUS), arterial vascularity, and cervical involvement, can determine PAS severity based on blood loss during surgery. This study aims to validate the TUPAS in an external cohort and associate it with surgical outcomes.

**Study Design**: This is a retrospective cohort study of consecutive patients referred to our center for suspected PAS from 2023‐2024. Transvaginal ultrasound evaluated the LUS and cervix per predefined protocol (evaluating the placental‐uterine/cervical and bladder interface with bladder half full). Patients were managed per usual clinical approach, not based on TUPAS, including c‐section with placenta removal, c‐hysterectomy, and conservative management. An examiner blinded to patient history and clinical outcomes retrospectively scored images/clips. Cumulative scores of 0‐9 were converted to 4 categories as previously published (0: score 0; 1: score 1‐3; 2: score 4‐6; 3: score 7‐9). The primary outcome was calculated estimated blood loss (cEBL) during the primary surgery. Secondary outcomes were surgery type, blood product transfusion, ICU admission and length of hospital stay.

**Results**: 86 patients were referred: 78 were included in the analysis, 6 had incomplete or no images, 2 had placenta in the upper uterine segment. Maternal demographics and number of c‐section deliveries did not differ by category. Surgical management differed by category: all Cat‐0 patients and 82% in Cat‐1 had c‐section with placenta removal (Tab 1). Cystotomy occurred only in Cat‐3. From multiple linear regression analysis in log scale, Cat‐3(β = 0.94, p = 0.002) was associated with greater blood loss than other categories, irrespective of surgery type, on multiple linear regression. C‐hysterectomy in Cat‐3 was associated with higher cEBL (Fig1.), requiring massive transfusion, and more severe pathology than Cat‐2.

**Conclusion**: Our scoring strongly associated with severity of PAS with blood loss regardless of management approach. Cat 3 represents the most severe cases; referral to a tertiary care center specializing in PAS management should be considered.

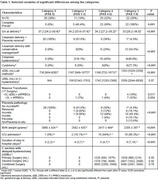

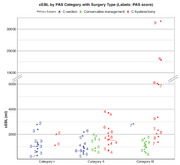

## 576 | Dietary Risk Factors for Excess Weight Gain in Pregnancy

Ngantu Le^1^; Megan L. Lawlor^1^; Jeannie C. Kelly^2^; Sarah K. England^3^; Antonina I. Frolova^4^; Nandini Raghuraman^2^; Peinan Zhao^3^

*^1^Washington University in St. Louis, St. Louis, MO; ^2^Washington University School of Medicine in St. Louis, St. Louis, MO; ^3^Washington University, St. Louis, MO; ^4^Washington University School of Medicine, St. Louis, MO*

4:00 PM ‐ 6:00 PM

**Objective**: Excess gestational weight gain (GWG) is associated with adverse pregnancy and metabolic outcomes. Patients are motivated to improve their health during pregnancy but describe a lack of knowledge on dietary recommendations. We investigated dietary components that are most associated with GWG.

**Study Design**: This is a nested case control study in a cohort of singleton pregnancies who completed the NIH Dietary Health Questionnaire II (DHQ‐II) in the third trimester or within 3 months of delivery. Cases were patients with excess GWG greater than the Institute of Medicine (IOM) recommended guidelines. Controls were patients with GWG within the IOM guidelines. Those with GWG below guidelines were excluded. Exposures of interest were Healthy Eating Index‐2015 (HEI‐2015) score components and other DHQ‐II dietary components. These components were compared between cases and controls. Multivariable logistic regression was used to adjust for BMI at first prenatal visit.

**Results**: 531 patients completed the DHQ‐II, among which 312 (58.8%) had excess GWG and 219 (41.2%) had appropriate GWG. There were no significant socioeconomic differences between those with excess and adequate GWG. The average BMI was 29.60±8.07 kg/m^2^ among patients with excess GWG and 27.20±7.49 kg/m^2^ among patients with appropriate GWG (P = 0.001). Overall HEI‐2015 scores were not significantly different between cases and controls. However, a diet composed of low whole grains (aOR 1.75, 95% CI 1.18‐ 2.60) or high dairy intake (aOR 1.84, 95% CI 1.18‐ 2.81) was associated with excess GWG.

**Conclusion**: A diet low in whole grains or high in dairy is associated with excess GWG. Obstetric providers should address specific dietary components when counseling patients about GWG.

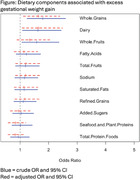

## 577 | Predicting Live Birth with the PRC2 Age Index, a Novel Epigenetic Biomarker

Nicola C. Perlman^1^; Andrea Cipriano^2^; Daniel J. Simpson^3^; Xixi D. Plummer^1^; Samantha L. Kruger^1^; Arian Korshid^2^; Janet Hurtado^1^; Ruth B. Lathi^1^; Danielle M. Panelli^1^; Vittorio Sebastiano^2^; Katherine Bianco^1^

*^1^Stanford University, Palo Alto, CA; ^2^Stanford University, Stanford, CA; ^3^Mayo Clinic, Rochester, MN*

4:00 PM ‐ 6:00 PM

**Objective**: Polycomb Repressive Complex 2 (PRC2) is a novel epigenetic biomarker of advanced cellular aging that has not yet been evaluated in pregnancy. PRC2 regions, present in all somatic cells, are associated with CpGs which gain DNA methylation with age. We evaluated whether PRC2 related methylation changes, as a marker of cellular aging, were associated with live birth in a preconception population.

**Study Design**: This pilot study utilized biobanked samples from patients desiring pregnancy at an academic center. Peripheral blood mononuclear cell DNA was extracted from buffy coat. Using the Illumina Infinium MethylationEPIC BeadChip array, we evaluated CpG methylation at PRC2 sites to generate the “PRC2 Age Index.” Mean PRC2 site methylation was reported for each patient. Patients were then grouped by chronologic age (age by birthdate) decade. The primary outcome was successful live birth, defined as pregnancy and delivery of live infant after 24 weeks. Using the PRC2 Age Index, mean methylation was compared between live birth and no live birth within chronologic age decades.

**Results**: 64 patient samples were analyzed. In the population, 57.2% had a live birth, of which 80% utilized assisted reproductive therapy. Demographics were overall similar between those with and without live birth. As expected, when the PRC2 Age Index was applied, patients in each chronologic age decade gained methylation. There were trends towards increased methylation of the PRC2 Age Index in people with no live birth across all chronologic age decades (Figure 1). Mean methylation was plotted for the live birth versus the no live birth groups (Figure 2).

**Conclusion**: The PRC2 Age Index correlated increased methylation at PRC2 sites with aging in a preconception population. Although insignificant, the PRC2 age index exhibited trends in increasing mean methylation for people without live birth, suggesting accelerated cellular aging compared to live birth counterparts. Larger studies evaluating the PRC2 Age‐Index may contribute to understanding of biological age and outcomes in a reproductive age population.

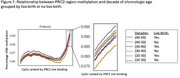

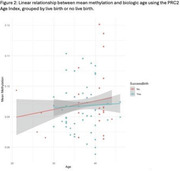

## 578 | The Association of Race and Ethnicity with Postpartum Hemorrhage Care Escalation

Nigel Madden; On behalf of the Eunice Kennedy Shriver National Institute of Child Health and Human Development Maternal‐Fetal Medicine Units Network

*Beth Israel Deaconess Medical Center, Boston, MA*

4:00 PM ‐ 6:00 PM

**Objective**: We aimed to evaluate whether there were racial and ethnic disparities in postpartum hemorrhage (PPH) care escalation after cesarean birth in a large, diverse cohort of birthing people.

**Study Design**: Secondary analysis of a multicenter placebo‐controlled randomized trial of prophylactic use of tranexamic acid during cesarean birth. We included participants who self‐reported as non‐Hispanic White (NHW), non‐Hispanic Black (NHB), or Hispanic and who had a PPH (estimated blood loss [EBL] > 1L). Based on a published algorithm for classifying PPH management, the primary outcome was level of PPH care intervention during the delivery admission: Level 0 (no intervention beyond standard third stage oxytocin), Level 1 (uterotonics only), Level 2 (performance of a procedure), and Level 3 (hysterectomy). We created multivariable ordinal regression models to evaluate the independent association between race/ethnicity and receipt of higher levels of anti‐hemorrhagic intervention. A secondary analysis stratified these results by PPH severity.

**Results**: Of 656 individuals who met inclusion criteria, 37% identified as NHW, 27% as NHB, and 36% as Hispanic. A majority received no interventions, with 360 (55%) Level 0, 154 (23%) Level 1, 118 (18%) Level 2, and 24 (4%) Level 3. NHB patients had a lower pre‐delivery hemoglobin (10.9 mg/dL NHB vs. 11.7 NHW vs. 11.8 Hispanic, p< 0.01). In adjusted analyses, NHB patients had lower odds of receiving higher levels of PPH care compared to NHW patients (aOR 0.48, 95% CI 0.31‐0.75, Table 1). As a proportion of Level 1 interventions, NHB patients received methylergonovine less often (9.0% NHB vs. 29.6% NHW vs. 30.3% Hispanic, p < 0.01). When stratified by severity of PPH, NHB patients had significantly lower odds of receiving higher levels of PPH care at an EBL between 1500‐1999mL (aOR 0.32, 95% CI 0.14‐0.78, Table 2).

**Conclusion**: NHB patients with PPH after cesarean birth had 50% lower odds of receiving higher levels of anti‐hemorrhagic intervention and this finding persisted at higher EBL. Racial differences in PPH care escalation may underlie disparities in PPH outcomes.

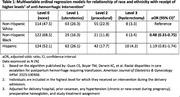

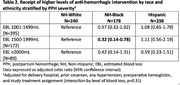

## 579 | Obesity and Weight Gain: How are the SMFM Annual Meetings Weighing in?

Nkechinyelum Ogu; Jacqueline C. Hairston

*Northwestern University Feinberg School of Medicine, Chicago, IL*

4:00 PM ‐ 6:00 PM

**Objective**: Obesity and weight gain are commonly understood risk factors for adverse pregnancy outcomes and the Society for Maternal‐Fetal Medicine (SMFM) Annual Meeting is one of the primary venues for high impact research on this topic. We sought to determine whether scientific presentations regarding obesity were focused on interventions to improve maternal and neonatal outcomes versus identification of risk factors.

**Study Design**: This is a descriptive study of abstract submissions related to obesity from the 2020 to 2024 SMFM Annual Meetings. Two independent researchers searched abstract titles with the following terms: “obesity”, “obese”, “weight gain”, “habitus”, “overweight”, “body mass index”, and “BMI”. The abstracts were then reviewed to determine if obesity was studied as a risk factor or if a clinical intervention was evaluated for the management of patients with obesity. Abstracts with multiple search terms in the title were only analyzed once and redundancies were eliminated. Only human studies were eligible for inclusion. The categorization of each abstract was compared and if a discrepancy arose a third‐party independent researcher provided final designation.

**Results**: Of the 6,023 abstracts presented at SMFM from 2020 to 2024, 254 (4.2%) were regarding obesity and weight gain and met criteria for inclusion. There were no abstracts with “habitus” or “overweight” in the title. 38 (15%) abstracts focused on interventions related to obesity and weight gain. 216 (85%) abstracts concentrated on obesity as a risk factor. 16 (6.3%) abstracts were oral presentations, of which eight featured interventions. Abstract categorization by year was assessed (Table 1).

**Conclusion**: Obesity is a well‐studied risk factor in perinatal outcomes, yet studies of interventions that aim to mitigate obesity‐related morbidity represent a minority of presented science on this topic. Further studies directed towards interventions and protocols to improve care surrounding obesity and weight gain are needed.

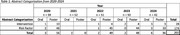

## 580 | The Association Between Group B Streptococcus Colonization and Long Term Infectious Morbidity of the Offspring

Noa Leybovitz Haleluya^1^; Tamar Wainstock^2^; Eyal Sheiner^2^

*^1^Soroka Medical Center, Meitar, HaDarom; ^2^Department of Obstetrics and Gynecology, Soroka University Medical Center, Ben‐Gurion University of the Negev, Beer‐Sheva, Israel., Beer Sheva, HaDarom*

4:00 PM ‐ 6:00 PM

**Objective**: Group B *Streptococcus* (GBS) colonizes the gastrointestinal and genital tracts of pregnant women. Determining colonization state near delivery time is an important determinant of infections in infants. Positive GBS carrier state is associated with short‐term infant morbidity and mortality.  However, long‐term effects were not thoroughly studied.  We aimed to study the association between positive test for GBS colonization near delivery and long‐term infectious morbidity of offspring.

**Study Design**: A population based cohort analysis was performed comparing infectious related pediatric morbidity among offspring to mothers with positive GBS status and negative or unknown GBS status. The analysis included all singletons born between the years 2002‐ 2021 at a single tertiary regional medical center. Infants with congenital malformations, multiple gestations, and cesarean deliveries were excluded from the analysis. Infectious related morbidities included hospitalizations or medical visits involving a pre‐defined set of ICD‐9 codes, as recorded in computerized files. A Kaplan‐Meier survival curve was constructed to compare the cumulative infectious morbidity, and a Cox proportional hazards model was used to adjust for confounders.  

**Results**: The study population included 146103 singletons. Positive GBS carrier state was recorded in 2225 (1.5%) patients. The cumulative incidence of long‐term infectious morbidity was higher among the offspring in the group of positive GBS (Figure; Log‐rank p< 0.001). The association remained significant and independent while adjusting for gestational age, maternal age, maternal hypertension and diabetes and ethnicity (Table, Adjusted HR = 1.15, 95%CI 1.09‐1.22, *p*< 0.001). 

**Conclusion**: GBS colonization in genital tract near delivery is independently associated with an increased risk for long‐term pediatric infectious morbidity of the offspring. Our results highlight the importance of GBS screening in pregnancy. In addition, we suggest using GBS status as a possible predictor for long‐term pediatric morbidities which may require special surveillance in this group of children.

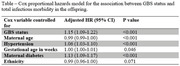

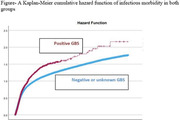

## 581 | Group B Streptococcus Vaginal Colonization and Long Term Respiratory Morbidity of the Offspring

Noa Leybovitz Haleluya^1^; Tamar Wainstock^2^; Eyal Sheiner^2^

*^1^Soroka Medical Center, Meitar, HaDarom; ^2^Department of Obstetrics and Gynecology, Soroka University Medical Center, Ben‐Gurion University of the Negev, Beer‐Sheva, Israel., Beer Sheva, HaDarom*

4:00 PM ‐ 6:00 PM

**Objective**: Group B *Streptococcus* (GBS) colonize the gastrointestinal and the genital tract. Vertical transmission primarily occurs following rupture of membranes or during passage through the birth canal. Early‐onset neonatal GBS infection manifests as sepsis, pneumonia, or meningitis, while late‐onset GBS disease most often presents as bacteremia. Both are associated with short‐term morbidity and mortality.  However, the long‐term effects are not well established.  We aimed to study the association between GBS colonization near and long‐term childhood respiratory morbidity of offspring.

**Study Design**: A retrospective population based cohort analysis was performed, in which respiratory pediatric morbidity was compared between offspring to mothers with positive GBS and offspring to mothers with negative or unknown GBS status. The analysis included all singletons born between the years 2002‐2021 at a tertiary medical center. Infants with congenital malformations, multiple gestations and cesarean deliveries were excluded from the analysis. Data for the diagnosis of respiratory morbidity was extracted from community‐based clinics and hospitalization records. A Kaplan‐Meier survival curve was constructed to compare the long‐ term cumulative respiratory morbidity, and a Cox proportional hazards model was used to adjust for confounders

**Results**: The study population included 146103 singletons. Positive GBS carrier state was recorded in 2225 (1.5%) patients. The cumulative incidence of long‐term respiratory morbidity was comparable between both groups (Figure, Log‐rank p = 0.540). Selected respiratory morbidities  groups are presented in the Table. Using a Cox proportional hazard model, no significant association was noted between GBS colonizationand the risk for long‐term pediatric respiratory morbidity, while adjusting for gestational age, maternal age, ethnicity, maternal hypertension and diabetes (Adjusted HR = 1.00, 95%CI 0.93‐1.08, *p* = 0.920). 

**Conclusion**: GBS colonization in maternal genital tract is not independently associated with a risk for long‐term pediatric respiratory morbidity of the offspring

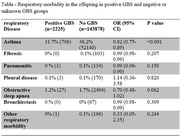

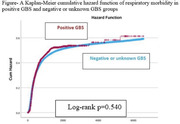

## 582 | Basal Insulin Type and Neonatal Outcomes: a Secondary Analysis of the Mompod Trial

Noelia Zork^1^; Qi Yan^1^; Gladys (Sandy) A. Ramos^2^; Celeste Durnwald^3^; Mark B. Landon^4^; On behalf of the MOMPOD consortium

*^1^Columbia University Irving Medical Center, New York, NY; ^2^University of California, San Diego, San Diego, CA; ^3^Hospital of University of Pennsylvania, Philadelphia, PA; ^4^The Ohio State University, Columbus, OH*

4:00 PM ‐ 6:00 PM

**Objective**: A variety of basal insulin types (e.g. intermediate vs long‐acting) are available for managing diabetes in pregnancy, however, the optimal choice of basal insulin remains uncertain in those with Type 2 diabetes (T2D) and diabetes diagnosed in early pregnancy (eDM). We aimed to compare neonatal outcomes according to basal insulin type.

**Study Design**: A secondary analysis of a randomized trial comparing the addition of metformin versus placebo in insulin treated pre‐existing T2D or eDM at < 23 weeks.  Included were those using either intermediate or long‐acting insulin at randomization, though specific insulin types were not recorded. The primary outcome was a composite of newborn complications (Table 2).  Categorical variables were compared between those on intermediate versus long‐acting insulin using Chi‐square or Fisher's exact test, and continuous variables using t‐test or Wilcoxon rank sum test. Logistic regression estimated the association between the basal insulin type and the composite outcome. Adjustments were made for confounding.

**Results**: A total of 812 participants were included in the analysis, 485 (59.7%) treated with intermediate and 327 (40.2%) with long‐acting insulin. There were several demographic differences between the groups (Table 1). The primary neonatal composite outcome was less common among participants treated with intermediate insulin (65.8% vs 72.8%, p = 0.035); however, after adjusting for confounders, there was no significant association (Table 2). There were no associations between basal insulin type and the individual components of the composite outcome.  A post hoc power analysis confirmed that the study had sufficient power to detect a 10% difference in the primary outcome.

**Conclusion**: In participants with T2DM and eDM, while basal insulin choice varied by maternal characteristics there was no significant difference in neonatal outcomes. Consistent with a shared decision making model, providers and patients can be reassured that their choice of basal insulin can be based on preference and/or insurance coverage without concerns for adverse nor varying neonatal outcomes.

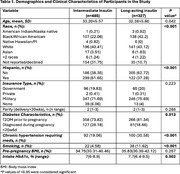

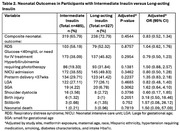

## 583 | The Triple Threat of Socioeconomic Insecurity: Housing, Financial, and Food Insecurity Association with Postpartum Depression

Noor Ali; Emily A. DeFranco

*Department of Obstetrics and Gynecology, University of Kentucky, Lexington, KY*

4:00 PM ‐ 6:00 PM

**Objective**: Examine the influence of housing, financial, and food insecurity on postpartum depression.

**Study Design**: Using data from the Pregnancy Risk Assessment Monitoring System (PRAMS) Core and Standard Questionnaire from 2016 to 2021 linked to US birth certificate data, a retrospective cohort study was performed to quantify the relative risk of the exposures of housing, financial, and/or food insecurity on the outcome of postpartum depression. Pre‐pregnancy depression was determined by anyone who reported depression in the three months before pregnancy. Postpartum depression was quantified by a previously created variable by PRAMS titled “Postpartum Depression Indicator.” Participant surveys occurred at 2‐6 months postpartum. Generalized linear modeling (GLM) estimated adjusted relative risk (adjRR) and 95% CIs while accounting for the confounding influences of maternal race, marital status, education, and WIC utilization.  

**Results**: 221,381 Postpartum patients completed the Pregnancy Risk Assessment Monitoring System (PRAMS) Core and Standard Questionnaire. Of those surveyed, 11,482 (5.2%), 373 (3.2%), and 4,302 (3.0%) reported financial, food, and housing insecurity, respectively, and 15,768 (7.1%) had any of the three. The prevalence of reported depression was 15.8% before pregnancy and 14.4% postpartum. Each exposure was associated with a significantly higher risk of pre‐pregnancy and postpartum depression, even after adjusting for race, marital status, education, and WIC usage (see table). The highest individual risks for depression were observed with food insecurity, with greater than 2‐fold increased risk for depression before pregnancy and postpartum.   

**Conclusion**: The physical and social environments of birthing people can impact their psychological state. Postpartum depression can influence the bond between the mother‐infant dyad, potentially contributing to adverse psychosocial outcomes. Public health initiatives should focus on improving access to financial, food, and housing resources for reproductive‐age people to mitigate postpartum depression effects. 

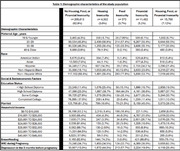

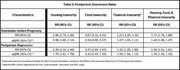

## 584 | Outcomes Among Patients in a Remote Postpartum Blood Pressure Monitoring Program: Does Medication Choice Matter?

Ntami P. Echeng^1^; Shana D. Talbot^1^; Scott Machado^2^; Tracy L. Jackson^2^; Nicole Konecke^2^; William Hunt^2^; Danielle Simmons^2^; Maria Mejia Castillo^2^; William A. Grobman^3^; Alisse Hauspurg^4^; Methodius G. Tuuli^1^; Adam K. Lewkowitz^2^

*^1^Women & Infants Hospital of Rhode Island / Alpert Medical School of Brown University, Providence, RI; ^2^Women & Infants Hospital of Rhode Island / Alpert Medical School of Brown University., Providence, RI; ^3^The Ohio State University, Columbus, OH; ^4^Alpert Medical School of Brown University, Providence, RI*

4:00 PM ‐ 6:00 PM

**Objective**: Participants in postpartum self‐measured blood pressure (SMBP) programs can have antihypertension medications initiated remotely, thereby preventing severe hypertension (HTN) and reducing HTN‐related readmissions, emergency department (ED) presentations, and severe maternal morbidity (SMM). Nifedipine and labetalol are commonly prescribed first‐line HTN medications, although outcomes related to their use may differ.  We aimed to compare outcomes between participants in a postpartum remote SMBP program who were prescribed nifedipine versus labetalol after hospital discharge.

**Study Design**: Postpartum patients with HTN at our tertiary care hospital are offered enrollment in our remote 6‐week SMBP program, during which antihypertensives are prescribed for BP >140/90 mmHg. For this analysis, participants were stratified by antihypertensive prescribed (nifedipine versus labetalol). Patients who were prescribed a different medication or who were prescribed both medications were excluded. The primary outcome was a composite of postpartum readmission or ED presentation for HTN within 30 days of delivery hospitalization. Secondary outcomes included HTN‐related SMM.  A generalized linear model was used to estimate relative risks (RR) after adjustment for differences in medical conditions.

**Results**: Among 2003 remote SMBP participants, 446 (22.3%) were prescribed either labetalol alone (n = 104; 23%) or nifedipine alone (n = 342; 77%) after hospital discharge. Differences between groups were identified in type of HTN diagnosis (Table 1). After controlling for these differences, the rate of the composite outcome of HTN‐related postpartum readmission or ED presentation was similar between those who received labetalol (n = 23 (22.1%)) or nifedipine (n = 65 (19.1%); adjusted relative risk = 1.26 (0.81, 1.94) (Table 2). There were no differences between groups for secondary outcomes.

**Conclusion**: In our remote SMBP program for postpartum patients with HTN, there was no difference in risk of HTN‐related healthcare utilization between those prescribed labetalol alone or nifedipine alone for persistent HTN after hospital discharge.

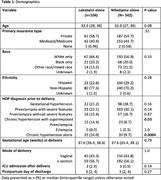

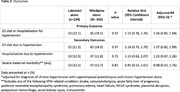

## 585 | Association Between Treatment Modalities for Gestational Diabetes Mellitus and Risk of Attention‐Deficit/Hyperactivity Disorder in Offspring

Ofer Fischer^1^; Shaked Yarza^2^; Eran Hadar^3^; Asnat Walfisch^4^; Keren Tzadikevitch Geffen^3^

*^1^Helen Schneider Hospital for Women, Rabin Medical Center, Kfar Hess, HaMerkaz; ^2^Meir medical center, Kfar Saba, HaMerkaz; ^3^Rabin Medical Center, Petach Tikva, HaMerkaz; ^4^Helen Schneider Hospital for Women, Rabin Medical Center,, Petah Tikva, HaMerkaz*

4:00 PM ‐ 6:00 PM

**Objective**: The association between gestational diabetes mellitus (GDM) and long‐term neurodevelopmental disorders (NDD), particularly attention‐deficit/hyperactivity disorder (ADHD), remains a topic of debate. We aimed to explore the association between GDM, either dietary controlled (GDMA1) or pharmacologic treated (GDMA2) and the risk of ADHD in the offspring.

**Study Design**: Retrospective population‐based study included all deliveries in “Clalit” HMO hospitals from 2012 to 2020. Women with unknown diabetic status during pregnancy or with pregestational diabetes were excluded from the study. Offspring were followed through electronic medical records starting at age 4 years.

**Results**: Among 15,074 children exposed to maternal GDM (13,566 GDMA1, 1,508 GDMA2) and 187,112 children born to mothers with normal glycemic index (control group), 13,446 were diagnosed with ADHD, median age was 6.5 years with interquartile range (IQR) 2.15 years. The incidence of ADHD was significantly higher in the GDM groups compared to 6.6% in the control group; 7.4% (pV< 0.001) in the GDMA1 group, and 8.5% (pV< 0.001) in the GDMA2 group. The adjusted odds ratios (aOR) for ADHD were 1.30 (95% CI: 1.06‐1.57, p = 0.008) for children exposed to GDMA2 and 1.09 (95% CI: 1.01‐1.16, p = 0.02) for those exposed to GDMA1, compared to unexposed children. Additionally, gestational hypertension during pregnancy was identified as a significant risk factor for ADHD, with an aOR of 1.19 (95% CI: 1.003‐1.39, p = 0.04).

**Conclusion**: Maternal GDM significantly increases the risk of ADHD in exposed offspring. The need for pharmacologic intervention to control gestational diabetes further amplifies this risk.

## 586 | False Positive Prenatal Diagnosis of Placenta Accreta Spectrum: Factors Associated with Overdiagnosis and Perinatal Outcomes

Olga Grechukhina^1^; Colleen Sinnott^2^; Khadija Alshowaikh^1^; Jennifer F. Culhane^1^; Lisbet S. Lundsberg^1^; Caitlin Partridge^1^; Mert Ozan Bahtiyar^2^; Sonya Abdel‐Razeq^1^

*^1^Yale School of Medicine, New Haven, CT; ^2^Yale University, New Haven, CT*

4:00 PM ‐ 6:00 PM

**Objective**: Accurate prenatal prediction of placenta accreta spectrum (PAS) is known to improve maternal and neonatal outcomes. However, when striving to increase sensitivity of prenatal sonographic identification of PAS, the risk of overdiagnosis also increases.  We aim to identify factors associated with false positive prenatal diagnosis of PAS and the impact on perinatal outcomes.

**Study Design**: All delivery encounters at a single academic institution from 2013‐2023 were queried for ICD‐10 codes for PAS. Cases (n = 775) were then manually reviewed and those with any degree of prenatal suspicion for PAS but no evidence of PAS at the time of delivery were included (n = 181). For each case, two variables were created by a consensus of 4 clinical experts: prenatal suspicion (low ‐ true negative; or high ‐ false positive diagnosis), determined by prenatal ultrasound reports and documentation of delivery planning, and *a priori* risk for PAS (low or high) based on clinical risk factors (i.e prior uterine surgeries and placenta previa). Other patient attributes and perinatal outcomes were identified through the EMR. Bivariate analyses comparing low *vs* high prenatal suspicion and patient attributes were conducted. Multivariate logistic regression was performed on significantly different outcome measure ‐ preterm birth (PTB) ‐ adjusting for covariates significant at p< 0.05.

**Results**: Of the 181cases, 73 (40.3%) were coded as high prenatal suspicion. Advanced maternal age, race, multiparity, higher gestational age at initial prenatal visit and ultrasound, high *a priori* risk and placenta previa were associated with high prenatal suspicion for PAS. High prenatal suspicion was an independent risk factor for PTB after adjusting for *a priori* risk and placenta previa (aOR 4.6, 95% 2.1‐10.2, p< 0.0001).

**Conclusion**: False positive PAS diagnosis (prenatal high suspicion) results in 4.6 times increased risk of iatrogenic preterm birth compared to those with low suspicion. New tools are urgently needed to accurately prenatally rule out PAS to decrease the risk of iatrogenic adverse neonatal outcomes.

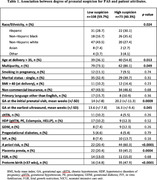

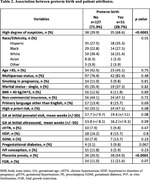

## 587 | Can B‐type Natriuretic Peptide (BNP) help Differentiate Gestational Hypertension and Preeclampsia from Chronic Hypertension?

Olivia Grubman^1^; Mackenzie MItchell^2^; Christina Cary^2^; Mia A. Heiligenstein^3^; Thomas Owens^4^; Guillaume Stoffels^5^; Zainab Al‐Ibraheemi^4^; Desmond M. Sutton^6^; Lois Brustman^4^

*^1^Westchester Medical Center, Westchester, NY; ^2^Icahn School of Medicine, New York, NY; ^3^Mount Sinai West, Astoria, NY; ^4^Icahn School of Medicine at Mount Sinai West, New York, NY; ^5^Icahn School of Medicine at Mount Sinai, New York, NY; ^6^Mount Sinai West Hospital, New York, NY*

4:00 PM ‐ 6:00 PM

**Objective**: B‐type natriuretic peptide (BNP) is a cardiac biomarker that is synthesized and secreted from the cardiac ventricles in response to volume expansion and pressure overload.  The primary objective was to determine if BNP value could help differentiate gestational hypertension, preeclampsia without severe features and preeclampsia with severe features from chronic hypertension (CHTN). Given the acuity of gestational hypertension (GHTN) and preeclampsia (PEC) versus the chronic nature of preexisting CHTN, we hypothesized that a higher BNP value will be associated with GHTN and PEC compared to CHTN. 

**Study Design**: This was a retrospective cross‐sectional study involving 391 patients evaluated for hypertension in pregnancy from January 2022 to February 2023 in an urban teaching hospital. CHTN, GHTN and PEC were defined according to ACOG. Those patients with superimposed preeclampsia were excluded. Demographic characteristics, BNP value, hypertension diagnosis and delivery information were collected for all patients. BNP value was collected on the labor and delivery unit at the time of initial evaluation for hypertensive disease.

**Results**: The study population consisted of 391 patients. There were 326 patients in the GHTN/PEC group (149 with GHTN, 54 with PEC w/oSF & 123 with PEC w/SF) and 65 in the CHTN group. There was no significant difference in patient demographics between the two groups. The mean BNP level for the GHTN/PEC group was 22.0 pg/mL, whereas the mean BNP level for the CHTN group was 11.2 pg/mL, which was significantly different (p = 0.0004) (Figure 1 & Table 1).

**Conclusion**: This study demonstrates that BNP levels are significantly higher in patients with GHTN/PEC compared to those with CHTN. Therefore, BNP may be a useful test in helping to differentiate between CHTN and GHTN/PEC at the time of initial diagnosis.This is important given the differences in management and delivery implications for each disease.

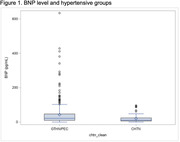

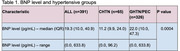

## 588 | Evaluating the Impact of Labor Induction on Autism Spectrum Disorder Risk

Omri Zamstein^1^; Tamar Wainstock^2^; Eyal Sheiner^2^

*^1^Soroka University Medical Center, Soroka University Medical Center, HaDarom; ^2^Department of Obstetrics and Gynecology, Soroka University Medical Center, Ben‐Gurion University of the Negev, Beer‐Sheva, Israel., Beer Sheva, HaDarom*

4:00 PM ‐ 6:00 PM

**Objective**: Significant effort has been dedicated in recent years to uncovering the environmental factors, particularly perinatal exposures, that contribute to the development of autism spectrum disorder (ASD). However, the overall picture remains incomplete, with many suggested associations lacking consistency. Given the frequent use of labor inductions for medical reasons as well as patient preference, we aimed to investigate their relationship with ASD development, considering synergistic factors that may influence ASD onset.

**Study Design**: A population‐based cohort study was conducted, focusing on singleton births. The study aimed to compare the occurrence of ASD in children, considering both hospital and community‐based diagnoses, in relation to whether labor was induced (using mechanical cervical ripening or prostaglandins, with or without oxytocin) or began spontaneously. A Kaplan‐Meier survival curve was employed to assess the cumulative ASD incidence, and a Cox proportional hazards model was used to account for confounders.

**Results**: Among 115,081 births, 13,071 (11.4%) were labor induced, with the remainder beginning spontaneously. Pregnancy complications, such as gestational diabetes mellitus, preeclampsia or eclampsia, and non‐reassuring fetal heart rate patterns, were significantly more common in the labor induction group (p< 0.001 for all). Conversely, preterm births were more prevalent in the spontaneous labor onset group (7.1% vs. 5.3%, p< 0.001; Table). During follow‐up, 767 children were diagnosed with ASD: 1.0% in the labor induction group and 0.6% in the spontaneous labor onset group (p< 0.001). The Kaplan‐Meier analysis showed a significantly higher cumulative hazard for ASD diagnosis in the labor induction group (log‐rank p‐value < 0.001; Figure). However, after adjusting for maternal, delivery, and fetal factors such as age, cesarean delivery, ethnicity, and gestational conditions, no significant association was found between labor induction and ASD risk (adjusted HR = 1.21, 95% CI 0.99–1.47, p = 0.063).

**Conclusion**: Labor induction does not appear to be associated with an increased risk of ASD.

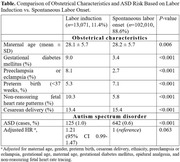

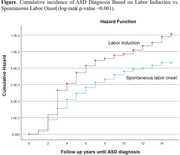

## 589 | Gender as a Determinant of Neurological Sequelae in Twin Gestations

Omri Zamstein^1^; Tamar Wainstock^2^; Eyal Sheiner^2^

*^1^Soroka University Medical Center, Soroka University Medical Center, HaDarom; ^2^Department of Obstetrics and Gynecology, Soroka University Medical Center, Ben‐Gurion University of the Negev, Beer‐Sheva, Israel., Beer Sheva, HaDarom*

4:00 PM ‐ 6:00 PM

**Objective**: Evidence suggests that biological and genetic differences between genders may influence the prevalence and severity of various perinatal and chronic health outcomes. To further investigate these discrepancies, we conducted a detailed evaluation of the long‐term neurological outcomes in male and female offspring from twin gestations.

**Study Design**: A population‐based sibling analysis was conducted at a tertiary referral center, focusing on twin births of male‐female dyads. The study aimed to compare the occurrence of neurological‐related morbidity throughout childhood, considering both community and hospital‐based diagnoses, in relation to offspring gender. A Kaplan‐Meier survival curve was utilized to evaluate the cumulative incidence of neurological morbidity, while a Cox proportional hazards model was applied to control for confounding variables.

**Results**: The analysis of 1,471 twin pairs revealed gender differences in neurological and psychiatric conditions (Table). Males exhibited higher rates of ADHD (8.3% vs. 3.5%, p< 0.001) and total neurological‐related morbidity (21.5% vs. 17.4%, p = 0.006). Notably, sleep disorders were only reported in males (0.3%, p = 0.02), and developmental disorders were also more prevalent among males compared to females (4.3% vs. 2.5%, p = 0.008). The Kaplan‐Meier analysis demonstrated an increased cumulative neurological morbidity among male offspring (log‐rank p‐value = 0.004; Figure). In a Cox regression hazards model, which accounted for mode of delivery and small‐for‐gestational‐age neonates, male gender remained independently associated with long‐term neurological sequelae (aHR = 1.28, 95% CI 1.08‐1.51, p = 0.004).

**Conclusion**: Male offspring from twin gestations are notably more prone to experiencing long‐term neurological morbidity.

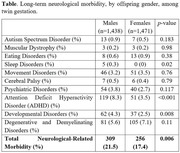

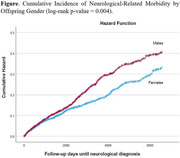

## 590 | Labor Progression in Women with or without Premature Rupture of Membranes

Or Bercovich^1^; Daniela Chen^2^; Natav Hendin^1^; Shay Sukenik^3^; Eran Hadar^1^; Ohad Houri^1^

*^1^Rabin Medical Center, Petach Tikva, HaMerkaz; ^2^Helen Schneider Hospital for Women, Rabin Medical Center, Petach‐Tikva, Tel Aviv, HaMerkaz; ^3^Beilinson, Tel Aviv, HaMerkaz*

4:00 PM ‐ 6:00 PM

**Objective**: To compare labor progression in women with premature rupture of membranes (PROM) versus those without PROM.

**Study Design**: Retrospective study conducted at a tertiary university medical center between July 2012 and September 2020. Women carrying singleton pregnancies who underwent vaginal delivery at or beyond 37 weeks of gestation were included. Exclusion criteria included multiple gestations, non‐vertex presentations, and cesarean deliveries. The cohort was divided into two groups: PROM and No‐PROM. Interval‐censored regression estimated median labor duration at each centimeter of cervical dilatation. Multivariate analyses adjusted for maternal age, chronic hypertension, thrombophilia, and gestational age were conducted.

**Results**: 26,438 women were included, with 6,626 (25.1%) in the PROM group and 19,812 (74.9%) in the No‐PROM group. In primiparous women, first stage of labor (3‐10cm) was significantly shorter in the PROM compared to the No PROM group, with medians of 5.00 (95^th^ percentile 12.79) versus 5.48 hours (95^th^ percentile 13.91; P< 0.001). Similarly, in multiparous women, the median times were 2.70 (95^th^ percentile 8.68) versus 3.16 hours (95^th^ percentile 10.06; P< 0.001). The active phase (6‐10 cm) did not significantly differ in primiparous women with medians of 2.76 (95^th^ percentile 8.53) versus 2.84 hours (95th percentile 9.01; P = 0.394). However, multiparous women with PROM had a shorter active phase with medians of 1.02 (95^th^ percentile 4.16) versus 1.16 hours (95^th^ percentile 4.82; P = 0.002).

**Conclusion**: Labor progression from 3 cm to full dilatation was significantly shorter in women with PROM compared to those without PROM, regardless of parity. Active phase duration was statistically significantly shorter in multiparous women with PROM, albeit the median difference of 8 minutes is not clinically significant.

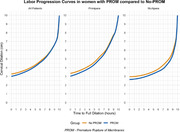

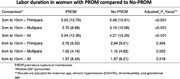

## 591 | Labor Progression in Women with SGA versus AGA Newborns

Or Bercovich^1^; Daniela Chen^2^; Natav Hendin^1^; Shay Sukenik^3^; Eran Hadar^1^; Ohad Houri^1^

*^1^Rabin Medical Center, Petach Tikva, HaMerkaz; ^2^Helen Schneider Hospital for Women, Rabin Medical Center, Petach‐Tikva, Tel Aviv, HaMerkaz; ^3^Beilinson, Tel Aviv, HaMerkaz*

4:00 PM ‐ 6:00 PM

**Objective**: To compare labor progression in women with small for gestational age (SGA) newborns versus those with appropriate for gestational age (AGA) newborns.

**Study Design**: Retrospective study at a tertiary university medical center from July 2012 to September 2020. Women with singleton pregnancies at or beyond 37 weeks of gestation were included. Exclusion criteria were multiple gestations, non‐vertex presentations, previous multiple cesarean deliveries, and neonates with congenital anomalies, 5‐minute Apgar  < 7, or admission to the intensive care unit. The cohort was divided into SGA (birth weight < 10^th^ percentile) and AGA (birth weight 10‐90^th^ percentile) newborns.

Interval‐censored regression estimated median labor durations at each centimeter of cervical dilatation. Multivariate analyses adjusted for maternal age, BMI, and gestational age.

**Results**: 23,629 women were included, with 2,283 (9.66%) in the SGA group and 21,346 (90.33%) in the AGA group. In both primiparous and multiparous women, the duration of the first stage of labor (3‐10cm dilatation) was not significantly different between the SGA and AGA groups, with medians of 4.45 (95^th^ percentile 13.31) vs. 4.65 hours (95^th^ percentile 12.27; P = 0.54) in primiparous women and 2.49 (95^th^ percentile 9.73) vs. 2.72 hours (95^th^ percentile 9.34; P = 0.88) in multiparous women, respectively. The active phase of labor (6‐10cm dilatation) was shorter in primiparous women with SGA compared to AGA, with a median of 1.92 (95^th^ percentile 7.61) vs. 2.22 hours (95^th^ percentile 7.58; P< 0.01). However, no significant difference was observed in multiparous women, with medians of 0.64 hours (95^th^ percentile 4.08) vs. 0.84 hours (95^th^ percentile 4.45; P = 0.95) in the SGA and AGA groups, respectively.

**Conclusion**: Women with SGA and AGA newborns have a similar duration of labor overall. Although active phase of labor was statistically significantly shorter in primiparous women with SGA, the difference of 18 minutes is of limited clinical significance.

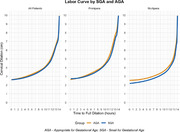

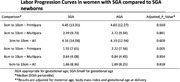

## 592 | Fetal Head Circumference Underestimation of Neonatal Head Circumference: Results from a Prospective Multicenter Study

Patrick Dicker^1^; Naomi Burke^1^; Fergal D. Malone^2^

*^1^Royal College of Surgeons in Ireland, Dublin, Dublin; ^2^Royal College of Surgeons in Ireland, Dubiln, Dublin*

4:00 PM ‐ 6:00 PM

**Objective**: Being able to prospectively predict difficult labor, and the need for operative vaginal delivery or cesarean delivery, is of great value in contemporary obstetric practice, in particular for nulliparous patients. Increasing fetal head circumference (FHC) has been the focus of predictors in this regard, but this assumes that prenatal ultrasound assessment of FHC is an accurate surrogate marker for neonatal head circumference (NHC). 

**Study Design**: We evaluated the accuracy of prenatal FHC measurement for predicting NHC in a secondary analysis of a prospective multicenter study of nulliparous term labor. 2,336 pregnancies with an ultrasound examination at 39 weeks’ gestation were included, in which FHC was measured in triplicate and averaged. NHC was measured at birth by a standard Holtain measuring tape, placed 2cm above the ears around the occipito‐frontal circumference. Linear regression was used to compare differences in FHC and NHC, adjusting for time to delivery (TTD) in days. An alternative approach without adjustment for TTD, FHC and NHC were compared using the 90^th^ centiles from the INTERGROWTH‐21^st^ study for FHC and NHC.

**Results**: The median [IQR] gestational age at ultrasound examination was 39.3 weeks [39.0 ‐ 39.6], and FHC was 337mm [329‐345]. The median [IQR] gestational age at delivery was 40.7 weeks [40.3–41.3], and NHC was 350mm [340‐360]. Regression analysis indicated that FHC underestimated NHC by 10.5mm on average (difference = ‐10.5–0.37 * TTD, see Figure 1). Centile‐based comparisons found that 17% of FHC measurements were above the 90^th^ centile, compared to 30% of NHC values above the 90^th^ centile.

**Conclusion**: Late pregnancy ultrasound measurement of FHC underestimates neonatal head circumference by approximately 1cm at term, and a centile‐based comparison did not compensate for this difference. This systematic error may have important implications and should be taken into account in the interpretation of fetal head circumference.

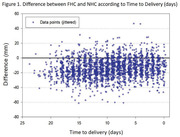

## 593 | Suboptimal Fetal Lung Growth After Fetal Endoluminal Tracheal Occlusion in Congenital Diaphragmatic Hernia: Clinical Characteristics

Percy N. Pacora‐Portella^1^; Mary T. Austin^2^; Sami Backley^3^; Eric P. Bergh^4^; Ashley Ebanks^5^; Jimmy Espinoza^3^; Matthew T. Harting^6^; Edgar A. Hernandez‐Andrade^7^; Katrina S. Hughes^8^; Suzanne Lopez^9^; Ramesha Papanna^7^; KuoJen Tsao^10^; Anthony Johnson^11^

*^1^Division of Fetal Intervention, Dpt Obstetrics,Gynecol and Reprod Sciences, McGovern Medical School at UTHealth Houston, Houston, TX; ^2^Department of Pediatric Surgery, McGovern Medical School at UT Health Houston, Houston, TX; ^3^Division of Fetal Intervention, Department of Obstetrics, Gynecology and Reproductive Sciences, McGovern Medical School at UTHealth Houston, Houston, TX; ^4^UT Health, Houston, TX; ^5^Comprenhensive Center for CDH Care. Department Pediatric Surgery. McGovern Medical School. UTHealth Houston, Houston, TX; ^6^Division of General & Thoracic Pediatric Surgery.McGovern Medical School.UTHealth Houston, Houston, TX; ^7^McGovern Medical School at UTHealth Houston, Houston, TX; ^8^McGovern Medical School. UThealth Houston, Houston, TX; ^9^McGovern Medical School. UTHealth Houston, Houston, TX; ^10^McGovern Medical School at the University of Texas Health Science Center at Houston (UTHealth) University of Texas Health Science Center, Houston, TX; ^11^McGovern Medical School University of Texas Health Science Center at Houston (UThealth), Houston, TX*

4:00 PM ‐ 6:00 PM

**Objective**: Suboptimal fetal lung growth (SFLG) affects cases that undergo Fetoscopic endoluminal tracheal occlusion(FETO) in severe left‐sided congenital diaphragmatic hernia (L‐CDH).We describe the demographic and clinical characteristics of SFLG  and optimal fetal lung growth (OFLG) after FETO

**Study Design**: Singleton pregnancies with isolated L‐CDH, defined as observed‐to‐expected lung‐to‐head ratio (O/E LHR) < 25% and normal chromosomes, underwent FETO under an IRB‐ and FDA‐approved clinical trial protocol (NCT02596802) at the UTHealth Houston Fetal Center from 2015‐2024. The FETO  balloon insertion was scheduled at 27‐30 weeks' gestation (wGA), and balloon removal at 34‐35‐wGA.Observed‐to‐expected total fetal lung volume (O/E TFLV) was measured by MRI before and 3 weeks after FETO, and percent changes from pre‐ to post‐FETO MRIs were calculated.Descriptive, analytical statistics and (ROC) curve analysis associating metrics with neonatal survival and the need for extracorporeal life support (ECLS)  were performed via R interface(jamovi.org).

**Results**: 19 pregnant women were enrolled, 4 children died, 2  cases did not undergo the post‐FETO‐MRI, 2 cases died before surgical repair.Area under the ROC curve(AUC) of O/E TFLV for the prediction of survival and not need for ECLS were 0.974, and  0.729, respectively.The best cut‐off for identification of SFLG was an increase of < 12.8%  of O/E TFLV 21 days post‐FETO with a sensitivity of 92.3% and specificity of 100% to predict childhood mortality at hospital discharge.An increase of ≥ 12.8 % of TFLV(OFLG) had a sensitivity of 90% and specificity of 57.1% to predict children that do not require ECLS.  Demographic and maternal characteristics were not different between  SFLG and OFLG(Table 1). SFLG  had lower  O/E TFLV and O/E LHR three weeks post‐FETO, and was associated with a lower frequency of surviving hospital discharge than OFLG(Table 2).                                                                                                                                                                                                                                                                                                            

**Conclusion**: SFLG, characterized by decreased lung volume, is associated with a lower survival than optimal fetal lung growth.   

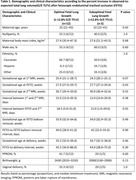

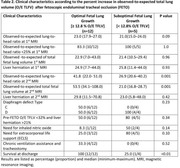

## 594 | Sleepiness in Pregnancy and Adverse Perinatal Outcomes

Rachel L. Friedlander^1^; Xiaoning Huang^2^; Phyllis Zee^3^; Sadiya S. Khan^3^; Philip Greenland^4^; Francesca Facco^5^; Judith H. Chung^6^; William A. Grobman^7^; David M. Haas^8^; Uma M. Reddy^9^; George R. Saade^10^; Robert M. Silver^11^; Lynn M. Yee^3^

*^1^Northwestern Feinberg School of Medicine, Chicago, IL; ^2^Northwestern University, Chicago, IL; ^3^Northwestern University Feinberg School of Medicine, Chicago, IL; ^4^Northwestern, Northwestern/Chicago, IL; ^5^University of Pittsburgh, Pittsburgh, PA; ^6^University of California‐ Irvine, Irvine, CA; ^7^The Ohio State University, Columbus, OH; ^8^Indiana University School of Medicine, Indianapolis, IN; ^9^Columbia University, New York, NY; ^10^Macon & Joan Brock Virginia Health Sciences Eastern Virginia Medical School at Old Dominion University, Norfolk, VA; ^11^University of Utah, Salt Lake City, UT*

4:00 PM ‐ 6:00 PM

**Objective**: Sleep disorders in pregnancy have been associated with adverse perinatal outcomes (APO) including hypertensive disorders of pregnancy (HDP), preterm birth (PTB), and gestational diabetes mellitus (GDM), yet prospective studies that use validated tools to assess sleepiness in pregnancy are limited. We aimed to assess the association of sleepiness in pregnancy with APOs.

**Study Design**: In this secondary analysis of data from a large, diverse observational cohort of nulliparous individuals conducted at eight US medical centers, those with self‐reported sleep data were included. Sleepiness was evaluated in early (6‐13 weeks) and mid pregnancy (22‐29 weeks) via the Epworth Sleepiness Scale, a validated tool to assess daytime sleep propensity, the ability to fall or stay asleep. Those with scores ≤10 were categorized as having no sleepiness (referent), 11‐15 moderate sleepiness, and 16‐24 severe sleepiness. The outcomes were HDP, GDM, PTB, and small‐for‐gestational‐age birth < 10%ile (SGA). Multivariable logistic regression analysis was performed to evaluate the associations of moderate and severe sleepiness with each APO. A sensitivity analysis that included “high risk for sleep disordered breathing (SDB),” measured by an adapted Berlin Questionnaire for Sleep Apnea, as a covariate in the multivariable model was performed.

**Results**: Of 6,806 eligible participants who completed sleep questionnaires at both time points, the average sleepiness score in early and mid pregnancy was 7.8 (SD 4.1) and 6.5 (SD 4.0) respectively. In early pregnancy, 20.0% and 4.3% of people experienced moderate and severe sleepiness, respectively; in mid pregnancy 14.4% and 2.5% experienced moderate and severe sleepiness. Severe sleepiness in mid‐pregnancy was associated with GDM (aOR 1.78, 95% CI 1.01‐3.12) and SGA (aOR 1.35, 95% CI 1.06‐1.71) (Table). Findings were similar when accounting for SDB.

**Conclusion**: Severe sleepiness during pregnancy, a symptom reflective of sleep quality, length, and often SDB, is associated with GDM and SGA suggesting a potential relationship with maternal glucose metabolism and placental function.

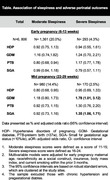

## 595 | Association of Sleepiness in Pregnancy with Cardiovascular Health 2‐7 years after first birth

Rachel L. Friedlander^1^; Xiaoning Huang^2^; Sadiya S. Khan^3^; Phyllis Zee^3^; Philip Greenland^4^; Natalie A. Bello^5^; Janet Catov^6^; Bethany B. Gibbs^7^; William A. Grobman^8^; David M. Haas^9^; Lisa D. Levine^10^; C. Noel Bairey Merz^11^; Eliza C. Miller^12^; Uma M. Reddy^13^; Lynn M. Yee^3^

*^1^Northwestern Feinberg School of Medicine, Chicago, IL; ^2^Northwestern University, Chicago, IL; ^3^Northwestern University Feinberg School of Medicine, Chicago, IL; ^4^Northwestern, Northwestern/Chicago, IL; ^5^Cedars Sinai Medical Center, Los Angeles, CA; ^6^Magee‐Womens Hospital, Pittsburgh, PA; ^7^West Virginia University, Morgantown, WV; ^8^The Ohio State University, Columbus, OH; ^9^Indiana University School of Medicine, Indianapolis, IN; ^10^University of Pennsylvania Perelman School of Medicine, Philadelphia, PA; ^11^Cedars‐Sinai Medical Center, Los Angeles, CA; ^12^Columbia University Medical Center, New York, NY; ^13^Columbia University, New York, NY*

4:00 PM ‐ 6:00 PM

**Objective**: Excessive daytime sleepiness has been associated with cardiovascular disease in non‐pregnant populations. This study examined the association of sleepiness during pregnancy and cardiovascular health (CVH) scores 2‐7 years after first birth.

**Study Design**: In this secondary analysis of longitudinal data from a large, diverse cohort of nulliparas conducted at eight US medical centers, individuals were followed 2‐7 years after their first birth. The Epworth Sleepiness Scale, a validated tool to assess daytime sleep propensity (i.e. the likelihood of falling or staying asleep), was used to evaluate sleepiness in early (6‐13 weeks) and mid‐ pregnancy (22‐29 weeks). Individuals with scores ≤10 were categorized as having no sleepiness (referent), 11‐15 as moderate sleepiness, and 16‐24 severe sleepiness. The primary outcome was CVH quantified by the American Heart Association's Life's Essential 8 (LE8) score, which is a validated surrogate of cardiovascular disease risk, with lower scores representing worse CVH. The LE8 score includes diet, physical activity, nicotine exposure, sleep, body mass index, blood pressure, blood glucose, and lipids, and ranges from 0 (worst) to 100 (best). Separate multivariable linear regression models were performed for early and mid‐pregnancy adjusting for maternal age, insurance type, smoking status, and study site.

**Results**: Of 2,193 participants, moderate and severe sleepiness was reported in 20.3% and 4.1%, respectively, in early pregnancy and in 14.2% and 2.2%, respectively, in mid‐pregnancy. Severe sleepiness in mid‐pregnancy was associated with lower mean LE8 score (76.9 [SD 13.8] vs. 80.4 [SD 12.9]) 2‐7 years after first birth (aβ ‐3.63 95% CI [‐6.90, ‐0.36]). In contrast, moderate and severe sleepiness in early pregnancy were not associated with LE8 score.

**Conclusion**: Severe sleepiness in mid‐pregnancy may be a previously unidentified risk factor for poorer CVH indices 2‐7 years after first birth.

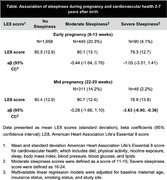

## 596 | Time to Treatment Initiation of Piperacillin‐Tazobactam Compared to Ampicillin‐Gentamicin for the Treatment of Intraamniotic Infection

Rachel P. Gerber; Lizmari Pena‐Perez; Hannah Sturm; Patricia Saunders‐Hao; Matthew J. Blitz; Burton Rochelson; Alpna Aggarwal; Melissa T. Chu Lam

*Northwell, New Hyde Park, NY*

4:00 PM ‐ 6:00 PM

**Objective**: Both the traditional regimen of ampicillin‐gentamicin and the new regimen of piperacillin‐tazobactam, have been proven to successfully treat intraamniotic infection (IAI). We sought to evaluate the time from diagnosis to initiation of antibiotics in the setting of IAI when comparing the recommended traditional regimen of ampicillin (2g IV q6h) and gentamicin (5mg/kg IV q24h), to the alternative regimen of piperacillin‐tazobactam (4.5 g IV q8h).

**Study Design**: Retrospective cohort study of women with suspected IAI as defined by the ACOG at ^3^ 34 weeks of gestation, with singleton gestation, who received either regimen at a university hospital from November 2022 to May 2024. The primary outcome was time to treatment initiation (TTI). This was defined by an analysis of both provider and nursing input into an electronic medical record system, from time of medication order placement to administration initiation.  Mann‐Whitney analysis was used to study the difference in time to treatment.

**Results**: We included 394 patients of which 199 received piperacillin‐tazobactam, and 195 received ampicillin‐gentamicin. Most patients (57.8%) were diagnosed with suspected IAI based on maternal fever and fetal tachycardia. Median TTI was 21 minutes (interquartile‐range 28 minutes) for the piperacillin‐tazobactam group, and 68 minutes (interquartile‐range 51.2) for the ampicillin‐gentamicin group (p‐value < 0.01). There was no significant difference in the rate of postpartum fever, ICU admission, or postpartum readmissions (Table 1). Regarding neonatal outcomes, there were no clinically significant differences in early onset sepsis (EOS) score, rate of NICU admission or cord gas pH (Table 2).

**Conclusion**: Interestingly, the TTI of the complete antibiotic regimen from time of diagnosis is over twice as long with ampicillin‐gentamicin than piperacillin‐tazobactam, likely due to the weight‐based dosing rather than fixed dosing regimen and ease of medication availability for administration to nursing staff.

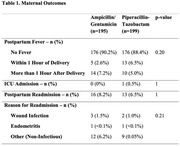

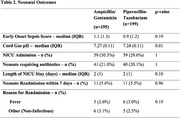

## 597 | A Comparison of Newborn Birthweights Between Opioid Use Disorders Treated with Methadone vs Buprenorphine

Rachel Lee^1^; Cassandra Heiselman^2^; Emily H. Stetler^3^; Zenobia Gonsalves^2^; Kimberly Herrera^3^; David Garry^2^

*^1^Stony Brook University Hospital, Lake Grove, NY; ^2^Stony Brook University Hospital, Stony Brook, NY; ^3^Stony Brook University, Stony Brook, NY*

4:00 PM ‐ 6:00 PM

**Objective**: To determine effects of in‐utero exposure to medications for opioid use disorder (MOUD) on birthweight (BW) and birthweight classification comparing methadone to buprenorphine. 

**Study Design**: This is a retrospective study of patients (N = 362) on MOUD who delivered at a single institution between Jan 2017 and Dec 2023. Patients were divided into comparison groups based on type of MOUD: buprenorphine or methadone. Demographics and clinical outcomes including gestational age at delivery and BW were extracted from medical records. BW was categorized using Fenton 2013 Growth Charts with small for gestational age (SGA) newborns defined as a BW less than 10%ile for gestational age. Univariate and multivariable statistical analyses were done across groups with a statistical significance considered at p < 0.05. 

**Results**: During the study period there were 362 patients, 105 (29%) utilizing methadone and 257 (71%) utilizing buprenorphine. Groups were similar when comparing parity, maternal age, ethnicity, preterm delivery rate, mode of delivery, and concurrent psychiatric diagnosis. BW was greater in the buprenorphine group (3020±615 g) compared to methadone group (2810±586 g; p = 0.003); however, there was a similar incidence of SGA newborns, 14.5% for buprenorphine vs 12.5% for methadone (p = 0.61). Dosing did not affect incidence of SGA infants [Table 1]. In regression modeling, there were no independent predictors of a SGA newborn, although chronic hypertension approached significance (OR 4.30: 95% CI 0.903 to 20.485; p = 0.066) [Table 2]. 

**Conclusion**: When using buprenorphine or methadone for MOUD, reassurance can be provided to patients that the incidence of SGA newborns is similar, even when accounting for medical comorbidities. 

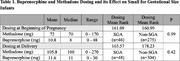

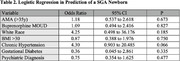

## 598 | Patient Outcomes: Preterm Prelabor Rupture of Membranes Before 34 weeks with Late Preterm Delivery

Rachelle Abdelnour; Christopher Ennen; Noora Batrash

*University of Virginia School of Medicine, Charlottesville, VA*

4:00 PM ‐ 6:00 PM

**Objective**: Preterm prelabor rupture of membranes (PPROM) affects 3% of births in the United States and is a significant cause neonatal morbidity and mortality. Guidelines recommend offering expectant management of delivery when appropriate. The objective of this study was to evaluate neonatal and maternal outcomes by gestational week of delivery for patients with PPROM before 34 weeks and delivery at 34 weeks or greater.

**Study Design**: This was a historical cohort study of patients delivered for PPROM at a single institution between January 2015 and October 2023. The study group included patients who had PPROM with a singleton gestation and non‐anomalous fetus. Maternal and neonatal outcomes were compared by gestational week of delivery.

**Results**: In the cohort, 72 patients had PPROM before 34 weeks with delivery at 34 weeks or greater. Maternal and neonatal outcomes are outlined in the table. Those who delivered at 34 weeks were more likely to have a vaginal delivery after spontaneous preterm labor, compared to those that delivered at 35 weeks, who were more likely to have a vaginal delivery after induction of labor (p = 0.039). Neonates delivered at 35 weeks were less likely to have hyperbilirubinemia (p = 0.019) and less likely to be admitted to the neonatal intensive care unit (p < 0.001) than those delivered at 34 weeks. Outcomes of cesarean delivery, chorioamnionitis, postpartum endometritis, postpartum hemorrhage, 5‐minute APGAR < 7, neonatal hypoglycemia, neonatal sepsis, respiratory distress or tachypnea, and neonatal resuscitation were not significantly different between groups. There were no differences in maternal death, neonatal death, necrotizing enterocolitis, intraventricular hemorrhage, and fetal demise.

**Conclusion**: For those with PPROM before 34 weeks and delivery at 34 weeks or greater, those that delivered at 34 weeks were more likely to have spontaneous preterm labor, neonatal intensive care unit admission, and neonatal hyperbilirubinemia than those delivered at 35 weeks. This data supports counseling patients for expectant management to 35 weeks or more when appropriate.

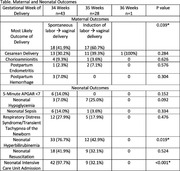

## 599 | Inpatient versus Outpatient Management of Preterm Prelabor Rupture of Membranes: A Systematic Review and Meta‐Analysis

Rafaela Germano Toledo^1^; Nadiha Noor Chelsea^1^; Tina Yi Jin Hsieh^1^; Carol Mita^2^; Anna M. Modest^1^; Cassandra R. Duffy^1^

*^1^Beth Israel Deaconess Medical Center, Boston, MA; ^2^Countway Library, Harvard Medical School, Boston, MA*

4:00 PM ‐ 6:00 PM

**Objective**: The management of preterm prelabor rupture of membranes (PPROM) varies across healthcare settings with limited data on the safety of outpatient management. The aim of this study was to compare neonatal sepsis and other maternal and perinatal outcomes by outpatient vs inpatient management of PPROM.

**Study Design**: We performed a systematic review in October 2023 of Cochrane Central, Embase, PubMed and Web of Science databases for studies comparing outpatient and inpatient management of PPROM. We included studies of pregnancies complicated by PPROM < 37 weeks’ gestational age (GA) published in full text English. Primary outcome was neonatal sepsis; secondary outcomes included neonatal intensive care unit (NICU) admission, NICU length of stay (LOS), intrauterine fetal demise (IUFD), neonatal death, pregnancy latency, chorioamnionitis, and maternal LOS. A random‐effects model was used to calculate pooled odds ratios (OR) or mean differences (MD) and 95% confidence intervals (CI) for all outcomes.

**Results**: Out of the 485 studies identified, 10 were included. These studies comprised a total of 2,286 patients, with 1,040 undergoing outpatient and 1,246 inpatient management. Included studies involved pregnancies from 20 to 35 weeks’ GA. Neonatal sepsis was assessed in all studies, and no significant statistical differences were found between the groups (pooled OR 0.92, 95% CI 0.63‐1.35, Figure 1). The latency period was longer (pooled MD 7.73; 95% CI 0.56‐14.90; Figure 2) and there was less chorioamnionitis (pooled OR 0.54; 95% CI 0.41‐0.72) in the outpatient setting. Regarding neonatal outcomes, the outpatient group had lower odds of NICU admission (pooled OR 0.57; 95% CI 0.36‐0.90) and a shorter NICU LOS (pooled MD ‐9.43 days; 95% CI ‐13.02 to ‐5.84). There were no significant differences in maternal LOS, IUFD, and neonatal death.

**Conclusion**: Outpatient management of PPROM was associated with a longer latency period, lower odds of chorioamnionitis, NICU admission, and shorter NICU length of stay, without an increase in rates of neonatal sepsis.

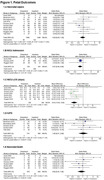

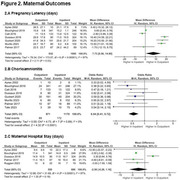

## 600 | Prepregnancy or Delivery BMI? Identifying the Better Predictor of Adverse Outcomes

Raina A. Meka^1^; Nandini Raghuraman^1^; Alison G. Cahill^2^; Jeannie C. Kelly^1^; Katherine H. Bligard^1^; Antonina I. Frolova^3^

*^1^Washington University School of Medicine in St. Louis, St. Louis, MO; ^2^Dell Medical School Health Transformation Research Institute, Dell Medical School, The University of Texas at Austin, Austin, TX; ^3^Washington University School of Medicine, St. Louis, MO*

4:00 PM ‐ 6:00 PM

**Objective**: Prepregnancy BMI (preBMI) represents baseline health status before pregnancy and is used to guide counseling and management to prevent adverse perinatal outcomes. However, preBMI is often unavailable or unreliable due to recollection bias. Delivery BMI (delBMI) encompasses the cumulative effects of preBMI and gestational weight gain (GWG). We sought to compare the ability of preBMI and delBMI to predict adverse maternal and neonatal outcomes.

**Study Design**: This was a secondary analysis of a prospective cohort study of all individuals with a singleton gestation who presented for induction or spontaneous labor at ≥ 37 weeks’ gestation at a single institution from 2010‐2014. Only individuals with a measured weight in the chart during the year prior to pregnancy were included in this analysis, allowing for accurate calculation of preBMI. Receiver operating characteristic (ROC) curves were used to compare the abilities of preBMI and delBMI to predict labor, maternal, and neonatal adverse outcomes.

**Results**: Among 8,580 patients in the original cohort, 2,964 had a prepregnancy weight recorded and were included in this study. In isolation, delBMI and preBMI were poor predictors of adverse outcomes. However, delBMI was a better predictor than preBMI of labor outcomes including primary cesarean section (Figure 1), labor > 24 hours, prolonged second stage of labor, and shoulder dystocia (Table 1). DelBMI was also a better predictor of hypertensive disorders of pregnancy (HDP), while preBMI was a better predictor of gestational diabetes (GDM) (Table 1). There was no difference in the ability of delBMI and preBMI to predict neonatal outcomes, including composite adverse neonatal outcome, fetal acidemia, NICU admission, and neonatal hypoglycemia (Table 1).

**Conclusion**: PreBMI and delBMI are individually poor predictors of adverse pregnancy outcomes. However, delBMI is a better predictor of adverse labor outcomes and HDP, while preBMI is a better predictor for GDM. Comprehensive risk assessment may need to reflect maternal weight throughout pregnancy, incorporating preBMI, delBMI and GWG as a dynamic measure.

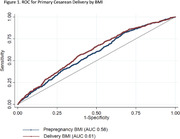

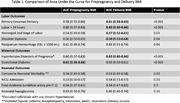

## 601 | Confirmation of the Physiological Principles of the Sequential Laser Technique for Twin‐Twin Transfusion Syndrome

Tucker E. Doiron^1^; Lisa M. Korst^2^; Arlyn Llanes^3^; Martha A. Monson^4^; Andrew H. Chon^5^; Ramen H. Chmait^3^

*^1^Saint Louis University, St. Louis, MO; ^2^Childbirth Research Associates, North Hollywood, CA; ^3^Keck School of Medicine, University of Southern California, Los Angeles, CA; ^4^Intermountain Healthcare, University of Utah Health, Salt Lake City, UT; ^5^Oregon Health & Science University, Portland, OR*

4:00 PM ‐ 6:00 PM

**Objective**: To assess changes in middle cerebral artery peak systolic velocity (MCA‐PSV) after sequential vs selective laser surgery for twin‐twin transfusion syndrome (TTTS).

**Study Design**: This is a post hoc analysis of data from the Sequential Trial, a randomized controlled trial (RCT) that compared laser techniques for TTTS patients between 18‐26 weeks of gestation. Patients received either the original (selective) or a modified selective (sequential) laser surgery, in which arteriovenous (AV) anastomoses with unidirectional blood flow from donor to recipient are laser‐ablated first, followed by ablation of AV anastomoses from recipient to donor that should allow for a net intraoperative blood transfer from the hypervolemic recipient to the hypovolemic donor twin. We compared the difference (Delta) in donor MCA‐PSV multiples of the median (MoM) (postoperative minus preoperative) by surgical technique. Because anemia is associated with a higher MCA‐PSV, a negative Delta is consistent with an increased blood volume.

**Results**: In the 606 study patients, the mean (SD) Delta MCA‐PSV MoM for the donor twin was ‐0.067 (0.301) (P< .0001) for the sequential and ‐0.017 (0.288) (P = .4186) for the selective techniques, with P< .0001 for the technique comparison. Based on donor critical abnormal Dopplers (CAD) and arterioarterial (AA) anastomoses, 4 risk Categories with donor survival rates were established (Figure). Mean (SD) Deltas for the sequential vs selective procedures are shown by Category in the Table. Linear regression for Delta, controlling for the risk groups, yielded a coefficient for the sequential vs selective techniques of ‐0.079 (0.024 SE), P = .0009.

**Conclusion**: Overall, patients receiving the sequential vs the selective technique had a greater drop in the mean donor MCA‐PSV MoM, suggesting that the sequential technique facilitated an intraoperative net blood transfusion to the donor. This benefit was apparent in all risk groups except Category 4, which had poor donor survival. Further study is needed to understand and optimize outcomes of Category 4 patients.

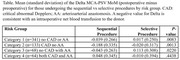

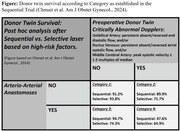

## 602 | Twin‐Twin Transfusion Syndrome with Intermittent (Stage I) versus Persistent (Stage III) Umbilical Artery Doppler Abnormalities

Raphael C. Sun^1^; Andrew H. Chon^2^; Lisa M. Korst^3^; Arlyn Llanes^4^; Martha A. Monson^5^; Ramen H. Chmait^4^

*^1^Department of Surgery, Division of Pediatric Surgery, Department of Obstetrics and Gynecology, Oregon Health & Science University, Portland, OR; ^2^Oregon Health & Science University, Portland, OR; ^3^Childbirth Research Associates, North Hollywood, CA; ^4^Keck School of Medicine, University of Southern California, Los Angeles, CA; ^5^Intermountain Healthcare, University of Utah Health, Salt Lake City, UT*

4:00 PM ‐ 6:00 PM

**Objective**: Per Quintero staging for twin‐twin transfusion syndrome (TTTS), umbilical artery (UA) Doppler with *intermittent* absent or reversed end diastolic flow (A/REDF) is classified as Stage I, and UA with *persistent* A/REDF is classified as Stage III. We compared laser outcomes in Stage I patients with UA normal vs Stage I with *intermittent* A/REDF vs Stage III with *persistent* A/REDF.

**Study Design**: Cases of monochorionic diamniotic twins with TTTS Quintero stages I and III treated with laser (2006‐2023) were reviewed and 3 groups analyzed: Stage I with no Doppler abnormalities (Stage‐I‐normal), Stage I with donor *intermittent* A/REDF of the UA (Stage‐I‐intermittent), and Stage III with donor *persistent* A/REDF of the UA (Stage‐III). The outcome was donor twin intrauterine fetal demise (IUFD).  

**Results**: Of 349 study patients, 101 were classified as Stage‐I‐normal, 36 were Stage‐I‐intermittent, and 212 were Stage III. Arterio‐arterial (AA) communications were most common in Stage‐I‐intermittent: 27.7% vs 69.4% vs 42.5%, *P*< .0001. The rate of donor IUFD was lowest in Stage‐I‐normal compared to Stage‐I‐intermittent and Stage‐III: 3.0% vs 25.0% vs 29.2%, *P*< .0001. At‐least‐one survivorship did not differ among the 3 groups (Table 1); dual survivorship was highest for Stage‐1‐normal and differed from both Stage‐I‐intermittent and Stage‐III in pairwise comparisons. In a multivariable logistic regression model, Stage‐I‐intermittent patients were 7 times more likely to have donor twin IUFD (OR 7.23 [1.69‐30.82], *P* = .0075), as were Stage‐III patients (7.24 [2.06‐25.47], *P* = .0020), compared to Stage‐I‐normal patients. (Table 2) In this same model, patients with AA were 3.6 times more likely to have donor IUFD (OR 3.59 [1.95‐6.61]; *P*< .0001).

**Conclusion**: TTTS stage I patients with *intermittent* A/REDF UA findings were at increased risk of donor IUFD after laser surgery. It is unknown whether laser surgery or expectant management is the optimal approach for this subset of patients. The role of AA anastomoses in TTTS needs further investigation.

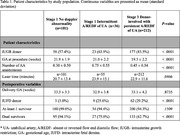

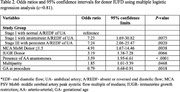

## 603 | Group B Streptococci Colonization and the Expectant Management of Late Preterm Prelabor Rupture of Membranes

Raneen Abu Shqara^1^; Lior Lowenstein^2^; Maya Frank Wolf^3^

*^1^Galilee Medical Center, Nahariya, HaZafon; ^2^Galilee Medical center, Nahariya, HaZafon; ^3^Galilee Medical Center, Naharyia, HaZafon*

4:00 PM ‐ 6:00 PM

**Objective**: The impact of maternal group B streptococci (GBS) colonization on outcomes of expectant management of late premature prelabor rupture of membranes (PPROM) is controversial. We explored maternal and neonatal infectious outcomes in women who presented with PPROM at 34‐36.6 weeks according to GBS colonization status.

**Study Design**: This retrospective cohort study included 412 women who presented with late PPROM and were admitted for expectant management. Outcomes were compared according to positive GBS status (n = 73) and negative GBS status (n = 339). The co‐primary outcomes were clinical chorioamnionitis and latency duration (ROM‐delivery duration). Expectant management consisted of betamethasone administration and 7‐day antibiotics regimen. Induction was performed at 37 weeks or earlier if chorioamnionitis or non‐reassuring fetal status was suspected. After delivery, chorioamniotic swabs were obtained. The required sample size was calculated to be at least 398, considering a 10% effect size in the rate of women with latency > 48 hours,  with a power of 80% and an alpha of 5%.

**Results**: Gestational age at PPROM was similar between the groups (Table). The rates of intrapartum fever, clinical chorioamnionitis, postpartum fever, latency duration, latency >48 hours, and delivery week were similar. For the positive vs. negative GBS status group, antibiotic administration in the neonatal intensive care unit was more common: 11.1% vs 3.5%, p = 0.013. Rates of neonatal complications such as early onset sepsis, respiratory distress syndrome, invasive ventilation, and meconium aspiration syndrome were similar between the groups. The distributions of pathogens in chorioamniotic swab cultures were similar (p = 0.461) (Figure).

**Conclusion**: Among women with PPROM at 34‐36.6 weeks, we report similar maternal and neonatal adverse outcomes. The increased neonatal antibiotic administration may be attributed to preventive measures related to the positive GBS status. Expectant management of PPROM in women with GBS colonization is reasonable.

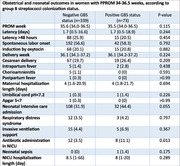

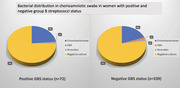

## 604 | Comparing Two Prophylactic Antibiotic Protocols for Treating Preterm Pre‐Labor Rupture of Membranes

Raneen Abu Shqara^1^; Gabriela Goldinfeld^2^; Lior Lowenstein^3^; Maya Frank Wolf^4^

*^1^Galilee Medical Center, Nahariya, HaZafon; ^2^Bar‐Ilan university, Bar‐Ilan University, HaZafon; ^3^Galilee Medical center, Nahariya, HaZafon; ^4^Galilee Medical Center, Naharyia, HaZafon*

4:00 PM ‐ 6:00 PM

**Objective**: Following our randomized trial, we changed the prophylactic antibiotic protocol for preterm pre‐labor rupture of membranes (PPROM) in our department from ampicillin‐roxithromycin to cefuroxime‐azithromycin. We compared the infectious morbidity of these two regimens.

**Study Design**: This retrospective study included 170 women with PPROM < 37 weeks, managed expectantly with betamethasone and prophylactic antibiotics. According to protocol, chorioamniotic membrane cultures were obtained after delivery. Maternal and neonatal outcomes were compared between 83 women who received ampicillin‐roxithromycin (February 2022 ‐ March 2023) and 87 who received cefuroxime‐azithromycin (April 2023 ‐ May 2024). A composite maternal postpartum infectious outcome consisted of endometritis, antibiotic treatment and surgical site infection. A composite neonatal adverse outcome consisted of early onset sepsis, admission to the neonatal intensive care unit, antibiotic treatment, sepsis workup and respiratory distress syndrome. 

**Results**: Gestational age at PPROM, parity, group B Streptococcus status and the median latency duration were comparable in the two periods. Among women treated with ampicillin‐roxithromycin vs. cefuroxime‐azithromycin, a greater proportion had intrapartum fever, clinical chorioamnionitis, cesarean delivery and a postpartum infectious outcome: 9.6% vs 1.1%, p = 0.013; 8.4% vs 1.1%, p = 0.046; 33.7% vs 19.5%, p = 0.036; and 4.8% vs. 0%, p = 0.038, respectively (Table). The composite neonatal adverse outcome did not differ between the groups (p = 0.874). Following the ampicillin‐roxithromycin vs. cefuroxime‐azithromycin regimen, prevalences were greater of positive chorioamniotic membrane cultures (50.0% vs. 32%, p< 0.001) (Figure) and of ampicillin‐resistant Enterobacteriaceae spp. (23% vs. 10%, p = 0.027).

**Conclusion**: Cefuroxime‐azithromycin compared to ampicillin‐roxithromycin as a prophylactic for PPROM < 37 weeks showed less maternal peripartum infections, which was supported by the bacteriologic findings.

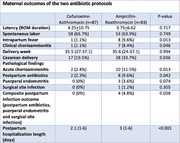

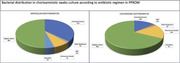

## 605 | Ehr‐Integration of an Evidence‐Based Calculator for Cesarean Risk During Induction: Impact on Utilization and Outcomes

Rebecca F. Hamm^1^; Mary C. Steele^1^; Allison Dreier^2^; Sameh Saleh^3^; Mika Epps^1^; Madeline Fagen^1^; Mucio K. Delgado^1^; Lisa D. Levine^1^

*^1^University of Pennsylvania Perelman School of Medicine, Philadelphia, PA; ^2^Sidney Kimmel Medical College at Thomas Jefferson University, Philadelphia, PA; ^3^Inova Health System, Fairfax, VA*

4:00 PM ‐ 6:00 PM

**Objective**: We developed a validated calculator to predict individualized likelihood of cesarean delivery (CD) for patients undergoing induction (IOL) [Levine 2018]. In a prospective cohort, calculator implementation via an external website was associated with reduced maternal morbidity and CD [Hamm 2020].  Here, we aimed to determine if Electronic Health Record (EHR) integration of the CD risk calculator would further improve utilization and clinical outcomes.

**Study Design**: This prospective cohort evaluated calculator utilization, maternal morbidity, and CD for 6 months PRE (10/2022‐4/2023) vs. 6 months POST EHR‐release of the CD risk calculator (4/2023‐10/2023) in 2 units. Prior to EHR‐integration, Site#1 had a 2018 push for calculator website use, while Site#2 had not. EHR‐integration recognized eligibility, allowed clinicians to confirm and utilize EHR variables for score automation, visible on electronic display. Patients were calculator eligible if undergoing term IOL with a singleton, intact membranes, and dilation ≤2cm without prior CD. Calculator utilization was measured as a score recorded in the EHR. Analysis was stratified by site and risk of CD based on calculator results (< 20%, 20‐39.9%, 40‐59.9%, ≥60%).

**Results**: 2018 patients met eligibility (PRE:998; POST:1010) with more IOLs for fetal indications, earlier gestational age, and higher rates of hypertension in the POST group. Calculator utilization significantly increased POST‐EHR implementation (Table). There were no differences PRE to POST in maternal morbidity overall (aRR 1.18 [0.91‐1.53]), by site or CD risk strata. There was no difference in CD overall (aRR 1.19 [0.99‐1.43). However, an increase in CD was found at Site#2 POST EHR‐integration (aRR 1.30 [1.02‐1.66]), specifically in the ≥60% CD risk strata.

**Conclusion**: EHR‐integration of a CD risk calculator was associated with substantial increases in utilization. While CD increased at one site PRE to POST, PRE rates were lower than anticipated, and POST rates consistent with institutional data. Care should be taken this calculator is not used to perform CD outside of clinical indications.

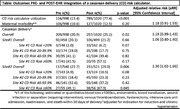

## 606 | Implementation of a Standardized Protocol for Labor Induction: Impact on Obstetric Disparities

Rebecca F. Hamm^1^; Sunni Mumford^1^; Markolline Forkpa^2^; Nicholas J. Seewald^1^; Stefanie N. Hinkle^1^; Rinad S. Beidas^3^; Sindhu K. Srinivas^1^; Samuel Parry^4^; Lisa D. Levine^1^

*^1^University of Pennsylvania Perelman School of Medicine, Philadelphia, PA; ^2^Pennsylvania Medicine Women's Health Clinical Research Center, Philadelphia, PA; ^3^Feinberg School of Medicine, Northwestern University, Chicago, IL; ^4^University of Pennsylvania, Philadelphia, PA*

4:00 PM ‐ 6:00 PM

**Objective**: Prior retrospective data demonstrated that standardization of labor induction (IOL) may reduce racial disparities in cesarean delivery (CD) and morbidity. Here, we aimed to determine the impact of prospectively implementing an IOL protocol on racially disparate outcomes.

**Study Design**: This was a prespecified secondary analysis of a type I hybrid effectiveness‐implementation trial comparing 2 years before (PRE) and 2 years after (POST) implementation of a standardized IOL protocol at 2 sites (2018‐2022). The protocol had 8 components and recommended active IOL management, frequent cervical exams, and amniotomy by first exam ≥4cm. All singletons ≥37 weeks with intact membranes requiring cervical ripening were eligible; prior CD was excluded. This analysis included only those with self‐recorded race, divided into Black, Indigenous, People of Color (BIPOC) and non‐BIPOC. Poisson regression with interaction terms evaluated the protocol's impact on disparities in CD and morbidity. Fidelity to the protocol was defined as adherence to ≥75% of the 8 protocol components.

**Results**: 8386 were included (PRE = 4167; POST = 4219); 59.3% identified as BIPOC. BIPOC patients differed in delivery site, insurance, BMI, parity, maternal age, diagnosis of diabetes and hypertension, gestational age, and IOL indication, included as confounders.  BIPOC patients were more likely to undergo CD in both PRE (aRR 1.36[1.18‐1.58]) and POST (aRR 1.55[1.33‐1.70]) groups, even when controlling for these differences. Adjusted relative risk of maternal morbidity was greater among BIPOC patients both PRE (aRR 1.25[1.07‐1.46]) and POST (aRR 1.34[1.14‐1.58]). There was no difference by race in neonatal morbidity in either PRE or POST. There was no differential effect of the protocol by race on CD or morbidity (Table). Protocol fidelity increased PRE to POST for both BIPOC (58.4% to 64.3%, p< 0.001) and non‐BIPOC patients (43.6% to 53.1%, p< 0.001; interaction p = 0.15).

**Conclusion**: Despite increased utilization of a standardized IOL protocol across races, this intervention did not mitigate observed racial disparities in CD or maternal morbidity.

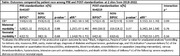

## 607 | Impact of Health Disparities in Patients with Twin‐to‐Twin Transfusion Syndrome

Rebecca M. Johnson^1^; Luis E. Delgadillo Chabolla^2^; David G. Mann^2^; Cara Buskmiller^3^; Roopali V. Donepudi^3^; Ahmed A. Nassr^3^; Magdalena Sanz Cortes^3^; Michael A. Belfort^3^; Jessian L. Munoz^1^

*^1^Baylor College of Medicine, Houston, TX; ^2^Baylor College of Medicine/Texas Children's Hospital, Houston, TX; ^3^Texas Children's Hospital and Baylor College of Medicine, Houston, TX*

4:00 PM ‐ 6:00 PM

**Objective**: Social determinants of health impact access to care for patients with complex pregnancies. This study aimed to determine whether patients diagnosed with twin‐to‐twin transfusion syndrome presented with more progressive disease as measured by Quintero stage (I‐IV) at presentation for evaluation for intervention based on their type of insurance coverage.

**Study Design**: This retrospective cohort study included monochorionic‐diamniotic pregnant patients diagnosed with twin‐to‐twin transfusion syndrome who underwent laser photocoagulation at our fetal center between 11/2011‐5/2024. Patients were categorized based on their type of insurance coverage, defined as commercial/private insurance (Private) or The Children's Health Insurance Program/Medicaid/No Insurance (Public). The primary outcome was the Quintero stage (I‐IV) at the initial consultation for surgery. Secondary outcomes included gestational age at intervention, selective fetal growth restriction (sFGR), latency from referral to evaluation, location of evaluation, gestational age at delivery, and survival at 30 days. Patients without 30‐day outcomes available were excluded.

**Results**: 484 patients underwent laser photocoagulation for TTTS at our center. Patients with public insurance were more likely to present with a higher Quintero stage than those with private insurance (3 [2,3], 3 [2,3], p = 0.046). The public insurance group was also more likely to be White/Hispanic (45.7% v 19.5%, p< 0.001) or Black/Non‐Hispanic (15.7% v 6.6%, p< 0.001), be evaluated as an inpatient (69% v 55%, p = 0.002), have sFGR (47% v 36%, p = 0.014) and have a higher gestational age at intervention (20.7[18.6,22.4] v 19.9[17.9,22.0], p = 0.015). Latency from referral to evaluation and GA at delivery were similar, and we found no difference in survival at 30 days between groups.

**Conclusion**: Our study shows that social disparities exist in this population, and insurance status may be a surrogate of these inequities. However, long‐term patient outcomes are equivalent with prompt, comprehensive care, aligning with the principle of distributive justice and the fair opportunity rule.

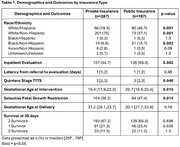

## 608 | Continuous Glucose Monitor Time in Range: Maternal Outcomes Amongst Pregnant Patients with T2Dm & Gdm

Yara Hage Diab^1^; Lauren A. Forbes^2^; Erkan Kalafat^3^; Theresa Corbine^1^; Margaret Mlynarczyk^1^; Marwan Ma'Ayeh^1^; George R. Saade^1^; Rebecca Horgan^1^

*^1^Macon & Joan Brock Virginia Health Sciences Eastern Virginia Medical School at Old Dominion University, Norfolk, VA; ^2^Eastern Virginia Medical School, Norfolk, VA; ^3^Koc University Hospital, Istanbul, Istanbul*

4:00 PM ‐ 6:00 PM

**Objective**: The Advanced Technologies & Treatments for Diabetes consensus group recommends target time in range (TIR) with a continuous glucose monitor (CGM) of 70% for pregnant patients with type 1 diabetes but concluded that there is inadequate evidence to recommend target TIR for type 2 diabetes (T2DM) or gestational diabetes (GDM). Our objective was to determine the impact of TIR categories on maternal outcomes amongst pregnant patients with T2DM and GDM.

**Study Design**: This retrospective cohort study included pregnant patients with T2DM or GDM using CGM for at least 30 days at our tertiary center from January 2020 to December 2022. Exclusion criteria were type 1 diabetes, multiple gestation, and unavailable delivery records. Target range for glucose on CGM was 63‐140 mg/dL. Mean TIR for duration of CGM use during pregnancy was recorded. Primary outcome was maternal adverse events defined as having a cesarean delivery, postpartum hemorrhage or developing a hypertensive disorder of pregnancy. Maternal outcome in TIR groups were compared with Fisher's Exact test and logistic regression.

**Results**: 71 patients were included, of whom 48 and 23 had a mean TIR ≥70% and < 70%, respectively. Patients with mean TIR ≥70% had lower rates of cesarean delivery (56.2% vs. 78.3%, P = 0.12), postpartum hemorrhage (14.6% vs. 21.7%, P = 0.68) and hypertensive disorders of pregnancy (35.4% vs. 47.8%, P = 0.46) but the differences did not reach statistical significance (Figure 1). Patients with mean TIR ≥70% had a reduced odds of composite maternal outcome after adjusting for maternal BMI and diabetes type (adjusted OR: 0.40, 95% CI: 0.10‐1.37, P = 0.166), but again the difference did not reach statistical significance.

**Conclusion**: All indicators trend towards better outcomes in pregnant patients with T2DM or GDM with mean TIR ≥70% on CGM compared with those with mean TIR < 70%. However, a larger sample size may be needed to statistically confirm this difference.

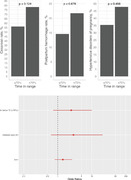

## 609 | Cgm Initial Time in Range & Trajectory: Impact on Neonatal Outcomes in T2Dm and Gdm

Rebecca Horgan^1^; Yara Hage Diab^1^; Lauren A. Forbes^2^; Erkan Kalafat^3^; Theresa Corbine^1^; Margaret Mlynarczyk^1^; George R. Saade^1^; Marwan Ma'Ayeh^1^

*^1^Macon & Joan Brock Virginia Health Sciences Eastern Virginia Medical School at Old Dominion University, Norfolk, VA; ^2^Eastern Virginia Medical School, Norfolk, VA; ^3^Koc University Hospital, Istanbul, Istanbul*

4:00 PM ‐ 6:00 PM

**Objective**: Continuous glucose monitor (CGM) is increasingly utilized to manage pregnant patients with type 2 diabetes (T2DM) and gestational diabetes (GDM), with target time in range (TIR) the main measure of glucose control. We sought to evaluate the independent contributions of initial TIR versus TIR trajectory during pregnancy on neonatal outcomes.

**Study Design**: This retrospective cohort study included pregnant patients with T2DM or GDM using CGM for ⩾ 30 days from Jan 2020 to Dec 2022. Exclusion criteria were type 1 diabetes, multiple gestation, and unavailable delivery records. Target glucose values on CGM were defined as 63‐140 mg/dL. Initial TIR was defined as mean TIR after 2 weeks of CGM use. The primary composite neonatal outcome was macrosomia, birth trauma, significant hyperbilirubinemia, stillbirth, or neonatal death. TIR values were modeled with mixed effect generalized additive models. TIR groups were formed using sample quartiles (Initial TIR: good, borderline, abnormal; TIR trajectory: worsened, stable and improved).

**Results**: 71 patients were included in the analysis. Initial TIR was abnormal in 17, borderline in 30, and good in 18, with the primary outcome occurring in 35%, 30%, and 17%, respectively. TIR trajectory worsened in 18, remained stable in 35, and improved in 12, with 28%, 29%, and 25%, respectively having the primary outcome. When initial TIR and trajectory were considered together, the primary outcome rates in patients with abnormal initial TIR were 25%, 50%, and 100% for improved, stable, and worsened TIR trajectory. For borderline initial TIR, rates were 0%, 30.4%, and 28.6%, respectively. For good initial TIR, rates were 12.5% and 20% for stable and worsened TIR. Initial TIR (aOR 0.26, 95% CI: 0.09‐0.61) and TIR trajectories (aOR 0.45, 95% CI: 0.18‐1.01) were independently associated with adverse outcomes after adjusting for diabetes type.

**Conclusion**: Pregnant patients with T2DM and GDM should be counseled that poor initial TIR values on CGM is associated with increased risk of adverse neonatal outcomes, and improvement in TIR during pregnancy reduces this risk significantly.

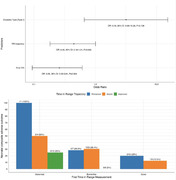

## 610 | Machine Learning Prediction of NICU Admission at the Point of Care

Reetam Ganguli^1^; Stephen Wagner^2^

*^1^Elythea, San Jose, CA; ^2^Beth Israel Deaconess Medical Center, Boston, MA*

4:00 PM ‐ 6:00 PM

**Objective**: NICU admissions significantly raise neonatal mortality risk and cost up to $168,000. No universal assessments predict NICU admission risk at the point of care. Literature shows early prenatal risk assessment and education/nutrition intervention reduced NICU admissions by 56% and NICU days by 59%. This study aims to develop an ML model predicting NICU admissions using early prenatal variables and demographic/clinical history.

**Study Design**: ML models were trained on sociodemographic, medical history, and obstetric variables obtainable at the point of care. Models were trained on years 2018‐2020 of the CDC Vital Statistics System and were tested on year 2021. NICU patients from years 2014‐2017 (with the exception of year 2015 due to an unavailable data file) were included in training to reduce class imbalance.

Exclusion criteria: missing NICU data and non‐reporting hospitals. The primary outcome was immediate NICU admission post‐delivery. Weight scaling algorithms enhanced training for preterm labor patients and NICU patients from prior years up to 2014 were used to improve class imbalance.

**Results**: 44 clinical variables obtainable in the 1st trimester across 12,127,124 patients were included in the training data. 2,041,555 (16.83%) experienced NICU admissions.  

The model was tested on 3,219,217 patients, of which 351,787 (10.93%) had a NICU admission. The developed model had an area under the receiver operating characteristic curve of 0.76, 77% accuracy, sensitivity of 0.60, and F1 score of 0.77 at a standard 50% threshold.

When Youden's J statistic was calculated, the optimal threshold was 45.55%, which yielded 0.66 sensitivity.

**Conclusion**: Machine learning models can identify nearly 2/3rds of all NICU admissions at the point of care using routinely collected factors. This early identification can potentially allow for targeted interventions to prevent over half of all NICU admissions and lead to cost savings for health plans.

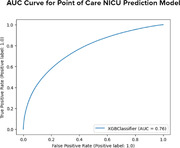

## 611 | Predicting Severe Hemorrhage in Operative Cesarean Deliveries Using Machine Learning

Reetam Ganguli^1^; Julia Sroda Agudogo^2^; Stephen Wagner^2^

*^1^Elythea, San Jose, CA; ^2^Beth Israel Deaconess Medical Center, Boston, MA*

4:00 PM ‐ 6:00 PM

**Objective**: Maternal hemorrhage necessitating transfusion is a significant complication in cesarean deliveries. This study aims to develop machine learning (ML) classifiers to predict hemorrhage necessitating transfusion in mothers undergoing cesarean delivery.

**Study Design**: AdaBoost, LightGBM, XGBoost, RandomForest, and Logistic Regression mdoels were developed. The models were trained and tested on data from the American College of Surgeons National Surgical Quality Improvement Program database from years 2009 to 2021. Included patients underwent any cesarean delivery identified by specific Current Procedural Terminology (CPT) codes: 59510, 59514, 59515, 59618, 59620, and 59622 with complete data for the outcome. Cases with missing data on the primary outcome were excluded. Training data included preoperative blood biomarkers, clinical history, and sociodemographic information. All models were evaluated using 5‐fold cross‐validation to ensure robustness. The training data included preoperative clinical and sociodemographic variables for a large cohort of obstetric patients. The primary outcome was severe hemorrhage requiring transfusion.

**Results**: Of the total 43,713 patients, 1,425 (3.3%) experienced the outcome of maternal transfusion. AdaBoost and RandomForest models demonstrated the highest accuracy and AUC ROC scores among the evaluated models. Specifically, the AdaBoost model achieved an AUC ROC of 0.72 (95% CI: 0.72‐0.72) and an accuracy of 96.52% (95% CI: 96.34%‐96.71%). The RandomForest model showed similar performance with an AUC ROC of 0.71 (95% CI: 0.71‐0.71) and an accuracy of 96.59% (95% CI: 96.45%‐96.73%).

**Conclusion**: AdaBoost and RandomForest models show the highest potential for predicting severe hemorrhage requiring transfusion in operative cesarean deliveries. These models can assist in early identification and intervention, potentially improving patient outcomes.

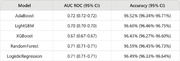

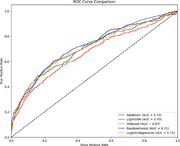

## 612 | Cost‐Effectiveness of Manual Rotation in the Setting of Persistent Occiput Posterior Position

Riane Bradbury‐Huang^1^; Aaron B. Caughey^2^

*^1^Barnard College of Columbia University, Portland, OR; ^2^Oregon Health & Science University, Portland, OR*

4:00 PM ‐ 6:00 PM

**Objective**: Persistent occiput posterior position during the second stage of labor occurs in approximately 5% of annual pregnancies. This fetal position is associated with longer labors, higher cesarean rates, and increased perinatal complications. Manual rotation of the fetal occiput can resolve the malposition in a subset of pregnancies. We examined the outcomes and costs of manual rotation versus expectant management in the setting of persistent occiput posterior position in the second stage of labor.

**Study Design**: We developed a decision‐analytic model using TreeAge to compare outcomes between manual rotation versus expectant management for a theoretical cohort of 70,400 individuals, the annual number of nulliparous patients with persistent fetal OP position. Outcomes included cesarean delivery, post‐op infection, instrumental delivery, severe perineal laceration, postpartum hemorrhage, and maternal death in addition to costs and quality‐adjusted life years (QALYs). The willingness‐to‐pay (WTP) threshold was set at $100,000/QALY. We conducted literature searches to find inputs for the model, and conducted sensitivity analyses to discover and vary the drivers of the model in order to study its strengths.

**Results**: Within the theoretical cohort of 70,400 pregnancies, manual rotation prevented 24,119 cesarean sections, 1,206 post‐op infections, 7,040 instances of postpartum hemorrhage, and 3 maternal deaths. However, it also resulted in an increased 10,353 instrumental deliveries and 4,796 severe perineal lacerations due to the reduction of cesareans. The manual rotation strategy was dominant to expectant management, resulting in more QALYs for lower costs. Sensitivity analyses demonstrated that manual rotation remained cost‐effective until the cost of vaginal delivery was above $23,037.

**Conclusion**: We found manual rotation led to better outcomes and lower costs for nulliparous patients with persistent fetal OP position compared to expectant management. Further study of the impact of manual rotation and how it can be more widely trained and adopted is important for future research.

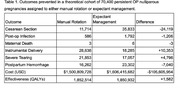

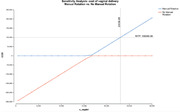

## 613 | Cerebrospinal Fluid Analysis in Fetuses with Myelomeningocele

Romain Corroenne^1^; Enrico R. Barrozo^2^; Roopali V. Donepudi^3^; Ahmed A. Nassr^3^; Brian Burnett^1^; Rebecca M. Johnson^1^; William E Whitehead^2^; Michael A. Belfort^3^; Magdalena Sanz Cortes^3^

*^1^Baylor College of Medicine, Houston, TX; ^2^Baylor College of Medicine and Texas Children's Hospital, Houston, TX; ^3^Texas Children's Hospital and Baylor College of Medicine, Houston, TX*

4:00 PM ‐ 6:00 PM

**Objective**: Fetuses with myelomeningocele (MMC) often present with ventriculomegaly (VMG). If severe, the pressure exerted on the brain parenchyma may potentially cause axonal demyelination and lead to neurological deficits. Previously, using flow cytometry, we observed heterogeneity in cellular populations in fetal cerebrospinal fluid (CSF) obtained immediately prior to beginning fetal surgery. We hypothesized that differences in proportional microglia and neuronal progenitor cells in fetal CSF correlate with neurological deficits observed in children with MMC and may reflect the degree of brain tissue injury.

**Study Design**: Retrospective study of CSF from 9 fetuses who underwent a laparotomy‐assisted fetoscopic MMC repair. Before surgery, fetal MRI and ultrasound scans were performed. Ventricular atrial width measurements, anatomical level of the lesion, and presence/absence of clubfeet were recorded. At birth, motor function was evaluated. Fetal CSF was collected from the MMC sac immediately prior to starting the fetoscopic MMC repair and subjected to single‐cell RNA‐sequencing (scRNA‐seq; n = 9, 25.1[23.7‐25.9] weeks gestation) to quantify proportional cell counts. Proportions of cellular populations were compared between cases with different neurological characteristics and outcomes.  

**Results**: A total of 18 distinct CSF cellular subpopulations were identified (Figures 1A, 1B). Significantly fewer macrophages were found in the CSF from fetuses with VMG compared to those with no VMG (Figure 1, C). Cases with an anatomical lesion higher than L2 had fewer immature neurons as compared to those where the lesion was < L2 (Figure 1, D). When club foot was present at the time of the surgery there were more trophoblast‐like cells than in those without club foot. Infants with impaired MF at birth, and those with club foot, showed significantly less proportional microglia cells than their counterparts (Figure 2).

**Conclusion**: In fetuses with MMC there are changes in the CSF cellular footprint that can be observed at midgestation and which may be associated with specific neurologic findings present at birth.

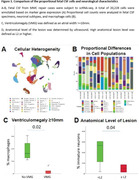

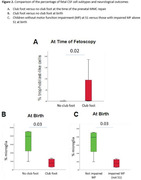

## 614 | Predicting Severe Blood Loss Before Cesarean Hysterectomy (CH): A Novel Method Focusing on Bladder Vascularity

Rosa Drummond; Mevlut Bucak; MaryEllen Mangione; Ozhan M. Turan

*University of Maryland Medical Center, Baltimore, MD*

4:00 PM ‐ 6:00 PM

**Objective**: Placenta accreta spectrum (PAS) grade 3b&c pose the highest risk for severe blood loss (SBL) during CH, primarily due to bladder dissection. Interventions like interval hysterectomy, conservative management and aortic occlusion (REBOA) are proposed to reduce SBL but come with complications. Current preoperative assessments are unreliable in identifying SBL cases. We hypothesize that cystoscopic (C) evaluation can accurately predict SBL.

**Study Design**: PAS cases had preoperative ultrasound, MRI, and postoperative pathological grading. Standardized cystoscopy and bilateral stents were used in non‐urgent cases. Quantitative blood loss (QBL), surgical complications, and transfusions were recorded. Same surgeon, standardized techniques, and blinded evaluation of C recordings were inclusion criteria (Figure 1). Odds ratio (OR) for cystoscopy variables were calculated using postoperative grading. The Cystoscopy Index (CI) was calculated from OR sums. Postoperative grades 3b&c were high grade (HG); 1, 2, and 3a were low grade (LG). Primary outcome was CI prediction of SBL > 2000ml. Secondary outcomes included surgical complications and REBOA placement. C results were analyzed using descriptive statistics, correlation, and ROC curve.

**Results**: Of 66 cases, 31 were HG and 35 LG. Postoperative grading identified more HG cases than preoperative (p = 0.002), with grade 3b being the most common (38%). Bridging vessels (BV) and distortion of the trigone (DT) were the strongest predictors of HG (odds ratios 3.5 and 5.1, p = 0.01). CI correlated with QBL (p = 0.036). ROC analysis calculated CI ≥ 2.8 is the predictor for HG cases (AUC:0.68, p = 0.009). CI ≥ 2.8 was identified in 82% (14/17) of cases with SBL and required >4U RBC transfusion (p = 0.01 and 0.03) (Table 1). DT was significantly related to cystotomy (p = 0.007). Two HG cases required REBOA and had CI ≥ 2.8.

**Conclusion**: Preoperative cystoscopy and CI can identify cases resulting in SBL, unavoidable cystotomy, and REBOA. High CI can guide discussions about alternatives like interval hysterectomy or REBOA. This tool aids in randomizing patients for conservative management.

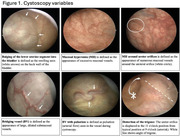

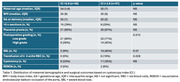

## 615 | Association Between Nt‐Probnp and Persistent Postpartum Hypertension Among Individuals with Hypertensive Disorders of Pregnancy

Rosemary Shay^1^; Carrie Bennett^1^; Caroline Smith^2^; Janet Catov^3^; Arun Jeyabalan^2^; Robin E. Gandley^4^; Malamo Countouris^1^; Alisse Hauspurg^5^

*^1^University of Pittsburgh Medical Center, Pittsburgh, PA; ^2^University of Pittsburgh School of Medicine, Pittsburgh, PA; ^3^Magee‐Womens Hospital, Pittsburgh, PA; ^4^Magee‐Womens Research Institute, Pittsburgh, PA; ^5^Alpert Medical School of Brown University, Providence, RI*

4:00 PM ‐ 6:00 PM

**Objective**: Natriuretic peptide (NP) concentrations are increased in hypertensive disorders of pregnancy (HDP), but the association of NP levels with postpartum outcomes among hypertensive individuals has not been well explored. Our objective was to evaluate the association of N‐terminal pro B‐type natriuretic peptide (NT‐proBNP) concentration at delivery with persistent postpartum hypertension (HTN) and blood pressure (BP) trajectory in the first two weeks postpartum among individuals with HDP.

**Study Design**: This cohort study included individuals with new‐onset HDP (gestational hypertension, preeclampsia) who participated in a remote BP monitoring program and an obstetric biobank. NT‐proBNP was measured from maternal plasma at delivery. Postpartum BP data were collected from delivery hospitalization, six‐week remote BP program, and outpatient visits. Outcomes were compared between low‐NT‐proBNP (< 150 pg/mL) and high‐NT‐proBNP (≥150 pg/mL) groups, with cut‐off of 150 pg/mL based on previously published reference intervals in pregnancy. We used repeated BP measures to fit mixed‐effects linear regression models using restricted cubic splines. Persistent HTN was defined as BP ≥140/90 mmHg or antihypertensive medication.

**Results**: Of 111 individuals with HDP, 31 (28%) had low NT‐proBNP, and 80 (72%) had high NT‐proBNP. Those with high NT‐proBNP had higher maximum DBP during the delivery hospitalization (101 vs. 94 mmHg, p = 0.01), higher maximum SBP during remote monitoring (145 vs. 139 mmHg, p = 0.049), and higher likelihood of being on antihypertensives at any time postpartum (49% vs. 23%, p = 0.01) compared with those with low NT‐proBNP. High NT‐proBNP was associated with persistent HTN at six weeks postpartum (40% vs. 19%, p = 0.04) compared with low NT‐proBNP. The high‐NT‐proBNP group had a significantly different SBP trajectory from the low‐NT‐proBNP group over the first two weeks postpartum (p = 0.04 for SBP, p = 0.13 for DBP).

**Conclusion**: Individuals with elevated NT‐proBNP may be at increased risk for ongoing HTN in the postpartum period and could warrant closer monitoring after delivery.

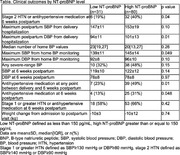

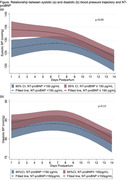

## 616 | Preeclampsia Compared to Gestational Hypertension Increases Short‐Term Renal Disease Morbidity

Ruby Lin^1^; Rachel Lee^1^; Emily E. Daggett^2^; Morgan C. Dunn^3^; Emily B. Rosenfeld^1^; Cande V. Ananth^1^

*^1^Rutgers Robert Wood Johnson Medical School, New Brunswick, NJ; ^2^Rutgers Robert Wood Johnson Medical School, Edison, NJ; ^3^Rutgers Robert Wood Johnson, New Brunswick, NJ*

4:00 PM ‐ 6:00 PM

**Objective**: Despite the physiologic differences, gestational hypertension and preeclampsia without severe features are managed similarly. We hypothesize that the presence of proteinuria in preeclampsia increases the risk of short‐term renal disease morbidity.

**Study Design**: Using the Healthcare Cost and Utilization Project Nationwide Readmissions Database, we performed a retrospective cohort study of hospital delivery discharges in the United States from 2010 to 2020. We used ICD 9 and 10 codes to identify deliveries complicated by hypertensive disease in pregnancy, as well as hospital readmissions within the calendar year of delivery for renal disease, including acute and chronic kidney disease. Chronic hypertension and superimposed preeclampsia were excluded to avoid the possibility of baseline renal disease due to preexisting hypertension. Associations were derived from Cox proportional hazard models expressed in the confounder‐adjusted hazard ratio (HR) with a 95% confidence interval (CI).

**Results**: There were 3,425,221 deliveries complicated by gestational hypertension, preeclampsia without severe features, or severe preeclampsia. Renal disease hospitalization rates for gestational hypertension, preeclampsia without severe features, and severe preeclampsia were 247, 408, and 648 per 100,000 delivery hospitalizations, respectively. Compared to those with gestational hypertension, preeclampsia without severe features was associated with increased risks of acute renal disease (HR 1.49, 95% CI 1.31‐1.68); risks for chronic renal disease were even higher (HR 2.31, 95% CI 1.70‐3.13). Patients with severe preeclampsia were at substantially increased risk of renal disease hospitalizations.

**Conclusion**: Preeclampsia has a 1.5‐ fold and severe preeclampsia a 2.4‐fold increase in renal disease morbidity compared to gestational hypertension. These findings highlight the different prognoses between gestational hypertension, non‐severe preeclampsia, and severe preeclampsia. Patients who have preeclampsia may warrant closer surveillance for long‐term kidney disease.

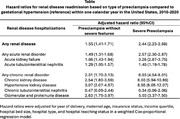

## 617 | Disparities in Fetal Autopsy and Placental Histopathology following Stillbirth in the United States

Sabrina Montgomery^1^; Katherine Bianco^2^; Hayley E. Miller^2^; Ruth B. Lathi^2^; Stephanie A. Leonard^2^

*^1^Charles R. Drew University of Medicine and Science, College of Medicine, Inglewood, CA; ^2^Stanford University, Palo Alto, CA*

4:00 PM ‐ 6:00 PM

**Objective**: Efforts to eliminate well‐known disparities in stillbirth require an improved understanding of the causes of death. Clinical guidelines recommend fetal autopsy and placental histopathology following a stillbirth, but many stillbirths do not receive these evaluations and remain unexplained. We sought to examine whether rates of fetal autopsy and placental histopathology following a stillbirth differ among racial/ethnic and education groups.

**Study Design**: We conducted a population‐based study using the U.S. CDC national fetal death files between 2020‐2022. We included singleton births ≥20 weeks’ gestation with complete information on study variables. The study outcomes were fetal autopsy and placental histopathology, categorized as none or yes: completed or planned. Birth parent race, ethnicity, and education were self‐reported and categorized by the CDC. We performed multivariable logistic regression models to evaluate differences in the outcomes among racial/ethnic and education groups. Covariates included age, late or no prenatal care, prior live birth, and gestational age.

**Results**: In this study of 43,954 stillbirths, 18% received a fetal autopsy and 67% had placental histopathology performed (Table 1). Among racial/ethnic groups, Non‐Hispanic (NH) Black people had higher rates of fetal autopsy (aOR 1.34, 95% CI: 1.26‐1.42) but lower rates of placental histopathology (aOR 0.87, 95% CI: 0.82‐0.92) than NH White people (Table 2). NH American Indian or Alaska Native and NH Asian people also had significantly lower rates of placental histopathology. Among education groups, increasing level of education was associated with increasing rate of both fetal autopsy and placental histopathology.  

**Conclusion**: Utilization of fetal autopsy and placental histopathology was lower than expected across all groups, with differences among racial/ethnic and education groups. Further research is needed to understand structural barriers, patient preferences, and causes of disparities in clinical evaluation following stillbirth. 

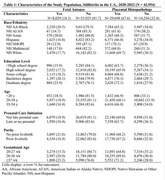

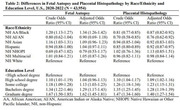

## 618 | Gender Representation in Mfm Fellowship Leadership: a Cross‐Sectional Analysis

Sally Kuehn^1^; Colleen P. Judge‐Golden^2^; Rachel L. Wood^3^; Sarah K. Dotters‐Katz^1^

*^1^Duke University School of Medicine, Durham, NC; ^2^Duke University Hospital, Durham, NC; ^3^Duke University, Durham, NC*

4:00 PM ‐ 6:00 PM

**Objective**: Women hold far fewer leadership positions in academic medicine than men. The purpose of this study was to describe the gender breakdown of maternal fetal medicine(MFM) fellowship program directors(PD) and to evaluate the association between gender and academic rank in this cohort.

**Study Design**: After IRB exemption, an inclusive list of active MFM fellowship programs was collected. Using online search tools, PD gender identity (extrapolated from written pronouns in online profiles), PD academic rank, number of years since PD fellowship graduation, and number and gender identity of current fellows were collected for each program. Data were analyzed using descriptive statistics and bivariate analyses.

**Results**: Biographical information was available for 104 of 106(98.1%) MFM fellowship PDs and 387 fellows representing 88 programs. Of the fellowship PDs, 63(61%) were women. On average, male directors had spent significantly more years out of fellowship than their female counterparts (21.0 yrs vs 15.1 yrs, p = 0.004) and were more likely to be full professors (54% vs. 30%, p = 0.03). However, when stratifying by number of years in practice (< 10 years, 10‐19 years, 20+ years), no difference in academic rank was seen between men and women within groups (Figure). Of fellows, 87(22.5%) were men. The proportion of male PDs was significantly higher than the proportion of male fellows (39% vs. 23%, p = 0.0008). There was no association between fellow gender identity and the gender identity of their respective PDs (Table).

**Conclusion**: Even in OBGYN—one of the few predominantly female specialties—gender disparities still exist in medical education leadership. In MFM, males are overrepresented in fellowship leadership compared to the gender distribution of current fellows. Our data suggest that this finding, and the difference in academic rank between male and female PDs, is related to males having been in practice for longer. As the specialty continues to evolve, attention must be paid to ensure that medical education leadership is reflective of the trainees it serves.

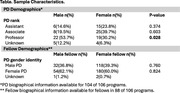

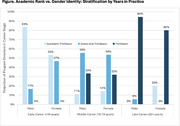

## 619 | Postpartum Mental Health Following Unplanned Modes of Delivery: Understanding Birth Trauma

Samantha L. Kruger^1^; Nicola C. Perlman^1^; Elizabeth B. Sherwin^1^; Yasser Y. El‐Sayed^2^; Deirdre J. Lyell^1^; Elliott K. Main^2^; Suzan L. Carmichael^1^; Katherine L. Wisner^3^; Pervez Sultan^1^; Stephanie A. Leonard^1^; Danielle M. Panelli^1^

*^1^Stanford University, Palo Alto, CA; ^2^Stanford University, Stanford, CA; ^3^Children's National Hospital, Washington, DC*

4:00 PM ‐ 6:00 PM

**Objective**: Birth trauma is an increasingly recognized and prevalent problem, yet the relationship between potentially traumatic birth events (such as unplanned mode of delivery) and postpartum mental health conditions is understudied. We evaluated associations between unplanned delivery modes and new onset postpartum mental health conditions.

**Study Design**: This was a retrospective cohort study of singleton live births between 2008‐2022 from the MarketScan Commercial Database. We excluded births with mental health conditions diagnosed during pregnancy, discontinuous insurance enrollment, preterm birth (< 37 weeks), contraindications to labor, and planned cesareans. The exposure was spontaneous vaginal delivery (SVD), successful operative vaginal delivery (OVD) with vacuum or forceps, unplanned cesarean without OVD attempt, or cesarean after failed OVD. The outcome was a mental health condition (depression, anxiety, post‐traumatic stress disorder (PTSD), or other serious psychiatric conditions) diagnosed between delivery and 6 months postpartum. Multivariable logistic regression models were conducted to assess associations between mode of delivery and any postpartum mental health condition, adjusting for confounders. Secondarily, models were replicated to evaluate each individual mental health condition as an outcome.

**Results**: A total of 829,917 births were included. Compared with SVD, postpartum mental health conditions were increased after unplanned cesarean without OVD [adjusted odds ratio (aOR) 1.15, 95% confidence interval (CI) 1.13‐1.18] and after unplanned cesarean with failed OVD (aOR 1.20, 95% CI 1.03‐1.38, Table 1). Prevalence of PTSD was significantly increased in all modes of delivery compared to SVD, most notably for failed OVD (aOR 1.60 95% CI 1.09‐2.26, Figure 1).

**Conclusion**: Postpartum mental health conditions were increased among people with unplanned cesarean births, most notably PTSD in those with failed OVD. Screening for PTSD should be considered, especially for people who have failed OVD attempts that result in cesarean birth.

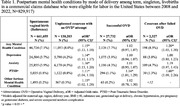

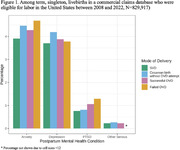

## 620 | Expectant Management versus. Prompt Laser Therapy for Stage II Twin‐Twin‐Transfusion‐Syndrome before 18 weeks

Sami Backley^1^; Eric P. Bergh^2^; Saul Snowise^3^; Anthony Johnson^4^; Jimmy Espinoza^1^; Nick Juckel^3^; Ashley Salazar^5^; Ramesha Papanna^6^; Clifton O. Brock^3^

*^1^Division of Fetal Intervention, Department of Obstetrics, Gynecology and Reproductive Sciences, McGovern Medical School at UTHealth Houston, Houston, TX; ^2^UT Health, Houston, TX; ^3^Midwest Fetal Care Center, Children's Minnesota, MN, Minneapolis, MN; ^4^McGovern Medical School University of Texas Health Science Center at Houston (UThealth), Houston, TX; ^5^Division of Fetal Intervention, Department of Obstetrics, Gynecology and Reproductive Sciences, McGovern Medical School at UT Health Houston, Houston, TX; ^6^McGovern Medical School at UTHealth Houston, Houston, TX*

4:00 PM ‐ 6:00 PM

**Objective**: In some reports, fetoscopy before 18 weeks (wks) gestational age (GA) is associated with a greater risk of poor perinatal outcomes. We assessed efficacy of expectant management (EM) followed by fetoscopic placental laser ablation (FLA) at 18 wks versus prompt intervention (PI) in patients with early‐Stage II twin‐to‐twin transfusion syndrome (TTTS).

**Study Design**: This is a secondary analysis of prospectively collected data from monochorionic diamniotic (MCDA) pregnancies with Stage 2 TTTS prior to 18 wks GA at two fetal centers. We compared outcomes between patients that had FLA promptly after evaluation (PI) to those where expectant management (EM), with twice‐weekly surveillance and intent to perform FLA at 18 wks GA, was undertaken.  For the EM group, we assessed incidence of early FLA (i.e., < 18 wks) for progressive polyhydramnios or worsening severity of disease (i.e., upstaging). Ultrasound characteristics, GA of delivery, incidence and timing of preterm prelabor rupture of membranes (PPROM) and survival were compared between groups.

**Results**: Stage 2 TTTS prior to 18 wks was confirmed in 54 MCDA pregnancies with 18 (33.3%) in the EM group and 36 (66.7%) in the PI group. In the EM group, 12 patients had FLA at 18 weeks, while 6 had early FLA (Figure). The PI group presented 3.5 days later than the EM group (p = 0.02), while time between evaluation and PLA was greater in the EM group (6 vs. 1 days, p < 0.01). Ultrasound characteristics between the groups were similar (Table). Rates of PPROM were similar between groups. However, the GA of PPROM trended later in the EM group (24.8 vs. 19.6 wks, p = 0.08) while the FLA to PPROM interval trended longer (6.7 v. 3.3 days, p = 0.31). GA at delivery was similar between groups. Dual survival rates were 88.9% and 75.0% (p = 0.30) in the EM and PI groups respectively. In the EM group, dual survival occurred in all upstaged cases.

**Conclusion**: Expectant management for early Stage II TTTS was noninferior to prompt intervention. The timing of PPROM, when it occurs, may be more favorable with EM, however further study is required to assess this possibility.

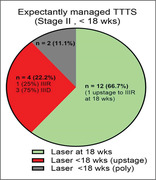

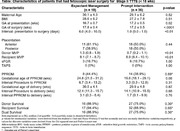

## 621 | Diagnosis of Vasa Previa: Is It Time to Sweep?

Sami Backley^1^; Eric P. Bergh^2^; Eleazar E. Soto^3^; Neha Agarwal^4^; Felicia V. LeMoine^1^; Gustavo Vilchez^5^; Anthony Johnson^6^; Jimmy Espinoza^1^; Edgar A. Hernandez‐Andrade^3^; Ashley Salazar^7^; Ramesha Papanna^3^

*^1^Division of Fetal Intervention, Department of Obstetrics, Gynecology and Reproductive Sciences, McGovern Medical School at UTHealth Houston, Houston, TX; ^2^UT Health, Houston, TX; ^3^McGovern Medical School at UTHealth Houston, Houston, TX; ^4^Division of Fetal Intervention, Department of Obstetrics, Gynecology and Reproductive Sciences, UTHealth McGovern Medical School, Houston, TX, USA, Houston, TX; ^5^UTHealth Houston, Houston, TX; ^6^McGovern Medical School University of Texas Health Science Center at Houston (UThealth), Houston, TX; ^7^Division of Fetal Intervention, Department of Obstetrics, Gynecology and Reproductive Sciences, McGovern Medical School at UT Health Houston, Houston, TX*

4:00 PM ‐ 6:00 PM

**Objective**: Undiagnosed vasa previa (VP) has devastating consequences due to fetal hemorrhage. Despite the evidence, there is a lack of screening guidelines. Utilizing the universal cervical length (CL) screening protocol at our center, the objective of the study was to identify and characterize the vasa previa distance from the internal os using color Doppler (CD) sweep on transvaginal ultrasound.

**Study Design**: This was a retrospective cohort study of singleton pregnancies that underwent universal transvaginal CL screening with CD, transvaginal sweep between 2016‐2024. VP cases were identified from an ultrasound database query. The images/clips were analyzed using the following screening methods: 1) midline still image with CD, 2) CD sweep over the cervix, and 3) CD sweep to the lateral lower uterine segment (Fig 1A). Quantitative assessment was performed for vessel type (artery, vein or both), Doppler flow (cm/sec), and distance from the internal os (cm) for each image.

**Results**: 139 of 67,369 pregnancies with transvaginal CL had VP in the diagnostic field. 98 (0.14%) VP were diagnosed of which: 47 (45%) were Type 1, 33 (34%) were Type 2 and 21 (21%) were Type 3. Midline CD view alone diagnosed 32 (33%) of cases. CD flow over the cervix diagnosed an additional 39 (40%) cases. CD flow in the lateral lower uterine segment provided diagnosis of 27 (27%) additional cases. The incidence of resolving placenta previa and velamentous cord insertion were similar in all groups. VP type 3 was more likely to be diagnosed on lateral sweep (p = 0.02). The distance between the cervical os and the fetal vessel was further away in cases diagnosed on lateral sweep compared to midline view and midline CD sweep of internal cervical os (Fig 1B).

**Conclusion**: Color Doppler sweep of the cervix and lateral lower uterine segment identifies a higher proportion of vasa previa prenatally. Fetal vessels lateral to the cervix constitute 1 in 5 of vasa previa cases. Guidelines to standardize the screening for vasa previa to include lateral aspects of the cervix should be considered.

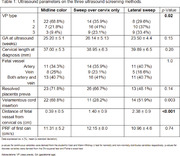

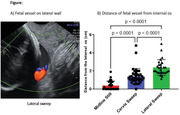

## 622 | Comorbid Anemia is Associated with Worsened Pregnancy Outcomes for Patients with Cardiac Disease in Pregnancy

Sara I. Jones^1^; Chun Xu^2^; Tracy Truong^3^; Sarah C. Snow^3^; Cary C. Ward^3^; Marie‐Louise Meng^3^; Jerome J. Federspiel^3^

*^1^Duke Univeristy, Durham, NC; ^2^Duke University, Durham, NC; ^3^Duke University School of Medicine, Durham, NC*

4:00 PM ‐ 6:00 PM

**Objective**: Our aim was to assess whether pregnant patients with comorbid anemia in the setting of cardiac disease experience an increased risk of severe maternal morbidity (SMM) and other adverse pregnancy outcomes.

**Study Design**: This was a retrospective cohort study using the 2016‐2021 National Readmissions Database of all patients with cardiac disease who delivered a singleton infant between 24 and 41 weeks. Patients with cardiac disease as identified by diagnosis codes were included and categorized based on modified World Health Organization (mWHO) criteria, which due to coding limitations was dichotomized as mWHO I or II disease (I‐II) versus mWHO II/III, III or IV (II/III+) disease. Logistic regression was used to assess the association between anemia and adverse pregnancy outcomes. A subgroup analysis of patients with iron‐deficiency anemia was conducted. Models were adjusted for age, ZIP code, median household income, primary payer, hospital size, hospital teaching status, delivery mode, and comorbid conditions.

**Results**: Cardiac disease was identified in 61,542 patients in our cohort (47,767 (77.6%) with mWHO I‐II disease and 13,775 (22.4%) with mWHO II/III+ disease). Of these patients, 16,122 (26.2%) had anemia. Comorbid anemia was associated with higher odds of SMM/mortality in both mWHO I‐II (aOR 4.65, 95% CI 4.16, 5.18) and mWHO II/III+ (aOR 2.25, 95% CI 2.05, 2.47) patients. Comorbid anemia was also associated with blood product transfusion, non‐transfusion SMM/mortality, preterm birth, and cesarean delivery (Figure). This relationship persisted for the subgroup of patients with iron‐deficiency anemia, with an increased odds of SMM in the setting of anemia seen for patients with both mWHO I‐II (aOR 3.68, 95% CI 2.95, 4.58) and mWHO II/III+ (aOR 2.14, 95% CI 1.77, 2.58) (Table).

**Conclusion**: Comorbid anemia, including iron‐deficiency anemia, adversely impacts pregnancy outcomes in pregnant patients with cardiovascular disease. Identifying and treating this modifiable risk factor offers an opportunity to reduce morbidity in this high‐risk population.

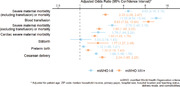

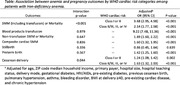

## 623 | Stigmatizing Language and the Association with Patient‐Related Factors in Pregnant Individuals with Diabetes

Anwei Gwan; Isai Ortiz; Renee Mahr; Anna Ayers Looby; Sanjana Molleti; Jessica Makori; Bukky Akingbola; Sereen K. Nashif; Katelyn M. Tessier; J'Mag Karbeah; Sarah A. Wernimont

*University of Minnesota, Minneapolis, MN*

4:00 PM ‐ 6:00 PM

**Objective**: Healthcare providers' biases can negatively influence the quality of care patients receive and exacerbate health disparities. These biases can manifest through verbal and written communication. In this study, we aimed to identify the prevalence of stigmatizing language in clinical notes and to identify the clinician and patient demographic characteristics associated with the use of such language in a population of pregnant individuals with diabetes.

**Study Design**: We conducted a retrospective cohort study from 2018‐2019 of individuals with singleton, non‐anomalous pregnancies diagnosed with pre‐existing or gestational diabetes using an academic‐community obstetric outcomes database. Using a Natural Language Processing (NLP) algorithm, we identified pre‐specified stigmatizing terms in electronic health record clinic notes (Fig. 1). We then compared the characteristics of patients with and without documented stigmatizing language in their medical records.

**Results**: Out of 1433 individuals, 128 (9%) had stigmatizing language in their medical records during their pregnancy, comprising 259 unique notes and a median of 2.0 (range 1.0‐12.0) notes per patient. The most frequently used terms were “noncompliant” (47%) and “refused” (42%). Physicians (48%), nurses (22%), and mid‐level providers (12%) were the primary providers who used this language. When adjusting for diabetes type, providers were 70% less likely to use stigmatizing language when describing white individuals compared to their black and other race counterparts (aOR 0.26, 95% CI 0.17‐0.39; p< 0.001). Additionally, all providers were almost five times more likely to use stigmatizing language when describing patients on public versus private insurance (aOR 4.95, 95% CI 3.26‐7.73; p< 0.001) (Table 1).  

**Conclusion**: Non‐white birthing individuals and those on public insurance diagnosed with diabetes in pregnancy were significantly more likely to have stigmatizing language documented within their medical records. This documentation may contribute to health inequities for marginalized birthing individuals. 

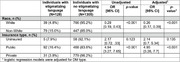

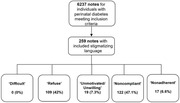

## 624 | Impact of Stigmatizing Language on Perinatal Health Outcomes of Birthing Individuals with Diabetes

Anwei Gwan; Isai Ortiz; Renee Mahr; Anna Ayers Looby; Sanjana Molleti; Jessica Makori; Bukky Akingbola; Sereen K. Nashif; Katelyn M. Tessier; J'Mag Karbeah; Sarah A. Wernimont

*University of Minnesota, Minneapolis, MN*

4:00 PM ‐ 6:00 PM

**Objective**: Healthcare providers' biases can influence patients' care and health outcomes. These biases can manifest through verbal and written communication. In this study, we evaluated the impact of stigmatizing language in clinical notes on maternal and neonatal outcomes among individuals with perinatal diabetes.

**Study Design**: We conducted a retrospective cohort study from 2018‐2019 of individuals with singleton, non‐anomalous pregnancies diagnosed with pre‐existing or gestational diabetes using an academic‐community obstetric outcomes database. Using a Natural Language Processing (NLP) algorithm, we identified ADA‐specified stigmatizing terms in electronic health record notes. We then compared the characteristics and outcomes of patients with and without documented stigmatizing language in their medical records.

**Results**: Out of 1433 individuals, 128 (9%) had stigmatizing language documented in their medical records during their pregnancy. Black individuals and those of another race were over three times more likely to have stigmatizing language than white individuals (p< 0.001).  Individuals described with stigmatizing language had birth one week earlier than their counterparts (median 38w0d vs 39w0d, p< 0.001). HgbA1c before birth was comparable between cohorts (median 5.9 vs. 5.5). Both groups had similar rates of glucose documented in the two weeks before birth, and those without stigmatizing language had more abnormal blood glucose levels recorded than those with stigmatizing language (73% vs 47%, p< 0.001). After adjusting for diabetes type and gestational age at delivery, no differences were seen between individuals with and without stigmatizing language in hypertensive disorders of pregnancy, NICU admission, or LGA infants.

**Conclusion**: The presence of stigmatizing language documented in medical records was used more often in Black and other minoritized racial groups than white individuals and was not clearly associated with markers of worse glucose control. No differences in clinical outcomes were identified, suggesting stigmatizing language may be used biasedly.

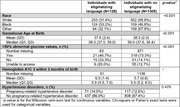

## 625 | Incidence of Placenta Accreta Spectrum in Pregnancies with Placenta Previa and Prior Dilation and Curettage

Sarah T. Mehl^1^; Abigail S. Zamorano^2^; Beatrice Valentini^3^; Joe Haydamous^4^; Neha Agarwal^5^; Han‐Yang M. Chen^1^; Edgar A. Hernandez‐Andrade^1^; Sean C. Blackwell^1^; Baha M. Sibai^1^; Farah H. Amro^1^

*^1^McGovern Medical School at UTHealth Houston, Houston, TX; ^2^Division of Gynecology Oncology, Department of Obstetrics, Gynecology, and Reproductive Sciences, University of Texas Health Science Center in Houston, McGovern Medical School, Houston, TX; ^3^University of Parma, University of Parma, Emilia‐Romagna; ^4^Division of Maternal‐Fetal Medicine, Department of Obstetrics, Gynecology and Reproductive Sciences, McGovern Medical School at UTHealth Houston, Houston, TX; ^5^Division of Fetal Intervention, Department of Obstetrics, Gynecology and Reproductive Sciences, UTHealth McGovern Medical School, Houston, TX, USA, Houston, TX*

4:00 PM ‐ 6:00 PM

**Objective**: Risk factors for placenta accreta spectrum (PAS) other than prior cesarean delivery (CD) and placenta previa remain unclear. In those with placenta previa, there is limited data regarding the role of dilation and curettage (D&C) and risk of PAS. We examined whether an association between D&C and PAS in pregnancies with placenta previa exists.

**Study Design**: Retrospective cohort study of pregnancies with finding of placenta previa on third trimester ultrasound from 2016–2024 and delivery within our hospital system. Pregnancies were divided into group 1: CD≤1 and group 2: CD≥2,  and then subclassified according to history of D&C. PAS was diagnosed based on pathology findings. Logistic regression was performed to calculate adjusted relative risk for PAS controlling for gestational age at delivery.

**Results**: A total of 274 pregnancies with third trimester placenta previa and delivery outcomes at our hospital were reviewed. Characteristics including maternal age, parity, BMI, and IVF were similar within groups (Table 1). The rate of PAS was not different among those with and without D&C in group 1 (26% versus 15%, p = 0.4) or group 2 (74% versus 67%, p = 0.2). Delivery at < 36 weeks was more frequent in those with D&C among group 1 (69% versus 47%, p = 0.016), and not different in group 2 (89% vs 83%, p = 0.73). Figure 1 demonstrates PAS pathology in both groups according to history of D&C.

**Conclusion**: In this cohort of pregnancies with placenta previa, rate of PAS was not changed based on history of D&C. We did note an association with increased placental invasion with history of D&C, although not statistically significant. Interestingly in those with prior CD≤1 and history of D&C, preterm delivery < 36 weeks was 1.5 times more likely than in those without history of D&C. A large trial is needed to evaluate the role of D&C with incidence of PAS and preterm labor.

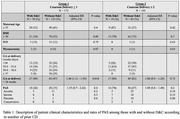

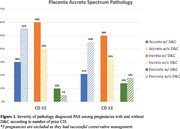

## 626 | Continuous Glucose Monitoring Profile Metrics in Pregnancies With and Without Adverse Neonatal Outcomes

Sarah A. Nazeer^1^; Rafael Bravo Santos^1^; Claudia Pedroza^1^; Joycelyn A. Corntwaithe, RD, CDE^1^; Sean C. Blackwell^2^; Suneet P. Chauhan^3^; Ghamar Bitar^2^; Baha M. Sibai^2^; Michal Fishel Bartal^4^

*^1^The University of Texas Health Science Center at Houston (UTHealth), Houston, TX; ^2^McGovern Medical School at UTHealth Houston, Houston, TX; ^3^Delaware Center of Maternal‐Fetal Medicine at Christiana Care, Delaware, DE; ^4^UTH Houston & Sheba Medical Center Israel, Houston, TX*

4:00 PM ‐ 6:00 PM

**Objective**: There is controversy concerning the degree of maternal hyperglycemia associated with adverse neonatal outcomes. Continuous glucose monitoring (CGM) is an innovative technology that allows for individualized glycemic profiles.  We aimed to determine whether CGM metrics during screening for gestational diabetes (GDM) can identify individuals who experienced adverse neonatal outcomes compared to those who did not.

**Study Design**: This was a prespecified, secondary analysis of a multi‐clinic, randomized controlled trial of individuals being screened for GDM with the use of CGM compared to the 2‐step method. Participants assigned to the 2‐step method had a blinded CGM (Dexcom G6 Pro) placed at the time of the 1‐hour glucose challenge test (GCT). CGM metrics evaluated included the mean glucose, time in range (TIR; 63–140 mg/dL), and time above the target range (TAR; >140mg/dL) expressed as % of all readings. The primary outcome was a composite of neonatal adverse outcomes (CNAO), including shoulder dystocia, birth injury, large for gestational age, need for IV/PO glucose, respiratory distress, and fetal/neonatal death. We compared CGM metrics for those with or without the CNAO.

**Results**: Of the 814 individuals randomized, 613 (75%) had CGM metrics that were analyzed (N = 331; CGM group, N = 282; 2‐step blinded CGM group). Of those 196 (32%) had the CNAO and 417 (68%) did not have CNAO. The CNAO was higher among individuals diagnosed with GDM (28% v 16%; p = 0.003) and those treated with hypoglycemic agents (10% v 5%, p = 0.04). Neonates with the CNAO had a higher mean CGM glucose (108.9 v. 105.0, p = 0.007, Figure), coefficient of variation (17.8 v. 17.1, p = 0.02), and TAR (6.5% v. 3.8%, p = 0.001) compared to those without (Table).

**Conclusion**: In this secondary analysis, CGM metrics at 24‐32 weeks of elevated mean glucose, higher coefficient of variation, and elevated TAR were associated with worse neonatal outcomes. Our findings suggest that further studies are needed to evaluate the role of various CGM metrics in diagnosing maternal hyperglycemia and related adverse outcomes.

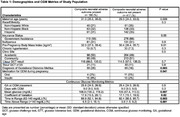

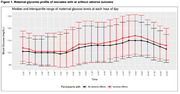

## 627 | Interval to Cerclage Placement and Delivery Timing in Patients with an Extremely Short Cervix

Sarah H. Abelman; Frank I. Jackson; Nathan A. Keller; Julie Chen; Luis A. Bracero; Cara S. Wetcher; Matthew J. Blitz

*Northwell, New Hyde Park, NY*

4:00 PM ‐ 6:00 PM

**Objective**: Patients with a very short cervical length < 1cm may benefit from cerclage placement, even in the absence of prior preterm birth. As with exam‐indicated cerclages, it is often assumed that rapid cerclage placement improves the benefit of the intervention. This study aimed to determine whether length of time from diagnosis of cervical length < 1cm to cerclage placement affects delivery timing.

**Study Design**: This was a retrospective cohort study of all patients who received an ultrasound‐indicated cerclage due to a transvaginal cervical length measurement of < 1cm during pregnancy, between January 2018 and December 2023, within a large health system in New York. Patients were excluded if they had a history of prior preterm birth. Patients were categorized based on time elapsed between this diagnosis and cerclage placement. The primary outcome evaluated was gestational age at delivery. Outcomes were compared using a linear mixed model regression analysis and were then adjusted for obesity and gestational age at diagnosis of short cervix.

**Results**: 99 patients were included who had an ultrasound‐indicated cerclage placed for a diagnosis of a cervical length < 1cm, with no prior history of preterm birth. The mean wait time for cerclage placement after diagnosis was 2.3 days. 58 (59%) patients received a cerclage the next day, and 41 (41%) waited 2 or more days. The mean cervical length at diagnosis was 5.8mm ± 2.7 mm in the next‐day placement group and was 6.9 mm ± 1.7 mm in the later placement group. The mean gestational age at diagnosis of a short cervix was 20.8 ± 1.8 weeks in the next‐day placement group and was 20.1 ± 1.7 weeks in the later placement group. The mean gestational age at delivery in the next‐day placement group was 35.4 ± 4.1 weeks, and in the later placement group was 34.7 ± 5.8 weeks, with no significant difference detected between the groups (*P* = 0.51).

**Conclusion**: For patients receiving an ultrasound‐indicated cerclage for a short cervix < 1cm, there was no benefit to rapid next‐day cerclage placement compared to longer wait times of 2 or more days.

## 628 | Accuracy of Placenta Accreta Spectrum Administrative Coding and Outcomes Inside and Outside of Referral Centers

Savvy Benipal^1^; Matthew Givens^2^; Amanda A. Allshouse^1^; Ibrahim Hammad^3^; Robert M. Silver^1^; Jason D. Wright^4^; Koji Matsuo^5^; Bret D. Einerson^6^

*^1^University of Utah, Salt Lake City, UT; ^2^Intermountain Health, Salt Lake City, UT; ^3^Intermountain Health, Intermountain Health, UT; ^4^Columbia University Irving Medical Center, New York, NY; ^5^University of Southern California/Los Angeles General Medical Center, Los Angeles, CA; ^6^University of Utah Health, Salt Lake City, UT*

4:00 PM ‐ 6:00 PM

**Objective**: Since 2015, ICD‐10 codes for placenta accreta spectrum (PAS) have been used for research for this life‐threatening pregnancy condition. However, this set of diagnostic codes have yet to be validated thoroughly and previous analyses suggest morbidity may be worse in referral centers. We sought to examine the accuracy of PAS ICD‐10 codes in and outside of PAS referral centers and to characterize the associated outcomes.

**Study Design**: We conducted a case series of all ICD‐10 diagnostic codes for PAS at two PAS referral centers within two health systems compromised of 17 delivery hospitals, representing the majority of deliveries in our state between September 2015 and December 2023. We performed ICD‐10 code validation to assess accuracy of codes and stratified results based on true and false positives. True positive codes were those cases with histopathologic evidence of PAS or FIGO clinical criteria for PAS in the absence of histopathology. We compared composite maternal morbidity with logistic regression adjusted for delivery characteristics (maternal age, prior caesarean, and gestational age) overall, stratified by PAS center.

**Results**: Of 394 deliveries with a PAS diagnostic code, 68% occurred at a PAS referral center. False positive rate of PAS diagnosis was significantly higher at non‐referral centers compared to referral centers (74% vs 24%, p< 0.001). All cases of PAS3/Percreta and nearly all cases of PAS2/Increta were managed at referral centers (Table). There was a higher odds of composite maternal morbidity at PAS centres compared to non‐referral centres (aOR 2.57; 95% CI 1.42‐4.68), but when excluding false positive cases, this association was not seen (aOR 1.55; 95%CI 0.65‐3.69, Figure). Absence of PAS3/Percreta at non‐referral centers precluded analysis of morbidity by severity. 

**Conclusion**: In contrast to referral centers, the PAS ICD‐10 code is most often inaccurate at non‐referral centres with three‐quarters being misdiagnosed. Among true positive PAS cases, despite a higher PAS severity at referral centers, maternal morbidity was not increased.

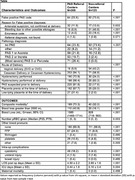

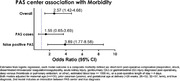

## 629 | Evaluation of Short‐term Perinatal Outcomes with Expanded Gestational Weight Gain Parameters

Savvy Benipal^1^; Susan Dalton^1^; Amanda A. Allshouse^1^; Torri D. Metz^1^; Ann M. Bruno^2^

*^1^University of Utah, Salt Lake City, UT; ^2^University of Utah Health, Salt Lake City, UT*

4:00 PM ‐ 6:00 PM

**Objective**: Most pregnant patients in the U.S. gain more weight than the Institute of Medicine (IOM) recommends. There is ongoing debate as to whether the weight cutoffs, especially for patients with obesity, optimize outcomes. We aimed to evaluate concordance with IOM gestational weight gain (GWG) guidelines compared with 10 additional pounds (lbs) and perinatal outcomes in a contemporary U.S. cohort.

**Study Design**: Secondary analysis of a prospective multicenter cohort study of nulliparous, singleton pregnancies (2010‐2013). Patients with a pre‐pregnancy body mass index (BMI) of 18.5 kg/m^2^ or greater, with pregnancy GWG within IOM guidelines or up to 10 lbs above were included. Pregnancies affected by genetic or structural anomalies, pregnancy loss < 20 weeks’, or missing key outcome data were excluded. The primary outcomes were a composite maternal morbidity and neonatal morbidity and mortality. We estimated the incidence of maternal and neonatal composites by GWG. Logistic regression modeling compared morbidity composites between individuals within IOM GWG guidelines compared with up to an additional 10 lbs, stratified by BMI category.

**Results**: Of 4487 pregnancies, 2137 (48%) had GWG within IOM guidelines and 2350 (52%) had up to an additional 10 lbs gained. Additional weight gain was associated with higher pre‐pregnancy BMI and age < 35 years. There was a higher odds of composite maternal morbidity (aOR 1.29, 95% CI 1.14‐1.47) but not neonatal morbidity (aOR 0.94, 95% CI 0.83‐1.08) in those with additional weight gain (Table). Findings were the same when stratified by BMI category among people with normal and overweight BMI but inference was limited among the obesity subgroups with statistical insignificance and wide confidence intervals (Figure).

**Conclusion**: Expanded GWG (10 lbs above IOM guidelines) was associated with higher odds of short‐term adverse maternal outcomes but not neonatal outcomes when compared with GWG within IOM guidelines. Further research is needed to identify GWG targets which optimize both maternal and neonatal health.

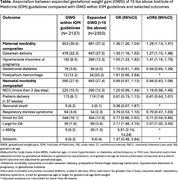

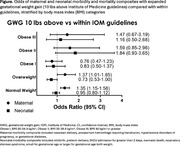

## 630 | Should Tranexamic Acid be Re‐dosed for Patients with Postpartum Hemorrhage Based on Blood Volume?

Sean C. Halsey^1^; Allison Dunn^1^; Sebastian Seifert^2^; Sixtine Gilliot^3^; Anne‐Sophie Ducloy‐Bouthors^3^; Haleem Shakur‐Still^4^; Amber Geer^4^; Naomi L. Luban^5^; Johannes van den Anker^6^; Jogarao Gobburu^1^; Ian Roberts^4^; Homa K. Ahmadzia^7^

*^1^University of Maryland School of Pharmacy, Baltimore, MD; ^2^Brigham & Women's Hospital, Boston, MA; ^3^University of Lille, Lille, Nord‐Pas‐de‐Calais; ^4^London School of Hygiene & Tropical Medicine, London, England; ^5^Children's National Hospital, Washington, DC; ^6^University Children's Hospital, Basel, Basel‐Stadt; ^7^Inova Health System, Falls Church, VA*

4:00 PM ‐ 6:00 PM

**Objective**: The objective of this study is to identify the relationship between blood loss and drug clearance and between blood loss and volume of distribution for tranexamic acid use at delivery to determine the need for scheduling subsequent doses at a fixed rate or based on the magnitude of blood loss.

**Study Design**: Four pharmacokinetic studies were reviewed, and estimates of drug clearance (CL) and volume of distribution in the central compartment (Vc) and the peripheral compartment (Vp) from prior modeling work were compared with volume of blood loss. Data from 205 individuals was divided into five equal groups based on volume of blood loss, ranging from 160 mL to 10 L. Then the relationships of interest were visualized and described. The data was further described by dividing it into clinically meaningful groups based on blood loss of < 1 L, 1‐2 L, 2‐3 L, and > 3 L. All data analysis and visualization were performed using R Statistical Software (version 4.2.2, R Core Team (2022), Viena, Austria, https://www.R‐project.org/).

**Results**: Of the 205 individuals studied, 67 experienced blood loss of at least 1 L and ten experienced a blood loss of at least 2 L. Clearance and distribution were both identified to have a minimal relationship with volume of blood lost, with CL having a weak positive trend as shown in Figure 1 (R‐squared of 0.44), and Vc and Vp having slightly negative trends (R‐squared of 0.055 and 0.20, respectively). Figure 2 shows the trend generated by Vc and blood loss. Data derived from clinically meaningful groups were very similar to that previously described with R‐square values for CL, Vc, and Vp equating 0.0061, 0.024, and 0.028, respectively.

**Conclusion**: No strong correlations were identified among the groups of interest, indicating that blood loss does not greatly affect the pharmacokinetic action of tranexamic acid. Therefore, there may be no need to base TXA redosing frequency on the magnitude of blood loss. Additional data in patients with blood loss >2 L would help to validate these findings.

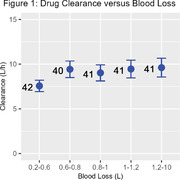

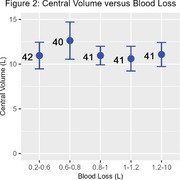

## 631 | The Risk of Hepatitis C Virus Infection on Development of Preeclampsia in Pregnancy

Sergio Karageuzian^1^; Stephen Contag^2^; Rang Kim^3^; Kriti N. Vedhanayagam^4^; Ilish Gedestad^5^; Synia Chunn^4^; Megan Marquez^3^; Ruofan Yao^4^

*^1^Loma Linda University School of Medicine, Pasadena, CA; ^2^University of Minnesota, Minneapolis, MN; ^3^Loma Linda University School of Medicine, Loma Linda University, CA; ^4^Loma Linda University School of Medicine, Loma Linda, CA; ^5^Loma Linda University School of Medicine, Redlands, CA*

4:00 PM ‐ 6:00 PM

**Objective**: To investigate the association between maternal hepatitis C virus (HCV) infection status and the development of preeclampsia during pregnancy.

**Study Design**: A retrospective cohort study was conducted using a total of 22,846,383 pregnant women who delivered preterm from 2015 to 2021. The primary exposure was HCV infection, while the primary outcome was preeclampsia. The data were obtained from medical records. Chi‐squared analyses were performed to evaluate the association between HCV and preeclampsia. 

**Results**: A total of 22, 846, 383 women were included in the study, with 104, 006 (0.46%) identified with HCV infection. Among women who are HCV negative, the prevalence of preeclampsia was 7.00% compared to 7.21% among women who are HCV positive. A significant association was indicated through chi‐squared test showing a significant association between HCV and preeclampsia (Chi‐squared = 7.4596, Pr = 0.006). 

Analysis revealed that HCV infection was associated with an increased risk of preeclampsia (adjusted OR = 1.07, 95% CI: 1.04 ‐ 1.10, P < 0.001). Other factors contributing to the risk of preeclampsia included higher BMI categories and a history of DM or chronic hypertension. Racial disparities were noted, with Black and Asian women having higher odds of preeclampsia compared to Hispanic women.

**Conclusion**: Maternal HCV infection is associated with an increased risk of developing preeclampsia.

## 632 | Ultrasound Alone versus Last Menstrual Period for Gestational Age Assignment: Implications for Fetal Growth Restriction

Shae Boguslawski^1^; Elise Turke^2^; Rebekah Pitpitan^2^; Kyra Orton^3^; Majid Shaman^4^; Sun Kwon Kim^4^; Gregory Goyert^4^

*^1^Henry Ford Health System, Department of Obstetrics and Gynecology, Detroit, MI; ^2^Wayne State University School of Medicine, Wayne State University School of Medicine/Detroit, MI; ^3^Michigan State University College of Human Medicine, Michigan State University College of Human Medicine/East Lansing, MI; ^4^Henry Ford Health, Detroit, MI*

4:00 PM ‐ 6:00 PM

**Objective**: Accurate pregnancy dating is crucial for evaluating fetal growth and diagnosing fetal growth restriction (FGR). In the first trimester, both the last menstrual period (LMP) and crown‐rump length (CRL) are typically used to determine gestational age. Our primary objective was to compare the rates of FGR diagnosed using LMP and CRL with those diagnosed using CRL alone. The secondary objective was to assess the differences in pregnancy outcomes between these two groups. 

**Study Design**: This cohort study utilized an ultrasound database including all singleton pregnancies with a first‐trimester ultrasound from 2015 to 2023. We identified two groups of FGR: those dated by LMP and CRL and those dated by CRL alone. FGR was diagnosed based on an estimated fetal weight or fetal abdominal circumference below the 10th percentile. Exclusion criteria included cases without a first‐trimester ultrasound (gestational age < 14 weeks 0 days), multiple pregnancies, in vitro fertilization pregnancies, and cases lacking a delivery record. 

**Results**: We identified 1067 pregnancies affected by FGR in our cohort (n = 14116): 733 out of 9251 pregnancy episodes were dated by LMP and CRL, and 334 out of 4865 were dated by CRL alone. The rate of FGR was higher in those dated by LMP and CRL compared to those dated by CRL alone (7.9% vs 6.8%, p<0.001).  A higher rate of FGR dated by LMP and CRL resolved by the end of pregnancy compared to FGR dated by CRL alone (28.6% vs 21.0%, p = 0.008). There were no significant differences between the groups in mean gestational age at delivery, mode of delivery, preterm births, severe FGR, abnormal umbilical artery Dopplers, and composite adverse neonatal complications. 

**Conclusion**: Although FGR diagnosed by CRL alone did not show statistically significant differences in pregnancy outcomes, the lower rate of FGR in this group suggests a potential over‐diagnosis when using both LMP and CRL. Future research should focus on the impact of modifying gestational age determination methods to potentially reduce the false positive rate of FGR and unnecessary antepartum interventions. 

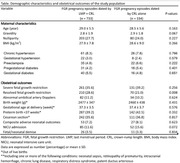

## 633 | Distant, But Connected: Pregnancy App Utilization in Maternity Care Deserts

Shannon Malloy; Katie Noddin; Leslie Saltzman

*Ovia Health, Boston, MA*

4:00 PM ‐ 6:00 PM

**Objective**: Maternity care deserts (MCDs), or counties with no hospitals or birth centers offering obstetric care and no obstetric providers, account for one‐third of all U.S. counties. MCDs are expected to grow with projected obstetric provider shortages. Digital health solutions are positioned as solutions to deliver prenatal education and care for pregnant patients in MCDs. However, little is known about digital health utilization among pregnant patients in MCDs. This analysis explores in‐app behavior, demographics, and maternity care desert status of a pregnancy tracking app's userbase.

**Study Design**: Deidentified self‐reported demographic and app usage data from users who downloaded a popular mobile pregnancy tracking app between 1/1/2022 and 7/31/2024 were assessed. The app provides gestational‐age appropriate educational content and encourages symptom logging throughout pregnancy. MCD status was assigned using self‐reported ZIP code.

**Results**: A total of n = 411,617 users qualified for inclusion. Users represented all 50 states. Almost 9% of app users lived in a maternity care desert, over double the proportion of U.S. women aged 18‐44 living in MCDs (3.8%). Comparatively, 79% of app users lived in counties with full access (FA) to maternity care, where 89% of women aged 18‐44 live. Users in MCDs were more likely to be multiparous, have a high school degree or lower educational attainment, subscribe to public insurance, report financial insecurity, and identify as American Indian or Alaska Native than users in full access counties. Users in MCDs were active in the app for 3.2 months on average, compared to 4.2 months for FA users. Almost all users enrolled in their first trimester of pregnancy. Users in MCDs logged fewer total sessions and had shorter engagement durations than FA users.

**Conclusion**: These results suggest reproductive‐aged women in MCD's are proportionally more likely to download this pregnancy tracking app compared to those in full access counties, and indicate pregnant people in MCDs may be receptive to digital health interventions to support prenatal and reproductive health.

## 634 | Female versus Male Fetuses—Variation in Fetal Heart Rate Tracing and Adverse Outcomes at Term

Shareen Patel^1^; Aaron W. Roberts^2^; Kristen A. Cagino^3^; Fabrizio Zullo^4^; Rachel L. Wiley^5^; Claudia J. Ibarra^1^; Christina Cortes^1^; Khalil M. Chahine^1^; Natalie L. Neff^6^; Kimen S. Balhotra^1^; Tala Ghorayeb^1^; Holly Flores^7^; Hector M. Mendez‐Figueroa^1^; Suneet P. Chauhan^8^

*^1^McGovern Medical School at UTHealth, Houston, TX; ^2^McGovern Medical School at UTHealth Houston, Houston, TX; ^3^UT Houston, Houston, TX; ^4^University of Rome La Sapienza, Rome, Lazio; ^5^University of California, San Diego, San Diego, CA; ^6^McGovern Medical School at UT Health, Houston, TX; ^7^University of Texas Health Science Center, Houston, TX; ^8^Delaware Center of Maternal‐Fetal Medicine at Christiana Care, Delaware, DE*

4:00 PM ‐ 6:00 PM

**Objective**: To compare the fetal heart rate tracing (FHRT) and adverse outcomes among female versus male newborns delivered at term (> 37 weeks). We hypothesized that compared to female, pregnancies with male fetuses will have more frequent FHRT abnormalities and adverse outcomes.

**Study Design**: The inclusion criteria of the retrospective study were non‐anomalous singletons, delivered at > 37 weeks after labor, whose sex at birth was either female or male. The consecutive deliveries occurred over 15 months. Using the guidelines by ACOG (Practice Bulletins 106 and 116), clinicians—blinded to maternal characteristics, gender, and outcomes—interpreted the FHRT for the last 120 min of labor. Composite neonatal and maternal adverse outcomes (CNAO and CMAO) were compared between the groups. Adjusted odds ratio (aOR) and 95% confidence intervals (CI) were calculated.

**Results**: Of the 5,160 deliveries during the study period, 3,165 (61%) met the inclusion criteria and among them 1,514 (48%) were female and 1,651 (52%), male. Compared to female, the FHRT among male was significantly more likely to have marked variability (P = 0.044), and prolonged deceleration (P = 0.039). The classification of FHRT as categories I, II or III were similar (Table 1). Cesarean delivery for non‐reassuring FHRT was more common among male (11.3%) than female newborns (6.9%; aOR 1.67; 95% 1.30‐2.14). CNAO for male (2.2%) was significantly higher than female newborns (0.9%; aOR 2.52; 95% CI 1.33‐4.77). Apgar score < 7 at 5 min (aOR 3.12, 95% CI 1.26‐7.77) was the component of the CNAO that differed significantly. CMAO was also significantly higher for individuals who delivered male (9.3%) vs female (6.3%; aOR 1.45, 95% CI 1.10‐1.92) newborns (Table 2).  

**Conclusion**: Among singleton pregnancies delivered at term, male fetuses are significantly more likely to have abnormalities for fetal heart rate, and adverse outcomes to neonatal‐maternal dyad. Investigations to elucidate the etiology and intervention trials to mitigate the disparate adverse outcomes are warranted.

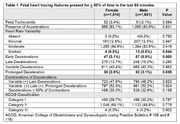

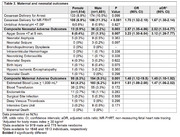

## 635 | Amniocentesis Prior to Physical Exam‐Indicated Cerclage Placement and Time to Delivery

Shweta Hosakoppal^1^; Jenna C. Meiman^1^; T. Caroline Bank^1^; Sofia Baena^1^; Courtney Denning‐Johnson Lynch^2^; Heather A. Frey^1^; Christine P. Field^1^

*^1^The Ohio State University, Columbus, OH; ^2^The Ohio State University College of Medicine, Columbus, OH*

4:00 PM ‐ 6:00 PM

**Objective**: To assess the association between amniocentesis prior to physical exam‐indicated cerclage and latency from presentation to delivery.

**Study Design**: This is a single center retrospective cohort study of pregnant individuals who presented for a physical exam‐indicated cerclage at 16w0d‐23w6d gestation from June 2012 through June 2023. The primary exposure was amniocentesis for evaluation of infection prior to attempted cerclage. The primary outcome was the latency from presentation for possible cerclage placement to delivery. Secondary outcomes were a neonatal morbidity composite, delivery < 28 weeks, delivery < 37 weeks, chorioamnionitis or endometritis, and postpartum hemorrhage. Latency from presentation to delivery was assessed by amniocentesis status using Kaplan‐Meier curves and the log‐rank test. Modified Poisson regression was used to estimate the relative risk for secondary outcomes, adjusting for confounding factors. A sensitivity analysis was performed, including only individuals with successful cerclage placement.

**Results**: Of the 78 individuals included who presented for exam‐indicated cerclage, 35 (44.9%) underwent amniocentesis. Those who underwent amniocentesis were more likely to have mild symptoms at presentation (54.3% vs 18.6%, p< 0.05), but had similar cervical length, dilation, gestational age at presentation, and history of preterm birth.  Among those in the amniocentesis group, 12 (34.3%) did not receive a cerclage compared to 3 (7%) who did not receive a cerclage in the no amniocentesis group. There was no significant difference in latency from presentation to delivery between those who underwent amniocentesis and those who did not (Figure 1). In adjusted analysis, there were no significant difference in secondary outcomes between groups (Table 1). The results were similar when limited to those with successful cerclage placement (Figure 1).

**Conclusion**: We found no association between amniocentesis prior to cerclage and latency from presentation to delivery. Given our modest sample size, these findings await confirmation in a larger study.

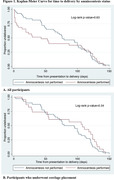

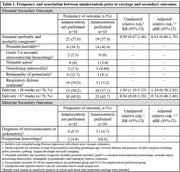

## 636 | Success of Labor Induction in Patients with Class III Obesity

Siddharth Hariharan; Shaun R. Wesley; Mary C. Gallo; Sarah Crimmins

*University of Rochester, Rochester, NY*

4:00 PM ‐ 6:00 PM

**Objective**: Labor induction in patients with class III obesity poses significant challenges and has been associated with higher complication rates. This study evaluates the success rate of labor induction and identifies factors influencing successful vaginal delivery in this population.

**Study Design**: We conducted a retrospective cohort study using US Vital Statistics Natality Birth Data from 2018 to 2022. The study included patients with class III obesity (BMI ≥ 40) who underwent labor induction. Statistical analyses included descriptive statistics and logistic regression to identify significant predictors of successful induction. Adjusted odds ratios (aOR) were calculated.

**Results**: Among the 1,053,085 patients with class III obesity who underwent induction of labor, the vaginal delivery was 25.1%.

Compared to White mothers, Black (aOR = 0.79, 95% CI: 0.78‐0.80), Asian (aOR = 0.80, 95% CI: 0.76‐0.84), and Native Hawaiian or Other Pacific Islander (aOR = 0.76, 95% CI: 0.71‐0.81) mothers had lower odds of successful induction.

Increasing maternal age was associated with decreased odds of successful induction, particularly in mothers aged 40+ years (aOR = 0.54, 95% CI: 0.52‐0.56).

Higher parity was associated with increased odds of success, especially at and after grand multiparity (aOR = 2.50, 95% CI: 2.40‐2.60).

Induction success rates were significantly lower at gestational age less than 37 weeks compared to 37 weeks and above (aOR = 0.567, 95% CI: 0.556‐0.577). The highest statistically significant success was at 40 weeks (aOR = 1.09, 95% CI: 1.081‐1.095) (Figure 1); this is likely not clinically significant given a small change in predictive probability.

At any gestational age, induction success against final birthweight followed a fitted parametric curve with peak at reference group of 3000‐3499g. (p < 0.001) (Figure 2).

**Conclusion**: The success rate of labor induction in patients with class III obesity is 25.1%. Identifying predictive factors can aid in better management and counseling. Targeted strategies are needed to improve induction success among diverse racial groups and older maternal age categories.

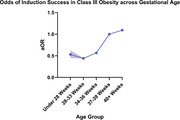

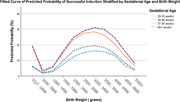

## 637 | Preconception Counseling: Understanding the Patient Experience

Simone Boney^1^; Parisa Khaligh^2^; Virginia Lijewski^3^; Jeanelle Sheeder^1^; Teresa Harper^4^

*^1^University of Colorado School of Medicine, Aurora, CO; ^2^University of Colorado, University of Colorado Obstetrics and Gynecology, CO; ^3^University of Colorado, School of Medicine, Aurora, CO; ^4^University of Colorado, Aurora, CO*

4:00 PM ‐ 6:00 PM

**Objective**: To characterize health conditions addressed during MFM preconception counseling and to evaluate patient understanding of these with visit satisfaction and provider recommendations regarding future conception.

**Study Design**: This is a prospective study of patients receiving preconception counseling by MFM specialists at the University of Colorado School of Medicine Clinics (8/20‐12/20).  Consented patients were asked to complete a 1‐week follow‐up survey about health conditions discussed at the appointment, conception recommendations, and satisfaction with counseling. Concordance of conditions discussed was defined as the patient identifying ≥80% of the conditions documented by the MFM as having been discussed. SAS was used to perform appropriate statistics.

**Results**: Of the 192 study participants, most were ≥30 years (77%), married/partnered (94%), and non‐Hispanic white (85%).  Topics discussed with the highest patient‐provider agreement were advanced maternal age (k = 0.84), hypothyroidism (k = 0.80), chronic hypertension (k = 0.70), uterine abnormalities (k = 0.68), and prior poor OB outcomes (k = 0.68). Topics with the lowest agreement were infertility (k = 0.5), genetic carrier (k = 0.08), and weight management (k = 0.14) (Figure 1). Patient satisfaction was high (97%) and similar for all topics except that those with low agreement were less likely to believe that the preconception visit would increase their chances for a healthy pregnancy (7 6.1% vs. 90.0%; p = 0.01).

**Conclusion**: Patient‐provider agreement was lowest for standardized preconception topics (e.g. weight, infertility) indicating that patients were less likely to recall topics they had not intended to discuss. Low agreement in counseling topics also impacted patients’ belief that they will have a healthy future pregnancy. Providing patient‐centered counseling using shared decision‐making is essential to improving preconception care.

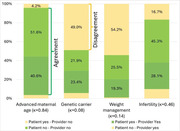

## 638 | Obstetric Antecedent Factors and Therapeutic Hypothermia: Associations with Magnetic Resonance Imaging

Sneha Paranandi^1^; Fabrizio Zullo^2^; Kelley Kovatis^1^; Anthony C. Sciscione^1^; Matthew K. Hoffman^1^; Suneet P. Chauhan^3^

*^1^Christiana Care Health System, Newark, DE; ^2^University of Rome La Sapienza, Rome, Lazio; ^3^Delaware Center of Maternal‐Fetal Medicine at Christiana Care, Delaware, DE*

4:00 PM ‐ 6:00 PM

**Objective**: Magnetic resonance imaging (MRI) abnormalities following neonatal therapeutic hypothermia (THT) for hypoxic ischemic encephalopathy (HIE) are associated with long‐term neurocognitive impairment. We compared the obstetric antecedent factors among newborns who underwent THT for HIE and had abnormal versus abnormal MRI findings.

**Study Design**: Utilizing ICD‐10 codes, newborns at a large teaching hospital with clinically diagnosed HIE and who underwent THT were identified.  Data was extracted from each chart by an obstetrician.  Inclusion criteria for analysis were THT for HIE at > 36 weeks on a non‐anomalous singleton newborn, who had a brain MRI during admission.  The MRI was considered abnormal if the following were noted: basal ganglion injury; white matter/watershed‐predominant pattern of injury; or, near total injury. A Chi‐square test was done with P < 0.05 considered significant.

**Results**: During the 5 year study period (2019‐2023), 136 neonates out of 32,765 live births received THT for moderate to severe HIE (4 per 1,000 births). Among them 117 (86%) met inclusion criteria and 28 (23%) had abnormal MRI findings. Nulliparity (P = 0.048) and tobacco use (P = 0.003) were more common among mothers of those neonates with abnormal MRIs. Fetal heart tracing (FHRT) categorization within 60 minutes of delivery was not significantly associated with neonatal MRI abnormalities (Table 1). Newborns delivered vaginally had a higher incidence of abnormal MRI (P = 0.006) compared to those delivered by cesarean. Apgar scores < 5 at 5 and 10 minutes and umbilical arterial base deficit were similar for the two groups.  Small for gestational age neonates were more likely to have abnormal MRI findings (P = 0.003; Table 2).

**Conclusion**: Hypoxic ischemic encephalopathy requiring therapeutic hypothermia occurred in 4 per 1,000 births, and among them maternal tobacco use is a modifiable risk factor for abnormal MRI. Fetal heart tracing, Apgar scores at 5 and 10 min, and umbilical arterial acid‐base status did not differentiate between abnormal versus normal MRI, a surrogate for long term sequelae.

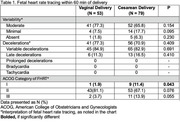

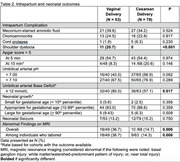

## 639 | Risk Factors for Post‐Operative Rectus Sheath Hematoma Following Cesarean Delivery: a Case Control Study

Sonya Fabricant; Ojiugo Onwumere; Camelita Thrift; Naomi Greene; Gabriela Dellapiana; Mariam Naqvi

*Cedars Sinai Medical Center, Los Angeles, CA*

4:00 PM ‐ 6:00 PM

**Objective**: To identify risk factors for rectus sheath hematoma (RSH) after cesarean delivery.

**Study Design**: We conducted a case‐control study of cesarean deliveries performed at a quaternary academic hospital between January 1, 2013 and December 31, 2023. Cases were patients who developed RSH following cesarean delivery and were identified using the Deep6 natural language processing search engine. Controls were patients without RSH and were matched 1:3 by age, race, and prior cesarean history. Demographic data, operative details, and perinatal outcomes were abstracted from the medical record and compared between cases and controls using t‐test and chi‐square as appropriate. Multivariable logistic regression was then performed to identify independent risk factors for RSH.

**Results**: Of 22,104 cesarean deliveries during the study period, 32 (0.15%) developed postoperative RSH. There were no significant differences between groups in the rates of rectus muscle closure, rectus muscle transection, peritoneal closure, or bladder flap creation (Table 1). Univariate analysis revealed an increased odds of RSH with hypertensive disorders of pregnancy (14.6 vs 34.4%, p = 0.01), placental abruption (0 vs 6.3%, p = 0.01), multiple gestation (3.1 vs 12.5%, p = 0.04), longer duration of cesarean (60.8 vs 78.1 minutes, p = 0.004), anticoagulation during hospitalization (1.0% vs 9.4%, p = 0.02) and post‐operative anticoagulation (0 vs 9.4%, p = 0.002) (Table 2). After multivariable adjustment, independent predictors of RSH were anticoagulation during hospitalization (adjusted odds ratio [aOR] 16.3, 95% confidence interval [CI] 1.5–174), multiple gestation (aOR 7.5, 95% CI 1.4–38.3), and cesarean duration > 90 minutes (aOR 8.7, 95% CI 2.8–27.3).

**Conclusion**: Anticoagulation and prolonged surgical time are independent risk factors for RSH after cesarean delivery. Notably, rectus muscle closure was not associated with RSH.

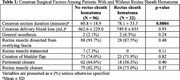

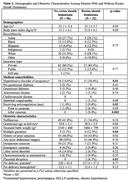

## 640 | Association Between Induction of Labor and Birth Experience: a National Population‐Based Study

Sophia Braund^1^; Francois Goffinet^2^; Aurélien Seco^3^; Camille Le Ray^4^

*^1^Université paris cité, inserm, CRESS, EPOPE, Paris, Ile‐de‐France; ^2^Université Paris Cité, Inserm, Centre for Research in Epidemiology and StatisticS (CRESS), Obstetrical Perinatal and Pediatric Epidemiology Research Team (EPOPé), Paris, Ile‐de‐France; ^3^Université Paris Cité, Inserm, Centre for Research in Epidemiology and StatisticS (CRESS), Obstetrical Perinatal and Pediatric Epidemiology Research Team (EPOPé),, Paris, Ile‐de‐France; ^4^INSERM UMR 1153, Obstetrical, Perinatal and Pediatric Epidemiology Research Team (Epopé), Center for Epidemiology and Statistics, FHU PREMA, Université de Paris, Paris, Ile‐de‐France*

4:00 PM ‐ 6:00 PM

**Objective**: Induction of labor (IOL) is rising in high income countries. The ARRIVE trial, with a selected population due to randomized trials, found increased satisfaction among induced women. However, birth experience in case of IOL should be assessed in real life. Our objective is to assess the association between IOL and birth experience using a national population‐based survey

**Study Design**: Using the French national perinatal survey 2021, we included women with a term singleton cephalic infant and a trial of labor. At 2 months, women answered the question “What kind of memories do you have about your childbirth?”. We used univariate and multivariate analyses to assess the association between IOL and birth experience, and weighting to account for missing data. We identified and assessed as potential mediators prolonged labor ≥12 hours and ‘birth with intervention or complication’ (caesarean and/or operative vaginal delivery and/or PPH and/or OASIS and/or NICU), and estimated the indirect and residual effects using mediation modelling.

**Results**: Of the 6271 women included in the main analysis, 1800 (28.7 %) underwent IOL. Overall, women with IOL had more often a bad birth experience than women with spontaneous onset of labor (SOL) (16.4% vs 8.8%, p< 0.01), and the association remained significant after adjustment (ORa 1.78 (1.47‐2.16)). In case of cervix ripening, the rate of bad birth experience rose to 18.8%. Analyses on a subgroup of women at low maternal risk found similar results. Prolonged labor explained only 6% of the association between IOL and bad experience.  Birth with complications or interventions concerned 43.8% of women with IOL and 31.1% with SOL (p< 0.01) and explained 26% of this association.

**Conclusion**: IOL was associated with a higher risk of bad birth experience than SOL.  Only a small part of this association is mediated by medical complications or interventions and prolonged labor. In the context of increase in inductions, our findings underline the importance of considering birth experience in the decision‐making and information for women and the need to explore consequences of IOL.

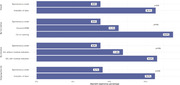

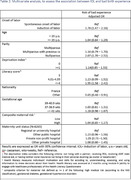

## 641 | Association of Maternal Hypotension with Abnormal Uterine Artery Dopplers and Small for Gestational Age Neonates

Stephanie Pearson^1^; Baillie A. Bronner^2^; Annie Dude^3^; Samantha de los Reyes^1^

*^1^Rush University Medical Center, Chicago, IL; ^2^MAHEC, Asheville, NC; ^3^University of North Carolina, Chapel Hill, NC*

4:00 PM ‐ 6:00 PM

**Objective**: To evaluate the association of abnormal uterine artery (UtA) dopplers in patients with persistent hypotension with delivery of a small for gestational age (SGA) neonate. We hypothesize that abnormal blood flow to the uterus may result in smaller neonates.

**Study Design**: This was a secondary analysis of the Nulliparous Pregnancy Outcomes Study: Monitoring Mothers‐to‐Be of patients with a singleton gestation at > 24 weeks with abnormal UtA dopplers (defined as pulsatility index > 95th percentile for gestational age or presence of a diastolic notch) between 18 weeks 0 days and 23 weeks 6 days gestation. Persistent hypotension was defined as systolic blood pressure < 100 mmHg and/or diastolic blood pressure < 60mmHg at all 3 study visits between 6 weeks 0 days and 29 weeks and 6 days gestation. The primary outcome was delivery of an SGA neonate. Univariable analyses were performed to evaluate demographic and clinical associations with the primary outcome. Multivariable analyses were performed to adjust for potential confounders selected a priori (age, race and ethnicity, insurance status, pre‐pregnancy body mass index (BMI), and history of chronic hypertension).

**Results**: 5,092 patients met inclusion criteria; 137 (2.7%) had persistent hypotension, 4,955 (97.3%) did not. Patients in the hypotension group were less likely to be of White race and Asian ethnicity, and more likely to have public insurance and a lower pre‐pregnancy BMI, and less likely to use tobacco within 3 months of pregnancy (Table 1). Patients with persistent hypotension and abnormal UtA dopplers were more likely to deliver an SGA neonate (11.8 vs. 18.2%, p‐value 0.025) but this did not persist in multivariable analysis (aOR 1.43, 95% CI 0.91, 2.24). In univariable analysis of secondary outcomes, the hypotension group had a lower median birth weight (3,323 vs. 3,239 grams, p‐value 0.01) (Table 2).

**Conclusion**: In patients with abnormal UtA dopplers, persistent hypotension was not associated with an increased risk of an SGA neonate.

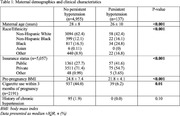

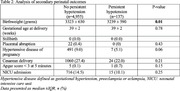

## 642 | Hybrid Closed‐Loop Therapy Versus Standard Therapy for Type 1 Diabetes in Pregnancy: a Meta‐Analysis

Lucas G. Miranda‐Martinez^1^; Molly Beestrum^2^; Charlotte M. Niznik^1^; Lynn M. Yee^1^; Stephanie A. Fisher^1^

*^1^Northwestern University Feinberg School of Medicine, Chicago, IL; ^2^Galter Health Sciences Library, Northwestern University Feinberg School of Medicine, Chicago, IL*

4:00 PM ‐ 6:00 PM

**Objective**: Hybrid closed‐loop (HCL) therapy, which integrates an insulin pump with a continuous glucose monitor to automatically adjust basal insulin rates, improves glycemic control in non‐pregnant individuals with type 1 diabetes (T1DM). Although it is not yet approved for pregnancy, recent studies suggest potential benefits to HCL in pregnant people with T1DM. This meta‐analysis aims to evaluate clinical outcomes associated with HCL vs. standard therapy (ST) during pregnancy.

**Study Design**: In this meta‐analysis, a predefined, systematic, librarian‐assisted search of Ovid MEDLINE, Embase, Scopus, Cochrane, ClinicalTrials.gov, and World Health Organization International Clinical Trial Registry Platform initially yielded 295 studies related to HCL in pregnancy. Glycemic metrics (time‐in‐range, TIR [63‐140mg/dL]; time‐above‐range, TAR; time‐below‐range, TBR; coefficient of variation, CV; mean glucose; hemoglobin A1c, HbA1c), were compared by trimester in those exposed to HCL vs. ST (i.e. sensor‐augmented pump therapy or multiple daily injections). Maternal and neonatal outcomes were secondarily assessed. We calculated standardized mean differences (SMD) and pooled odds ratios (OR) with 95% confidence intervals (CI) using random effects models.

**Results**: Five studies (3 randomized trials, 1 prospective cohort, 1 case series) published from 2022‐24 met eligibility criteria and evaluated 183 pregnancies exposed to HCL vs. 178 exposed to ST. TIR and TAR did not differ by HCL vs. ST in any trimester (Table 1). TBR was lower with HCL use in the 2^nd^ (‐1.1% [‐2.2%, ‐0.03%]) and 3^rd^ (‐1.4% [‐1.9%, ‐0.8%]) trimesters. CV was also lower with HCL use in the 1^st^ (33.8 vs. 36.1%, SMD ‐2.6% [95%CI ‐4.3%, ‐0.8%]), 2^nd^ (31.6% vs. 33.6%, SMD ‐2.1% [95%CI ‐2.9%, ‐1.3%]), and 3^rd^ (29.1% vs. 30.9%, SMD ‐1.5% [95%CI ‐2.2%, ‐0.8%]) trimesters. Maternal and neonatal outcomes did not differ between groups (Table 2).

**Conclusion**: Use of HCL for pregnant people with T1DM is associated with lower TBR and glycemic variability, with similar maternal and neonatal outcomes, suggesting a potential safety benefit that warrants further study.

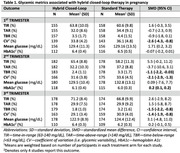

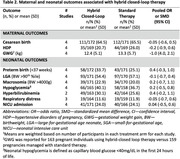

## 643 | Clinical Outcomes in Pregnant People with Type 1 Diabetes: Does Age of Diabetes Onset Matter?

Lucas G. Miranda‐Martinez; Charlotte M. Niznik; Lynn M. Yee; Stephanie A. Fisher

*Northwestern University Feinberg School of Medicine, Chicago, IL*

4:00 PM ‐ 6:00 PM

**Objective**: Metabolic differences exist between childhood‐ and adult‐onset type 1 diabetes mellitus (T1DM), yet how these may be associated with perinatal outcomes is poorly understood. We aimed to evaluate the association of age of onset of T1DM with antenatal glycemic control and perinatal outcomes.

**Study Design**: In this single‐center retrospective cohort study (2018‐2023), we compared pregnant people with childhood‐ (< 18 years of age) vs. adult‐onset (≥18 years of age) T1DM who used a continuous glucose monitor (CGM). We assessed maternal (gestational weight gain [GWG], hypertensive disorders of pregnancy [HDP], cesarean birth), neonatal (preterm birth [PTB], large‐for‐gestational age, neonatal intensive care unit admission, hypoglycemia), and glycemic outcomes (CGM metrics, hemoglobin A1c [HbA1c]; by trimester). Multivariable logistic and linear regression were used to determine adjusted odds ratios (aOR) or β‐coefficients (β) and 95% confidence intervals (CI), adjusted for pre‐gravid body mass index (BMI).

**Results**: Of 102 eligible individuals, 61 (60%) had childhood‐onset T1DM. Individuals with childhood‐onset (vs. adult‐onset) T1DM were younger (32 vs. 35 years old, p = 0.01) and had higher pre‐gravid BMI (26 vs. 24 kg/m^2^, p = 0.01). Although HDP was more frequent among those with childhood‐onset T1DM (39% vs. 27%), adjusted odds of all maternal outcomes were similar by age of onset (Table 1). The adjusted odds of PTB (aOR 3.48 [95% CI 1.02, 11.8]) were higher in individuals with childhood‐onset, compared to those with adult‐onset T1DM, but all other neonatal outcomes were similar. Childhood‐onset (vs. adult‐onset) T1DM was associated with lower 1^st^ trimester time‐in‐range (57% vs. 65%, β ‐7.36 [95% CI 0.63, 14.08]); all other per‐trimester glycemic metrics were similar between groups (Table 2).

**Conclusion**: Childhood‐onset, compared to adult‐onset, T1DM is associated with higher odds of PTB, but similar glycemic control in pregnancy aside from lower 1^st^ trimester TIR. These findings may inform risk counseling and surveillance in those with childhood‐onset T1DM to mitigate risk of adverse perinatal outcomes.

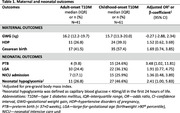

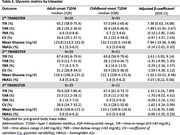

## 644 | Diagnostic rate of Pulmonary Embolism during Pregnancy: An update on VQ vs CT

Suzanne O'Nan^1^; Julia Burd^2^; Nandini Raghuraman^3^; Antonina I. Frolova^4^; Michael Dombrowski^5^; Roxane Rampersad^2^; Jeannie C. Kelly^3^

*^1^University of Minnesota, Minneapolis, MN; ^2^Washington University in St. Louis, St. Louis, MO; ^3^Washington University School of Medicine in St. Louis, St. Louis, MO; ^4^Washington University School of Medicine, St. Louis, MO; ^5^Washington University in Saint Louis, St. Louis, MO*

4:00 PM ‐ 6:00 PM

**Objective**: Diagnosing pulmonary embolism (PE) during pregnancy can be challenging. We sought to compare PE diagnosis rates during pregnancy based on the original imaging modality of ventilation‐perfusion (VQ) scan versus CT angiography (CTA).

**Study Design**: We performed a retrospective chart review of pregnant participants who underwent either VQ scan or CTA due to clinical suspicion of PE from January 2018‐ April 2024 at a single academic institution. Patients who underwent imaging for other clinical reasons, postpartum patients, and deliveries at an outside hospital were excluded. Our primary outcome was a scan considered diagnostic by the radiology read. Secondary outcomes included if a second imaging modality was pursued for diagnosis and reasons why initial imaging was non‐diagnostic. Chi‐squared, Fisher's exact, Student's t and Wilcoxon Rank sum tests were used as appropriate.

**Results**: 113 patients were included; 80 (71%) were imaged by VQ scan, and 33 (29%) were imaged by CTA. Baseline demographics and major VTE risk factors were similar between groups, including BMI and trimester of pregnancy; most patients were in the third trimester (Table 1). Diagnostic rates were similar between VQ and CTA groups (62.5% v 72.7%, P = 0.29), and 33.3% of patients in each group with non‐diagnostic findings underwent further testing to evaluate for PE (Table 2). PE was present in a minority of patients in both cohorts, and most non‐diagnostic scans were due to poor timing of dye/opacification of vessels (Table 2).

**Conclusion**: Despite improvements in technological imaging studies, many studies of both VQ and CTA are non‐diagnostic to exclude PE in pregnancy. Causes for non‐diagnostic imaging studies should be investigated in the future.

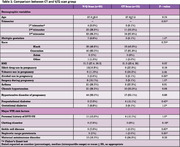

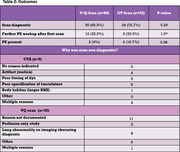

## 645 | First‐Trimester Fasting Glucose Levels Associated with Abnormal Glucose Challenge and Tolerance Tests Later in Pregnancy

Tamar Katzir^1^; Tal Schiller^2^; Ronit Koren^3^; Moran Frid^4^; Alon Ben Arie^5^; Edi Vaisbuch^2^; Oren Barak^5^

*^1^Kaplan Medical Center, Tal shahar, HaMerkaz; ^2^Kaplan Medical Center, Rehovot, HaMerkaz; ^3^Shamir medical center, Shamir medical center, HaMerkaz; ^4^Kaplan Medical Center, Kaplan medical center, HaMerkaz; ^5^Kaplan medical center, rehovot, HaMerkaz*

4:00 PM ‐ 6:00 PM

**Objective**: Gestational diabetes mellitus (GDM) is diagnosed using an initial glucose challenge test (GCT), and if exceeds 140 mg/dL is followed by an oral glucose tolerance test (OGTT). The definition of pathological first‐trimester fasting glucose (FG) is controversial, and its correlation with abnormal GCT and OGTT is not well established. This study aims to evaluate the association between first‐trimester FG levels and pathological GCT and OGTT results.

**Study Design**: We retrospectively analyzed the electronic medical records of a large healthcare provider searching for individuals who delivered between 2012 and 2024. Inclusion criteria were singleton pregnancies and a documented first‐trimester FG, excluding those with preexisting diabetes. Only the first documented pregnancy was included. We assessed the cohort's distribution of first‐trimester FG values and compared the rates of abnormal GCT and OGTT between those with FG above and below the 95th percentile (two standard deviations).  The primary outcome was the incidence of abnormal GCT and OGTT above vs. below the 95th percentile of FG.

**Results**: The study comprised 202,960 individuals who met the inclusion criteria. The mean first‐trimester FG level was 84.6±9.9 mg/dL, and the 95th percentile was 100 mg/dL. Those with an FG≥100 mg/dL were older and had a higher BMI (31.0±5.7 vs. 29.8±5.5 years, p< 0.001 and 28.2±6.7 vs. 25.1±14.9 kg/m^2^, p< 0.0001, respectively). Moreover, they had higher pathological rates of GCT and OGTT (19.2% vs. 13.1%, p>0.001, and 6.9% vs. 2.4%, p>0.001, respectively). We observed a positive association between increasing FG levels and pathological GCT and OGTT results **(Fig. 1)**.

**Conclusion**: First‐trimester fasting glucose levels of 100mg/dL or higher are significantly associated with increased rates of pathological glucose challenge test and oral glucose tolerance test. Further consideration should be given to adjusting pregnancy management for these high‐risk populations.

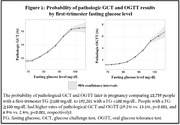

## 646 | Fetal Fibronectin for Prediction of Spontaneous Labor at Term

Taylor S. Freret^1^; Anjali J. Kaimal^2^; William H Barth, Jr.^3^; Shuo Wang^4^; Amy Herring^4^; Julianna Schantz‐Dunn^5^; Michael Ruma^6^; Sarah E. Little^1^

*^1^Beth Israel Deaconess Medical Center, Boston, MA; ^2^University of South Florida, Tampa, FL; ^3^Massachusetts General Hospital, Boston, MA; ^4^Duke University, Durham, NC; ^5^Brigham and Women's Hospital, Boston, MA; ^6^Perinatal Associates of New Mexico, Albuquerque, NM*

4:00 PM ‐ 6:00 PM

**Objective**: Elective induction of labor (IOL) at 39 weeks reduces cesarean delivery; however, many individuals hope to labor spontaneously and be informed participants in IOL decision‐making. Fetal fibronectin (fFN) predicts risk of spontaneous preterm delivery. We sought to determine if fFN predicts spontaneous term labor.

**Study Design**: Prospective observational multicenter study within one healthcare system. Nulliparous patients without a medical indication for IOL prior to 41 weeks were offered enrollment at their 38‐week prenatal visit. After informed consent, a provider collected a fFN and performed a cervical exam. The primary outcome was spontaneous onset of labor. The predictor of interest, quantitative fFN value, was dichotomized to positive (≥50 ng/mL) or negative (< 50 ng/mL) based on qualitative standards in the US. Secondary outcomes included time from study visit until delivery admission. Logistic and Cox proportional hazards regression were used; adjusted models controlled for gestational age and Bishop score at study visit.

**Results**: 290 patients consented, of whom 284 were included in the analysis with a mean age of 30.1 years and BMI of 24.9. 59% were White, and 22% delivered by cesarean. 173 (60.9%) went into spontaneous labor and 109 (38.4%) were induced, of which 32 (29.4%) were medically indicated and 67 (61.5%) were elective inductions prior to 41 weeks. Mean quantitative fFN value was 46 ± 109 ng/mL, and mean Bishop score was 3.6 ± 2.2 cm. A positive fFN was associated with an increased odds of spontaneous labor (aOR 2.16, 95% CI 1.04–4.86, Table). All patients with a positive fFN who were not induced prior to 40w6d (medically indicated or elective) entered labor spontaneously. A positive fFN was associated with an increased adjusted hazard of spontaneous labor (aHR 1.88, 95% CI 1.27–2.78, Figure), which persisted after accounting for potential informative censoring due to non‐spontaneous deliveries.

**Conclusion**: A positive fFN is associated with an increased odds of spontaneous labor after 38 weeks. Our findings may help in counseling patients considering elective IOL after 39 weeks.

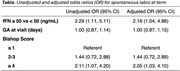

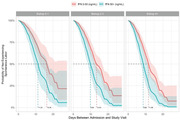

## 647 | Neonatal Outcomes in Individuals with Periviable Pre‐Eclampsia

Tejumola Apata; Sarah Crimmins; On behalf of the Maternal‐Fetal Medicine Units (MFMU) Network and NICHD

*University of Rochester, Rochester, NY*

4:00 PM ‐ 6:00 PM

**Objective**: To compare neonatal outcomes in pregnancies complicated by pre‐eclampsia versus patients without pre‐eclampsia between 22w0d and 25w0d.

**Study Design**: Retrospective cohort study utilizing the Registry of Obstetrical Determinants of Neonatal Survival (ODNS) of pregnancies with delivery between 22w0d‐25w0d.  Individuals with preeclampsia were compared to individuals delivered during the same gestational age without preeclampsia. The primary outcome is a composite measure of neonatal outcomes which includes neonatal death (death within 120 days of delivery), seizures, necrotizing enterocolitis requiring surgery, oxygen dependence at discharge or at 120 days of age, Grade III or IV retinopathy of prematurity and Grade III/IV CNS hemorrhage. Multivariate analysis was performed with Pearson Chi‐squared tests, fisher's exact test to analyze categorical variables and two tailed test to analyze continuous variables.

**Results**: A total of 339 pregnancies met inclusion criteria. 18(5.3%) was delivered for severe pre‐eclampsia while 321(94.75%) delivered for other indications. Both groups were similar in terms of umbilical pH, estimated gestational age, number of days on a ventilator and number of days spent in the NICU. There was a significant difference in the median birth weight of the pre‐eclampsia group compared to the non‐pre‐eclampsia group (519grams versus 614gram, P = 0.016) In the pre‐eclampsia group, 6(33.3%) of neonates were alive at  120 days of birth, compared to 121(37.7%) in the non‐pre‐eclampsia group(p = 0.806). The composite neonatal outcome was similar between both groups (16(88.%) for preE vs 274(84.8%), p = 0.638). 

**Conclusion**: Infants born in the periviable period secondary to preeclampsia experience similar neonatal outcomes in comparison to other indications for preterm delivery. 

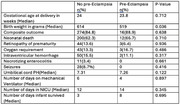

## 648 | Neonatal Survival in Individuals with Periviable Pre‐Eclampsia

Tejumola Apata; Sarah Crimmins

*University of Rochester, Rochester, NY*

4:00 PM ‐ 6:00 PM

**Objective**: To compare neonatal survival in pregnancies complicated by preeclampsia versus patients without pre‐eclampsia(preE) between 22w0d and 24w6d.   

**Study Design**: Retrospective cohort study utilizing the US Vital Statistic Natality Birth Data of pregnancies with delivery between 22w0d‐24w6d from 2018 to 2022.  Individuals with preeclampsia were compared to individuals delivered during the same gestational age without preeclampsia. The primary outcome is infant survival as documented at the time of birth certificate report . Secondary outcomes include neonatal  seizures and assisted ventilation for >6 hours. Multivariate analysis was completed with Pearson Chi‐squared tests to analyze categorical variables. 

**Results**: A total of 38,024 met inclusion criteria. 253(0.7%) of pregnancies were affected by PreE while 37,660(99.0%) did not have preE. At the time of the birth certificate report, 201(79.4%) infants survived in the preE group compared to 26,691(70.9%) infants in the non‐preE group(P< 0.003, OR 1.6; 95% CI: 1.18‐2.20). However, when analyzed by gestational age week by week, neonatal survival rates were similar between the two groups: [22 weeks (18(52.9%) preE vs. 3,832(44.7%) non‐preE, P = 0.336), 23 weeks(69(78.4%) preE vs. 8,922(72.1%) non‐ preE,  P = 0.095,  and at 24 weeks(114(87.0%) preE vs.13,937(84.4%) non‐ preE, p = 0.530]. There is a statistically significant difference in number of neonates with seizures (0.4% in the preE group vs 0.2% in the non‐ preE group P< 0.001) and neonates requiring ventilation for more than 6 hours (33.1% in the preE group versus 32.9% in the non‐preE group p< 0.001).

**Conclusion**: The overall survival of neonates born in the periviable period in pregnancies complicated by preeclampsia is 79.4%.  The chance of survival increases with each week of continued pregnancy in the periviable period. Given the rarity of periviable preeclampsia, the rates of survival are similar to neonates born in the periviable period for other indications. 

## 649 | Deliveries with a Disability Diagnosis and Risk for Adverse Maternal Outcomes in the U.S., 2000‐2021

Teresa C. Logue^1^; Fabrizio Zullo^2^; Suneet P. Chauhan^3^; Kartik K. Venkatesh^4^; Timothy Wen^5^

*^1^Christiana Care Health System, Newark, DE; ^2^University of Rome La Sapienza, Rome, Lazio; ^3^Delaware Center of Maternal‐Fetal Medicine at Christiana Care, Delaware, DE; ^4^The Ohio State University, Columbus, OH; ^5^University of California, San Diego, Irvine, CA*

4:00 PM ‐ 6:00 PM

**Objective**: There is limited data on pregnancy outcomes of people with disabilities. People with disabilities are more likely to have chronic conditions and adverse social determinants of health, which are risk factors for pregnancy complications. We evaluated the association between disability diagnosis and severe maternal morbidity (SMM) and other adverse outcomes.

**Study Design**: We conducted a serial cross‐sectional analysis of delivery hospitalizations with a physical, intellectual or sensory disability diagnosis in the National Inpatient Sample during 2000‐2021. Our primary outcome was non‐transfusion SMM. Secondary outcomes were transfusion, hypertensive disorders of pregnancy (HDP), preterm delivery (PTD), and maternal death. We described temporal trends in SMM by presence of any disability, reporting average annual percent change (AAPC). We fit logistic regression models to evaluate the association between any disability and our outcomes, adjusting for demographic and obstetric factors and chronic conditions. In subgroup analyses, we evaluated risk for SMM by disability type.

**Results**: Of 83.7 million total deliveries, 857,876 (1.0%) had a disability diagnosis, with 83.8% of disabilities physical, 6.0% intellectual, and 10.2% sensory. Deliveries with any disability had threefold higher risk of SMM (2.7% vs 0.7%; adjusted OR [aOR] 3.33, 95% CI 3.22, 3.44) as well as higher risk for transfusion (aOR 2.02, 95% CI 1.94, 2.09), HDP (aOR 1.50, 95% CI 1.47, 1.52), PTD (aOR 1.38, 95% CI 1.36, 1.41) and death (unadjusted OR 8.26, 95% CI 6.43, 10.61). Risk for SMM was elevated across all disability types but highest in the setting of physical disability (aOR 3.37, 95% CI 3.25, 3.49). Incidence of SMM among deliveries with any disability increased from 2000 to 2021 (AAPC 3.1%, 95% CI 2.4%, 3.8%).

**Conclusion**: Pregnant people with disabilities have threefold higher risk of SMM, even after adjusting for social risk factors and chronic disease. This risk persists across all disability types and is increasing over time. Future research is needed to develop interventions to improve pregnancy outcomes in this population.

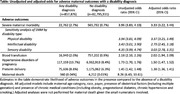

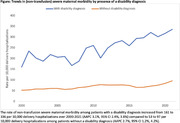

## 650 | Patient‐Directed Discharge During Antepartum Hospitalization and Risk for Adverse Outcomes at Delivery

Teresa C. Logue^1^; Timothy Wen^2^; Fabrizio Zullo^3^; Fiamma van Biema^4^; Alexander M. Friedman^5^

*^1^Christiana Care Health System, Newark, DE; ^2^University of California, San Diego, Irvine, CA; ^3^University of Rome La Sapienza, Rome, Lazio; ^4^Hackensack Meridian School of Medicine, Hackensack Meridian School of Medicine, NJ; ^5^Columbia University Irving Medical Center, New York, NY*

4:00 PM ‐ 6:00 PM

**Objective**: Antepartum (AP) hospitalizations are typically unplanned and often cause social stress. Patients with social and mental health risk factors may be more likely to leave the hospital via patient‐directed discharge (PDD). We evaluated predictors of PDD at AP hospitalization and the effect of AP PDD on risk for adverse outcomes at delivery.

**Study Design**: We conducted a serial cross‐sectional analysis of October‐December delivery hospitalizations with an AP admission in the preceding 9 months included in the National Readmissions Database during 2016‐2021. Our primary exposure was PDD (versus routine discharge) at first AP hospitalization. Using the chi‐square test, we evaluated the association between demographic and clinical factors and AP PDD. Using survey‐adjusted logistic regression, we evaluated the association between AP PDD and the following adverse outcomes at delivery: non‐transfusion severe maternal morbidity (SMM), transfusion, hypertensive disorders (HDP), preterm delivery (PTD) and stillbirth.

**Results**: We identified 152,748 AP hospitalizations with delivery follow‐up in NRD during 2016‐2021, with 3.3% of AP discharges categorized as PDD. Compared to routine discharge, AP PDD was associated with higher likelihood of any mental health condition (38.4% vs 27.0%; p< 0.01), any substance use disorder (44.4% vs 14.1%, p < 0.01), lowest income quartile (45.2% vs 36.0%; p< 0.01), and Medicaid insurance (78.8% vs 57.9%; p < 0.01). At delivery, history of AP PDD was associated with increased risk for SMM (adjusted OR [aOR] 1.42, 95% CI 1.16, 1.73), transfusion (aOR 1.63, 95% CI 1.32, 2.02), HDP (aOR 1.44, 95% CI 1.28, 1.61), PTD (aOR 1.45, 95% CI 1.29, 1.63) and stillbirth (aOR 1.80, 95% CI 1.39, 2.33).

**Conclusion**: About 3% of antepartum patients will self‐discharge. Patient‐directed discharge is more common in patients with adverse social determinants of health and mental health conditions. PDD is associated with higher risk of adverse delivery outcomes. Screening for PDD risk factors during antepartum admission and providing interventions to at‐risk patients may reduce adverse delivery outcomes.

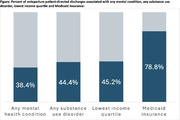

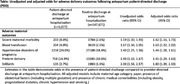

## 651 | Environmental Protection Agency (EPA) National Walkability Index: Does it Effect Weight Gain in Gdm Pregnancies?

Thomas Owens^1^; Erica Glaser^2^; Johanna Suskin^3^; Guillaume Stoffels^4^; Mia A. Heiligenstein^5^; Olivia Grubman^6^; Xiteng Yan^2^; Zainab Al‐Ibraheemi^1^; Lois Brustman^1^

*^1^Icahn School of Medicine at Mount Sinai West, New York, NY; ^2^Mount Sinai West, New York, NY; ^3^Mount Sinai West, New York City, NY; ^4^Icahn School of Medicine at Mount Sinai, New York, NY; ^5^Mount Sinai West, Astoria, NY; ^6^Westchester Medical Center, Westchester, NY*

4:00 PM ‐ 6:00 PM

**Objective**: An important factor in the management of gestational diabetes (GDM) is exercise. The primary aim of this study was to assess neighborhood walkability and weight gain from time of diagnosis of GDM, and total weight gain of the pregnancy.

**Study Design**: This is a retrospective cohort study of pregnant patients that were diagnosed with GDM at an academic center between 2018‐2023. Patients' addresses at the time of their gestation were correlated with the EPA National Walkability Index. The categories of walkability index included: least walkable(score 1‐5), below average(6‐10), above average(11‐15), and most walkable(16‐20). Descriptive statistics and restricted cubic spline plot were used. Multivariate analysis was conducted adjusting for median income, age, BMI, gravidity, parity, race, and hypertension.

**Results**: 1231 patients were identified. There were 81 patients(6.6%) in the least walkable group, 225 patients(18.3%) in the below average walkable group, 428 patients(34.8%) in the above average walkable group, and 497 patients(40.4%) in the most walkable group.The above‐average walkable group(scores 10.51 to 15.25) was defined as the reference group, as it included the median walkability score. In multivariable analysis, the total weight gain of patients in the least walkable group was, on average, 5.42 pounds greater compared to the reference group(p< 0.0001)(table 1). The relationship between walkability score and total weight gain was approximately linear(Fig. 1). In the adjusted linear trend analysis, each unit increase in walkability score was associated with a 0.40‐pound decrease in total weight gain (p< 0.0001)(table 1). Additionally, each unit increase in walkability score was associated with a 0.25‐pound decrease from time of GDM diagnosis(p< 0.0001).

**Conclusion**: This study suggests that patients with GDM who live in a lower walkability area are more likely to gain weight during their pregnancy. Therefore, it's important when providers are counseling patients with GDM to stress the relationship of exercise to weight gain and how that may impact blood glucose control.

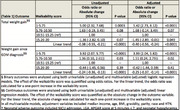

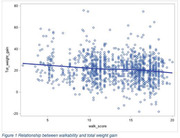

## 652 | Environmental Protection Agency (EPA) National Walkability Index and its Impact on the Severity of Gdm

Thomas Owens^1^; Johanna Suskin^2^; Erica Glaser^3^; Guillaume Stoffels^4^; Mia A. Heiligenstein^5^; Olivia Grubman^6^; Xiteng Yan^3^; Zainab Al‐Ibraheemi^1^; Lois Brustman^1^

*^1^Icahn School of Medicine at Mount Sinai West, New York, NY; ^2^Mount Sinai West, New York City, NY; ^3^Mount Sinai West, New York, NY; ^4^Icahn School of Medicine at Mount Sinai, New York, NY; ^5^Mount Sinai West, Astoria, NY; ^6^Westchester Medical Center, Westchester, NY*

4:00 PM ‐ 6:00 PM

**Objective**: An important factor in the management of gestational diabetes (GDM) is exercise. The aim of this study is to assess neighborhood walkability and need for hypoglycemic agents (HA) in patients with GDM.

**Study Design**: A retrospective cohort study of pregnant patients that were diagnosed with GDM at an academic center between 2018‐2023. Patients' addresses at the time of their gestation were correlated with the Environmental Protection Agency (EPA) National walkability index. The categories of EPA walkability index included:least walkable (score 1‐5), below average (6‐10), above average (11‐15), and most walkable (16‐20). A2GDM was defined as patients requiring HA. Secondary analysis included a neonatal composite outcome consisting of neonatal hypoglycemia(NH), large for gestational age(LGA), NICU admission, and APGAR score< 7. Descriptive statistics and restricted cubic spline plot (RP) were used. Multivariate analysis was conducted adjusting for median income, age, BMI, gravidity, parity, race, and hypertension.

**Results**: 1231 patients identified. There were 81 patients(6.6%) in the least walkable group, 225 patients(18.3%) in the below average walkable group, 428 patients(34.8%) in the above average walkable group, and 497 patients(40.4%) in the most walkable group. In multivariable analysis patients in the least walkable and below‐average walkable groups had a significantly higher likelihood of having A2GDM(least walkable: p = 0.004; below average walkable: p = 0.02 (Table 1)). A RP showed that the relationship between walkability score and the probability of A2GDM was linear(Fig. 1).When walkability score was treated as continuous, each one‐unit increase in walkability score was significantly associated with a 6% decrease in the odds of A2GDM(p = 0.0001). Neonatal composite did not differ between the groups(p = 0.30).

**Conclusion**: Patients who live in a higher walkability area are less likely to need medication. Thus, providers counseling patients with GDM should stress the importance of exercise in the control of blood glucose and its relationship to the severity and subsequent management.

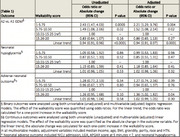

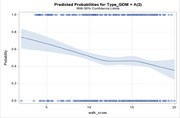

## 653 | Does Early Administration of IV Iron During Pregnancy Improve Maternal Morbidity?

Tina M. Bui; Guillermo Valenzuela; Kristina Roloff; Mary Tsaturian; Phoebe Jen

*Arrowhead Regional Medical Center, Colton, CA*

4:00 PM ‐ 6:00 PM

**Objective**: Anemia in pregnancy is a significant contributor to maternal morbidity, including increased risks of blood transfusion, longer hospital stays, depression, preterm birth, and low birth weight. Oral (PO) iron has been traditionally recommended for its simplicity and cost‐effectiveness but often suffers from poor adherence and gastrointestinal side effects. This study aims to investigate whether early administration of IV iron can improve hemoglobin (Hgb) levels prior to delivery and decrease the need for blood transfusion.

**Study Design**: Patients with a ferritin level < 30 ng/mL were recruited. Exclusion criteria included age < 18, multifetal gestations, thalassemia or sickle cell disease, and chronic kidney or liver disease. After obtaining consent, patients were randomly assigned to either the PO or IV iron groups. The PO iron group received Ferrous sulfate 325 mg every other day, while the IV iron group received Venofer 200 mg every other day until the iron deficit was corrected. Hgb and ferritin levels were assessed before treatment and four weeks after treatment initiation. Patients also completed an anemia symptom questionnaire at enrollment and at the 4‐week mark. Statistical comparisons were conducted using two‐tailed T‐tests with significance set at P < 0.05.

**Results**: The IV iron group showed a significant increase in Hgb and ferritin levels compared to the PO iron group four weeks after treatment, with P‐values of 0.002 and 0.0001, respectively. The blood transfusion rate was 3.7% in the PO iron group and 0% in the IV iron group.

**Conclusion**: Our findings demonstrate a significant improvement in ferritin levels among patients treated with IV iron compared to those receiving PO iron therapy. Additionally, the absence of blood transfusions in the IV iron group highlights its potential in reducing alloimmunization and blood transfusion reactions, suggesting that early administration of IV iron during pregnancy can improve maternal health outcomes.

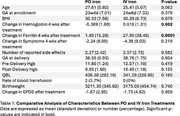

## 654 | Perceived Racism and Placentally Mediated Adverse Pregnancy Outcomes

Joy McNeal; Sarah Heerboth; Ashlyn Tolbert; Johanna Quist‐Nelson; Rebecca Fry; Tracy A. Manuck

*University of North Carolina, Chapel Hill, NC*

4:00 PM ‐ 6:00 PM

**Objective**: Racism is consistently implicated in obstetric disparities and is associated with hypertension (HTN) among non‐pregnant individuals. We sought to quantify the extent to which racism, including its characteristics (domains, timing), is associated with placentally‐mediated pregnancy complications (plac‐comps).

**Study Design**: Primary analysis of a prospective cohort. Participants identifying as Black, White, and/or Hispanic, with singleton, non‐anomalous gestations were recruited < 22 weeks, 2017‐2022, and completed the validated Perceived Racism Scale at enrollment to assess any‐time (at any time during life) and recent (within the last year) racism in 11 public/personal, 3 public/observed experience, and 7 employment domains. The primary outcome was a diagnosis of plac‐comps (HTN disorders of pregnancy, birthweight < 10% for GA and sex, ± placental abruption).

**Results**: 414 individuals (47% Black, 42% White, 11% Hispanic) were included; 93 (22.4%) had plac‐comps. Surveys were completed at a mean of 17.3 (IQR 16.1‐20.0) wks. Clinical factors are shown in Table 1. Compared to those without plac‐comps, those with plac‐comps were more likely to experience racism at any time (65% vs. 47%, p = 0.02) and racism across a greater number of domains any‐time (8.1 v. 5.6, p = 0.002) and recently (mean 4.6 v. 2.6, p< 0.001). Differences were most pronounced in public/personal domains, as those with plac‐comps had higher any‐time perceived racism in 7 of 11 public/personal domains and higher recent perceived racism in 7 of 11 public/personal domains (all p< 0.05), Figure. In regressions, each additional any‐time racism domain conferred a 9% increased odds of plac‐comps (aOR 1.09, 95% CI 1.03‐1.15). Similarly, each additional recent racism domain conferred a 8% increased odds of plac‐comps (aOR 1.08, 95% CI 1.03‐1.13).

**Conclusion**: Among pregnant individuals at high‐risk for adverse pregnancy outcomes, racism is present across multiple facets of daily life. Perceived racism was associated with an increased risk of adverse placental outcomes, irrespective of the timing of the exposure.

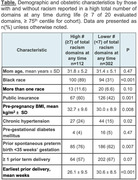

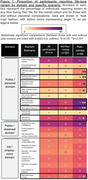

## 655 | Objective Quantification of Sleep Throughout Pregnancy, Preterm Birth, and Delivery Gestational Age

Emily Gascoigne^1^; Megan Ragusa^2^; Ashlyn Tolbert^2^; Annie Dude^2^; Rebecca Fry^2^; Tracy A. Manuck^2^

*^1^UNC Chapel Hill School of Medicine, Carrboro, NC; ^2^University of North Carolina, Chapel Hill, NC*

4:00 PM ‐ 6:00 PM

**Objective**: Sleep quality and quantity (evaluated using traditional duration/stage measures and novel metrics including the sleep regularity index, a parameter assessing sleep pattern regularity and consistency) are associated with health outcomes. We sought to determine which sleep parameters are associated with PTB.

**Study Design**: Primary analysis of a prospective cohort enriched for those at high *a priori *risk of PTB. Participants identifying as Black, White, and/or Hispanic, with singleton, non‐anomalous gestations were recruited < 14 weeks, 2017‐2022. Participants were provided FitBit wristband activity trackers at enrollment and instructed to maintain their usual activity. Those with ≥7 nights of sleep monitoring (verified by FitBit recorded heart rate) were included. The primary outcomes were delivery gestational age and PTB < 35 weeks.  We evaluated individual sleep parameters and a composite ‘poor sleep’ score based on previously established metrics of sleep health. The poor sleep score was calculated by adding +1 point for (1) mean total min/asleep < 360 min/night; (2) mean % time in REM < 20%; (3) mean sleep efficiency (time asleep/time in bed) < 85%; (4) >50% of sleep midpoints ≥ 05:00; (5) ≥ 2 median night awakenings; (6) sleep regularity index in lowest quartile (< 95.2). Cox regression models included adjustments for multiple and differing numbers of observations per subject.

**Results**: 154 individuals were included, delivering at a median 38.2 (IQR 37.0, 39.3) weeks; 25% had PTB < 37 and 14% PTB < 35 weeks. The median sleep score was 2 (IQR 1,3); range 0‐5. Clinical characteristics are shown in Table 1. Each sleep parameter's variation over gestation is shown in Figure 1. In cox regression models, a poor sleep score (≥3; aHR 1.49, 95% CI 1.04, 2.13) and % time in REM sleep (aHR 0.21, 95% CI 0.06‐0.73) were associated with delivery gestational age.

**Conclusion**: Several sleep metrics vary across gestation, though tend to be similar among those with PTB < 35 weeks vs. those with later delivery. However, individuals with ≥3 poor sleep habits throughout pregnancy carried a higher risk of early delivery.

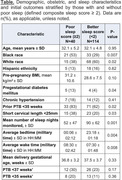

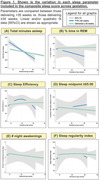

## 656 | Impact of Low‐Dose Furosemide on Breastfeeding and Newborn Weight: a Randomized Controlled Trial Secondary Analysis

Ukachi N. Emeruwa^1^; Kysha Mercader^2^; Chaukim Peters^3^; Shai Bejerano^4^; Toluwalasé A. Ajayi^5^; Gregory A. Aarons^6^; Bonnie Kaiser^6^; Sarah Averbach^6^; Michelle Leff^6^; Russell S. Miller^4^; Cynthia Gyamfi‐Bannerman^1^

*^1^University of California, San Diego, San Diego, CA; ^2^University of California, San Diego School of Medicine, La Jolla, CA; ^3^St. George's University School of Medicine, St. George's, Virgin Islands, U.S.; ^4^Columbia University Medical Center, New York, NY; ^5^University of California San Diego/Rady Children's Hospital, San Diego, CA; ^6^University of California San Diego, La Jolla, CA*

4:00 PM ‐ 6:00 PM

**Objective**: Growing evidence supports loop‐diuretic use to reduce postpartum (PP) hypertensive morbidity, but little is known about how these medications affect breastfeeding and associated neonatal outcomes. We aimed to explore breastfeeding rates and newborn weight loss among lactating patients taking low‐dose furosemide after delivery.

**Study Design**: This was a secondary analysis of the Lasix for the Prevention of de novo Postpartum Hypertension Trial (PMID: 38641089), which randomized 82 normotensive patients at high risk for de novo PP hypertension to 5 days of daily furosemide 20mg PO or placebo within 8 hours after delivery. Parent trial participants completed electronic surveys at 2 and 6 weeks PP, which included infant feeding practice questions. Data on feeding practices and neonatal weight were also abstracted from electronic medical records. The primary outcome of this analysis was exclusive breastfeeding (EBF) continuation rates among those who reported EBF intent on delivery admission. Secondary outcomes included EBF rates among those practicing EBF at discharge, any breastfeeding rates, formula supplementation reasons, and newborn delivery to discharge weight change.

**Results**: Of 79 parent trial participants enrolled through 6 weeks PP, 70 (89%) at least partially completed the 2 week survey; 55 (70%) at least partially completed the 6 week survey. There was no difference in EBF continuation rates for participants randomly assigned to furosemide who intended to exclusively breastfeed (54% at 2 weeks; 64% at 6 weeks) compared to placebo (38% at 2 weeks, p = 0.43; 31% at 6 weeks, p = 0.11). (**Figure 1**) Of reported supplementation reasons, there was no difference in milk supply or neonatal concerns between groups. Newborn weight declined a median of 5% [IQR ‐7,‐3%] in the furosemide group and 6% [IQR ‐8,‐4%] in the placebo group (p = 0.17) by discharge, consistent with physiologic norms. (**Table 1**)

**Conclusion**: There is no evidence in our trial that low‐dose furosemide impacts breastfeeding continuation rates or physiologic newborn weight loss. These data are reassuring for loop‐diuretic use in lactating people.

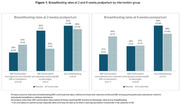

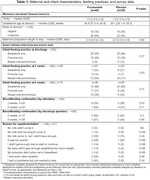

## 657 | Automated Unattended Office BP: Is Your Clinician Raising Your Blood Pressure?

Whitney A. Booker^1^; Patrick Conley^2^; Marissa Petralia^2^; Jeannette Pacheco^2^; Eliza C. Miller^3^; Uma M. Reddy^4^; Ying Kuen K. Cheung^3^; Mariam Naqvi^5^; Natalie A. Bello^5^

*^1^Columbia University, new York, NY; ^2^Cedars Sinai Medical Center, Cedars Sinai, CA; ^3^Columbia University Medical Center, New York, NY; ^4^Columbia University, New York, NY; ^5^Cedars Sinai Medical Center, Los Angeles, CA*

4:00 PM ‐ 6:00 PM

**Objective**: There is emerging data that in non‐pregnant adults measuring clinic blood pressure (CBP) without a clinician present (unattended CBP) may reduce the “white coat effect” and be closer to mean out‐of‐clinic blood pressure (BP) compared to traditional CBP measurements that involve a clinician in the room (attended CBP).  Our objective was to compare unattended CBP with attended CBP in pregnant women with hypertension (HTN).

**Study Design**: Pregnant patients with a history of chronic or gestational HTN defined using ACOG diagnostic criteria were included in this analysis. Demographic variables were described as median [IQR] for continuous variables and frequencies for categorical variables. Automated attended and unattended CBP were measured at up to two prenatal visits and compared using a paired t‐test to account for repeated measures and between‐subject variability. Bland‐Altman plots were created to evaluate the agreement between unattended and attended CBP.

**Results**: Of 26 pregnant patients with HTN, women were on average 36.5 [5.1] years old and 24 weeks pregnant, with a BMI of 32.2 [8.3] kg/m^2^, 19% had diabetes, and the majority were working full time (**Table**). In 49 paired measurements, the mean unattended CBP was 115.8/72.3 mmHg and the mean attended CBP was 116.5/71.5 mmHg. The mean difference (95% CI) between unattended and attended systolic BP was 0.69 (‐1.40, 2.78) and diastolic BP was 0.00 (‐1.02, 1.02); both mean differences were not statistically significant P >0.5 (**Figure**).

**Conclusion**: In pregnant patients with HTN, having a provider present during CBP measurements did not result in a statistically significant BP elevation. Based on these findings unattended CBP measurement may not be able to replace out‐of‐office BP measurement for the diagnosis of white coat hypertension; further research is needed.

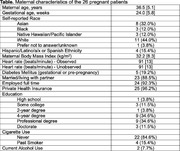

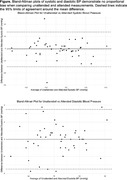

## 658 | Differential Expression of Placental CD300 Receptors in Preeclampsia (PE) and Preterm Birth (PTB)

William E. Ackerman, IV^1^; Meera M. Thakkar^1^; Guomao Zhao^1^; Zoe B. Strong^1^; Elizabeth Feoktistov^2^; Ekaterina Snegovskikh^2^; Catalin S. Buhimschi^1^; Irina A. Buhimschi^1^

*^1^University of Illinois at Chicago, College of Medicine, Chicago, IL; ^2^University of Illinois at Chicago, Chicago, IL*

4:00 PM ‐ 6:00 PM

**Objective**: Through their binding to lipids and other ligands, the 7 member CD300 family of receptors is poised to have a significant role in biological processes with an inflammatory component. Their roles in major inflammatory obstetrical syndromes such as PE and PTB +/‐ Triple I have not been fully elucidated. We performed a targeted computational analysis of existing placental datasets with transcriptional profiling to understand the expression and distribution of placental CD300 receptors in these pathological contexts.

**Study Design**: We interrogated bulk and single‐cell (sc) placental RNA sequencing (RNAseq) datasets (GEO: GSE73714, GSE203507, GSE114691, GSE173193; Figshare: 23264102) for CD300 expression in PE (n = 65) and PTB (NO‐Triple I n = 26; YES‐Triple I, n = 5). This analysis was complemented by RNAseq in a new set of 8 fetal membrane (FM) samples as follows: YES‐Triple I (n = 4, GA 28±3w), NO‐Triple I (n = 4, GA 32±1w). Selected targets were cross validated by qPCR.

**Results**: Across placental RNAseq datasets, all 7 CD300 mRNAs could be detected, with *CD300LG* predominating in the villous placenta and *CD300A* and *‐E* most abundant in FM. By scRNAseq, *CD300LB*, *‐E*, and *‐LF* displayed myeloid‐limited expression, *CD300A* was expressed in myeloid and lymphoid cells, *CD300LG* was abundant in vascular endothelial cells, and *CD300LC* and *‐D* expression fell below detection limits. Villous *CD300LG* was significantly more abundant in PE than non‐PE samples (FDR< 0.05). In Triple I, *CD300A*, *‐LB*, *‐LD*, *‐E*, *‐LF*, and *‐LG* were elevated (FDR< 0.05) in FM but not villous placenta by RNAseq, with CD300LF being most significant. Increased expression of placental *CD300LG* in PE and FM *CD300LF* in Triple I were confirmed by qPCR (p< 0.05).

**Conclusion**: The CD300 family of receptors is present in the human placenta. In pregnancy conditions with strong inflammatory components the signaling pathways emanating from this family of receptors are likely diverse based on disease and their location.

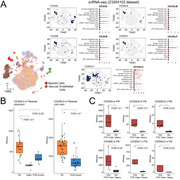

## 659 | Early Recognition of Fetal Growth Restriction via Clinical Features, Metabolomic Profiles and Polygenic Risk Scores

Xiaoqing He^1^; Jun Zhang^2^; Yun Huang^2^

*^1^Ministry of Education‐Shanghai Key Laboratory of Children's Environmental Health, Xinhua Hospital, affiliated with School of Medicine, Shanghai Jiao Tong University, Shanghai, China, Shanghai, Shanghai; ^2^Shanghai Jiao Tong University School of Medicine, Shanghai, Shanghai*

4:00 PM ‐ 6:00 PM

**Objective**: To integrate various factors to predict FGR, enabling early identification and risk stratification of high‐risk populations.

**Study Design**: This study was conducted based on a prospective birth cohort. A total of 1,739 mother‐child pairs were included. Three nested case‐control studies were further conducted: 1) 58 FGR cases (< 3rd percentile); 2) 78 X5th cases (< 5th percentile); 3) 180 SGA cases (< 10th percentile). The control group included 679 healthy controls (BW in the 25th to 75th percentile, without major complications). We developed 6 risk prediction models for birth outcomes (FGR, X5th, and SGA) using clinical risk factors, life factors, maternal and fetal polygenic risk scores (PRSs), and maternal plasma metabolic profiles in early pregnancy.

**Results**: Eight clinical factors and two life factors (health behaviors and diet class) were included. Both maternal and fetal PRSs were derived from the 2019 GWAS meta‐analysis for birth weight from the EGG consortium. Univariate analysis and the Unbiased Variable selection in R (MUVR) package identified potential metabolomic biomarkers. For the three case‐control groups, 6, 7, and 10 metabolites were identified as potential biomarkers for FGR, X5th, and SGA, respectively.

The final model based on 8 clinical features, 2 life factors, maternal and fetal PRSs, and the 6 metabolite sets demonstrated the best predictive ability, achieving a maximum AUC of 0.826 (95% CI: 0.763‐0.879) for predicting FGR. A model built solely on early pregnancy data achieved a maximum AUC of 0.744 (95% CI: 0.681‐0.806) for predicting FGR.

**Conclusion**: The inclusion of metabolic and genetic information significantly enhanced predictive performance compared to traditional models. Integrating genetic and metabolic data enhances the accuracy of fetal growth assessments, enabling earlier and more precise identification of high‐risk pregnancies and facilitating timely interventions to improve outcomes.

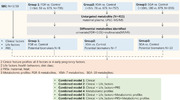

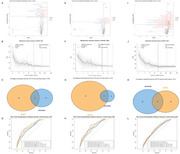

## 660 | Risk Factors for Adverse Outcomes in Uterine Conserving Cesarean Deliveries for Placenta Accreta Spectrum

Yonatan Dror^1^; Elias Castel^2^; Raanan Meyer^3^; Lior Fridrich^2^; Nizan Mor^2^; Aviran Ohayon^2^; Michal Axelrod^4^; Alina Weissmann‐Brenner^2^; Gabriel Levin^5^; Shlomi Toussia Cohen^2^

*^1^Chaim Sheba Medical Center, Tel‐Hashomer, Ramat‐Gan, Israel, Chaim Sheba Medical Center, Tel‐Hashomer, Ramat‐Gan, Israel, HaMerkaz; ^2^Sheba Medical Center, Ramat Gan, HaMerkaz; ^3^Cedars Sinai Medical Center, Los Angeles, CA; ^4^Department of Obstetrics and Gynecology, Sheba Medical Center, Tel HaShomer, Ramat Gan, HaMerkaz; ^5^McGill University, Montreal, PQ*

4:00 PM ‐ 6:00 PM

**Objective**: While uterine conserving cesarean delivery for placenta accreta spectrum (PAS) has become more common, data on outcomes are still limited. The aim of this study is to describe the characteristics of patients undergoing uterine conserving cesarean for PAS with and without adverse maternal outcomes, and identify risk factors for their occurrence.

**Study Design**: A retrospective cohort study conducted at a single tertiary center between 03/2011 and 01/2022. Women with PAS who underwent uterine conserving cesarean delivery were included. Women with scheduled cesarean hysterectomy were excluded. A composite of adverse maternal outcome was defined as the occurrence of at least one of the following: bladder injury, ureteral injury, bowel injury, ≥6 units of blood transfusion, postoperative fever, unplanned cesarean hysterectomy, relaparotomy, intensive care unit admission and hospital readmission.

We compared patients with and without the composite adverse outcome.

Multivariable regression analysis was used to identify factors associated with the composite of adverse outcome.

**Results**: 238 women with PAS were included in the study group, 70 had composite adverse maternal outcome and 168 did not. The maternal adverse outcome group had earlier gestational age at delivery (median 36^0/7^ vs. 36^3/7^ weeks), higher preoperative uterine rupture rates (6.2% vs. 0.6%), longer cesarean delivery duration (median 118.5 vs. 68.0 minutes), more drainage placement (72.5% vs. 23.8%) and higher estimated blood loss (median 2500.0 vs. 1200.0 ml).

In multivariable logistic regression analysis, earlier gestational age at delivery, and the sonographic findings of lacunae, bridging vessels and loss of clear zone were independently associated with the composite adverse maternal outcome.

**Conclusion**: Among women undergoing uterine conserving cesarean delivery for PAS, an earlier gestational age at delivery, and three sonographic findings, are associated with adverse maternal outcomes.

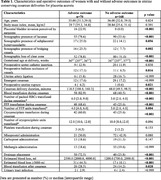

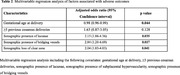

## 661 | Maternal Outcomes Associated with Antepartum versus Postpartum Eclampsia

Yossi Bart^1^; Farah H. Amro^1^; Ghamar Bitar^1^; Sandra Sadek^2^; Sean C. Blackwell^1^; Baha M. Sibai^1^

*^1^McGovern Medical School at UTHealth Houston, Houston, TX; ^2^University of Texas Health Science Center in Houston, McGovern Medical School, Houston, TX*

4:00 PM ‐ 6:00 PM

**Objective**: The data on eclampsia‐related outcomes in the literature relies mainly on studies performed over two decades ago. Our objective was to compare maternal outcomes associated with the timing of eclampsia using a contemporary dataset.

**Study Design**: We conducted a retrospective cohort study of all patients diagnosed with eclampsia between June 2011 and June 2024 at a tertiary medical center. Patients with antepartum eclampsia (AE) were compared to those with postpartum eclampsia (PPE). The primary outcome was defined as a composite maternal morbidity including stroke, neurologic deficits at discharge, cardiac arrest, myocardial infarction, liver infarction or rupture, pulmonary edema, acute kidney injury, HELLP, disseminated intravascular coagulation, or death. Maternal outcomes were reported using Chi‐Square or Fisher's exact tests.

**Results**: Overall, 80 patients met the inclusion criteria; 39 (49%) had AE, and 41 (51%) had PPE. Baseline maternal characteristics (age, race, BMI, chronic hypertension, diabetes mellitus) were similar between the groups. Four out of every five patients reported symptoms before the seizure, including headache (70%) and visual symptoms (31%), evenly distributed between groups. Simultaneous severe range blood pressure at the time of eclampsia diagnosis was reported in 52 (73%) of the patients, with similar rates between groups. PPE occurred >2 days after delivery in 90% of the cases and >7 days after delivery in 32%. Patients with AE had higher rates of composite maternal morbidity (59.0% vs. 31.7%, p = 0.01), driven mainly by higher rates of acute kidney injury and hemolysis, elevated liver enzymes, and low platelets syndrome (Table). There were also higher rates of post‐seizure intubation (30.8% vs. 7.3%, p = 0.01) and intensive care unit admission (51.3% vs. 26.8%, p = 0.02) among patients who had AE.

**Conclusion**: Eclampsia continues to serve as an important contributor to maternal morbidity and mortality nowadays. Compared to postpartum eclampsia, antepartum eclampsia was associated with higher rates of maternal morbidity.

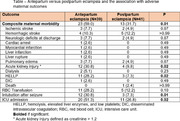

## 662 | Do Maternal Outcomes Differ by Specific Blood Pressure Parameters?

Yossi Bart^1^; Hector M. Mendez‐Figueroa^2^; Farah H. Amro^1^; Baha M. Sibai^1^

*^1^McGovern Medical School at UTHealth Houston, Houston, TX; ^2^McGovern Medical School at UTHealth, Houston, TX*

4:00 PM ‐ 6:00 PM

**Objective**: The role of severe hypertension (sHTN) based on isolated severe systolic or diastolic blood pressure has not been well studied. We aimed to evaluate adverse maternal outcomes in those with isolated sHTN to those with both severe systolic and diastolic hypertension.

**Study Design**: We conducted a secondary analysis of the Assessment of Perinatal Excellence database. All the patients who had sHTN at the time of delivery, defined as systolic BP (SBP) ≥ 160 mmHg and/or diastolic BP (DBP) ≥ 110 mmHg on two occasions at least 30 minutes apart were included. Individuals with isolated SBP or DBP in the severe range (iSRBP) were compared to those with both (bSRBP). The primary outcome was defined as a composite of maternal outcomes, including hypertensive stroke, pulmonary edema, acute kidney injury, disseminated intravascular coagulation, cardiopulmonary arrest, and death. Multivariate logistic regression was applied to address confounders.

**Results**: A total of 7,788 individuals met the inclusion criteria. Of those, 5,968 (77%) had isolated SBP ≥ 160, 272 (3%) had isolated DBP ≥ 110 mmHg, and 1,548 (20%) had both. In comparison to those with bSRBP, individuals with iSRBP were older, had higher rates of obesity and multifetal gestation, and lower rates of chronic hypertension. Compared to bSRBP, those with iSRBP had lower composite outcome rates (adjusted relative risk 0.44, 95% CI 0.34‐0.57), driven mainly by lower rates of pulmonary edema and acute kidney injury (Table). Intensive‐care unit admission rate was also lower among individuals with iSRBP. In a sub‐analysis of iSRBP, comparing individuals with isolated SBP vs. isolated DBP in the severe range, no differences in maternal outcomes were noted. Another comparison following further stratification by blood pressure showed a lower composite outcome rate among those with iSRBP compared to bSRBP when SBP was ≥ 180 mmHg (Figure).

**Conclusion**: Isolated severe systolic or diastolic hypertension was associated with lower rates of maternal morbidity compared to sHTN with both. This was primarily driven by lower rates of acute kidney injury and pulmonary edema.

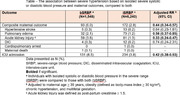

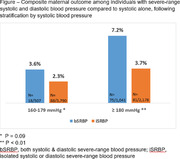

## 663 | Doulas In Black Birthing Experiences: Perceptions And Utilization Among Black Participants Following Doula‐led Education Initiative

Yuliya Faryna^1^; Tonya Simone Wright^2^; Melissa Haslam^2^

*^1^Pennsylvania State College of Medicine, Hershey, PA; ^2^Penn State College of Medicine, Hershey, PA*

4:00 PM ‐ 6:00 PM

**Objective**: ACOG and CDC endorse doula use as an evidence‐based practice to narrow disparities in Black maternal health outcomes. Despite their benefit, doulas remain underutilized, particularly in Black birthing experiences. Our objective is to assess baseline doula awareness, knowledge, and utilization among Black birthing people and evaluate changes in their attitudes toward integrating doulas into obstetric spaces following an education initiative.

**Study Design**: During a Black maternal health community initiative, three Black doulas led 15‐minute education sessions with small groups. Surveys were given to assess participant knowledge and awareness of doula care, pre‐ and post‐doula education sessions (pre‐DES and post‐DES). Inclusion criteria were Black participants of reproductive age. Data were analyzed using paired t‐tests and McNemar's tests; p‐values < 0.05 were considered significant.

**Results**: Fifteen participants met criteria. All reported no previous doula use or physician‐dispensed doula education. Pre‐DES, 40% felt well‐informed about doulas, compared to 87% post‐DES, p = 0.04. Participants knew an average of 4.1 doula health benefits pre‐DES and 6.5 benefits post‐DES, p = 0.0007 (Graph 1). This mirrors the 54% increase in people who felt more well‐informed about what doula care entails post‐DES, p = 0.005 (Graph 2). There was no significant difference in number of cited barriers to doula use pre‐ vs. post‐DES, p = 0.31, but the main barrier changed from not knowing how to access doulas (N = 7) to lacking doula insurance coverage (N = 9). Lastly, 87% indicated intent to use doulas in the future.

**Conclusion**: While baseline knowledge of doula health benefits was high, DES still increased participant knowledge and intent to use doulas in future pregnancies. However, their utilization barriers reveal that increasing doula use among Black birthing people will require an equity framework for better integration of doulas into our health system. Reform is needed in medical trainee education on doula care and patient resourcing, health policy, and legislation for equitable doula insurance coverage and reimbursement.

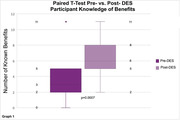

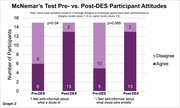

## 664 | Variations in MFM Fellowship Clinical Competency through Curriculum Requirements Surpassing ACGME/ABOG Minimums by Geographic Region

Zenobia Gonsalves^1^; Rachel Lee^2^; Chaitali Korgaonkar‐Cherala^3^; Kimberly Herrera^4^; Cassandra Heiselman^1^

*^1^Stony Brook University Hospital, Stony Brook, NY; ^2^Stony Brook University Hospital, Lake Grove, NY; ^3^Stony Brook University Hospital, Stonybrook, NY; ^4^Stony Brook University, Stony Brook, NY*

4:00 PM ‐ 6:00 PM

**Objective**: To evaluate curricular differences across geographic regions for fellowship programs by assessing the variations in the distribution of specific types of rotations above the minimum core MFM requirements as defined by ACGME and ABOG.

**Study Design**: This was a cross‐sectional analysis of MFM fellowship curricula conducted in July 2024 for the 109 ACGME‐accredited programs. All fellowship program websites were evaluated for rotation schedules. If not available online, programs were contacted via email. The total number of mandated months by rotation for each program were tallied across the three years of fellowship: supervisory L&D (min: 2 mo), inpatient/antepartum service (no minimum), outpatient MFM clinic (min: 2 mo), ultrasound (min: 3 mo), genetics/genomics (min: 2 mo), research (min: 12 mo), electives, and program‐specific mandated rotations. For each rotation, the percentage of programs that exceeded ACGME/ABOG minimum duration requirements were compared across geographic regions (Northeast, Midwest, South, and West). Chi‐square and Fisher's exact analyses were performed with a p‐value < 0.05. 

**Results**: Rotation schedules were available for 64 (59%) of programs. The percentage of total programs that exceeded minimums were 87% for outpatient, 83% for ultrasound, 31% for research, 28% for labor and delivery, and 12% for genetics. When comparing the percentage of programs surpassing minimums by geographic region, only ultrasound requirements differed across regions (Figure 1), specifically 100% of Northeast programs exceeded ultrasound requirements versus 44% in the South (p = 0.006). Twenty programs (31%) specified requirements for non‐OB, non‐ACGME/ABOG rotations, most frequently NICU, anesthesia, and pathology (Table 1). Of these programs, anesthesia was more frequently required in the South (p = 0.013). 100% of programs offered flexible elective time.

**Conclusion**: While there are curricular differences among individual MFM fellowship programs, variability above minimum requirements was only noted for ultrasound and non‐obstetric rotations across geographic regions.

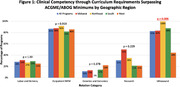

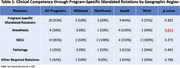

## 665 | Developing Core Endpoints with Stakeholders for Perinatal Clinical Trials

Zoe Butters^1^; Jannik Aagerup^2^; Clare Whitehead^3^; Lene Seidler^2^

*^1^Royal Women's Hospital, Melbourne, Victoria; ^2^NHMRC Clinical Trials Centre, University of Sydney, Sydney, New South Wales; ^3^Royal Women's Hospital, University of Melbourne, Melbourne, Victoria*

4:00 PM ‐ 6:00 PM

**Objective**: The use of core outcome sets (COS) in obstetric and perinatal clinical trials is essential to standardise trial reporting and allow evidence synthesis, but there has been limited assessment of how these outcomes were developed and how they can be used to compare endpoints in clinical trials. Identifying the outcomes that are most meaningful to patients, healthcare professionals and society and standardising their definitions will ensure trial endpoints can be reliably collected and allow results to more rapidly lead to changes in clinical practice.

**Study Design**: MEDLINE, Embase, PsycINFO, CINAHL and the COMET registry were systematically searched in September 2023. Two authors independently screened abstracts/full texts and performed data extraction. A third reviewer resolved conflicts. The characteristics of each COS, stakeholders and consensus methods were extracted. The development methodology quality was evaluated according to the COS‐STAR statement.

**Results**: 789 studies were screened and 29 COS included. The top 5 reported obstetric outcomes were maternal mortality, patient satisfaction with intervention, adverse outcome of intervention, delivery mode, and breastfeeding at discharge. The top 5 neonatal outcomes were neonatal mortality, gestational age at birth, birthweight, congenital anomaly, and hypoxic ischaemic encephalopathy. Only 59% of COS demonstrated compliance with COS‐STAR. 100% included clinician/expert stakeholders. 90% included patients or patient representative stakeholders, although the proportion varied between 4.1% to 81.6%. Standardised definitions and measurement tools are proposed for each of the top 10 outcomes.

**Conclusion**: We have identified a minimum COS with standardised definitions and measurement tools that can be included in perinatal trials in order to improve the quality of endpoint reporting and implementation. Future research will rank outcomes according to both clinician and patent preference, to develop an ordinal scale that improves trial efficiency and identification of clinically meaningful evidence.

